# Stereoinvertive Nucleophilic Substitution at Quaternary Carbon Stereocenters of Cyclopropyl Ketones and Ethers

**DOI:** 10.1002/anie.202203673

**Published:** 2022-05-11

**Authors:** Xu Chen, Ilan Marek

**Affiliations:** ^1^ Schulich Faculty of Chemistry Technion-Israel Institute of Technology Technion City Haifa 3200009 Israel

**Keywords:** Azidation, Cyclobutonium, Cyclopropane, Nucleophilic Substitution, Stereoinvertive

## Abstract

A highly regio‐ and diastereoselective nucleophilic substitution at the quaternary carbon stereocenter of cyclopropyl ketones and cyclopropyl carbinol derivatives using TMSBr, DMPSCl and TMSN_3_ as nucleophiles has been developed. A variety of acyclic tertiary alkyl bromides, chlorides and azides were therefore prepared with excellent diastereopurity. The substitution occurs at the most substituted quaternary carbon center in a stereoinvertive manner, which may be attributed to the existence of a bicyclobutonium species.

## Graphical abstract

A Lewis‐acid catalyzed stereoinvertive nucleophilic substitution at the quaternary carbon stereocenter of cyclopropyl ketones and cyclopropyl carbinol derivatives is reported. A variety of tertiary alkyl bromides, chlorides and azides are prepared in good yields with excellent stereopurity via a postulated bicyclobutonium intermediate.

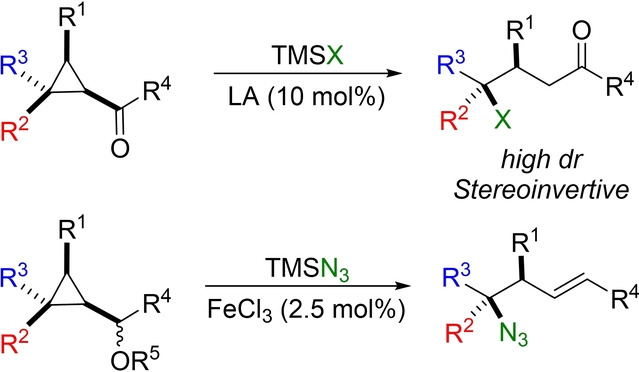

Nucleophilic substitution (S_N_1 or S_N_2) is a fundamental textbook reaction in organic chemistry, which has proved to be an extremely useful strategy for the construction of carbon–carbon or carbon–heteroatom bonds.^[1]^ In a S_N_2 reaction, “backside attack” at the electrophilic carbon center by the nucleophile with simultaneous departure of the leaving groups occurs usually with predictable inversion of configuration (Scheme 1a).^[2]^ One intrinsic limitation of stereoinvertive S_N_2 reaction is that it is generally sensitive to steric hindrance and albeit a few reported examples,^[3]^ realization of stereospecific S_N_2 reaction at tertiary carbon center still remains a difficult task.^[4]^ On the other hand, S_N_1 reaction is an attractive way to achieve this goal considering that the formed tertiary carbocation is insensitive to steric hindrance.^[5]^ However, carbocation can undergo undesired elimination and rearrangement pathways in preference to normal substitution. Furthermore, the continuum of intermediates ranging from intimate ion pairs, solvent separated ion pairs to fully dissociated ions make the stereoselective nucleophilic substitution rather challenging (Scheme 1a).^[6]^

**Scheme 1:**
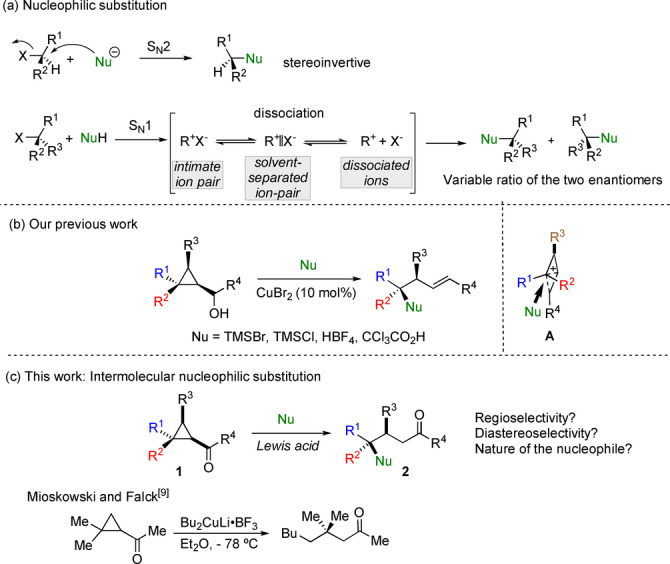
Nucleophilic substitution.

While the counterintuitive selective nucleophilic substitution at quaternary carbon stereocenter seems even more complicated as it would involve the cleavage of a carbon‐carbon bond, we have recently reported an efficient strategy demonstrating the possibility of nucleophilic substitution at the quaternary carbon stereocenter of cyclopropyl carbinol derivatives. The acyclic products were obtained as a single diastereomer with a complete inversion of configuration at quaternary carbon stereocenter (Scheme 1b).^[7]^ A bicyclobutonium species **A** was proposed as a possible transient intermediate, although the direct reaction at the quaternary carbon stereocenter of cyclopropylcarbinyl intermediate could not be excluded. Albeit it paved the way to the stereospecific nucleophilic substitution at congested quaternary carbon stereocenter, the reaction was restricted to halogen and carboxylic acid nucleophiles. One important addition would also be the addition of nitrogen‐based nucleophiles.

In addition, we were also wondering whether different sources of β‐cyclopropyl carbocation could be used, that would provide different final acyclic molecular backbones possessing synthetically interesting functionalities. We were particularly interested by the reactivity of cyclopropyl ketone, an important building block in organic synthesis,^[8]^ that would provide the expected acyclic ketone that could potentially be subsequently functionalized (Scheme 1c). However, our mechanistic scenario was opposite to the reported homo‐conjugate nucleophilic addition to cyclopropyl ketone occurring only on the least substituted carbon center (Scheme 1c, Mioskowski and Falck).^[9]^ To have an answer on this potential discrepancy,^[10]^ we started our study by preparing various polysubstituted cyclopropyl ketones **1** via our previously reported copper‐catalyzed carbomagnesiation reaction of cyclopropenes^[11]^ followed by reaction with various acyl chlorides, except for **1 n** that results from the transformation of the ester into a ketone and **1 o** where DMF was used instead of acyl chloride (Scheme 2 and Supporting Information). All cyclopropyl ketones were obtained in high yields with excellent diastereoselectivities (*dr*>95 : 5 : 0 : 0). With an easy access to cyclopropyl ketones in hand, we then tested our model substrate **1 a** in the bromination reaction using Me_3_SiBr as nucleophile in DCM as solvent. To our delight, the desired product **2 a** could be obtained with an excellent diastereoselectivity (*dr*>95:05) with no detectable presence of different isomers but in a moderate 56 % yield. Various Lewis acid and conditions were then tested (see Supporting Information for all details) and the optimized condition consists of using 10 mol % of Yb(OTf)_3_ in DCM at room temperature. Under this condition, the expected product was now obtained in 80 % yield with a diastereoselectivity higher than 95 : 05 (Scheme 2). The opposite diastereomer **2 b** could equally be smoothly prepared by permuting the nature of the two substituents at the quaternary carbon stereocenter (Scheme 2). Various aryl cyclopropyl ketones with electron‐donating or electron‐withdrawing groups could participate in the reaction to yield the corresponding *γ*‐bromo ketones (**2 c**–**2 f**) in good yields with excellent diastereoselectivities. Cyclopropyl ketone **1 g** bearing electron‐rich thiophene also underwent highly efficient and selective nucleophilic substitution to give **2 g**. The reaction proceeds similarly on aliphatic ketones (formation of **2 h** and **2 i**, Scheme 2) and the cleavage of a C−C bond is preferred to the nucleophilic substitution at a primary carbon‐chlorine bond (see **2 j**, Scheme 2). It should be noted that **1 n** (R^1^=H) provide exclusively tertiary bromide **2 k** in high yield instead of the concerted homo‐conjugate nucleophilic addition pathway.^[9]^ This discrepancy could be rationalized by the presence of a bicyclobutonium intermediate **A** (Scheme 1b) controlling the regioselective addition at the carbon center possessing the highest density of positive charge. It is worth noting that cyclopropyl ketones **1 p** and **1 q** containing the molecular backbone of probenecid and indomethacin with basic nitrogen atoms were also smoothly transformed into **2 l** and **2 m** in satisfactory yield with a complete selectivity (Scheme 2). The relative configurations were determined by X‐ray analysis of **2 c** and **2 d** and all other configurations were assigned by analogy.^[12]^ The nucleophilic substitution at quaternary carbon stereocenter proceeds with a complete inversion of configuration. We then turned our attention to the chlorination reactions and our initial attempts to promote the ytterbium‐catalyzed chlorination reaction of cyclopropyl ketone **1 a** with TMSCl failed with **1 a** being fully recovered. Several different catalysts were therefore evaluated (see Supporting Information for all details) and ZnCl_2_ was found to be the most suitable catalyst when MeCN was used as solvent. In addition, owning to the moisture sensitive nature of TMSCl, the alternative Me_2_PhSiCl was used as chlorinating agent. With a suitable condition for the chlorination of cyclopropyl ketone **1 a**, the scope of substrates was further investigated as described in Scheme 2. A variety of cyclopropyl ketones **1** were transformed to tertiary chlorides in moderate to good yields with a complete diastereoselectivity (**3 a**–**3 i**). It should be emphasized that the nature of the R^1^ substituent (R^1^=CO_2_Et) could be varied as **1 m** (R^1^=CH_2_OMe) was converted to **3 h** under similar mild conditions. To our delight, cyclopropyl aldehyde **1 o** could also be smoothly transformed into **3 i** even without the use of ZnCl_2_ as catalyst. In all cases, the nucleophilic substitution proceeds at the most substituted quaternary carbon center with a complete inversion of configuration. The successful realization of bromination and chlorination reactions prompted us to explore the use of other nucleophiles, and as briefly mentioned above, we were particularly interested to add nitrogen nucleophile.

**Scheme 2:**
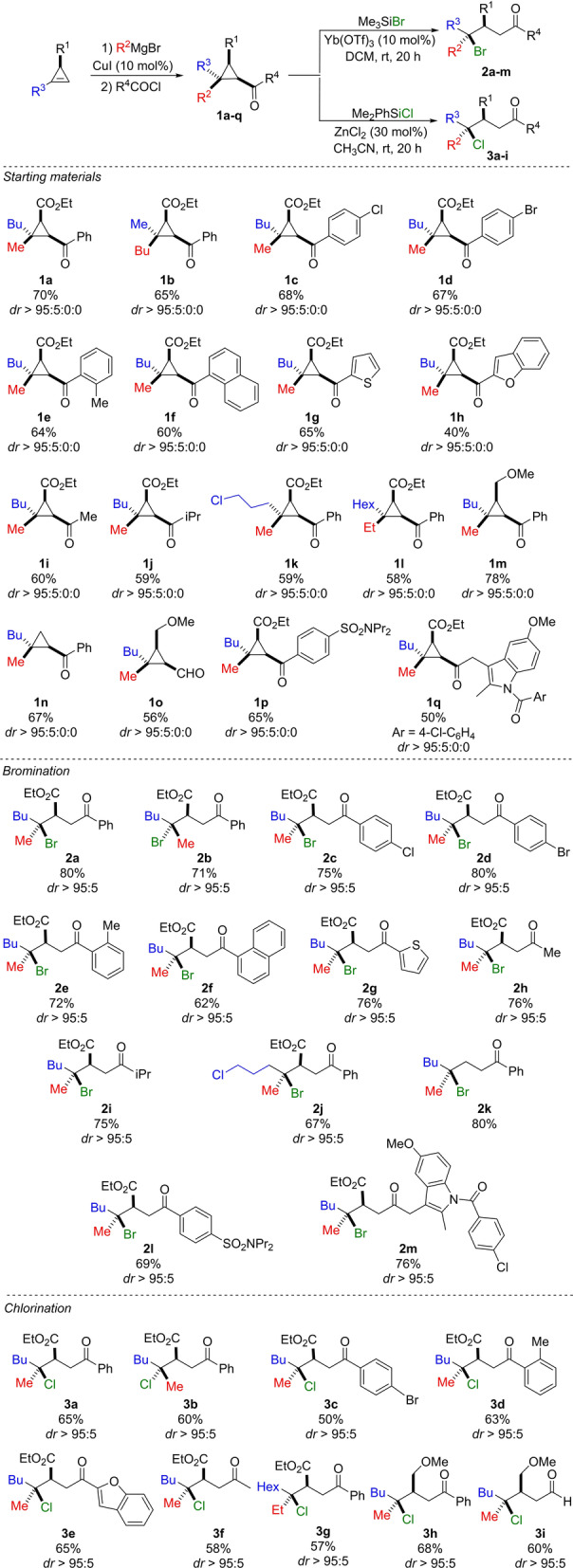
Nucleophilic substitution of cyclopropyl ketones with halides.

Among all possible sources of nitrogen, organic azide represents a versatile synthetic intermediate as it provides a convenient access to a large variety of nitrogen‐containing functional groups such as amines, imines, amides, and triazoles.^[13]^ Although some notable examples have been reported,[^3g^, ^3k^, ^3l^, ^14^] the development of new approaches to the stereodefined synthesis of tertiary azides is still highly desirable. With this idea in mind, the Lewis acid‐catalyzed nucleophilic substitution of cyclopropyl ketones using TMSN_3_ as nucleophile was tested but met only moderate success. We then had to turn our attention to the azidation of cyclopropyl carbinol derivatives, easily achieved as single diastereomer, that should be smoothly activated by the presence of a Lewis acid. We initially prepared stereodefined cyclopropyl methyl ethers **5** via the copper‐catalyzed carbomagnesiation of cyclopropene followed by reaction of the resulting stereodefined cyclopropyl magnesium halide with aldehydes and subsequent etherification. Cyclopropyl methyl ethers **5 a**–**j** were prepared in good yields with excellent diastereoselectivities over the four stereocenters (Scheme 3). Only **4 g**, substituted by a propyl group (R^1^=Pr, R^2^=Me, R^3^=Bu, R^4^=Ph), is produced as two diastereomers at the carbinol center. The relative configuration of cyclopropyl methyl ether was determined on **4 b** by X‐ray analysis (R^1^=CH_2_OH, R^2^=Bu, R^3^=Me, R^4^=C_6_H_5_)^[15]^ and all other configurations were assigned by analogy. With **5 a** as our model substrate, the addition of TMSN_3_, as the source of azide, was investigated and after extensive screening, the inexpensive and environmentally friendly catalysts FeCl_3_ (2.5 mol %) in toluene at −30 °C for 20 h provided the optimal transformation (see Supporting Information for all details). Under this condition, the resulting azide **6 a** was obtained in a good yield and excellent diastereomeric ratio. For subsequent purification, azides **6** could either be fully reduced by H_2_ on Pd/C or by treating the crude reaction mixture under the Staudinger condition,^[16]^ the latter presenting the advantage to preserve the carbon‐carbon unsaturation. In all cases, the primary amines are then protected to give **7** and **8**, respectively.

**Scheme 3:**
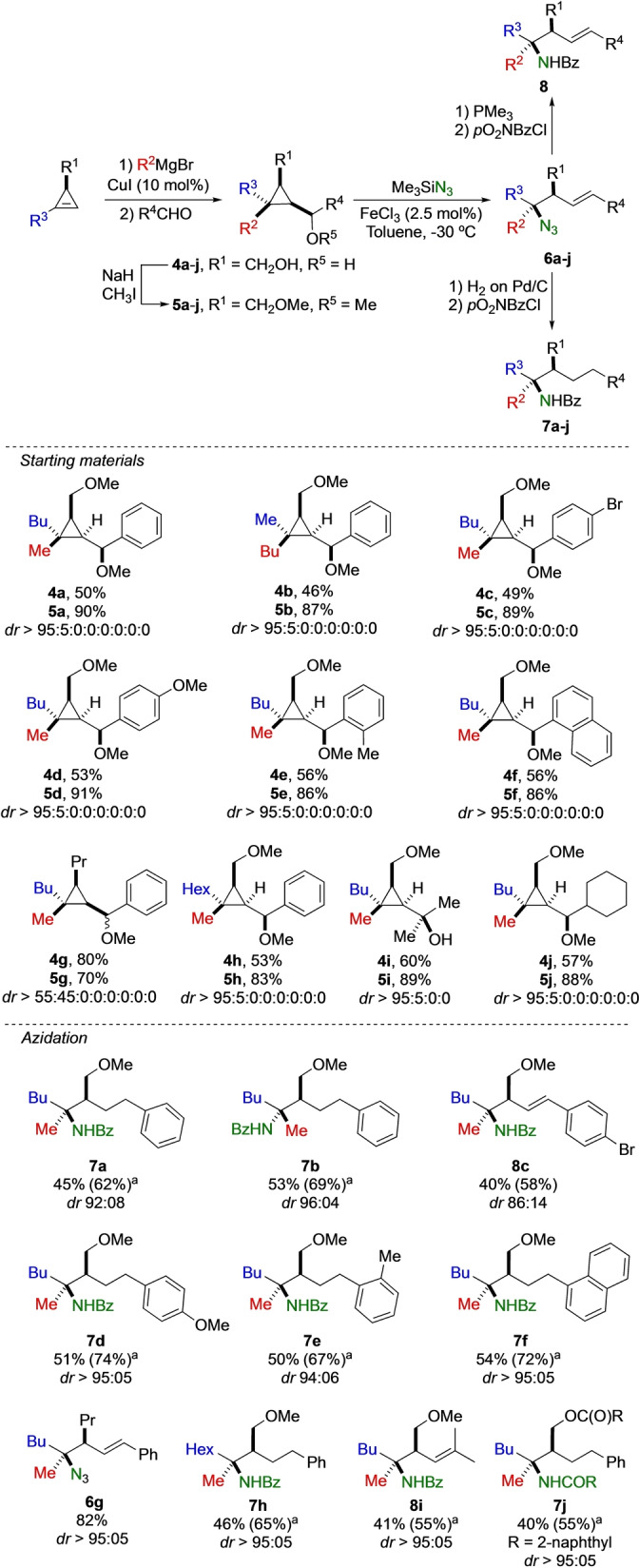
Fe‐catalyzed nucleophilic substitution of cyclopropyl ethers with azide. [a] Yields of isolated products **7**. In parenthesis, yields of the azides **6**.

With suitable conditions in hand, we have then investigated a range of diversely substituted cyclopropyl carbinol derivatives **5 a**–**j** to determine the scope of the azidation reaction (Scheme 3). All cyclopropyl ethers were smoothly converted into the corresponding tertiary azides in moderate to good yields with high to excellent diastereoselectivities at the exception of **5 j**. Generally, cyclopropyl methyl ethers **5 a**–**h** possessing electron‐rich R^4^ substituents lead to a smooth and highly diastereoselective transformation in the azidation reaction. In contrast, when non‐activated aliphatic cyclopropyl methyl ether **5 j** was treated under the same experimental condition, it proved to be unreactive, underlining the necessity to smoothly generate an initial cyclopropyl carbinyl intermediate. To further corroborate this hypothesis, cyclopropyl carbinol derivative **5 i** possessing a tertiary alcohol instead of an ether could be smoothly transformed into **6 i** and then into **8 i** in moderate yield but with high diastereomeric ratio (Scheme 3). It should be noted that the nature of the group R^1^ could again be varied as the reaction proceeds similarly when R^1^=CH_2_OMe (**6 a**–**f**), with a free alcohol (R^1^=CH_2_OH, **4 a** providing **6 j** that was subsequently protected as **7 j**), or when R^1^ is an alkyl group (R^1^=Pr) as in **5 g**. In the latter case, the azide **6 g** could be prepared in good yield with high diastereomeric ratio. It should be noted, here again, that in all cases, the nucleophilic displacement occurred at the quaternary carbon stereocenter with a complete inversion of configuration, which was confirmed by structural analysis of X‐ray crystallographic data of amide **7 a**.^[17]^ It is worth mentioning that this approach represents a nice complement to the previous report on the construction of highly congested tertiary amine via stereoinvertive substitution at tertiary trifluoroacetate under solvolytic conditions, where moderate diastereoselectivities were observed for highly branched acyclic tertiary alcohols.^[6f]^

In conclusion, a highly regio‐ and stereoselective nucleophilic substitution at quaternary carbon stereocenter of cyclopropyl ketones and cyclopropyl methyl ether derivatives was developed, enabling a new access to diastereomerically enriched tertiary γ‐halogenated ketones and highly congested tertiary alkyl amides or azides from cyclopropenes, easily accessible from commercially available alkynes. The presence of a bicyclobutonium intermediate is suggested to account for the high regioselectivity as well as the predictable stereoinvertive substitution at the quaternary carbon stereocenter. Further, this method represents an additional example for the stereospecific nucleophilic displacement at the sterically encumbered carbon stereocenter via cationic intermediates.

## Conflict of interest

The authors declare no conflict of interest.

## Supporting information

As a service to our authors and readers, this journal provides supporting information supplied by the authors. Such materials are peer reviewed and may be re‐organized for online delivery, but are not copy‐edited or typeset. Technical support issues arising from supporting information (other than missing files) should be addressed to the authors.

Supporting Information

## Data Availability

The data that support the findings of this study are available in the Supporting Information of this article.
