# Supplementary material for: Stereoinvertive Nucleophilic Substitution at Quaternary Carbon Stereocenters of Cyclopropyl Ketones and Ethers
Source: Angew Chem Int Ed Engl. 2022 May 11;61(26):e202203673. doi: 10.1002/anie.202203673 (PMC9324837; doi:10.1002/anie.202203673)
Supplement: Supplementary file 1 — Supporting Information [file ANIE-61-0-s001.pdf]

Angewandte Chemie
Eine Zeitschrift der Gesellschaft Deutscher Chemiker

Supporting Information

Stereoinvertive Nucleophilic Substitution at Quaternary Carbon Stereocenters of Cyclopropyl Ketones and Ethers

*X. Chen, I. Marek**

I. General Information 2

II. Preparation of Starting materials 2

III. Optimization Studies 17

IV. Experimental Procedures and Characterization Data 20

V. References 36

VI. NMR Spectra 36

I. General Information

Ether and THF were dried from Pure-Solv® Purification System (Innovative Technology©). Dichloromethane, acetonitrile and toluene were distilled from CaH_2. CuI, $Rh(OAc)_2$, $Yb(OTf)_3$, TMSBr, $FeCl_3$ and MeMgBr (3.0 M in diethyl ether) were purchased from Aldrich. BuMgBr and PhMgBr were prepared in solution in Et_2O and freshly titrated with menthol and 1,10-phenanthroline as indicator prior use. All other commercially obtained reagents were used as received. NMR spectra were recorded on a Bruker©spectrometers AVIII400. ^{1}H NMR chemical shifts were referenced to $CHCl_3$ signal (7.26 ppm),^{13}C NMR chemical shifts were referenced to the solvent resonance (77.00 ppm, $CDCl_3$). The following abbreviations were used to explain multiplicities: s = singlet, d = doublet, t = triplet, q = quadruplet, m = multiplet, br = broad. High-resolution mass spectra (HRMS) were recorded on ESI-TOF (electrospray ionization-time of flight) or APCI. Crystal XRD data were collected on a diffractometer *Nonius Kappa CCD* at Schulich Faculty of Chemistry at Technion-Israel Institute of Technology.

II. Preparation of Starting materials

Cyclopropenyl esters and cyclopropenyl methanol derivatives were prepared according to the previous reported methods.[1] Cyclopropyl ketones and cyclopropyl carbinol derivatives were prepared according to the methods reported by our group[2] by simply changing the nature of the electrophile to acyl chloride or aldehyde.

General procedure A for the preparation of cyclopropyl ketones 1.

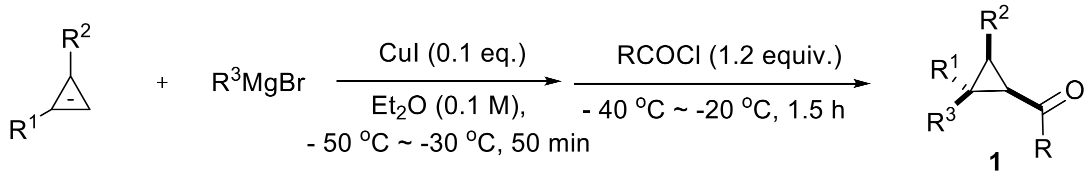

A 50 mL oven-dried three-necked round-bottom flasks was charged with CuI (0.3 mmol, 0.1 equiv.), Et_2O (30 mL) and cyclopropenes (3.0 mmol, 1.0 equiv.). The flask was cooled to -50 ºC, and MeMgBr (1.2 mL, 3.0 M in Et_2O, 1.2 equiv.) was added dropwise. The resulting mixture was then stirred at -50 to -30 ºC for 50 minutes. Then, acyl chloride (3.6 mmol) was added slowly to the reaction mixture at -30 ºC and stirred further for 1.5 h at -30 to -20 ºC. The reaction was quenched with an aqueous NH_4Cl/NH_4OH (2:1) solution. The aqueous layer was extracted with Et_2O (3×30 mL). The combined

organic phases were dried over $NaSO_4$, filtered, and concentrated under reduced pressure. The crude mixture was purified by column chromatography using petroleum ether/ethyl acetate as eluent (abbreviated as PE/EA) in a 20/1 to give cyclopropyl ketone **1**.

General procedure B for the preparation of cyclopropyl carbinols 4.

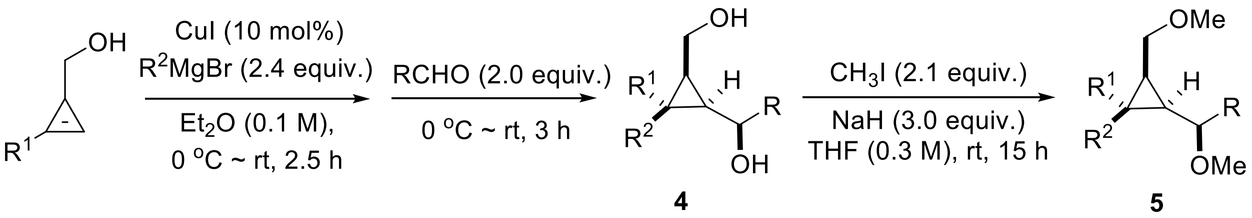

Under argon atmosphere, a 100 mL oven-dried round-bottom flask was charged with CuI (0.5 mmol), cyclopropenes (5.0 mmol), and Et_2O (50 mL). MeMgBr (4.0 mL, 3.0 M in Et_2O, 2.4 equiv.) was added dropwise to the resulting mixture at 0 ºC. When the addition was over, the reaction mixture was warmed to room temperature and stirred for 2.5 h. Then, aldehyde (10 mmol) was added slowly to this reaction mixture at 0 °C and stirred at room temperature for 3 h. The reaction was quenched with an aqueous NH_4Cl/NH_4OH (2:1) solution. The aqueous layer was extracted with EtOAc (3×40 mL). The combined organic phases were dried over $NaSO_4$, filtered, and concentrated under reduced pressure. The resulting crude mixtures were analyzed by ^1H NMR to determine the *dr* using CH_2Br_2 (50 uL) as internal standard. Then, the crude mixture was purified by column chromatography using PE/EA (4/1 to 2/1) as eluent to give the products **4**.

General procedure C for the preparation of 5.

A 50 mL oven-dried round-bottom flask was charged with **4** (2.5 mmol), and THF (10 mL). NaH (7.5 mol, 60% wt. dispersion in mineral oil, 3.0 equiv.) was added portion wise to the resulting mixture at 0 °C and stirred for 1 h at room temperature. Then, CH_3I (5.2 mmol) was added dropwise at 0 °C and stirred for further 16 h upon warming to room temperature. The reaction was quenched with an aqueous NH_4Cl solution. The aqueous layer was extracted with Et_2O (3×20 mL). The combined organic phases were dried over $NaSO_4$, filtered, and concentrated under reduced pressure. The resulting crude mixtures were purified by column chromatography using PE/EA (20/1 to 10/1) as the eluent to give the products **5**.

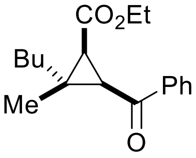

Ethyl (1S*,2S*,3R*)-3-benzoyl-2-butyl-2-methylcyclopropane-1-carboxylate (**1a**). Prepared according to the general procedure A. 70% yield (2.019 g, 7.0 mmol on 10 mmol scale), *dr* >95/5, Colorless oil.^{1}H NMR (400 MHz, $CDCl_3$) δ 8.03 – 7.87 (m, 2H), 7.57 – 7.50 (m, 1H), 7.47 – 7.41 (m, 2H), 4.11 (q, *J* = 7.2 Hz, 2H), 2.57 (d, *J* = 9.2 Hz, 1H), 2.05 (d, *J* = 9.2 Hz, 1H), 1.64 – 1.54 (m, 1H), 1.51 – 1.30 (m, 8H), 1.18 (t, *J* = 7.2 Hz, 3H), 0.92 (t, *J* = 7.2 Hz, 3H). ^{13}C NMR (100 MHz, $CDCl_3$) δ 195.2, 169.7, 138.1, 132.8, 128.5, 128.0, 60.4, 41.9, 36.1, 32.8, 31.8, 28.5, 22.6, 14.07, 14.06, 13.0. HRMS (APCI) calcd. for $C_{18}H_{25}O_3$ [M+H]: 289.1804, found: 289.1820.

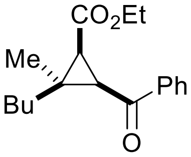

Ethyl (1S*,2R*,3R*)-3-benzoyl-2-butyl-2-methylcyclopropane-1-carboxylate (**1b**). Prepared according to the general procedure A. 65% yield (0.562 g, 1.95 mmol on 3 mmol scale), *dr* >95/5, Colorless oil. ^{1}H NMR (400 MHz, $CDCl_3$) δ 7.95 (d, *J* = 8.0 Hz, 2H), 7.54 (t, *J* = 7.2 Hz, 1H), 7.44 (t, *J* = 7.6 Hz, 2H), 4.10 (q, *J* = 7.2 Hz, 2H), 2.58 (d, *J* =8.8 Hz, 1H), 2.06 (d, *J* = 9.2 Hz, 1H), 1.85 – 1.70 (m, 2H), 1.41 – 1.22 (m, 7H), 1.17 (t, *J* = 7.2 Hz, 3H), 0.82 (t, *J* = 7.2 Hz, 3H). ^{13}C NMR (100 MHz, $CDCl_3$) δ 195.0, 169.5, 138.1, 132.7, 128.4, 127.9, 60.4, 37.1, 33.8, 32.2, 29.3, 28.4, 25.4, 23.0, 14.0. HRMS (APCI) calcd. for $C_{18}H_{25}O_3$ [M+H]: 289.1804, found: 289.1826.

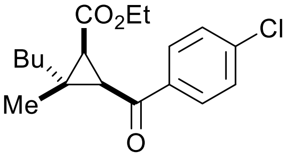

Ethyl (1S*,2S*,3R*)-2-butyl-3-(4-chlorobenzoyl)-2-methylcyclopropane-1-carboxylate (**1c**). Prepared according to the general procedure A. 68% yield (0.352 g, 1.09 mmol on 1.6 mmol scale), *dr* >95/5, Pale yellow oil. ^{1}H NMR (400 MHz, $CDCl_3$) δ 7.92 – 7.70 (m, 2H), 7.41 (d, *J* = 8.4 Hz, 2H), 4.09 (q, *J* = 7.2 Hz, 2H), 2.49 (d, *J* = 9.2 Hz, 1H), 2.06 (d, *J* = 9.2 Hz, 1H), 1.58 – 1.30 (m, 9H), 1.18 (t, *J* = 7.2 Hz, 3H), 0.92 (t, *J* = 7.2 Hz, 3H). ^{13}C NMR (100 MHz, $CDCl_3$) δ 193.9, 169.5, 139.2, 136.4, 129.3, 128.8, 60.5, 41.9, 35.8, 32.9, 31.8, 28.5, 22.6, 14.09, 14.05, 13.0. HRMS (APCI) calcd. for $C_{18}H_{24}ClO_3$ [M+H]: 323.1414, found: 323.1424.

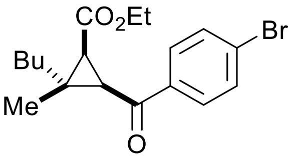

Ethyl (1S*,2S*,3R*)-3-(4-bromobenzoyl)-2-butyl-2-methylcyclopropane-1-carboxylate (**1d**). Prepared according to the general procedure A. 67% yield (0.492 g, 1.34 mmol on 2 mmol scale), *dr* >95/5, Pale yellow oil. ^{1}H

NMR (400 MHz, $CDCl_3$) δ 7.82 (d, *J* = 8.4 Hz, 2H), 7.58 (d, *J* = 8.4 Hz, 2H), 4.09 (q, *J* = 7.2 Hz, 2H), 2.48 (d, *J* = 9.2 Hz, 1H), 2.06 (d, *J* = 9.2 Hz, 1H), 1.57 – 1.27 (m, 9H), 1.19 (t, *J* = 7.2 Hz, 3H), 0.92 (t, *J* = 7.2 Hz, 3H). ^{13}C NMR (100 MHz, $CDCl_3$) δ 194.1, 169.5, 136.8, 131.8, 129.5, 127.9, 60.5, 41.9, 35.8, 32.9, 31.8, 28.5, 22.6, 14.09, 14.05, 13.0. HRMS (APCI) calcd. for $C_{18}H_{24}BrO_3$ [M+H]: 367.0909, found: 367.0921.

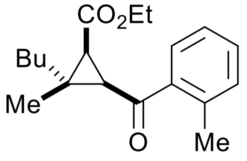

Ethyl (1S*,2S*,3R*)-2-butyl-2-methyl-3-(2-methylbenzoyl)cyclopropane-1-carboxylate (**1e**). Prepared according to the general procedure A. 64% yield (0.387 g, 1.28 mmol on 2 mmol scale), *dr* >95/5, Colorless oil. ^1H NMR (400 MHz, $CDCl_3$) δ 7.65 (d, *J* = 7.2 Hz, 1H), 7.37 – 7.31 (m, 1H), 7.26 – 7.20 (m, 2H), 4.21 – 4.06 (m, 2H), 2.53 – 2.47 (m, 4H), 2.01 (d, *J* = 9.2 Hz, 1H), 1.62 – 1.55 (m, 1H), 1.51 – 1.41 (m, 2H), 1.38 – 1.26 (m, 6H), 1.22 (t, *J* = 7.2 Hz, 3H), 0.91 (t, *J* = 7.2 Hz, 3H). ^{13}C NMR (100 MHz, $CDCl_3$) δ 199.1, 169.5, 139.0, 138.0, 131.7, 131.0, 128.5, 125.5, 60.4, 41.9, 38.6, 33.6, 32.8, 28.5, 22.6, 20.9, 14.1, 14.0, 12.8. HRMS (APCI) calcd. for $C_{19}H_{27}O_3$ [M+H]: 303.1960, found: 303.1980.

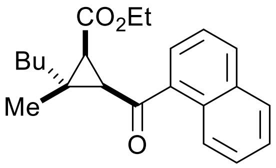

Ethyl(1S*,2S*,3R*)-3-(1-naphthoyl)-2-butyl-2-methylcyclopropane-1-carboxyl-ate (**1f**). Prepared according to the general procedure A. 60% yield (0.406 g, 1.20 mmol on 2 mmol scale), *dr* >95/5, Colorless oil. ^1H NMR (400 MHz, $CDCl_3$) δ 8.61 (d, *J* = 8.4 Hz, 1H), 7.97 (d, *J* = 8.4 Hz, 1H), 7.89 – 7.83 (m, 2H), 7.62 – 7.46 (m, 3H), 4.24 – 4.15 (m, 2H), 2.69 (d, *J* = 8.8 Hz, 1H), 2.09 (d, *J* = 9.2 Hz, 1H), 1.68 – 1.60 (m, 1H), 1.52 – 1.31 (m, 8H), 1.26 (t, *J* = 7.2 Hz, 3H), 0.93 (t, *J* = 7.2 Hz, 3H). ^{13}C NMR (100 MHz, $CDCl_3$) δ199.1, 169.4, 137.2, 133.8, 132.3, 130.1, 128.3, 127.7, 127.5, 126.4, 125.9, 124.3, 60.5, 41.9, 39.1, 34.0, 33.3, 28.5, 22.6, 14.2, 14.1, 12.8. HRMS (APCI) calcd. for $C_{22}H_{27}O_3$ [M+H]: 339.1955, found: 339.1982.

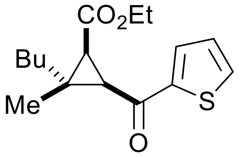

Ethyl (1S*,2S*,3R*)-2-butyl-2-methyl-3-(thiophene-2-carbonyl)cyclopropane-1-carboxylate (**1g**). Prepared according to the general procedure A. 65% yield (0.476 g, 1.62 mmol on 2.5 mmol scale), *dr* >95/5, Pale yellow oil. ^1H NMR (400 MHz, $CDCl_3$) δ 7.71 (dd, *J* = 4.0, 1.2 Hz, 1H), 7.59 (dd, *J* = 4.8, 0.8 Hz, 1H), 7.11 (dd, *J* = 4.8, 3.6 Hz, 1H), 4.13 (q, *J* = 7.2 Hz, 2H), 2.55 (d, *J* = 9.2 Hz, 1H), 2.03 (d, *J* = 9.2 Hz, 1H), 1.53 – 1.40

(m, 4H), 1.39 – 1.29 (m, 5H), 1.21 (t, J = 7.2 Hz, 3H), 0.92 (t, J = 7.2 Hz, 3H). ^{13}C NMR (400 MHz, $CDCl_3$) δ 187.8, 169.3, 145.3, 133.0, 131.4, 127.9, 60.5, 41.9, 36.2, 33.2, 32.2, 28.4, 22.6, 14.08, 14.05, 12.8. HRMS (APCI) calcd. for $C_{16}H_{23}O_3S$ [M+H]: 295.1362, found: 295.1383.

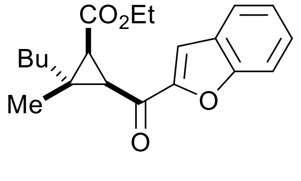

Ethyl (1S*,2S*,3R*)-3-(benzofuran-2-carbonyl)-2-butyl-2-methylcyclopropane-1-carboxylate (**1h**). Prepared according to the general procedure A. 40% yield (0.263 g, 0.80 mmol on 2 mmol scale), *dr* >95/5, Pale yellow oil. ^{1}H NMR (400 MHz, $CDCl_3$) δ 7.70 (d, J = 8.0 Hz, 1H), 7.56 (d, J = 8.4 Hz, 1H), 7.50 – 7.42 (m, 2H), 7.30 (t, J = 7.6 Hz, 1H), 4.22 – 4.03 (m, 2H), 2.72 (d, J = 9.2 Hz, 1H), 2.12 (d, J = 9.2 Hz, 1H), 1.53-1.44 (m, 4H), 1.42 – 1.30 (m, 5H), 1.22 (t, J = 7.2 Hz, 3H), 0.93 (t, J = 7.2 Hz, 3H). ^{13}C NMR (400 MHz, $CDCl_3$) δ186.1, 169.1, 155.4, 153.5, 127.9, 127.1, 123.8, 123.2, 112.4, 111.9, 60.6, 41.9, 35.3, 33.9, 32.9, 28.5, 22.6, 14.1, 14.08, 12.6. HRMS (APCI) calcd. for $C_{20}H_{25}O_4$ [M+H]: 329.1747, found: 329.1776.

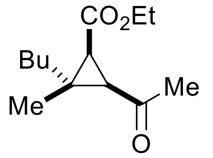

Ethyl (1S*,2S*,3R*)-3-acetyl-2-butyl-2-methylcyclopropane-1-carboxylate (**1i**). Prepared according to the general procedure A. 60% yield (0.407 g, 1.80 mmol on 3 mmol scale), *dr* >95/5, Colorless oil. ^{1}H NMR (400 MHz, $CDCl_3$) δ 4.21 – 4.08 (m, 2H), 2.22 (s, 3H), 2.01 (d, J = 9.2 Hz, 1H), 1.86 (d, J = 9.2 Hz, 1H), 1.43 – 1.22 (m, 12H), 0.90 (t, J = 7.2 Hz, 3H); ^{13}C NMR (100 MHz, $CDCl_3$) δ 203.5, 169.4, 60.5, 41.8, 38.8, 33.1, 31.9, 31.6, 28.5, 22.6, 14.2, 14.1, 12.4. HRMS (APCI) calcd. for $C_{13}H_{23}O_3$ [M+H]: 227.1642, found: 227.1652.

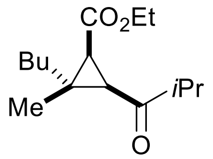

Ethyl (1S*,2S*,3R*)-2-butyl-3-isobutyryl-2-methylcyclopropane-1-carboxylate (**1j**). Prepared according to the general procedure A. 59% yield (0.450 g, 1.77 mmol on 3 mmol scale), *dr* >95/5, Colorless oil. ^{1}H NMR (400 MHz, $CDCl_3$) δ 4.19 – 4.11 (m, 2H), 2.76 – 2.63 (m, 1H), 2.12 (d, J = 9.2 Hz, 1H), 1.86 (d, J = 9.2 Hz, 1H), 1.43 – 1.22 (m, 12H), 1.10 (t, J = 7.2 Hz, 6H), 0.90 (t, J = 7.2 Hz, 3H). ^{13}C NMR (100 MHz, $CDCl_3$) δ 209.3, 169.3, 60.4, 42.0, 41.9, 36.6, 33.6, 32.1, 28.4, 22.6, 18.2, 17.7, 14.1, 14.0, 12.6. HRMS (APCI) calcd. for $C_{15}H_{27}O_3$ [M+H]: 255.1960, found: 255.1947.

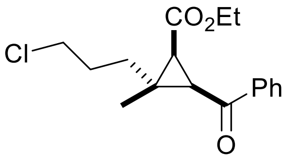

Ethyl (1S*,2S*,3R*)-3-benzoyl-2-(3-chloropropyl)-2-methylcyclopropane-1-carboxylate (**1k**). Prepared according to the general procedure A. 59% yield (0.547 g, 1.77 mmol on 3 mmol scale), *dr* >95/5, Colorless oil. ^{1}H NMR (400 MHz, $CDCl_3$) δ 7.96 (d, *J* = 8.0 Hz, 2H), 7.59 – 7.51 (m, 1H), 7.45 (t, *J* = 7.6 Hz, 2H), 4.09 (q, *J* = 7.2 Hz, 2H), 3.61 (t, *J* = 6.4 Hz, 2H), 2.62 (d, *J* = 9.2 Hz, 1H), 2.12 (d, *J* = 9.2 Hz, 1H), 2.05 – 1.95 (m, 2H), 1.79 – 1.69 (m, 1H), 1.64 – 1.54 (m, 1H), 1.37 (s, 3H), 1.17 (t, *J* = 7.2 Hz, 3H). ^{13}C NMR (100 MHz, $CDCl_3$) δ 194.8, 169.3, 137.9, 132.9, 128.5, 127.9, 60.6, 44.5, 38.9, 35.7, 32.7, 30.5, 29.3, 14.1, 12.8. HRMS (APCI) calcd. for $C_{17}H_{22}ClO_3$ [M+H]: 309.1252, found: 309.1277.

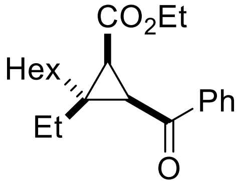

Ethyl (1*S**,2*S**,3*R**)-3-benzoyl-2-ethyl-2-hexylcyclopropane-1-carboxylate (**1l**). Prepared according to the general procedure A. 58% yield (0.383 g, 1.16 mmol on 2 mmol scale), *dr* >95/5, Colorless oil. ^{1}H NMR (400 MHz, $CDCl_3$) δ 8.00-7.94 (m, 2H), 7.56 – 7.49 (m, 1H), 7.47 – 7.41 (m, 1H), 4.10 (q, *J* = 7.2 Hz, 2H), 2.60 (d, *J* = 9.2 Hz, 1H), 2.07 (d, *J* = 9.2 Hz, 1H), 1.88 (q, *J* = 7.2 Hz, 2H), 1.50 – 1.40 (m, 3H), 1.36 – 1.25 (m, 7H), 1.18 (t, *J* = 7.2 Hz, 3H), 0.93-0.81 (m, 6H). ^{13}C NMR (100 MHz, $CDCl_3$) δ 195.1, 169.4, 138.2, 132.6, 128.4, 127.9, 60.4, 37.7, 37.1, 36.1, 33.7, 31.8, 29.3, 25.9, 22.6, 18.1, 14.1, 14.0, 10.9. HRMS (APCI) calcd. for $C_{21}H_{31}O_3$ [M+H]: 331.2268, found: 331.2256.

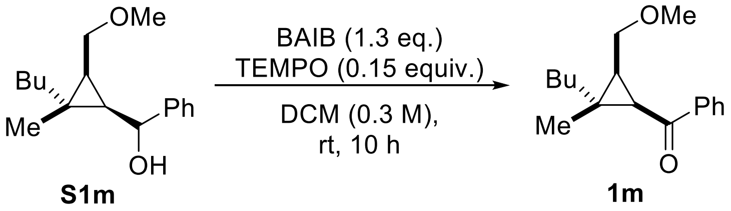

To a solution of bis(acetoxy)iodobenzene (BAIB) (2.6 mmol) and cyclopropyl alcohol **S1m**[1b] (2 mmol) in DCM (6 mL) in a round-bottom flask, (2,2,6,6-tetramethyl-1-piperidin-1-yl)oxy (TEMPO) (0.3 mmol) was added at r.t. and the resulting reaction mixture was stirred for 10 h. Then, the reaction mixture was diluted with DCM (30 mL) and washed with a saturated aqueous solution of $Na_2S_2O_3$ (20 mL). The aqueous layer was extracted with DCM (2 x 20 mL) and the combined organic layers were dried over $NaSO_4$, filtered, and evaporated to give the crude product which was further purified by column chromatography using PE/EA (20/1) as the eluent to give the product **1m** (0.406 g, 1.56 mmol, 78% yield) as a colorless oil. ^{1}H NMR (400 MHz, $CDCl_3$) δ 7.90 (d, *J* = 7.6 Hz, 2H), 7.52 (t,

J = 7.2 Hz, 1H), 7.44 (t, J = 7.2 Hz, 2H), 3.92 – 3.82 (m, 2H), 3.35 (s, 3H), 2.53 (d, J = 8.4 Hz, 1H), 1.66 – 1.53 (m, 2H), 1.50 – 1.29 (m, 5H), 1.20 (s, 3H), 0.91 (t, J = 7.2 Hz, 3H). ^{13}C NMR (100 MHz, $CDCl_3$) δ 198.8, 139.6, 132.3, 128.4, 127.7, 67.9, 58.5, 42.7, 34.4, 33.3, 32.8, 28.7, 22.8, 14.1, 11.5. HRMS (APCI) calcd. for $C_{17}H_{25}O_2$ [M+H]: 261.1849, found: 261.1866.

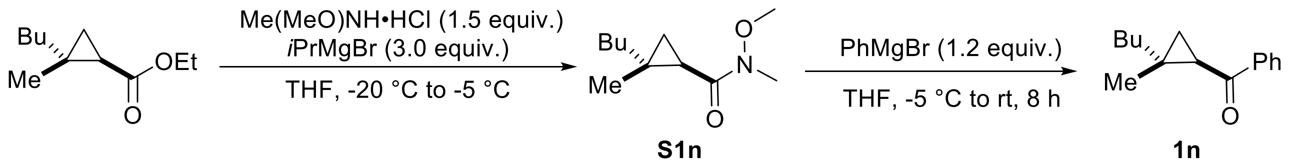

S1n **1n**

Under argon atmosphere, a 50 mL oven-dried three-necked round-bottom flask was charged with cyclopropyl ester[2a] (3.0 mmol), Me(MeO)NH•HCl (4.5 mmol), and THF (10 mL). *i*-PrMgCl (4.5 mmol) was added dropwise to the resulting mixture at -20 °C. When the addition was over, the reaction mixture stirred for 0.5 h at -10 °C. The reaction was then quenched with an aqueous solution of NH_4Cl. The aqueous layer was extracted with Et_2O (3×30 ml). The combined organic phases were dried over $NaSO_4$, filtered, and concentrated under reduced pressure to give the crude product **S1n**.

To a solution of crude **S1n** in THF (5 mL) in a three-necked round-bottom flask, PhMgBr (3.0 mmol, 1.5 M in THF) was added dropwise at -5 °C and the resulting reaction mixture was slowly warmed to room temperature and stirred for 8 h. The reaction was then quenched with diluted HCl (10 mL, 1M) and extracted with Et_2O (3×30 mL). The combined organic phases were dried over $NaSO_4$, filtered, and concentrated under reduced pressure. The crude mixture was purified by column chromatography using PE/EA (20/1) as the eluent to give the product **1n** (0.432g, 2.0 mmol, 67% yield). ^{1}H NMR (400 MHz, $CDCl_3$) δ 7.99 – 7.94 (m,2H), 7.57 – 7.51 (m, 1H), 7.50 – 7.44 (m, 2H), 2.50 (dd, J = 7.5, 5.6 Hz, 1H), 1.63 – 1.53 (m, 1H), 1.53 – 1.41 (m, 4H), 1.41 – 1.30 (m, 2H), 1.07 (s, 3H), 0.99 – 0.92 (m, 4H). ^{13}C NMR (100 MHz, $CDCl_3$) δ 198.5, 139.1, 132.4, 128.4, 128.0, 40.9, 32.1, 31.3, 28.9, 22.9, 21.5, 15.7, 14.1. HRMS (APCI) calcd. for $C_{15}H_{21}O$ [M+H]: 217.1587, found: 217.1617.

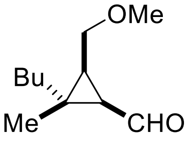

(1*R**,2*R**,3*S**)-2-butyl-3-(methoxymethyl)-2-methylcyclopropane-1-carbaldehyde (**1o**). Prepared according to the previous methods reported by our group[1b]. A 100 mL three-necked round bottom flask containing LiCl (3.0 mmol, 1 equiv.) was flame-dried under vacuum. Under argon, copper bromide dimethyl sulfide complex (0.3 mmol, 10

mol%) and toluene (15 mL) were added to the flask. The Grignard reagent (3.6 mmol, 3.0 M in Et_2O, 1.2 equiv) was added dropwise to the mixture at –50 °C. A bright yellow color appeared in the following 5 minutes. After fifteen minutes, the cyclopropene (3.6 mmol, 1.2 equiv.) was added to the mixture dropwise while keeping the temperature between –40 °C to –50 °C. After about 30 minutes, the temperature was allowed to rise to –30 °C. DMF (3.0 mmol, 1.0 equiv.) was then added dropwise. The reaction mixture was allowed to warm slowly in the cooling bath that reached up to –20 °C in 2 h. The reaction was quenched by 20 mL of NH_4Cl/NH_4OH aqueous solution. The aqueous layer was extracted by diethyl ether (3 x 20 mL) and dried over Na_2SO_4. The combined organic phases were evaporated to give the crude product which was further purified by column chromatography using PE/EA (20/1) as the eluent to give the product **1o** (0.309 g, 1.68 mmol, 56% yield, *dr* >95/5) as a colorless oil. ^{1}H NMR (400 MHz, $CDCl_3$) δ 9.54 (d, *J* = 5.2 Hz, 1H), 3.77 (dd, *J* = 10.4, 8.0 Hz, 1H), 3.65 (dd, *J* = 10.4, 7.2 Hz, 1H), 3.31 (s, 3H), 1.73 (dd, *J* = 8.8, 5.2 Hz, 1H), 1.68 – 1.60 (m, 1H), 1.38 – 1.20 (m, 9H), 0.86 (t, *J* = 7.2 Hz, 3H). ^{13}C NMR (100 MHz, $CDCl_3$) δ 200.7, 67.4, 58.4, 42.2, 37.7, 35.3, 33.6, 28.4, 22.5, 14.0, 12.3. HRMS (APCI) calcd. for $C_{11}H_{21}O_2$ [M+H]: 185.1536, found: 185.1560.

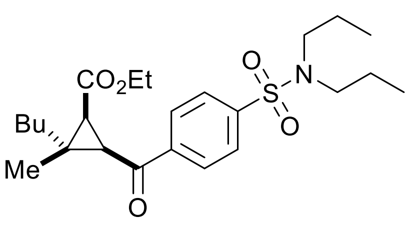

Ethyl (*1S*,2S*,3R**)-2-butyl-3-(4-(*N,N*-dipropylsulfamoyl) benzoyl)-2-methylcyclopropane-1-carboxylate (**1p**). Prepared according to the general procedure A using acyl chloride prepared from *Probenecid*. 65% yield (0.880 g, 1.95 mmol on 3 mmol scale), *dr* >95/5, Pale yellow oil. ^{1}H NMR (400 MHz, $CDCl_3$) δ 8.05 (d, *J* = 8.0 Hz, 2H), 7.87 (d, *J* = 7.6 Hz, 2H), 4.09 (q, *J* = 7.2 Hz, 2H), 3.09 (q, *J* = 7.6 Hz, 4H), 2.52 (d, *J* = 9.2 Hz, 1H), 2.12 (d, *J* = 9.2 Hz, 1H), 1.61 – 1.41 (m, 8H), 1.39 – 1.30 (m, 5H), 1.18 (t, *J* = 7.2 Hz, 3H), 0.93 (t, *J* = 7.2 Hz, 3H), 0.87 (t, *J* = 7.2 Hz, 6H). ^{13}C NMR (100 MHz, $CDCl_3$) δ 194.2, 169.3, 143.8, 140.7, 128.4, 127.2, 60.6, 49.9, 41.8, 35.9, 33.3, 32.2, 28.5, 22.6, 21.9, 14.1, 14.0, 12.9, 11.1. HRMS (APCI) calcd. for $C_{24}H_{38}NO_5S$ [M+H]: 452.2465, found: 452.2446.

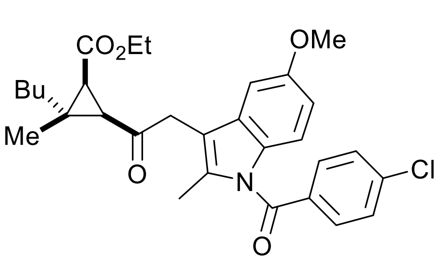

Ethyl (*1S*,2S*,3R**)-2-butyl-3-(2-(1-(4-chlorobenzoyl)-5-methoxy -2-methyl-1H-indol-3-yl)acetyl)-2-methylcyclo-propane-1-carboxylate (**1q**). Prepared according to the general procedure A using acyl chloride prepared from *Indomethacin*. 50%

yield (0.786 g, 1.50 mmol on 3 mmol scale), *dr* >95/5, Pale yellow oil. ^{1}H NMR (400 MHz, $CDCl_3$) δ 7.65 (d, *J* = 8.0 Hz, 2H), 7.47 (d, *J* = 8.0 Hz, 2H), 6.89 (d, *J* = 2.0 Hz, 1H), 6.83 (d, *J* = 8.8 Hz, 1H), 6.66 (dd, *J* = 8.8, 2.0 Hz, 1H), 4.21 – 4.10 (m, 2H), 3.90 – 3.78 (m, 4H), 3.72 (d, *J* = 16.4 Hz, 1H), 2.37 (s, 3H), 2.05 (d, *J* = 9.2 Hz, 1H), 1.86 (d, *J* = 9.2 Hz, 1H), 1.30 – 1.01 (m, 12H), 0.80 (t, *J* = 6.8 Hz, 3H). ^{13}C NMR (100 MHz, $CDCl_3$) δ 202.4, 169.2, 168.2, 156.1, 139.3, 135.9, 133.8, 131.1, 130.85, 130.82, 129.1, 115.0, 112.8, 111.8, 101.2, 60.6, 55.6, 41.6, 40.4, 37.0, 33.9, 32.6, 28.0, 22.5, 14.2, 14.0, 13.4, 12.5. HRMS (APCI) calcd. for $C_{30}H_{35}ClNO_5$ [M+H]: 524.2198, found: 524.2227.

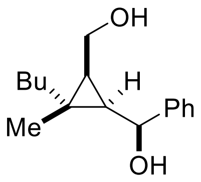

(*S**)-((*1R*,2R*,3S**)-2-butyl-3-(hydroxymethyl)-2-methylcyclopropyl)(phenyl)-methanol (**4a**). Prepared according to the general procedure B. 50% yield (1.242 g, 5.0 mmol on 10 mmol scale), *dr* >95/5, pale yellow oil. ^{1}H NMR (400 MHz, $CDCl_3$) δ7.45 – 7.25 (m, 5H), 4.73 – 4.60 (m, 1H), 4.18 – 4.06 (m, 1H), 3.65 – 3.59 (m, 1H), 3.44 (s, 3H), 1.50 – 1.00 (m, 11H), 0.88 (t, *J* = 7.2 Hz, 3H). ^{13}C NMR (100 MHz, $CDCl_3$) δ 144.0, 128.4, 127.5, 126.0, 70.9, 60.0, 36.0, 30.3, 29.4, 29.3, 26.1, 24.9, 23.1, 14.1. HRMS (ESI) calcd. for $C_{16}H_{24}O_2Na$ [M+Na]: 271.1674, found: 271.1674. (cx12074)

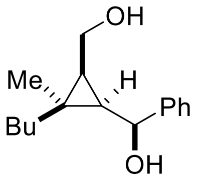

(*S**)-((*1R*,2S*,3S**)-2-butyl-3-(hydroxymethyl)-2-methylcyclopropyl)(phenyl) methanol (**4b**). Prepared according to the general procedure B. 46% isolated yield (0.457 g, 1.84 mmol on 4 mmol scale), *dr* > 95/5, colorless oil. ^{1}H NMR (400 MHz, $CDCl_3$) δ7.45 – 7.25 (m, 5H), 4.73 – 4.60 (m, 1H), 4.18 – 4.06 (m, 1H), 3.65 – 3.59 (m, 1H), 3.44 (s, 3H), 1.50 – 1.00 (m, 11H), 0.88 (t, *J* = 7.2 Hz, 3H). ^{13}C NMR (100 MHz, $CDCl_3$) δ 144.0, 128.4, 127.5, 126.0, 70.9, 60.0, 36.0, 30.3, 29.4, 29.3, 26.1, 24.9, 23.1, 14.1. HRMS (ESI) calcd. for $C_{16}H_{24}O_2Na$ [M+Na]: 271.1674, found: 271.1674. (cx12100)

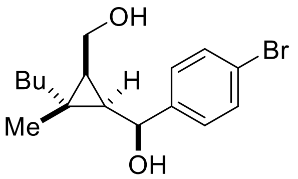

(*S**)-(4-bromophenyl)((*1R*,2R*,3S**)-2-butyl-3-(hydroxymethyl)-2-methyl-cyclopropyl)methanol (**4c**). Prepared according to the general procedure B. 49% yield (1.282 g, 3.92 mmol on 8 mmol scale), *dr* >95/5, pale yellow oil. ^{1}H NMR (400 MHz, $CDCl_3$) δ 7.48 – 7.42 (m, 2H), 7.26-7.21 (m, 2H), 4.52 (d, *J* = 10.4 Hz, 1H), 4.16 (brs, 1H), 4.03 (dd, *J* = 11.6, 5.2 Hz, 1H), 3.57 (t, *J* = 11.6 Hz, 1H), 3.46 (brs, 1H), 1.25-0.98 (m, 11H), 0.80 (t, *J* = 7.2 Hz, 3H); ^{13}C NMR (100 MHz,

$CDCl_3$) δ 143.0, 131.4, 127.5, 121.1, 70.5, 59.9, 42.4, 35.0, 28.4, 28.2, 24.5, 22.5, 14.0, 13.6. HRMS (ESI) calcd. for $C_{16}H_{23}O_2NaBr$ [M+Na]: 349.0779, found: 349.0777. (cx12077)

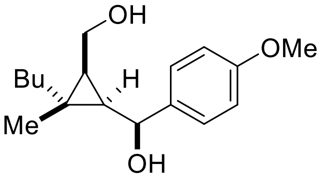

(*S**)-((*1R**,*2R**,*3S**)-2-butyl-3-(hydroxymethyl)-2-methylcyclopropyl)(4-methoxyphenyl)methanol (**4d**). Prepared according to the general procedure B. 53% yield (1.031 g, 3.71 mmol on 7 mmol scale), *dr* >95/5, colorless oil. ^{1}H NMR (400 MHz, $CDCl_3$) δ 7.33 – 7.27 (m, 2H), 6.90 – 6.83 (m, 2H), 4.58 – 4.49 (m, 1H), 4.04-3.96 (m, 1H), 3.90-3.52 (m, 6H), 1.22 – 1.02 (m, 11H), 0.79 (t, *J* = 7.2 Hz, 3H). ^{13}C NMR (100 MHz, $CDCl_3$) δ 158.8, 136.2, 127.0, 113.7, 70.7, 59.8, 55.2, 42.4, 34.7, 28.4, 28.2, 24.3, 22.5, 14.0, 13.4. HRMS (ESI) calcd. for $C_{17}H_{26}O_3Na$ [M+Na]: 301.1780, found: 301.1779. (cx12089/cx13001)

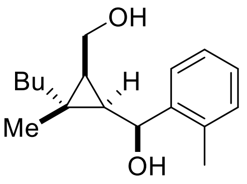

(*S**)-((*1R**,*2R**,*3S**)-2-butyl-3-(hydroxymethyl)-2-methylcyclopropyl)(o-tolyl)methanol (**4e**). Prepared according to the general procedure B. 56% isolated yield (1.028 g, 3.92 mmol on 7 mmol scale), crude nmr shows *dr* = 2.5/1, isolate the major isomer, colorless oil. ^{1}H NMR (400 MHz, $CDCl_3$) δ 7.51 – 7.45 (m, 1H), 7.24 – 7.12 (m, 3H), 4.75 (d, *J* = 10.4 Hz, 1H), 3.99 (dd, *J* = 11.6, 5.6 Hz, 1H), 3.64 (t, *J* = 11.6 Hz, 1H), 3.42 (s, 2H), 2.42 (s, 3H), 1.32 (dd, *J* = 10.4, 8.8 Hz, 1H), 1.28 – 1.05 (m, 7H), 0.96 (s, 3H), 0.81 (t, *J* = 7.2 Hz, 3H). ^{13}C NMR (100 MHz, $CDCl_3$) δ 141.1, 135.7, 130.7, 127.5, 126.5, 126.0, 68.5, 59.8, 42.4, 33.2, 28.7, 28.2, 24.4, 22.5, 19.4, 14.0, 13.4. HRMS (ESI) calcd. for $C_{17}H_{26}O_2Na$ [M+Na]: 285.1830found: 285.1830. (cx12090)

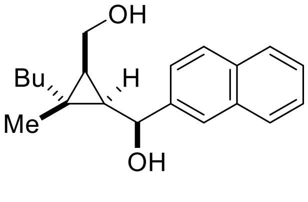

(*S**)-((*1R**,*2R**,*3S**)-2-butyl-3-(hydroxymethyl)-2-methylcyclopropyl)(naph-thalen-2-yl)methanol (**4f**). Prepared according to the general procedure B. 56% yield (0.834 g, 2.80 mmol on 5 mmol scale), *dr* >95/5, colorless oil. ^{1}H NMR (400 MHz, $CDCl_3$) δ 7.85 – 7.76 (m, 4H), 7.54 – 7.42 (m, 3H), 4.69 (d, *J* = 10.0 Hz, 1H), 4.30 (s, 1H), 4.09 – 3.99 (m, 1H), 3.75 (s, 1H), 3.62 (t, *J* = 11.2 Hz, 1H), 1.18 – 1.02 (m, 11H), 0.76 (t, *J* = 6.8 Hz, 3H). ^{13}C NMR (100 MHz, $CDCl_3$) δ 141.4, 133.3, 132.8, 128.1, 128.0, 127.6, 125.9, 125.7, 124.3, 124.2, 71.2, 59.9, 42.4, 34.8, 28.6, 28.2, 24.5, 22.5, 13.9, 13.5. HRMS (ESI) calcd. for $C_{20}H_{26}O_2Na$ [M+Na]: 321.1830, found: 321.1836.(cx13003)

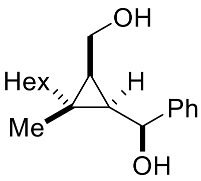

(S^*)-(($1R^*,2R^*,3S^*$)-2-hexyl-3-(hydroxymethyl)-2-methylcyclopropyl)(phenyl) methanol (**4h**). Prepared according to the general procedure B. 53% yield (2.109 g, 7.63 mmol on 14.4 mmol scale), *dr* >95/5, colorless oil. ^{1}H NMR (400 MHz, $CDCl_3$) δ 7.41 – 7.29 (m, 4H), 7.28-7.20 (m, 1H), 4.60 – 4.50 (m, 1H), 4.20 (s, 1H), 4.02 – 3.94 (m, 1H), 3.81 (s, 1H), 3.64 – 3.51 (m, 1H), 1.30 – 1.01 (m, 15H), 0.82 (t, J = 7.2 Hz, 3H).^{13}C NMR (100 MHz, $CDCl_3$) δ 144.0, 128.3, 127.2, 125.7, 71.0, 59.8, 42.7, 34.9, 31.7, 29.1, 28.4, 25.9, 24.4, 22.5, 14.0, 13.5. HRMS (ESI) calcd. for $C_{18}H_{28}O_2Na$ [M+Na]: 299.1987, found: 299.1987.

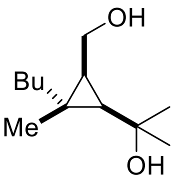

2-(($1R^*,2R^*,3S^*$)-2-butyl-3-(hydroxymethyl)-2-methylcyclopropyl)propan-2-ol (**4i**). Prepared according to the general procedure B. 58% yield (0.580 g, 2.9 mmol on 5 mmol scale), *dr* >95/5, colorless oil. ^{1}H NMR (400 MHz, $CDCl_3$) δ 4.08 (dd, J = 11.2, 8.0 Hz, 1H), 3.74 (dd, J = 11.2, 10.0 Hz, 1H), 2.62 (s, 1H), 2.49 (s, 1H), 1.39 (s, 3H), 1.35 – 1.17 (m, 11H), 1.11 – 0.93 (m, 2H), 0.86 (t, J = 7.2 Hz, 3H), 0.60 (d, J = 9.6 Hz, 1H). ^{13}C NMR (100 MHz, $CDCl_3$) δ70.7, 59.9, 44.1, 37.4, 34.1, 29.9, 28.8, 28.7, 23.9, 22.9, 14.1, 12.5. HRMS (APCI) calcd. for $C_{12}H_{23}O_2$ [M-H]: 199.1693, found: 199.1700.(cx12065)

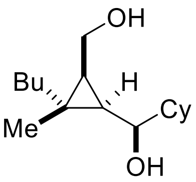

(R^*)-(($1R^*,2R^*,3S^*$)-2-butyl-3-(hydroxymethyl)-2-methylcyclopropyl)(cyclohexyl)methanol (**4j**). Prepared according to the general procedure B. 57% isolated yield (1.031 g, 4 mmol on 7 mmol scale), crude nmr shows *dr* = 5.7/1, isolate the major isomer, colorless oil. ^{1}H NMR (400 MHz, $CDCl_3$) δ 3.96 (dd, J = 11.6, 5.2 Hz, 1H), 3.57 (s, 2H), 3.44 (t, J = 11.6 Hz, 1H), 3.26 (dd, J = 10.4, 6.8 Hz, 1H), 1.95 (d, J = 12.8 Hz, 1H), 1.81 – 1.63 (m, 4H), 1.48 – 0.92 (m, 16H), 0.87 (t, J = 6.8 Hz, 3H), 0.80 (dd, J = 10.4, 8.8 Hz, 1H). ^{13}C NMR (100 MHz, $CDCl_3$) δ 73.5, 60.1, 44.9, 42.7, 32.1, 28.9, 28.8, 28.3, 27.6, 26.5, 26.4, 26.1, 24.4, 22.8, 14.1, 14.0. HRMS (ESI) calcd. for $C_{16}H_{30}O_2Na$ [M+Na]: 277.2143, found: 271.2141. (cx12092)

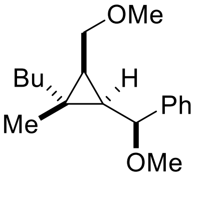

((S^*)-(($1R^*,2R^*,3S^*$)-2-butyl-3-(methoxymethyl)-2-methylcyclopropyl)(methoxy) methyl)benzene (**5a**). Prepared according to the general procedure C. 90% yield (0.647 g, 2.34 mmol on 2.6 mmol scale), *dr* >95/5, colorless oil. ^{1}H NMR (400 MHz, $CDCl_3$) δ 7.39 – 7.25 (m, 5H), 3.91 – 3.75 (m, 2H), 3.51 – 3.35 (m, 4H), 3.15 (s, 3H), 1.26-1.20 (m, 1H), 1.13 – 0.88 (m, 10H), 0.73 (t, J = 7.2 Hz, 3H).^{13}C NMR (100 MHz, $CDCl_3$)

δ 142.2, 128.4, 127.5, 126.7, 81.1, 69.8, 58.5, 56.0, 42.5, 33.4, 28.2, 26.3, 23.1, 22.5, 14.0, 13.2. HRMS (ESI) calcd. for $C_{18}H_{28}O_2Na$ [M+Na]: 299.1987, found: 299.2001. (cx10074)

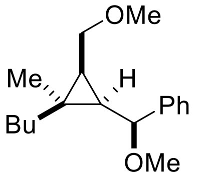

((*S**)-((1*R**,2*S**,3*S**)-2-butyl-3-(methoxymethyl)-2-methylcyclopropyl)(methoxy) methyl)benzene (**5b**). Prepared according to the general procedure C. 87% yield (0.240 g, 0.87 mmol on 1.0 mmol scale), *dr* >95/5, colorless oil. ^{1}H NMR (400 MHz, $CDCl_3$) δ 7.40 – 7.26 (m, 5H), 3.90 (d, *J* = 10.0 Hz, 1H), 3.76 (dd, *J* = 10.4, 5.2 Hz, 1H), 3.47 (t, *J* = 9.6 Hz, 1H), 3.40 (s, 3H), 3.12 (s, 3H), 1.46-1.36 (m, 1H), 1.34 – 1.07 (m, 7H), 0.95 (s, 3H), 0.87 (t, *J* = 6.8 Hz, 3H). ^{13}C NMR (100 MHz, $CDCl_3$) δ 142.3, 128.3, 127.6, 127.0, 80.6, 69.7, 58.5, 55.6, 34.2, 29.7, 28.9, 27.2, 26.1, 23.5, 23.2, 14.1. HRMS (ESI) calcd. for $C_{18}H_{28}O_2Na$ [M+Na]: 299.1987, found: 299.1985.(cx12033)

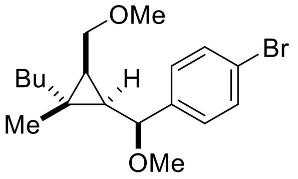

1-bromo-4-((*S**)-((*1R*,2R*,3S**)-2-butyl-3-(methoxymethyl)-2-methylcyclo-propyl)(methoxy)methyl)benzene (**5c**). Prepared according to the general procedure C. 89% yield (1.108 g, 3.12 mmol on 3.5 mmol scale), *dr* >95/5, pale yellow oil. ^{1}H NMR (400 MHz, $CDCl_3$) δ 7.50 – 7.45 (m, 2H), 7.21 – 7.16 (m, 2H), 3.83 (d, *J* = 9.6 Hz, 1H), 3.76 (dd, *J* = 10.8, 5.2 Hz, 1H), 3.42 (dd, *J* = 10.8, 8.8 Hz, 1H), 3.38 (s, 3H), 3.13 (s, 3H), 1.26 – 0.92 (m, 11H), 0.74 (t, *J* = 7.2 Hz, 3H). ^{13}C NMR (100 MHz, $CDCl_3$) δ 141.4, 131.5, 128.4, 121.3, 80.4, 69.6, 58.5, 56.0, 42.4, 33.2, 28.2, 26.2, 23.1, 22.5, 13.9, 13.2. HRMS (ESI) calcd. for $C_{18}H_{27}O_2NaBr$ [M+Na]: 377.1092, found: 377.1089. (cx12082)

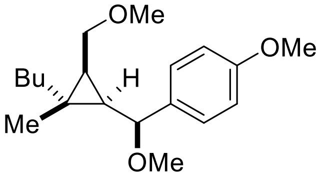

1-((*S**)-((1R*,2R*,3S*)-2-butyl-3-(methoxymethyl)-2-methylcyclo-propyl) (methoxy)methyl)-4-methoxybenzene (**5d**). Prepared according to the general procedure C. 91% yield (1.114 g, 3.64 mmol on 4 mmol scale), *dr* >95/5, colorless oil. ^{1}H NMR (400 MHz, $CDCl_3$) δ 7.89 – 7.81 (m, 3H), 7.70 (s, 1H), 7.54 – 7.44 (m, 3H), 4.05 (d, *J* = 10.0 Hz, 1H), 3.85 (dd, *J* = 10.8, 5.6 Hz, 1H), 3.50 (dd, *J* = 10.8, 9.2 Hz, 1H), 3.43 (s, 3H), 3.18 (s, 3H), 1.26 – 0.93 (m, 11H), 0.67 (t, *J* = 7.2 Hz, 3H).^{13}C NMR (100 MHz, $CDCl_3$) δ 139.6, 133.2, 133.1, 128.4, 127.8, 127.7, 126.0, 125.8, 124.6, 81.2, 69.8, 58.5, 56.0, 42.4, 33.1, 28.2, 26.3, 23.2, 22.5, 13.9, 13.2. HRMS (ESI) calcd. for $C_{19}H_{30}O_3Na$ [M+Na]: 329.2093, found: 329.2091. (cx12096)

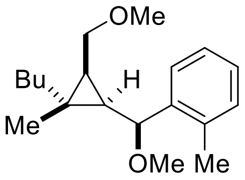

1-((S^*)-((1R*,2R*,3S*)-2-butyl-3-(methoxymethyl)-2-methylcyclopropyl)(metho-xy)methyl)-2-methylbenzene (**5e**). Prepared according to the general procedure C. 82% yield (0.953 g, 3.28 mmol on 4 mmol scale), *dr* >95/5, colorless oil. ^{1}H NMR (400 MHz, $CDCl_3$) δ 7.37 – 7.32 (m, 1H), 7.22 – 7.11 (m, 3H), 4.13 (d, J = 10.0 Hz, 1H), 3.81 (dd, J = 10.4, 5.6 Hz, 1H), 3.46 (dd, J = 10.8, 9.2 Hz, 1H), 3.40 (s, 3H), 3.12 (s, 3H), 2.39 (s, 3H), 1.30 – 1.00 (m, 8H), 0.98 (s, 3H), 0.75 (t, J = 7.2 Hz, 3H). ^{13}C NMR (100 MHz, $CDCl_3$) δ 139.7, 135.8, 130.6, 127.7, 127.3, 126.0, 78.1, 69.9, 58.5, 55.4, 42.5, 31.9, 28.2, 26.3, 23.2, 22.5, 19.4, 14.0, 13.3. HRMS (ESI) calcd. for $C_{19}H_{30}O_2Na$ [M+Na]: 313.2143, found: 313.2144. (cx12097)

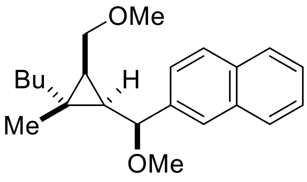

2-((S^*)-((1R*,2R*,3S*)-2-butyl-3-(methoxymethyl)-2-methylcyclopropyl)(methoxy)methyl)naphthalene (**5f**). Prepared according to the general procedure C. 86% yield (0.620 g, 1.89 mmol on 2.2 mmol scale), *dr* > 95/5, colorless oil. ^{1}H NMR (400 MHz, $CDCl_3$) δ 7.89 – 7.81 (m, 3H), 7.70 (s, 1H), 7.54 – 7.44 (m, 3H), 4.05 (d, J = 10.0 Hz, 1H), 3.85 (dd, J = 10.8, 5.6 Hz, 1H), 3.50 (dd, J = 10.8, 9.2 Hz, 1H), 3.43 (s, 3H), 3.18 (s, 3H), 1.26 – 0.93 (m, 11H), 0.67 (t, J = 7.2 Hz, 3H). ^{13}C NMR (100 MHz, $CDCl_3$) δ 139.6, 133.2, 133.1, 128.4, 127.8, 127.7, 126.0, 125.8, 124.6, 81.2, 69.8, 58.5, 56.0, 42.4, 33.1, 28.2, 26.3, 23.2, 22.5, 13.9, 13.2. HRMS (ESI) calcd. for $C_{22}H_{30}O_2Na$ [M+Na]: 349.2143, found: 349.2175. (cx13009)

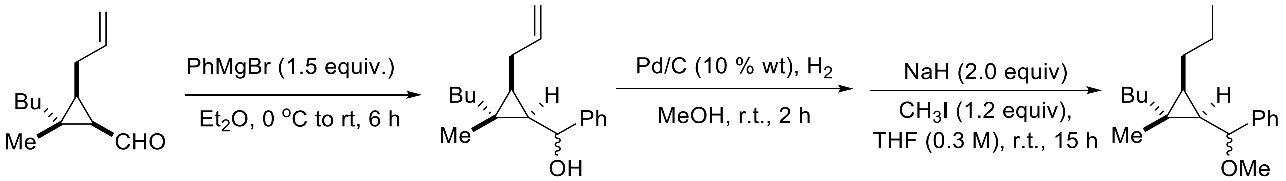

S4g' **S4g** **5g**

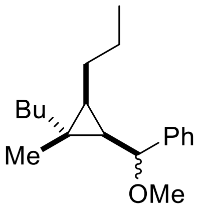

(($1R^*,2R^*,3S^*$)-3-allyl-2-butyl-2-methylcyclopropyl)(phenyl)methanol (**S4g**). A 50 mL oven-dried round-bottom flask was charged with aldehyde **S4g'** [3](3 mmol), and Et_2O (10 mL). PhMgBr (3 mL, 1.5 M in Et_2O, 1.5 equiv.) was added dropwise to the resulting mixture at 0 °C and then stirred for 8 h at room temperature. The reaction was quenched with an aqueous solution of NH_4Cl. The aqueous layer was extracted with

Et_2O (3×30 ml). The combined organic phases were dried over $NaSO_4$, filtered, and concentrated under reduced pressure. The resulting crude mixtures were analyzed by ^1H NMR to determine the *dr* (1/1). Then, the crude mixture was purified by column chromatography using PE/EA (10/1) as eluent to give the product **S4g** (2.4 mmol, 80% yield, *dr* = 5/4) as a colorless oil.^1H NMR (400 MHz, $CDCl_3$) δ 7.47 – 7.21 (m, 5H), 6.13-6.0 (m, 0.55H), 5.80-5.68 (m, 0.43H), 5.23 (ddd, *J* = 17.2, 3.6, 1.6 Hz, 0.56H), 5.10 (ddd, *J* = 10.0, 2.8, 1.2 Hz, 0.56H), 4.99 (ddd, *J* = 17.2, 3.6, 1.6 Hz, 0.44H), 4.92 (ddd, *J* = 10.4, 2.4, 1.2 Hz, 0.44H), 4.48 (dd, *J* = 10.0, 6.8 Hz, 1H), 2.40 – 2.13 (m, 1.60H), 2.09 – 1.99 (m, 0.44H), 1.89 (s, 0.56H), 1.70 (s, 0.45H), 1.43 – 1.01 (m, 10H), 0.94 – 0.68 (m, 4H). HRMS (APCI) calcd. for $C_{18}H_{25}O$ [M-H]: 257.1905, found: 257.1905.

S4g (2 mmol) was dissolved in MeOH (2 mL) and transferred to a 25 mL flame-dried Schlenk flask cooled under argon. 10% Pd/C (5 wt%) was added and a balloon containing hydrogen was attached. The reaction mixture was allowed to stir at ambient temperature for 1 h. Most of the Pd/C was removed by filtration through a celite pad and washed with MeOH (3 x 20 mL). The combined MeOH solution was concentrated to yield crude product. A 50 mL oven-dried round-bottom flask was charged with crude alcohol obtained, and THF (5 mL). NaH (4 mmol, 60% wt. dispersion in mineral oil, 2.0 equiv.) was added portion wise to the resulting mixture at 0 °C and stirred for 1 h at room temperature. Then, CH_3I (2.4 mmol) was added dropwise at 0 °C and stirred for further 15 h upon warming to room temperature. The reaction was quenched with an aqueous solution of NH_4Cl. The aqueous layer was extracted with Et_2O (3×20 mL). The combined organic phases were dried over $NaSO_4$, filtered, and concentrated under reduced pressure. The resulting crude mixtures were purified by column chromatography using PE/EA (20/1) as eluent to give the product **5g** as a colorless oil (1.4 mmol, 70% yield, *dr* = 1.2/1). ^1H NMR (400 MHz, $CDCl_3$) δ 7.38 – 7.24 (m, 5H), 3.86 (d, *J* = 3.2 Hz, 0.43H), 3.84 (d, *J* = 3.2 Hz, 0.52H), 3.15 (d, *J* = 0.8 Hz, 3H), 1.61 – 0.83 (m, 18.6 H), 0.78 – 0.72 (m, 1.75 H), 0.71 – 0.66 (m, 0.55H), 0.54 – 0.45 (m, 0.42 H). ^{13}C NMR (100 MHz, $CDCl_3$) δ 142.8, 142.6, 128.3, 128.2, 127.4, 127.3, 127.1, 126.9, 81.4, 81.1, 55.9, 43.0, 33.1, 33.0, 28.7, 28.3, 27.3, 27.0, 26.7, 26.4, 23.4, 23.2, 23.0, 22.7, 22.6, 22.2, 14.3, 14.14, 14.07, 14.0, 13.1, 12.5. HRMS (APCI) calcd. for $C_{18}H_{27}O$ [M-OMe]: 243.2113, found: 243.2127.(cx13085*)*

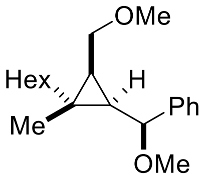

((*S**)-((1*R**,2*R**,3*S**)-2-hexyl-3-(methoxymethyl)-2-methylcyclopropyl)(methoxy)methyl)benzene (**5h**). Prepared according to the general procedure C. 83% yield (1.262 g, 4.15 mmol on 5.0 mmol scale), *dr* >95/5, colorless oil. ^{1}H NMR (400 MHz, $CDCl_3$) δ 7.39 – 7.22 (m, 5H), 3.92 – 3.84 (m, 1H), 3.83-3.77 (m, 1H), 3.50 – 3.35 (m, 4H), 3.14 (s, 3H), 1.28 – 0.91 (m, 15H), 0.80 (t, *J* = 7.2 Hz, 3H).^{13}C NMR (100 MHz, $CDCl_3$) δ 142.2, 128.3, 127.5, 126.7, 81.0, 69.7, 58.4, 55.9, 42.8, 33.4, 31.7, 29.1, 26.3, 26.0, 23.1, 22.4, 14.0, 13.2. HRMS (ESI) calcd. for $C_{20}H_{32}O_2Na$ [M+Na]: 327.2300, found: 327.2299.

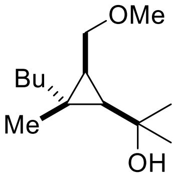

2-((1R*,2R*,3S*)-2-butyl-3-(methoxymethyl)-2-methylcyclopropyl)propan-2-ol (**5i**). Prepared according to the general procedure C. 89% yield (0.381 g, 1.78 mmol on 2 mmol scale), *dr* >95/5, pale yellow oil.^{1}H NMR (400 MHz, $CDCl_3$) δ 3.77 (dd, *J* = 10.4, 8.0 Hz, 1H), 3.61 (t, *J* = 10.4 Hz, 1H), 3.35 (s, 3H), 2.85 (s, 1H), 1.40 – 1.17 (m, 14H), 1.13 – 1.02 (m, 1H), 0.97 (dd, *J* = 17.6, 9.6 Hz, 1H), 0.87 (t, *J* = 7.2 Hz, 3H), 0.64 (d, *J* = 9.6 Hz, 1H). ^{13}C NMR (100 MHz, $CDCl_3$) δ70.1, 70.0, 57.7, 44.1, 37.7, 33.8, 29.2, 28.8, 25.6, 23.9, 22.9, 14.1, 12.4. HRMS (APCI) calcd. for $C_{13}H_{25}O$ [M-OH]: 197.1900, found: 197.1907. (cx12069)

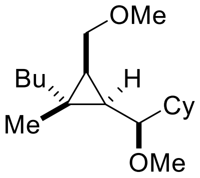

((*R**)-((1R*,2R*,3S*)-2-butyl-3-(methoxymethyl)-2-methylcyclopropyl)(methoxy) methyl)cyclohexane (**5j**). Prepared according to the general procedure C. 88% yield (0.880 g, 3.10 mmol on 3.5 mmol scale), *dr* >95/5, colorless oil. ^{1}H NMR (400 MHz, $CDCl_3$) δ 3.67 (dd, *J* = 10.8, 5.6 Hz, 1H), 3.34 (s, 3H), 3.30 – 3.21 (m, 4H), 2.84 (dd, *J* = 10.4, 5.6 Hz, 1H), 1.81 – 1.61(m, 6H), 1.40 – 0.95 (m, 15H), 0.87 (t, *J* = 7.2 Hz, 3H), 0.83 – 0.72 (m, 1H). ^{13}C NMR: (100 MHz,) delta 81.4, 70.6, 58.4, 54.9, 42.8, 41.3, 28.5, 28.4, 28.35, 28.31, 26.7, 26.6, 26.5, 25.7, 23.3, 23.0, 14.1, 13.6. HRMS (ESI) calcd. for $C_{18}H_{34}O_2Na$ [M+Na]: 305.2456, found: 305.2455. (cx12099)

III. Optimization Studies

Table S1. Optimization studies of bromination reaction

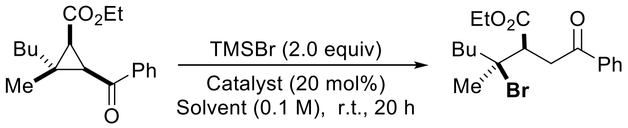

1a **2a**

Entry	Solvent	Catalyst	**2a**/%[a]	**1a**/%[a]
1	DCM	/	56	40
2	Toluene	/	52	42
3	THF	/	0	91
4	Et_2O	/	32	60
5	MeCN	/	42	58
6	DCM	$ZnBr_2$	70	0
7	DCM	BF_3	55	33
8	DCM	$EtAlCl_2$	12	80
9	DCM	$Yb(OTf)_3$	85	6
10[b]	DCM	$Yb(OTf)_3$	85	3
11[c]	DCM	$Yb(OTf)_3$	64	30

[a] ^{1}H NMR yield using CH_2Br_2 as internal standard, the *dr* value (>95:5 in all entries) was determined by ^{1}H NMR [b] DCM (0.2 M), $Yb(OTf)_3$ (10 mol%). [c] DCM (0.2 M), $Yb(OTf)_3$ (5 mol%).

Table S2. Optimization studies of chlorination reaction[a]

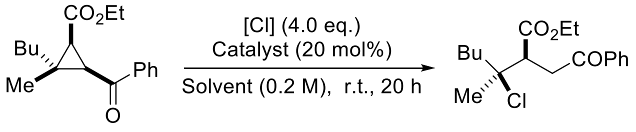

1a **3a**

Entry	Catalyst	Solvent	[Cl]	**3a**/%[b]	**1a**/%
1	Yb(OTf)3	DCM	TMSCl	0	92
2	Yb(OTf)3	MeCN	TMSCl	4	90
3	MgBr2	MeCN	TMSCl	20 [Br]	79
4	CuBr2	MeCN	TMSCl	15 [Br]	80
5	FeCl3	MeCN	TMSCl	0	90
6	Cu(OTf)2	MeCN	TMSCl	8	88
7	Cu(OTf)2	DCM	TMSCl	0	88
8	Zn(OTf)2	MeCN	TMSCl	16	27
9	Zn(OAc)2	MeCN	TMSCl	20	80
10	ZnBr2	MeCN	TMSCl	35+ unidentified products	
11	ZnCl2	MeCN	TMSCl	30~40	
12[c]	ZnCl2 (30 mol%)	MeCN (0.4 M)	TMSCl	40~60	
13[d]	ZnCl2 (50 mol%)	MeCN (0.4 M)	TMSCl	Complex mixture	
14	ZnCl2	MeCN (0.4 M)	DMPSCl	34	60
15	ZnCl2	MeCN (0.4 M)	TBSCl	13	76
16	ZnCl2	MeCN (0.4 M)	TESCl	18	70
17[c]	ZnCl2 (30 mol%)	MeCN (0.4 M)	DMPSCl	68	0
**18[c] [e]**	**ZnCl2 (30 mol%)**	**MeCN (0.4 M)**	**DMPSCl**	**67(65%)**	**0**

[a] **1a** (0.1 mmol), [Cl] (0.4 mmol) and catalyst (20 mol%) in solvent (0.1 M) stirred at rt for 12 h.

[b] ^{1}H NMR yield using CH_2Br_2 as an internal standard. All *Dr* values are greater than 95/5.

[c] $ZnCl_2$ (30 mol%). [d] $ZnCl_2$ (50 mol%). [e] DMPSCl (2.0 equiv.)

Table S3. Optimization studies of azidation reactions[a]

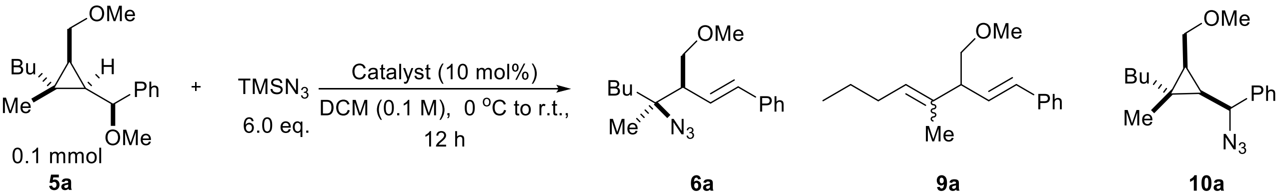

Entry	Cat*	T/°C	Solvent	**6a**/%[b]	**9a**/%	**10a**/%	*Dr*[c]
1	$SnCl_2$•$2H_2O$	0	DCM	24	42	16	N.D.
2	$Zn(OTf)_2$	0	DCM	8	72	4	N.D.
3	AgOTf	0	DCM	/	76	/	N.D.
4	CuCl	0	DCM	22	51	16	N.D.
5	$Cu(OTf)_2$	0	DCM	/	80	/	N.D.
6	$Yb(OTf)_3$	0	DCM	/	63	12	N.D.
7	$FeCl_2$	0	DCM	42	16	29	92/8
8	CuBr	0	DCM	37	33	12	88/12
9	$Cu(OAc)_2$	0	DCM	33	24	29	86/14
10	$FeCl_3$	0	DCM	50	30	12	82/16
11	$FeCl_3$ (2.5 mol%)	0	DCM	56	22	13	91/9
12	$FeCl_3$ (1.0 mol%)	0	DCM	52	15	22	91/9
13	$FeCl_3$ (1.0 mol%)	0	Toluene	46	7	35	94/6
14	$FeCl_3$ (1.0 mol%)	0	THF/MeCN/ Et_2O/Hexane			No reaction	
15	$FeCl_3$ (1.0 mol%)	0	HFIP	12	48	24	N.D.
16	$FeCl_3$ (2.5 mol%)	0	Toluene	60	11	15	90/10
17	$FeCl_3$ (1.0 mol%)	25	Toluene	57	11	28	86/14
18[d]	$FeCl_3$ (2.5 mol%)	-20	Toluene	64	10	12	91/9
19[d]	$FeCl_3$ (2.5 mol%)	-30	Toluene	62	6	19	95/5
20[d]	$FeCl_3$ (2.5 mol%)	-40	Toluene	62	7	17	94/6
21[e, f]	$FeCl_3$ (2.5 mol%)	-40	Toluene	55	12	20	94/6
**22[d, f]**	**$FeCl_3$ (2.5 mol%)**	**-30**	**Toluene**	**62**	**12**	**16**	**92/8**

[a] **5a** (0.1 mmol), $TMSN_3$ (0.6 mmol) and catalyst (10 mol%) in DCM (0.1 M) stirred at 0 °C for 12 h.
[b] ^1H NMR yield using CH_2Br_2 as an internal standard. [c] *Dr* determined by ^{13}C NMR of crude mixture.
[d] Reaction time: 20 h. [e] Reaction time: 36 h. [f] Using 0.5 mmol of **5a**.

IV. Experimental Procedures and Characterization Data

General procedure D for the bromination of cyclopropyl ketones

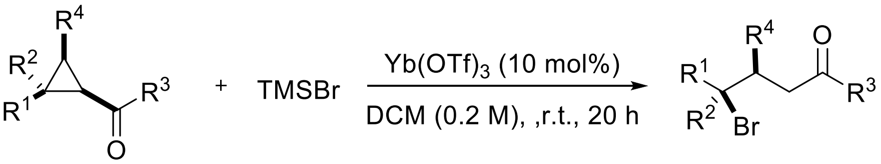

To a 25 mL flame-dried Schlenk flask cooled under argon, $Yb(OTf)_3$ (0.03 mmol), DCM (1.5 mL), cyclopropyl ketone (0.3 mmol) and TMSBr (0.6 mmol) were added in sequence. Then, the mixture was stirred at room temperature for 20 h. The resulting mixture was concentrated and purified by flash column chromatography using PE/EtOAc = 20/1 as the eluent to give the corresponding product.

The diastereomeric ratios were determined by ^{1}H NMR of crude mixture. As can be seen in Scheme S1, the two diastereomers **2a** and **2b** were independently prepared and analysis of their NMR shows different patterns. For all other addition of nucleophiles, α-protons adjacent to carbonyl groups of the two diastereomers are distinguished by ^{1}H NMR.

General procedure E for chlorination of cyclopropyl ketones

To a 25 mL flame-dried Schlenk flask cooled under argon, $ZnCl_2$ (0.03 mmol) was added and gently heated under vacuum. After cooling to room temperature, the flask is backfilled with argon. MeCN (3 mL), cyclopropyl ketone (0.3 mmol) and DMPSCl (0.6 mmol) were added in sequence. Then, the mixture was stirred at room temperature for 24 h. The resulting mixture was concentrated and purified by flash column chromatography using PE/EtOAc = 20/1 as eluent to give the corresponding product. The diastereomeric ratios were determined by ^{1}H NMR of crude mixture. As can be seen in Scheme S2, the two diastereomers **3a** and **3b** were independently prepared and analysis of their NMR shows different patterns. For all other addition of nucleophiles, α-protons adjacent to carbonyl groups of the two diastereomers are distinguished by ^{1}H NMR.

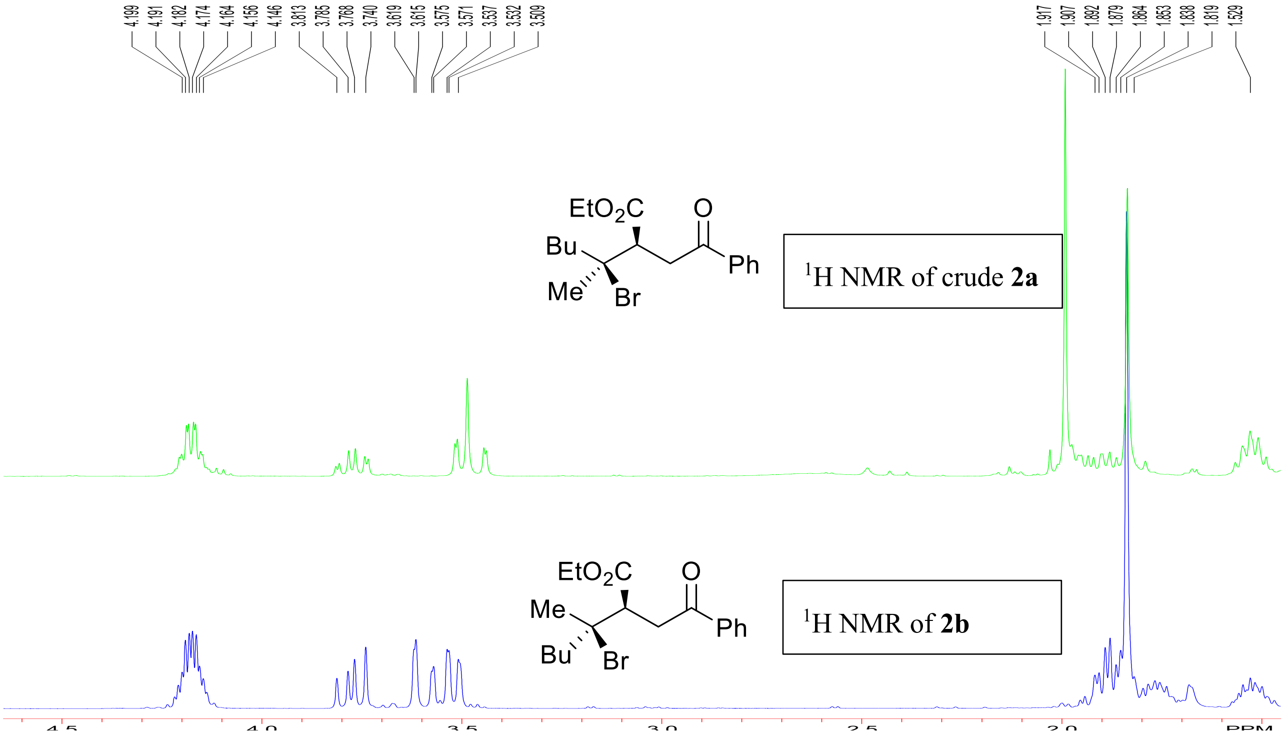

Scheme S1 Comparison between the ^{1}H NMR of crude **2a** and **2b**

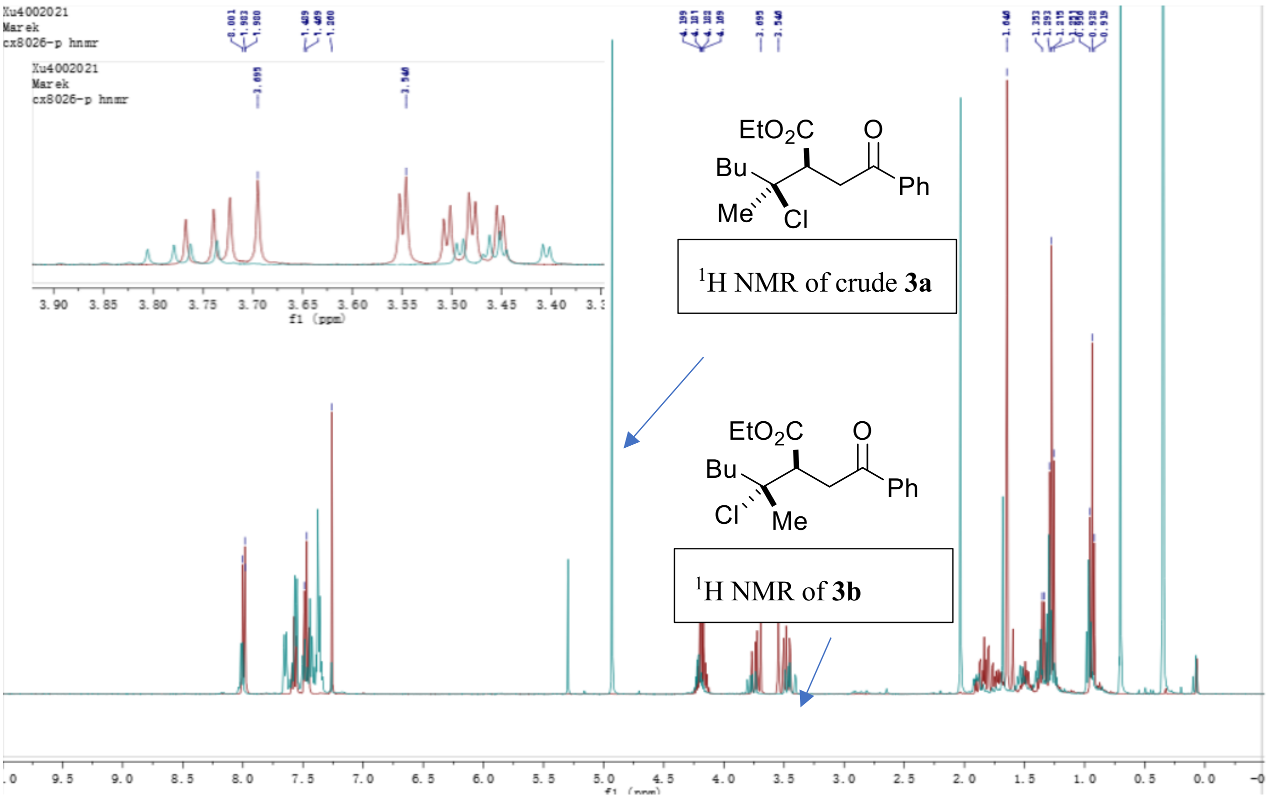

Scheme S2 Comparison between the ^{1}H NMR of crude **3a** and **3b**

General procedure F for azidation of cyclopropanes 5

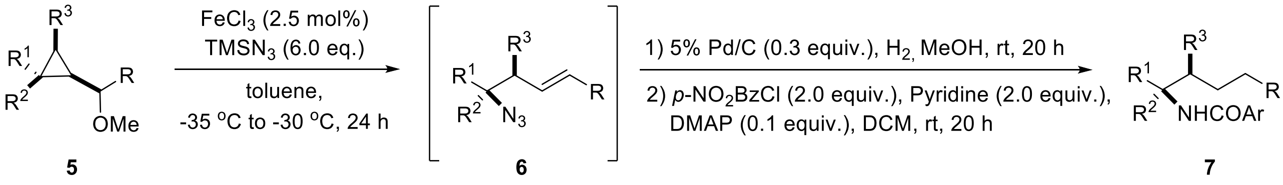

To a 25 mL flame-dried Schlenk flask cooled under argon, toluene (3 mL), **5** (0.4 mmol), $TMSN_3$ (2.4 mmol, 6.0 eq.) were added. Then, $FeCl_3$ (0.8 mL, 8 mg in 4 mL toluene) was added at -30 °C and stirred for 24 h. The resulting solution was concentrated, and the resulting crude mixtures were analyzed by ^1H NMR and ^{13}C NMR to determine the yield and *dr* using CH_2Br_2 (40 uL) as the internal standard. Crude azide **6** was dissolved in MeOH (2 mL) and transferred to a 25 mL flame dried Schlenk flask cooled under argon. Pd/C (0.3 equiv, 5 wt. %) was added and a balloon containing hydrogen was attached. The reaction mixture was allowed to stir at ambient temperature for 24 h. Most of the Pd/C was removed by filtration through a celite pad and washed with MeOH (3 x 20 mL). The combined MeOH solution was concentrated to yield crude amine. Crude amine was dissolved in DCM (3.0 mL) and cooled to 0 °C. Pyridine (0.8 mmol, 2.0 equiv. based on **5**), DMAP (0.04 mmol) and *p*-NO_2BzCl (0.8 mmol, 2.0 equiv. based on **5**) were added in sequence, and the cooling bath was removed. The reaction mixture was stirred at ambient temperature for 20 h then diluted with DCM (30 mL) and washed with water (10 mL). The combined organic phases were dried over $NaSO_4$, filtered, and concentrated under reduced pressure. The crude mixture was purified by column chromatography using PE/EA (5/1) as eluent to give the amide **7**.

General procedure G for azidation of cyclopropanes 5

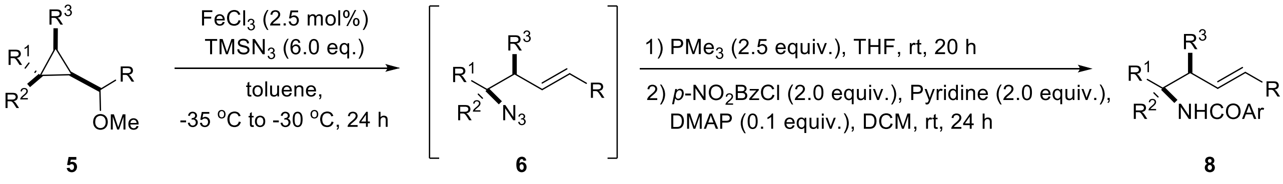

The procedure for preparation of crude azide **6** (from 0.4 mmol **5**) is the same as general procedure F. Crude azide **6** was dissolved in THF (2 mL) and transferred to a 25 mL flame dried Schlenk flask cooled under argon. H_2O (50 uL), PMe_3 (1.0 mL, 1.0 M in 2-MeTHF) were added. The reaction mixture was allowed to stir at ambient temperature for 20 h. The reaction mixture was condensed,

and the corresponding amine was dissolved in DCM (3.0 mL) and cooled to 0 °C. Pyridine (0.8 mmol, 2.0 equiv. based on **5**), DMAP (0.04 mmol) and *p*-NO_2BzCl (0.8 mmol, 2.0 equiv. based on **5**) were added in sequence, and the cooling bath was removed. The reaction mixture was stirred at ambient temperature for 24 hours then diluted with DCM (30 mL) and washed with water (20 mL). The aqueous layer was extracted with DCM (10 mL). The combined organic phases were dried over $NaSO_4$, filtered, and concentrated under reduced pressure. The crude mixture was purified by column chromatography using PE/EA (4/1) as the eluent to give the amide **8**.

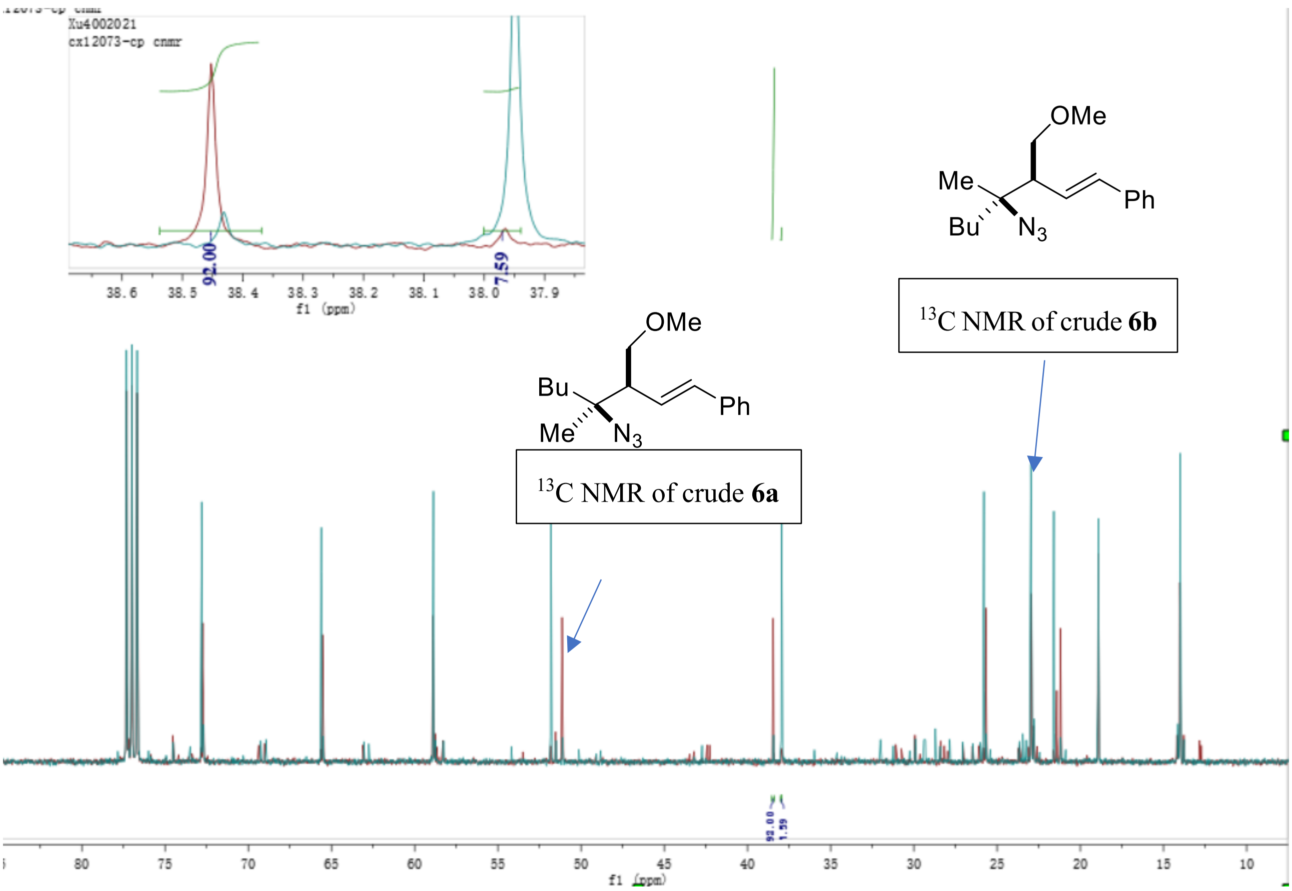

Scheme S3 Comparison between the ^{13}C NMR of crude **6a** and crude **6b**

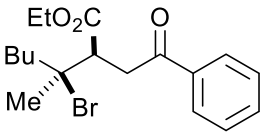

Ethyl (2*R**,3*S**)-3-bromo-3-methyl-2-(2-oxo-2-phenylethyl)heptanoate (**2a**). Prepared according to the general procedure D. 80% yield (0.030 g, 0.08 mmol on 0.1 mmol scale), *dr* >95/5, pale yellow oil. ^{1}H NMR (400 MHz, $CDCl_3$) δ 8.05 – 7.94 (m, 2H), 7.62 – 7.54 (m, 1H), 7.47 (t, *J* = 7.6 Hz, 2H), 4.24 – 4.13 (m, 2H), 3.84 – 3.70 (m, 1H), 3.55 – 3.40 (m, 2H), 2.04 – 1.86 (m, 2H), 1.85 (s, 3H), 1.58-1.48 (m, 2H), 1.42 – 1.32 (m, 2H), 1.31 – 1.25 (m, 3H), 0.95 (t, *J* = 7.2 Hz, 3H). ^{13}C NMR (100 MHz, $CDCl_3$) δ 197.9, 171.5, 136.4,

133.3, 128.6, 128.1, 70.9, 61.0, 53.4, 42.2, 39.4, 30.6, 27.9, 22.7, 14.1, 14.0. HRMS (APCI) calcd. for $C_{18}H_{26}BrO_3$ [M+H]: 369.1060, found: 369.1048.

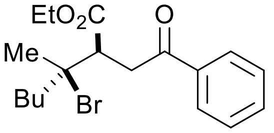

Ethyl (2*R**,3*R**)-3-bromo-3-methyl-2-(2-oxo-2-phenylethyl)heptanoate (**2b**). Prepared according to the general procedure D. 71% yield (0.079 g, 0.213 mmol on 0.3 mmol scale), *dr* >95/5, pale yellow oil. ^{1}H NMR (400 MHz, $CDCl_3$) δ 7.99 (d, *J* = 8.0 Hz, 2H), 7.57 (dd, *J* = 7.6, 7.2 Hz, 1H), 7.47 (dd, *J* = 7.6, 7.2 Hz, 2H), 4.24 – 4.12 (m, 2H), 3.78 (dd, *J* = 18.0, 11.2 Hz, 1H), 3.63 – 3.49 (m, 2H), 2.00 – 1.68 (m, 6H), 1.58 – 1.46 (m, 1H), 1.41 – 1.31 (m, 2H), 1.27 (t, *J* = 7.2 Hz, 3H), 0.94 (t, *J* = 7.2 Hz, 3H). ^{13}C NMR (100 MHz, $CDCl_3$) δ198.1, 171.3, 136.4, 133.3, 128.6, 128.1, 71.4, 61.0, 51.6, 45.1, 40.0, 29.1, 27.9, 22.6, 14.1, 14.0. HRMS (APCI) calcd. for $C_{18}H_{26}BrO_3$ [M+H]: 369.1060, found: 369.1046.

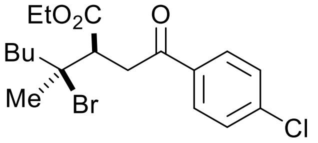

Ethyl (2*R**,3*S**)-3-bromo-2-(2-(4-chlorophenyl)-2-oxoethyl)-3-methylheptanoate (**2c**). Prepared according to the general procedure D. 75% yield (0.091 g, 0.225 mmol on 0.3 mmol scale), *dr* >95/5, pale yellow solid. ^{1}H NMR (400 MHz, $CDCl_3$) δ 7.97 – 7.89 (m, 2H), 7.46 – 7.42 (m, 2H), 4.24 – 4.12 (m, 2H), 3.74 (dd, *J* = 17.2, 10.4 Hz, 1H), 3.52-3.40 (m, 2H), 2.02 – 1.85 (m, 2H), 1.84 (s, 3H), 1.58-1.48 (m, 2H), 1.40-1.30 (m, 2H), 1.27 (t, *J* = 7.2 Hz, 3H), 0.94 (t, *J* = 7.2 Hz, 3H). ^{13}C NMR (100 MHz, CDCl3) δ 196.8, 171.4, 139.8, 134.7, 129.5, 128.9, 70.8, 61.0, 53.4, 42.1, 39.3, 30.6, 27.8, 22.7, 14.1, 14.0. HRMS (APCI) calcd. for $C_{18}H_{25}BrClO_3$ [M+H]: 403.0670, found: 403.0642.

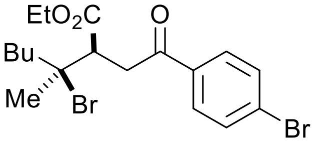

Ethyl (2*R**,3*S**)-3-bromo-2-(2-(4-bromophenyl)-2-oxoethyl)-3-methylheptanoate (**2d**). Prepared according to the general procedure D. 80% yield (0.108 g, 0.24 mmol on 0.3 mmol scale), *dr* >95/5, pale yellow oil. ^{1}H NMR (400 MHz, $CDCl_3$) δ 7.85 (d, *J* = 7.6 Hz, 2H), 7.61 (d, *J* = 7.6 Hz, 2H), 4.24 – 4.13 (m, 2H), 3.74 (dd, *J* = 17.6, 10.8 Hz, 1H), 3.53-3.39 (m, 2H), 2.02 – 1.85 (m,2H), 1.84 (s, 3H), 1.59 – 1.49 (m, 2H), 1.40-1.32 (m, 2H), 1.28 (t, *J* = 7.2 Hz, 3H), 0.95 (t, *J* = 7.2 Hz, 3H). ^{13}C NMR: (100 MHz, $CDCl_3$) δ197.0, 171.4, 135.1, 131.9, 129.6, 128.6, 70.8, 61.1, 53.4, 42.1, 39.3, 30.6, 27.9, 22.7, 14.1, 14.0. HRMS (APCI) calcd. For $C_{18}H_{25}Br_2O_3$ [M+H]: 447.0165, found: 447.0163.

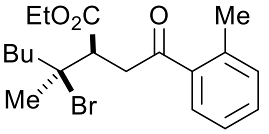

Ethyl (2*R**,3*S**)-3-bromo-3-methyl-2-(2-oxo-2-(o-tolyl)ethyl)heptanoate (**2e**). Prepared according to the general procedure D. 72% yield (0.084 g, 0.22 mmol on 0.3 mmol scale), *dr* >95/5, pale yellow oil. ^{1}H NMR (400 MHz, $CDCl_3$) δ 7.73 (dd, *J* = 7.6, 1.2 Hz, 2H), 7.42-7.35 (m, 1H), 7.32 – 7.22 (m, 2H), 4.24 – 4.15 (m, 2H), 3.71 (dd, *J* = 17.6, 11.2 Hz, 1H), 3.50 (dd, *J* = 11.2, 2.8 Hz, 1H), 3.36 (dd, *J* = 17.6, 2.8 Hz, 1H), 2.47 (s, 3H), 2.00 – 1.85 (m, 2H), 1.84 (s, 3H), 1.58-1.48 (m, 2H), 1.40-1.32 (m, 2H), 1.29 (t, *J* = 7.2 Hz, 3H), 0.94 (t, *J* = 7.2 Hz, 3H). ^{13}C NMR (100 MHz, $CDCl_3$) δ 201.8, 171.5, 138.1, 137.3, 131.9, 131.5, 128.6, 125.7, 70.8, 61.0, 53.6, 42.3, 42.1, 30.6, 27.9, 22.7, 21.2, 14.1, 14.0. HRMS (APCI) calcd. for $C_{19}H_{28}BrO_3$ [M+H]: 383.1216, found: 383.1237.

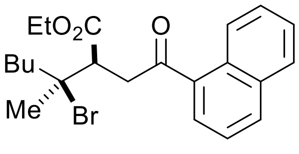

Ethyl (2*R**,3*S**)-3-bromo-3-methyl-2-(2-(naphthalen-1-yl)-2-oxoethyl) heptanoate (**2f**). Prepared according to the general procedure D. 62% yield (0.0780 g, 0.186 mmol on 0.3 mmol scale), *dr* >95/5, pale yellow oil. ^{1}H NMR (400 MHz, $CDCl_3$) δ 8.55 (d, *J* = 8.4 Hz, 1H), 8.03-7.96 (m, 2H), 7.90 – 7.84 (m, 1H), 7.62 – 7.50 (m, 3H), 4.22 (q, *J* = 7.2 Hz, 2H), 3.89 (dd, *J* = 17.6, 11.2 Hz, 1H), 3.62 (dd, *J* = 11.2, 2.8 Hz, 1H), 3.52 (dd, *J* = 17.6, 2.8 Hz, 1H)., 2.05 – 1.90 (m, 2H), 1.88 (s, 3H), 1.60-1.51 (m, 2H), 1.40-1.34 (m, 2H), 1.30 (t, *J* = 7.2 Hz, 3H), 0.95 (t, *J* = 7.2 Hz, 3H). ^{13}C NMR (100 MHz, $CDCl_3$) δ 202.1, 171.6, 135.3, 133.9, 132.9, 130.0, 128.4, 128.0, 127.8, 126.5, 125.7, 124.4, 70.9, 61.1, 53.8, 42.7, 42.3, 30.6, 27.9, 22.7, 14.1, 14.0. HRMS (APCI) calcd. for $C_{22}H_{28}BrO_3$ [M+H]: 419.1216, found: 419.1215.

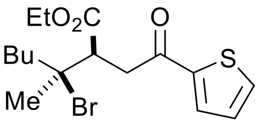

Ethyl (2*R**,3*S**)-3-bromo-3-methyl-2-(2-oxo-2-(thiophen-2-yl)ethyl) heptanoate (**2g**). Prepared according to the general procedure D using 3.0 equivalent TMSBr. 76% yield (0.086 g, 0.228 mmol on 0.3 mmol scale), *dr* >95/5, pale yellow oil. ^{1}H NMR (400 MHz, $CDCl_3$) δ 7.80 (d, *J* = 4.0 Hz, 1H), 7.64 (d, *J* = 4.8 Hz, 1H), 7.13 (t, *J* = 4.4 Hz, 1H), 4.22-4.10 (m, 2H), 3.69 (dd, *J* = 16.8, 10.8 Hz, 1H), 3.49 (dd, *J* = 10.8, 2.4 Hz, 1H), 3.41 (dd, *J* = 16.8, 2.4 Hz, 1H), 1.99 – 1.85 (m, 2H), 1.83 (s, 3H), 1.57 – 1.46 (m, 2H), 1.41 – 1.29 (m, 2H), 1.26 (t, *J* = 7.2 Hz, 3H), 0.94 (t, *J* = 7.2 Hz, 3H). ^{13}C NMR (100 MHz, $CDCl_3$) δ 190.7, 171.3, 143.4, 133.8, 132.2, 128.1, 70.8, 61.0, 53.3, 42.2, 39.7, 30.4, 27.8, 22.7, 14.1, 14.0. HRMS (APCI) calcd. for $C_{16}H_{24}BrO_3S$ [M+H]: 375.0624, found: 375.0615.

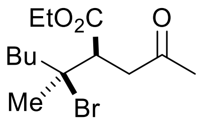

Ethyl (2*R**,3*S**)-3-bromo-3-methyl-2-(2-oxopropyl)heptanoate (**2h**). Prepared according to the general procedure D. 76% yield (0.070 g, 0.228 mmol on 0.3 mmol scale), *dr* >95/5, pale yellow oil. ^{1}H NMR (400 MHz, $CDCl_3$) δ 4.15 (q, *J* = 6.8 Hz, 2H), 3.34-3.16 (m, 2H), 2.90 (dd, *J* = 17.6, 2.4 Hz, 1H), 2.17 (s, 3H), 1.91 – 1.78 (m, 2H), 1.76 (s, 3H), 1.50-1.41 (m, 2H), 1.37 – 1.25 (m, 5H), 0.91 (t, *J* = 7.2Hz, 3H). ^{13}C NMR (100 MHz, $CDCl_3$) δ 206.3, 171.4, 70.5, 60.9, 53.0, 43.9, 42.1, 30.4, 29.9, 27.8, 22.7, 14.1, 14.0. HRMS (APCI) calcd. for $C_{13}H_{24}BrO_3$ [M+H]: 307.0903, found: 307.0892.

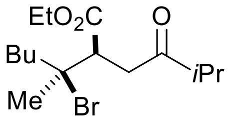

Ethyl (2*R**,3*S**)-3-bromo-3-methyl-2-(3-methyl-2-oxobutyl)heptanoate (**2i**). Prepared according to the general procedure D. 75% yield (0.075 g, 0.224 mmol on 0.3 mmol scale), *dr* >95/5, pale yellow oil. ^{1}H NMR (400 MHz, $CDCl_3$) δ 4.21 – 4.08 (m, 2H), 3.32-3.18 (m, 2H), 2.91 (d, *J* = 17.2 Hz, 1H), 2.63 (hept, *J* = 6.8 Hz, 1H), 1.90 – 1.78 (m, 2H), 1.77 (s, 3H), 1.53 – 1.40 (m, 2H), 1.38 – 1.28 (m, 2H), 1.26 (t, *J* = 7.2 Hz, 3H), 1.10 (d, *J* = 7.2 Hz, 6H), 0.91 (t, *J* = 7.2 Hz, 3H).^{13}C NMR (100 MHz, $CDCl_3$) δ 212.5, 171.5, 70.8, 60.9, 53.1, 42.1, 40.8, 40.7, 30.4, 27.8, 22.7, 18.2, 18.1, 14.1, 14.0. HRMS (APCI) calcd. for $C_{15}H_{28}BrO_3$ [M+H]: 335.1216, found: 335.1209.

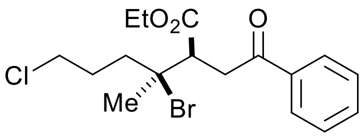

Ethyl (2*R**,3*S**)-3-bromo-6-chloro-3-methyl-2-(2-oxo-2-phenylethyl) hexanoate (**2j**). Prepared according to the general procedure D. 67% yield (0.078 g, 0.20 mmol on 0.3 mmol scale), *dr* >95/5, pale yellow oil. ^{1}H NMR (400 MHz, $CDCl_3$) δ 7.99 (d, *J* = 8.0 Hz, 2H), 7.58 (t, *J* = 7.2 Hz, 1H), 7.47 (t, *J* = 7.6 Hz, 2H), 4.19 (q, *J* = 7.2 Hz, 2H), 3.83-3.73 (m, 1H), 3.63-3.45 (m, 4H), 2.22 – 1.99 (m, 4H), 1.86 (s, 3H), 1.28 (t, *J* = 7.2 Hz, 3H). ^{13}C NMR (100 MHz, $CDCl_3$) δ 197.7, 171.3, 136.3, 133.4, 128.6, 128.1, 69.2, 61.1, 53.5, 44.6, 39.7, 39.3, 30.5, 29.0, 14.1. HRMS (APCI) calcd. for $C_{17}H_{23}BrClO_3$ [M+H]: 389.0514, found: 389.0521.

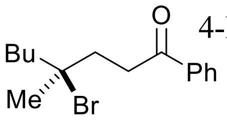

4-Bromo-4-methyl-1-phenyloctan-1-one (**2k**). Prepared according to the general procedure D. 80% ^{1}H NMR yield without isolation, pale yellow oil (containing CH_2Br_2) ^{1}H NMR (400 MHz, $CDCl_3$) δ 8.05 – 7.99 (m, 2H), 7.62-7.54 (m, 1H), 7.52-7.45 (m, 2H), 3.30 – 3.24 (m, 2H), 2.35 – 2.24 (m, 1H), 2.23 – 2.14 (m, 1H), 2.00-1.91 (m, 1H), 1.89-1.80 (m, 1H),

1.76 (s, 3H), 1.54 – 1.42 (m, 2H), 1.40 – 1.30 (m, 2H), 0.93 (t, J = 7.2 Hz, 3H).^{13}C NMR (100 MHz, $CDCl_3$) δ 199.4, 136.7, 133.1, 128.6, 128.1, 73.0, 45.7, 39.1, 35.7, 31.4, 27.9, 18.9, 14.0. HRMS (APCI) calcd. for $C_{15}H_{22}BrO$ [M+H]: 297.0849, found: 297.0846.

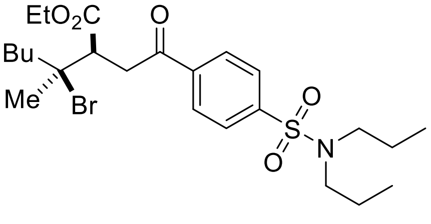

Ethyl (2*R**,3*S**)-3-bromo-2-(2-(4-(*N,N*-dipropylsulfamoyl)-phenyl)-2-oxoethyl)-3-methylheptanoate (**2l**). Prepared according to the general procedure D. 69% yield (0.073 g, 0.138 mmol on 0.2 mmol scale), *dr* >95/5, pale yellow oil. ^{1}H NMR (400 MHz, $CDCl_3$) δ 8.12-8.07 (m, 2H), 7.93-7.86 (m, 2H), 4.24-4.12 (m, 2H), 3.86-3.76 (m, 1H), 3.56 – 3.42 (m, 2H), 3.15 – 3.04 (m, 4H), 2.02 – 1.80 (m, 5H), 1.59-1.48 (m, 6H), 1.42-1.34 (m, 2H), 1.28 (t, J = 7.2 Hz, 3H), 0.94 (t, J = 7.2 Hz, 3H), 0.86 (t, J = 7.2 Hz, 6H). ^{13}C NMR: (100 MHz, $CDCl_3$) δ 197.0, 171.3, 144.4, 139.0, 128.7, 127.3, 70.7, 61.2, 53.4, 49.9, 42.1, 39.8, 30.7, 27.9, 22.7, 21.9, 14.1, 14.0, 11.1. HRMS (APCI) calcd. for $C_{24}H_{39}BrNO_5S$ [M+H]: 532.1732, found: 532.1729.

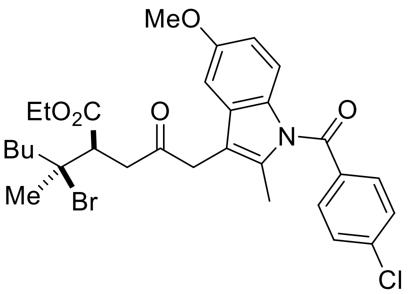

Ethyl (2*R**,3*S**)-3-bromo-2-(3-(1-(4-chlorobenzoyl)-5-methoxy-2-methyl-1H-indol-3-yl)-2-oxopropyl)-3-methylheptanoate (**2m**). Prepared according to the general procedure D. 76% yield (0.046 g, 0.076 mmol on 0.1 mmol scale), *dr* >95/5, pale yellow oil. ^{1}H NMR (400 MHz, $CDCl_3$) δ 7.69 – 7.64 (m, 2H), 7.50 – 7.43 (m, 2H), 6.90 (d, J = 9.2 Hz, 1H), 6.84 (d, J = 2.4 Hz, 1H), 6.67 (dd, J = 9.2, 2.4 Hz, 1H), 4.16 -4.00 (m, 2H), 3.83 (s, 3H), 3.77 (s, 2H), 3.34 – 3.25 (m, 2H), 3.01 – 2.90 (m, 1H), 2.34 (s, 3H), 1.87 – 1.75 (m, 2H), 1.74 (s, 3H), 1.50 – 1.40 (m, 2H), 1.37 – 1.27 (m, 2H), 1.23 (t, J = 7.2 Hz, 3H), 0.90 (t, J = 7.2 Hz, 3H).^{13}C NMR (100 MHz, $CDCl_3$) δ 205.4, 171.3, 168.3, 156.1, 139.3, 135.8, 133.8, 131.2, 130.9, 130.5, 129.1, 115.0, 112.2, 111.9, 100.9, 70.6, 61.0, 55.7, 53.1, 42.3, 42.1, 38.9, 30.5, 27.8, 22.6, 14.1, 14.0, 13.5. HRMS (APCI) calcd. for $C_{30}H_{36}BrClNO_5$ [M+H]: 604.1465, found: 604.1483.

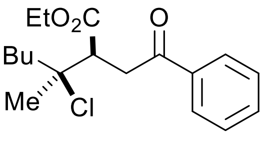

Ethyl (2*R**,3*S**)-3-chloro-3-methyl-2-(2-oxo-2-phenylethyl)heptanoate (**3a**). Prepared according to the general procedure E. 65% yield (0.042 g, 0.13 mmol on 0.2 mmol scale), *dr* >95/5, colorless oil. ^{1}H NMR (400 MHz, $CDCl_3$) δ 8.02

– 7.96 (m, 2H), 7.60 – 7.54 (m, 1H), 7.50-7.48 (m, 2H), 4.24-414 (m, 2H), 3.75 (dd, *J* = 17.6, 10.8 Hz, 1H), 3.48-3.35 (m, 2H), 1.90-1.84 (m, 2H), 1.66 (s, 3H), 1.56 – 1.46 (m, 2H), 1.40-1.32 (m, 2H), 1.28 (t, *J* = 7.2 Hz, 3H), 0.94 (t, *J* = 7.2 Hz, 3H). ^{13}C NMR (100 MHz, $CDCl_3$) δ198.0, 171.9, 136.4, 133.3, 128.6, 128.1, 73.3, 60.9, 52.4, 41.1, 38.1, 28.9, 26.7, 22.8, 14.1, 14.0. HRMS (APCI) calcd. for $C_{18}H_{26}ClO_3$ [M+H]: 325.1565, found: 325.1536.

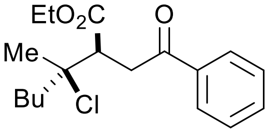

Ethyl (2*R**,3*R**)-3-chloro-3-methyl-2-(2-oxo-2-phenylethyl)heptanoate (**3b**). Prepared according to the general procedure E. 60% yield (0.039 g, 0.12 mmol on 0.2 mmol scale), *dr* >95/5, colorless oil. ^{1}H NMR (400 MHz, $CDCl_3$) δ 8.02-7.96 (m, 2H), 7.61 – 7.53 (m, 1H), 7.50-7.44 (m, 2H), 4.26 – 4.11 (m, 2H), 3.73 (dd, *J* = 17.6, 11.2 Hz, 1H), 3.53 (dd, *J* = 17.6, 2.8 Hz, 1H), 3.47 (dd, *J* = 11.2, 2.8 Hz, 1H), 1.92 – 1.67 (m, 3H), 1.65 (s, 3H), 1.54-1.42 (m, 1H), 1.40-1.30 (m, 2H), 1.27 (t, *J* = 7.2 Hz, 3H), 0.94 (t, *J* = 7.2 Hz, 3H). ^{13}C NMR (100 MHz, $CDCl_3$) δ 198.2, 171.9, 136.5, 133.3, 128.6, 128.1, 73.6, 61.0, 50.9, 43.7, 38.4, 27.5, 26.6, 22.7, 14.1, 14.0. HRMS (APCI) calcd. for $C_{18}H_{26}ClO_3$ [M+H]: 325.1565, found: 325.1578.

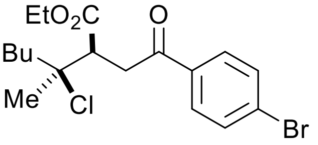

Ethyl (2*R**,3*S**)-2-(2-(4-bromophenyl)-2-oxoethyl)-3-chloro-3-methylheptanoate (**3c**). Prepared according to the general procedure E. 50% yield (0.040 g, 0.10 mmol on 0.2 mmol scale), *dr* >95/5, pale yellow oil. ^{1}H NMR (400 MHz, $CDCl_3$) δ 7.89 – 7.82 (m, 2H), 7.64 – 7.59 (m, 2H), 4.25 – 4.16 (m, 2H), 3.70 (dd, *J* = 17.6, 10.8 Hz, 1H), 3.47-3.32 (m, 2H), 1.90 – 1.81 (m, 2H), 1.65 (s, 3H), 1.55 – 1.46 (m, 2H), 1.40-1.32 (m, 2H), 1.28 (t, *J* = 7.2 Hz, 3H), 0.94 (t, *J* = 7.2 Hz, 3H).^{13}C NMR (100 MHz, $CDCl_3$) δ 197.1, 171.7, 135.2, 131.9, 129.6, 128.6, 73.3, 61.0, 52.5, 41.0, 38.0, 28.9, 26.7, 22.8, 14.1, 14.0. HRMS (APCI) calcd. for $C_{18}H_{25}BrClO_3$ [M+H]: 403.0670, found: 403.0698.

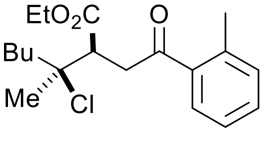

Ethyl (2*R**,3*S**)-3-chloro-3-methyl-2-(2-oxo-2-(o-tolyl)ethyl)heptanoate (**3d**). Prepared according to the general procedure E. 63% yield (0.043 g, 0.126 mmol on 0.2 mmol scale), *dr* >95/5, colorless oil. ^{1}H NMR (400 MHz, $CDCl_3$) δ 7.72 (d, *J* = 8.0 Hz, 2H), 7.42 – 7.35 (m, 1H), 7.31 – 7.20 (m, 2H), 4.25 – 4.14 (m, 2H), 3.68 (dd, *J* = 17.6, 11.2 Hz, 1H), 3.44 (dd, *J* = 11.2, 2.8 Hz, 1H), 3.28 (dd, *J* = 17.6, 2.8 Hz, 1H), 2.46 (s, 3H), 1.92 – 1.77 (m, 2H), 1.65 (s, 3H), 1.56 – 1.44 (m, 2H), 1.40 – 1.32 (m, 2H), 1.29 (t, *J* = 7.2 Hz, 3H),

0.93 (t, J = 7.2 Hz, 3H). ^{13}C NMR (100 MHz, $CDCl_3$) δ 202.0, 171.9, 138.1, 137.4, 131.9, 131.5, 128.6, 125.7, 73.3, 61.0, 52.6, 41.1, 40.9, 28.9, 26.7, 22.8, 21.2, 14.1, 14.0. HRMS (APCI) calcd. for $C_{19}H_{28}ClO_3$ [M+H]: 339.1721, found: 339.1741.

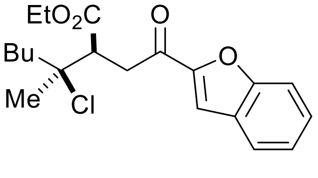

Ethyl (2*R**,3*S**)-2-(2-(benzofuran-2-yl)-2-oxoethyl)-3-chloro-3-methylheptanoate (**3e**). Prepared according to the general procedure E. 65% yield (0.042 g, 0.13 mmol on 0.2 mmol scale), *dr* >95/5, colorless oil. ^{1}H NMR (400 MHz, $CDCl_3$) δ 7.71 (d, J = 8.0 Hz, 1H), 7.61 – 7.55 (m, 2H), 7.52 – 7.45 (m, 1H), 7.36 – 7.29 (m, 1H), 4.19 (q, J = 7.2 Hz, 2H), 3.73 (dd, J = 17.6, 10.8 Hz, 1H), 3.48 (dd, J = 10.8, 2.8 Hz, 1H), 3.48 (dd, J = 17.6, 2.8 Hz, 1H),1.94 – 1.84 (m, 2H), 1.67 (s, 3H), 1.57 – 1.47 (m, 2H), 1.43 – 1.31 (m, 2H), 1.27 (t, J = 7.2 Hz, 3H), 0.95 (t, J = 7.2 Hz, 3H).^{13}C NMR (100 MHz, $CDCl_3$) δ189.0, 171.6, 155.6, 152.1, 128.4, 126.9, 124.0, 123.3, 113.0, 112.5, 73.1, 61.0, 52.0, 41.1, 38.3, 28.8, 26.6, 22.8, 14.1, 14.0. HRMS (APCI) calcd. for $C_{20}H_{26}ClO_4$ [M+H]: 365.1514, found: 365.1489.

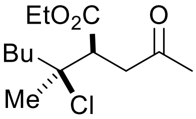

Ethyl (2*R**,3*S**)-3-chloro-3-methyl-2-(2-oxopropyl)heptanoate (**3f**). Prepared according to the general procedure E. 58% yield (0.046 g, 0.174 mmol on 0.3 mmol scale), *dr* >95/5, colorless oil. ^{1}H NMR (400 MHz, $CDCl_3$) δ 4.20-4.10 (m, 2H), 3.25 (dd, J = 11.2, 2.0 Hz, 1H), 3.17 (dd, J = 17.2, 11.2 Hz, 1H), 2.84 (dd, J = 17.2, 2.0 Hz, 1H), 2.17 (s, 3H), 1.84 – 1.69 (m, 2H), 1.58 (s, 3H), 1.50 – 1.39 (m, 2H), 1.38 – 1.29 (m, 2H),1.27 (t, J = 7.2 Hz, 3H), 0.91 (t, J = 7.2 Hz, 3H). ^{13}C NMR (100 MHz, $CDCl_3$) δ 206.5, 171.8, 73.0, 60.9, 52.0, 42.6, 41.0, 29.9, 28.7, 26.6, 22.8, 14.1, 14.0. HRMS (APCI) calcd. for $C_{13}H_{24}ClO_3$ [M+H]: 263.1408, found: 263.1434.

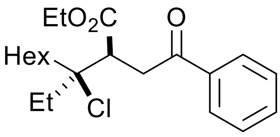

Ethyl (2*R**,3*S**)-3-chloro-3-ethyl-2-(2-oxo-2-phenylethyl)nonanoate (**3g**). Prepared according to the general procedure E. 57% yield (0.042 g, 0.114 mmol on 0.2 mmol scale), *dr* >95/5, colorless oil. ^{1}H NMR (400 MHz, $CDCl_3$) δ 8.03 – 7.96 (m, 2H), 7.61 – 7.52 (m, 1H), 7.50-7.43 (m, 2H), 4.25 – 4.10 (m, 2H), 3.80 (dd, J = 17.6, 10.8 Hz, 1H), 3.55 (dd, J = 10.8, 2.8 Hz, 1H), 3.36 (dd, J = 17.6, 2.8 Hz, 1H), 2.02-1.78 (m, 4H), 1.54 – 1.39 (m, 2H), 1.36 – 1.22 (m, 9H), 1.09 (t, J = 7.2 Hz, 3H), 0.90 (t, J = 7.2 Hz, 3H). ^{13}C NMR (100 MHz, $CDCl_3$) δ 198.3, 171.8, 136.5, 133.3, 128.6, 128.1, 77.7, 60.9, 48.9, 39.1, 38.0, 32.4, 31.7, 29.5, 24.2, 22.6, 14.1, 14.0, 8.7. HRMS (APCI) calcd. for $C_{21}H_{32}ClO_3$ [M+H]: 367.2034, found:

367.2033.

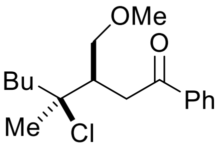

(3*R**,4*S**)-4-chloro-3-(methoxymethyl)-4-methyl-1-phenyloctan-1-one (**3h**). To a 25 mL flame-dried Schlenk flask cooled under argon, MeCN (3 mL), cyclopropyl ketone (0.2 mmol) and DMPSCl (0.8 mmol) were added in sequence. Then, the mixture was stirred at 50 °C for 30 h. 68% yield (0.040 g, 0.136 mmol on 0.2 mmol scale), *dr* >95/5, colorless oil. ^{1}H NMR (400 MHz, $CDCl_3$) δ 8.03 – 7.97 (m, 2H), 7.59 – 7.52 (m, 1H), 7.50 – 7.44 (m, 2H), 3.64 (dd, *J* = 9.6, 4.4 Hz, 1H), 3.47 (dd, *J* = 9.6, 7.2 Hz, 1H), 3.30 – 3.13 (m, 5H), 2.98 – 2.89 (m, 1H), 1.93 – 1.80 (m, 2H), 1.54 (s, 3H), 1.52 – 1.42 (m, 2H), 1.39 – 1.29 (m, 2H), 0.93 (t, *J* = 7.2 Hz, 3H). ^{13}C NMR (100 MHz, $CDCl_3$) δ 199.3, 137.3, 132.9, 128.6, 128.2, 77.5, 72.7, 58.7, 44.3, 42.5, 37.7, 28.7, 26.9, 23.0, 14.1. HRMS (APCI) calcd. for $C_{17}H_{26}ClO_2$ [M+H]: 297.1616, found: 297.1637.

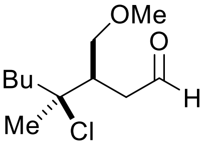

(3*R**,4*S**)-4-chloro-3-(methoxymethyl)-4-methyloctanal (**3i**). Prepared according to the general procedure E using DCM as solvent instead of MeCN and without using $ZnCl_2$ as catalyst. 60% yield (0.026 g, 0.12 mmol on 0.2 mmol scale), *dr* >95/5, pale yellow oil. ^{1}H NMR (400 MHz, $CDCl_3$) δ 9.74 (dd, *J* = 2.8, 0.8 Hz, 1H), 3.70 (dd, *J* = 9.2, 4.0 Hz, 1H), 3.38 – 3.27 (m, 4H), 2.73 – 2.50 (m, 3H), 1.82 – 1.72 (m, 2H), 1.51 (s, 3H), 1.48 – 1.40 (m, 2H), 1.38 – 1.29 (m, 2H), 0.93 (t, *J* = 7.2 Hz, 3H).^{13}C NMR (100 MHz, $CDCl_3$) δ 201.0, 75.7, 72.8, 58.7, 44.1, 43.7, 42.2, 28.3, 26.6, 22.8, 14.0. HRMS (APCI) calcd. for $C_{11}H_{22}ClO_2$ [M+H]: 221.1303, found: 221.1309.

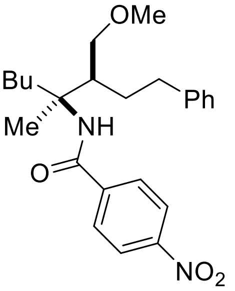

N-((3*S**,4*S**)-3-(methoxymethyl)-4-methyl-1-phenyloctan-4-yl)-4-nitrobenzamide (**7a**). Prepared according to the general procedure **F.** 45% yield, *dr* = 92/8 (the *dr* of crude azide is 92/8), white solid. ^{1}H NMR (400 MHz, $CDCl_3$) δ 8.28 – 8.21 (m, 2.0 H), 8.06 (s, 0.92H), 7.91 – 7.80 (m, 2H), 7.50 (s, 0.08H), 7.35 – 7.26 (m, 2H), 7.24-7.17 (m, 2H), 3.76 – 3.58 (m, 2H), 3.43 (s, 3H), 2.88-2.78 (m, 1H), 2.63-2.52 (m, 1H), 2.44 (td, *J* = 13.2, 4.0 Hz, 0.08H), 2.25 (td, *J* = 13.2, 4.4 Hz, 1H), 1.95 – 1.65 (m, 3H), 1.55 – 1.38 (m, 4H), 1.32 – 1.12 (m, 2H), 1.10 – 0.97 (m, 1H), 0.99 – 0.85 (m, 1.27H), 0.81 (t, *J* = 7.2 Hz, 2.73H).^{13}C NMR (100 MHz, $CDCl_3$) δ164.0, 149.1, 141.8, 141.4,

128.5, 128.4,127.7, 126.1, 123.5, 72.5, 60.2, 59.1, 42.4, 35.3, 34.2, 27.8, 25.7, 22.9, 20.4, 14.0. HRMS (ESI) calcd. for $C_{24}H_{32}O_4Na$ [M+Na]: 435.2260, found: 435.2259.

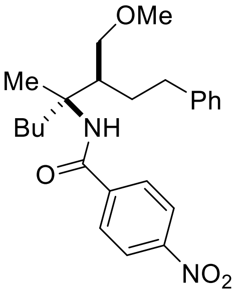

N-((3S*,4R*)-3-(methoxymethyl)-4-methyl-1-phenyloctan-4-yl)-4-nitrobenzamide (**7b**). Prepared according to the general procedure **F**. 53% yield, *dr* > 95/5 (the *dr* of crude azide is 92/8), pale yellow oil.^{1}H NMR (400 MHz, $CDCl_3$) δ 8.28 – 8.22 (m, 2H), 7.87 – 7.81 (m, 2H), 7.51 (s, 1H), 7.33 – 7.27 (m, 2H), 7.24 – 7.17 (m, 3H), 3.77 – 3.66 (m, 2H), 3.41 (s, 3H), 2.92 – 2.80 (m, 1H), 2.57 (dt, *J* = 13.6, 8.0 Hz, 1H), 2.44 (td, *J* = 13.6, 4.0 Hz, 1H), 1.89 – 1.68 (m, 4H), 1.51 (s, 3H), 1.36 – 1.13 (m, 3H), 1.12 – 1.00 (m, 1H), 0.88 (t, *J* = 7.2 Hz, 3H). ^{13}C NMR (100 MHz, $CDCl_3$) δ 164.4, 149.2, 141.82, 141.81, 128.5, 128.4, 127.7, 126.0, 123.6, 71.4, 60.2, 59.3, 44.0, 35.4, 34.0, 27.4, 26.3, 23.2, 23.0, 14.1. HRMS (ESI) calcd. for $C_{24}H_{32}N_2O_4Na$ [M+Na]: 435.2260, found: 435.2256.

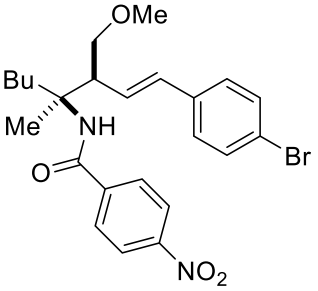

N-((3*S**,4*S**,*E*)-1-(4-bromophenyl)-3-(methoxymethyl)-4-methyloct-1-en-4-yl)-4-nitrobenzamide (**8c**). Prepared according to the general procedure **G** but reaction was stirred at -50 °C for 36 h. 40% yield, *dr* = 86/14 (the *dr* of crude azide is 88/12), white solid. ^{1}H NMR (400 MHz, $CDCl_3$) δ 8.29 – 8.22 (m, 2H), 7.94 – 7.81 (m, 3H), 7.47 – 7.38 (m, 2H), 7.25 – 7.16 (m, 2H), 6.48 (d, *J* = 15.6 Hz, 1H), 6.31 (dd, *J* = 16.0, 9.6 Hz, 0.14H), 6.12 (dd, *J* = 15.6, 9.6 Hz, 0.86H), 3.76 (dd, *J* = 9.6, 7.6 Hz, 1H), 3.71 – 3.62 (m, 1H), 3.42 (s, 2.61H), 3.38 (s, 0.41H), 2.97 – 2.85 (m, 1H), 2.35 – 2.11 (m, 1H), 1.86-1.73 (m, 1H), 1.53 (s, 3H), 1.38 – 1.20 (m, 4H), 0.92 – 0.84 (m, 3H). ^{13}C NMR (100 MHz, $CDCl_3$) δ 164.2, 149.2, 141.7, 135.7, 132.6, 131.7, 127.8, 127.7, 126.7, 123.7, 121.4, 74.3, 59.8, 59.3, 48.5, 36.2, 25.6, 22.9, 21.2, 14.1. HRMS (ESI) calcd. for $C_{24}H_{30}N_2O_4Br$ [M+H]: 489.1389, found: 489.1398.

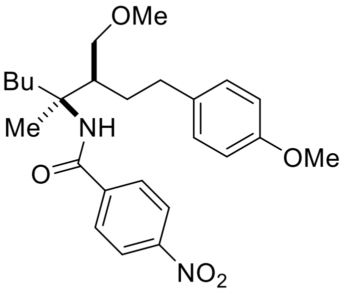

N-((3*S**,4*S**)-3-(methoxymethyl)-1-(4-methoxyphenyl)-4-methyloctan-4-yl)-4-nitrobenzamide (**7d**). Prepared according to the general procedure **F** but reaction was stirred at 0 °C for 12 h. 51% yield, *dr* > 95/5 (the *dr* of crude azide is 96/4), white solid. ^{1}H NMR (400 MHz, $CDCl_3$) δ 8.28 – 8.20 (m, 2H), 8.07 (s, 1H), 7.90 – 7.84 (m, 2H), 7.14 – 7.07 (m,2H), 6.90 – 6.80 (m, 2H), 3.78 (s, 3H), 3.70 – 3.56 (m, 2H), 3.42 (s, 3H), 2.82-2.73 (m, 1H), 2.51 (dt, *J* = 13.6, 8.0 Hz, 1H), 2.30 – 2.19 (m, 1H), 1.92-1.83 (m, 1H), 1.74 – 1.59 (m, 2H), 1.49 – 1.37 (m, 4H), 1.27 – 1.10 (m, 2H), 1.06 – 0.85 (m, 2H), 0.80 (t, *J* = 7.2 Hz, 3H). ^{13}C NMR (100 MHz, $CDCl_3$) δ 164.0, 158.0, 149.2, 141.8, 133.5, 129.4, 127.7, 123.6, 113.9, 72.6, 60.2, 59.1, 55.2, 42.3, 35.3, 33.3, 28.0, 25.7, 22.9, 20.3, 14.0. HRMS (ESI) calcd. for $C_{25}H_{34}N_2O_5Na$ [M+Na]: 465.2365, found: 465.2362.

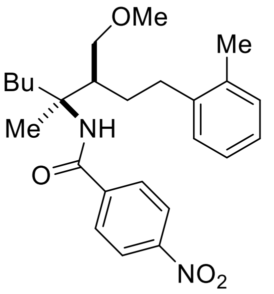

N-((3*S**,4*S**)-3-(methoxymethyl)-4-methyl-1-(o-tolyl)octan-4-yl)-4-nitrobenzamide (**7e**). Prepared according to the general procedure **F**. 50% yield, *dr* = 94/6 (the *dr* of crude azide is 95/5), white solid. ^{1}H NMR (400 MHz, $CDCl_3$) δ 8.30 – 8.21 (m, 2H), 8.05 (s, 1H), 7.92 – 7.82 (m, 2H), 7.19 – 7.08 (m, 4H), 3.75 – 3.62 (m, 2H), 3.45 (s, 3H), 2.90-2.75 (m, 1H), 2.65-2.55 (m, 1H), 2.38 – 2.23 (m, 4H), 1.97 – 1.88 (m, 1H), 1.78 – 1.63 (m, 2H), 1.49 – 1.35 (m, 4H), 1.29 – 1.16 (m, 2H), 1.16 – 0.95 (m, 2H), 0.83 (t, *J* = 7.2 Hz, 3H). ^{13}C NMR (100 MHz, $CDCl_3$) δ 164.0, 149.2, 141.8, 139.7, 135.6, 130.4, 129.0, 127.7, 126.3, 126.1, 123.6, 72.6, 60.3, 59.1, 43.0, 35.4, 31.8, 26.7, 25.7, 22.9, 20.4, 19.2, 14.1. HRMS (ESI) calcd. for $C_{25}H_{35}N_2O_4$ [M+H]: 427.2597, found: 427.2618.

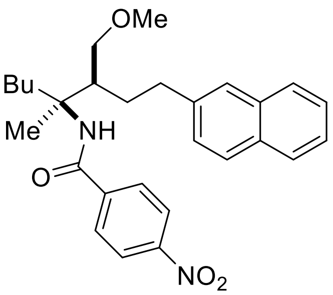

N-((*3S**,*4S**)-3-(methoxymethyl)-4-methyl-1-(naphthalen-2-yl)octan-4-yl)-4-nitrobenzamide. (**7f**). Prepared according to the general procedure **F**. 54% yield, *dr* > 95/5 (the *dr* of crude azide is 96/4), pale yellow oil. ^{1}H NMR (400 MHz, $CDCl_3$) δ 8.28 – 8.19 (m, 2H), 8.06 (s, 1H), 7.90 – 7.84 (m, 2H), 7.83-7.76 (m, 3H), 7.64 (s, 1H), 7.52 – 7.39 (m, 2H), 7.34 (dd, *J* = 8.4, 1.6 Hz, 1H), 3.74 – 3.65 (m, 2H), 3.44 (s, 3H), 3.05 – 2.95 (m, 1H), 2.80 – 2.70 (m, 1H), 2.29-2.20 (m, 1H), 1.98 – 1.79 (m, 2H), 1.72 – 1.53 (m, 2H), 1.47 (s,

3H), 1.18 – 0.82 (m, 4H), 0.67 (t, J = 7.2 Hz, 3H). ^{13}C NMR (100 MHz, $CDCl_3$) δ 164.0, 149.1, 141.7, 138.9, 133.5, 132.1, 128.1, 127.7, 127.6, 127.3, 127.0, 126.6, 126.0, 125.3, 123.5, 72.5, 60.2, 59.1, 42.4, 35.4, 34.3, 27.7, 25.7, 22.9, 20.4, 13.9. HRMS (ESI) calcd. for $C_{28}H_{34}N_2O_4Na$ [M+Na]: 485.2416, found: 485.2396.

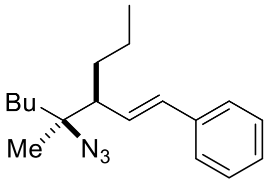

((3S*,4S*,E*)-4-azido-4-methyl-3-propyloct-1-en-1-yl)benzene (**6g**). Prepared according to the general procedure **F** without transforming to the corresponding amide. 75% yield, *dr* > 95/5, colorless oil.^{1}H NMR (400 MHz, $CDCl_3$) δ 7.42-7.30 (m, 4H), 7.26 – 7.20 (m, 1H), 6.40 (d, J = 15.6 Hz, 1H), 5.95 (dd, J = 15.6, 9.6 Hz, 1H), 2.26 – 2.17 (m, 1H), 1.69 – 1.53 (m, 3H), 1.46 – 1.28 (m, 6H), 1.25 (s, 3H), 1.22 – 1.10 (m, 1H), 0.96-0.88 (m, 6H). ^{13}C NMR: (100 MHz,) delta 137.3, 132.7, 129.9, 128.5, 127.3, 126.1, 66.6, 51.5, 38.4, 31.2, 25.7, 23.0, 20.9, 20.6, 14.1, 14.0. HRMS (ESI) calcd. for $C_{18}H_{27}N$ [M-N_2]: 257.2143, found: 257.2150.

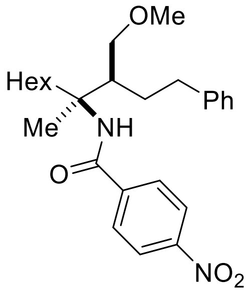

N-((3*S**,4*S**)-3-(methoxymethyl)-4-methyl-1-phenyldecan-4-yl)-4-nitrobenzamide (**7h**). Prepared according to the general procedure **F**. 46% yield, *dr* > 95/5 (the *dr* of crude azide is 93/7), colorless oil. ^{1}H NMR (400 MHz, $CDCl_3$) δ 8.28 – 8.21 (m, 2H), 8.05 (s, 1H), 7.90 – 7.85 (m, 2H), 7.35 – 7.25 (m, 2H), 7.24 – 7.17 (m, 3H), 3.73-3.58 (m, 2H), 3.43 (s, 3H), 2.88-2.78 (m, 1H), 2.58 (dt, J = 13.6, 8.0 Hz, 1H), 2.31 – 2.20 (m, 1H), 1.94 – 1.84 (m, 1H), 1.80 – 1.60 (m, 2H), 1.54 – 1.40 (m, 4H), 1.29 – 1.12 (m, 6H), 1.11 – 0.91 (m, 2H), 0.85 (t, J = 7.2 Hz, 3H). ^{13}C NMR (100 MHz, $CDCl_3$) δ 164.0, 149.1, 141.8, 141.5, 128.5, 128.4, 127.7, 126.1, 123.5, 72.5, 60.3, 59.1, 42.5, 35.6, 34.3, 31.7, 29.5, 27.8, 23.5, 22.6, 20.4, 14.0. HRMS (ESI) calcd. for $C_{26}H_{36}N_2O_4Na$ [M+Na]: 463.2573, found: 463.2573.

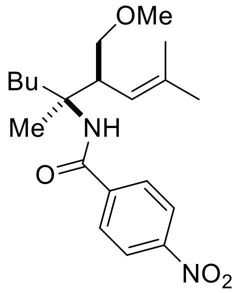

N-((4S*,5S*)-4-(methoxymethyl)-2,5-dimethylnon-2-en-5-yl)-4-nitrobenzamide (**8i**). Prepared according to the general procedure **G** but reaction was stirred at 0 °C for 12 h. 41% yield, *dr* > 95/5 (the *dr* of crude azide is 95/5), pale yellow oil. ^{1}H NMR (400 MHz, $CDCl_3$) δ 8.29 – 8.21 (m, 2H), 8.05 (s, 1H), 7.95 – 7.88 (m, 2H), 5.02 (dt, *J* = 10.4, 1.2 Hz, 1H), 3.60 (dd, *J* = 9.6, 8.4 Hz, 1H), 3.45 (dd, *J* = 9.6, 3.2 Hz, 1H), 3.38 (s, 3H), 2.96 – 2.88 (m, 1H), 2.31 – 2.21 (m, 1H), 1.75 (d, *J* = 1.2 Hz, 3H), 1.70 (d, *J* = 1.2 Hz, 3H), 1.66 – 1.56 (m, 1H), 1.46 (s, 3H), 1.32 – 1.15 (m, 4H), 0.85 (t, *J* = 7.2 Hz, 3H).^{13}C NMR (100 MHz, $CDCl_3$) δ 164.0, 149.2, 141.9, 135.4, 127.8, 123.6, 120.0, 74.6, 60.1, 59.2, 43.3, 36.1, 26.1, 25.8, 23.0, 20.4, 18.3, 14.1. HRMS (ESI) calcd. for $C_{20}H_{31}N_2O_4$ [M+H]: 363.2284, found: 363.2284.

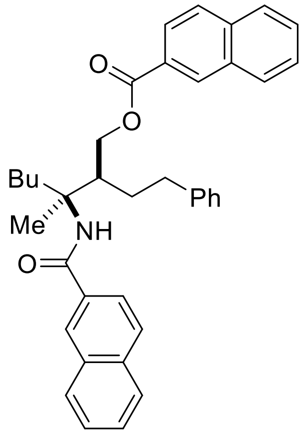

(2S*,3S*)-3-(2-naphthamido)-3-methyl-2-phenethylheptyl 2-naphthoate (**7j**). Prepared according to the general procedure **F** but reaction was stirred at -5 °C for 12 h. 40% yield, *dr* > 95/5, colorless oil. ^{1}H NMR (400 MHz, $CDCl_3$) δ 8.56 (s, 1H), 8.28 (s, 1H), 8.01 (dd, *J* = 8.8, 1.6 Hz, 1H), 7.87 – 7.73 (m, 7H), 7.63 – 7.55 (m, 1H), 7.54 – 7.40 (m, 3H), 7.30 – 7.16 (m, 5H), 6.78 (s, 1H), 4.87 (dd, *J* = 12.0, 4.0 Hz, 1H), 4.52 (dd, *J* = 12.0, 4.4 Hz, 1H), 3.07 – 2.96 (m, 1H), 2.80-2.70 (m, 1H), 2.67 – 2.61 (m, 1H), 2.14 – 1.91 (m, 3H), 1.87 – 1.72 (m, 1H), 1.52 (s, 3H), 1.39 – 1.26 (m, 3H), 1.20-1.13 (m, 1H), 0.87 (t, *J* = 7.2 Hz, 3H). ^{13}C NMR: (100 MHz,) delta 167.1, 167.0, 142.1, 135.6, 134.5, 132.8, 132.6, 132.4, 131.2, 129.3, 128.8, 128.5, 128.43, 128.39, 128.3, 127.7, 127.6, 127.4, 127.1, 126.7, 126.5, 126.0, 125.0, 123.7, 66.1, 59.5, 43.8, 35.7, 34.6, 30.4, 25.7, 23.0, 21.2, 14.1. HRMS (ESI) calcd. for $C_{38}H_{39}NO_3Na$ [M+Na]: 580.2828, found: 580.2828.

Crystal structure for **2c**. (CCDC: 2128937)

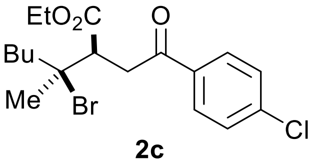

2c

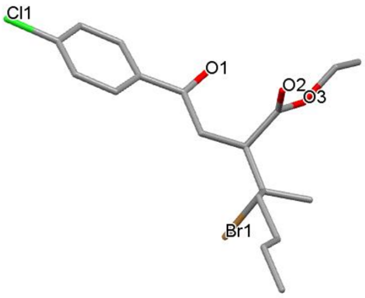

Crystal structure for **2d** (CCDC: 2128934)

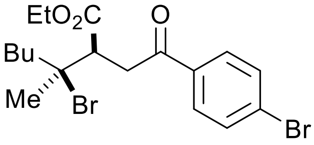

2d

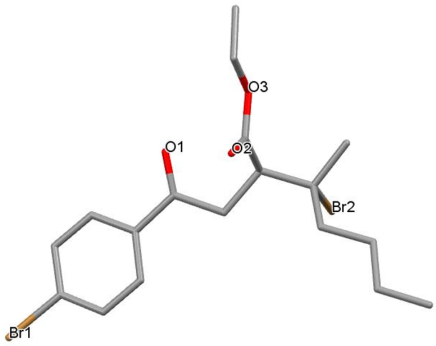

Crystal structure for **4b** (CCDC: 2128939)

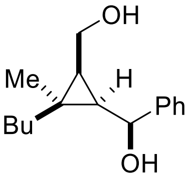

4b

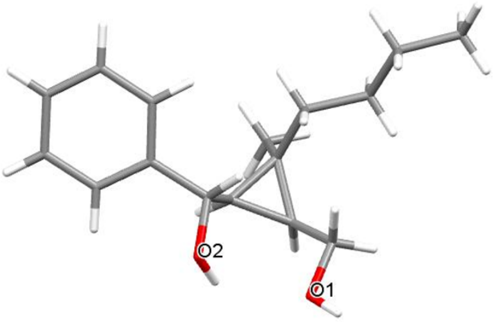

Crystal structure for **7a** (CCDC: 2128938)

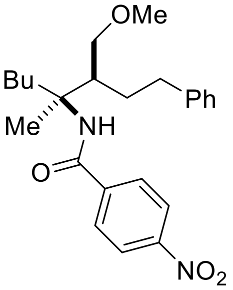

7a

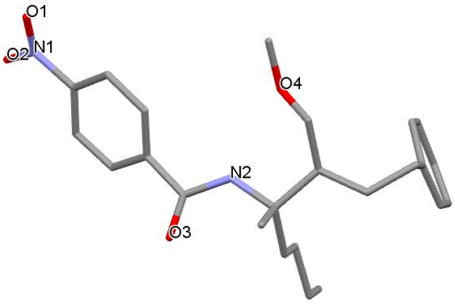

V. References

[1] a) H. Sommer, I. Marek, *Chem. Sci.* **2018**, *9*, 6503-6508; b) F. G. Zhang, I. Marek, *J. Am. Chem. Soc.* **2017**, *139*, 8364-8370.

[2] a) D. Didier, P. O. Delaye, M. Simaan, B. Island, G. Eppe, H. Eijsberg, A. Kleiner, P. Knochel, I. Marek, *Chem. Eur. J.* **2014**, *20*, 1038-1048; b) Y. Cohen, I. Marek, *Org. Lett.* **2019**, *21*, 9162-9165.

[3] S. Singh, J. Bruffaerts, A. Vasseur, I. Marek, *Nat. Commun.* **2017**, *8*, 14200.

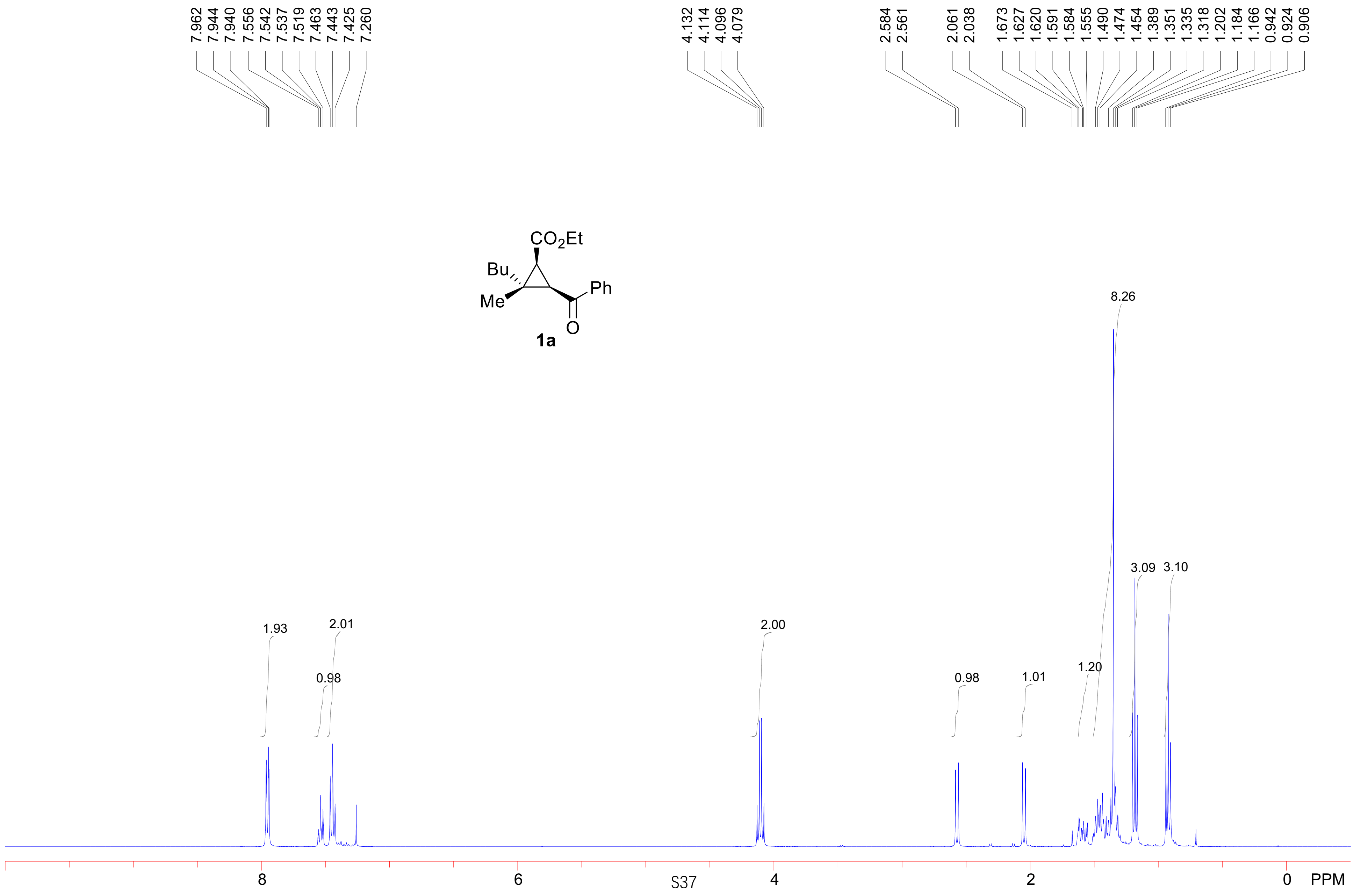
7.962 7.944 7.940 7.556 7.542 7.537 7.519 7.463 7.443 7.425 7.260
4.132 4.114 4.096 4.079
2.584 2.561
2.061 2.038
1.673 1.627 1.620 1.591 1.584 1.555 1.490 1.474 1.454 1.389 1.351 1.335 1.318 1.202 1.184 1.166 0.942 0.924 0.906
CO_2Et
Bu
Me
Ph
O
1a
1.93
0.98
2.01
2.00
0.98
1.01
1.20
8.26
3.09
3.10
8
6
4
2
0
PPM

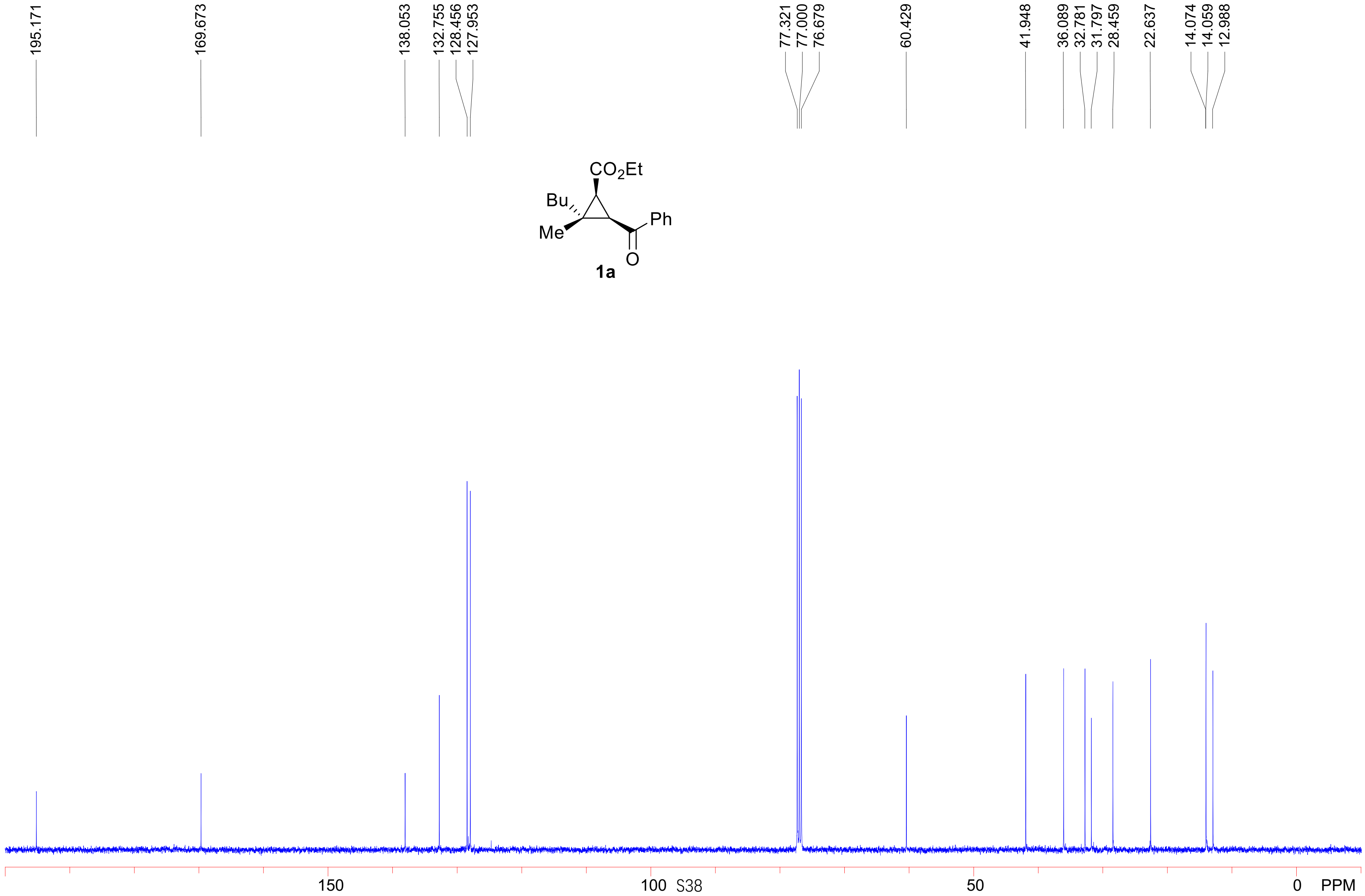
195.171
169.673
138.053
132.755
128.456
127.953
77.321
77.000
76.679
60.429
41.948
36.089
32.781
31.797
28.459
22.637
14.074
14.059
12.988
CO2Et
Bu
Me
Ph
O
1a
150
100
50
0
PPM

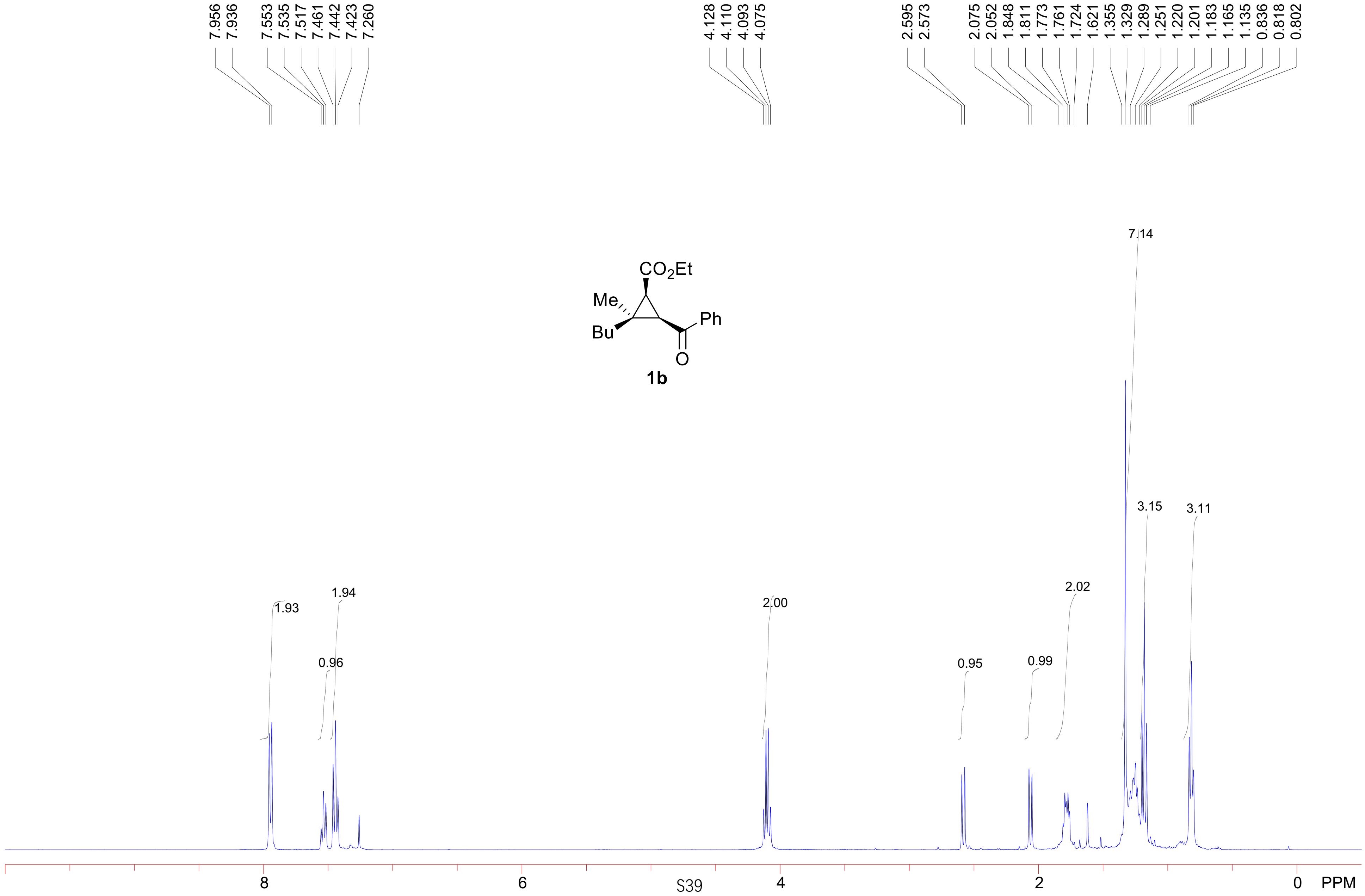
7.956
7.936
7.553
7.535
7.517
7.461
7.442
7.423
7.260
4.128
4.110
4.093
4.075
2.595
2.573
2.075
2.052
1.848
1.811
1.773
1.761
1.724
1.621
1.355
1.329
1.289
1.251
1.220
1.201
1.183
1.165
1.135
0.836
0.818
0.802
CO2Et
Me
Bu
Ph
O
1b
7.14
3.15
3.11
2.02
2.00
1.93
1.94
0.96
0.95
0.99
8
6
4
2
0
PPM

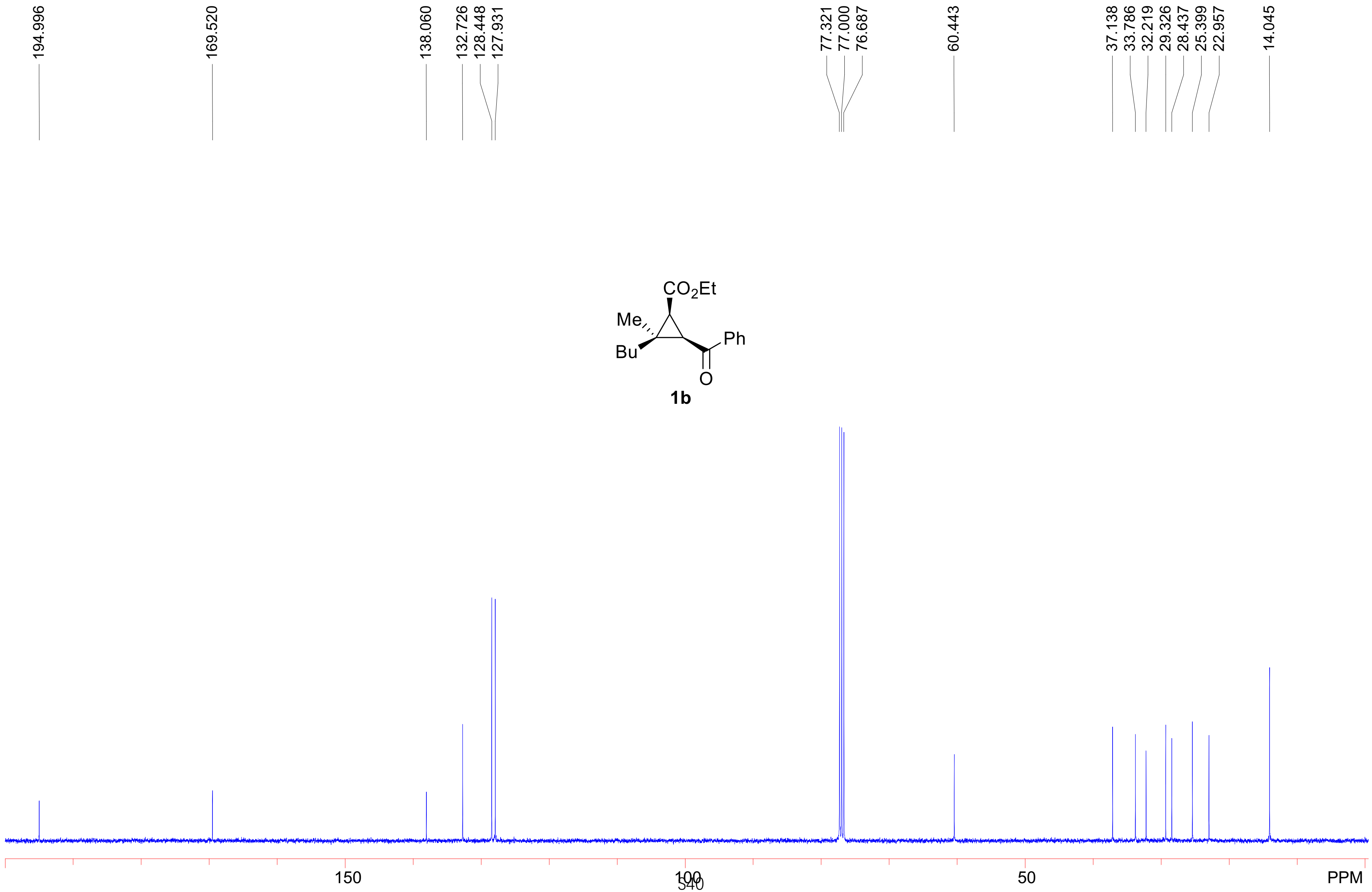
194.996
169.520
138.060
132.726
128.448
127.931
77.321
77.000
76.687
60.443
37.138
33.786
32.219
29.326
28.437
25.399
22.957
14.045
CO2Et
Me
Bu
Ph
O
1b
150
100
50
PPM

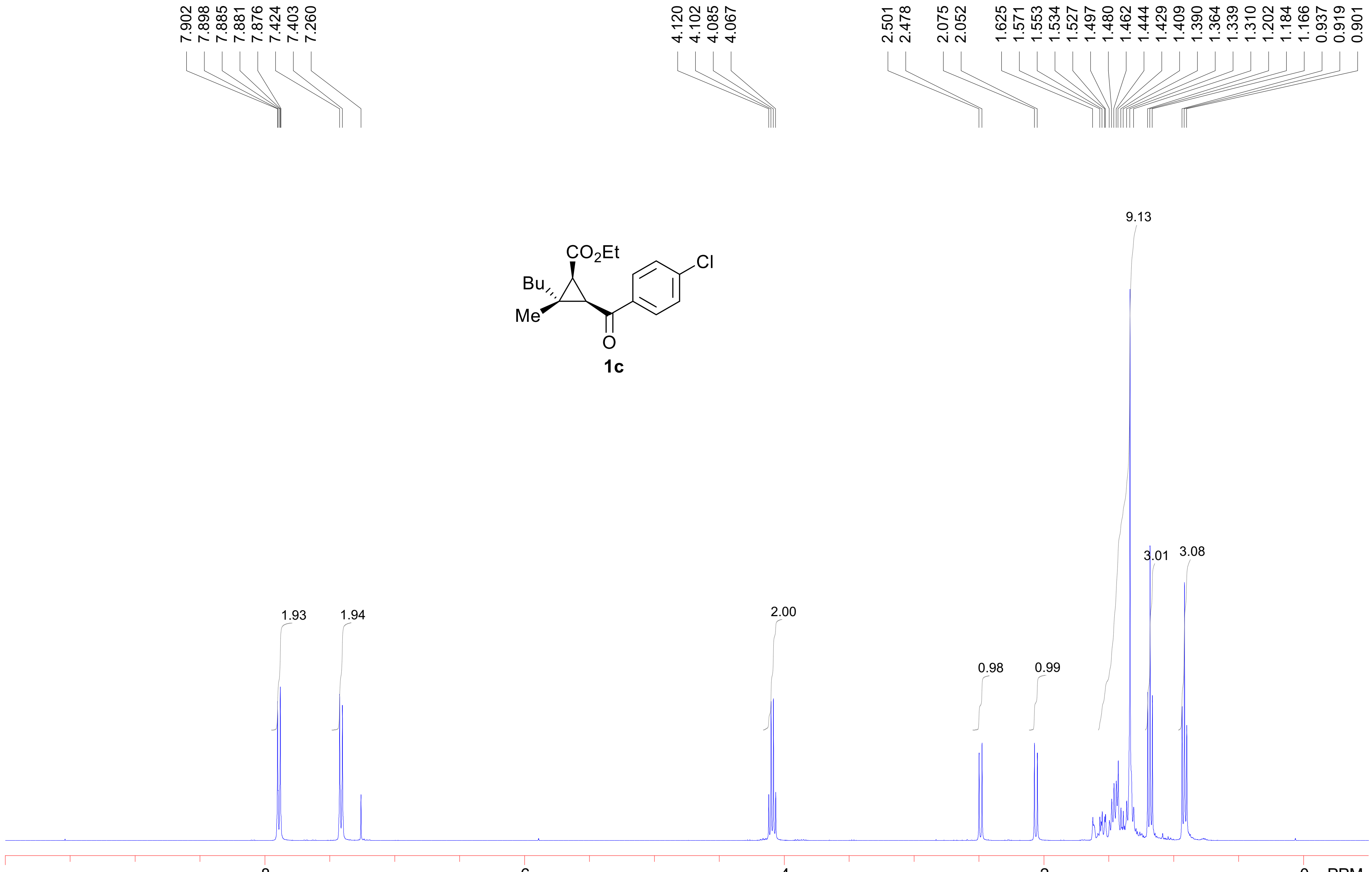
7.902
7.898
7.885
7.881
7.876
7.424
7.403
7.260
4.120
4.102
4.085
4.067
2.501
2.478
2.075
2.052
1.625
1.571
1.553
1.534
1.527
1.497
1.480
1.462
1.444
1.429
1.409
1.390
1.364
1.339
1.310
1.202
1.184
1.166
0.937
0.919
0.901
CO_2Et
Bu
Me
Cl
O
1c
9.13
3.01
3.08
1.93
1.94
2.00
0.98
0.99
8
6
4
2
0
PPM

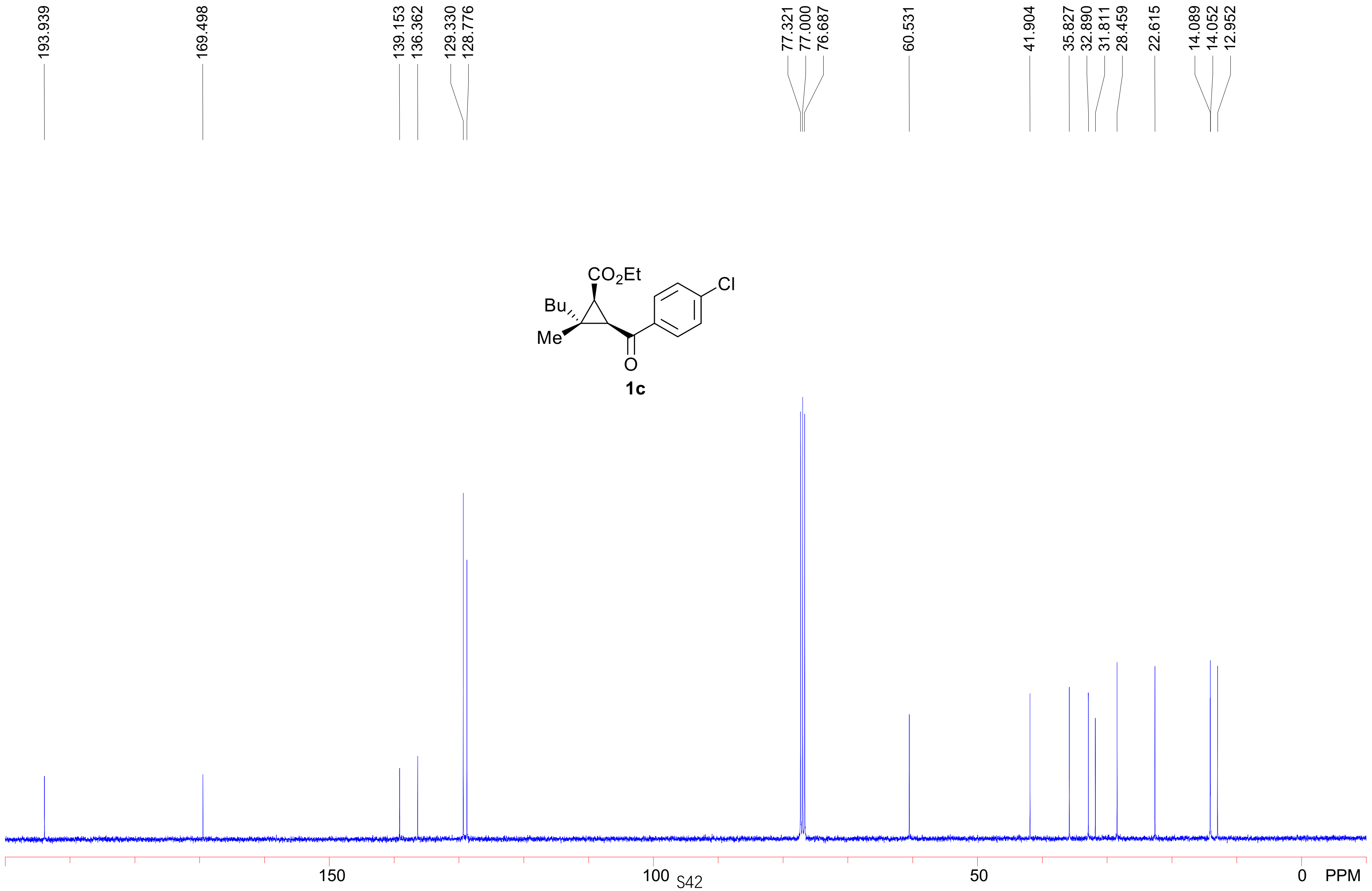
193.939
169.498
139.153
136.362
129.330
128.776
77.321
77.000
76.687
60.531
41.904
35.827
32.890
31.811
28.459
22.615
14.089
14.052
12.952
CO2Et
Bu
Me
O
Cl
1c
150
100
50
0
PPM

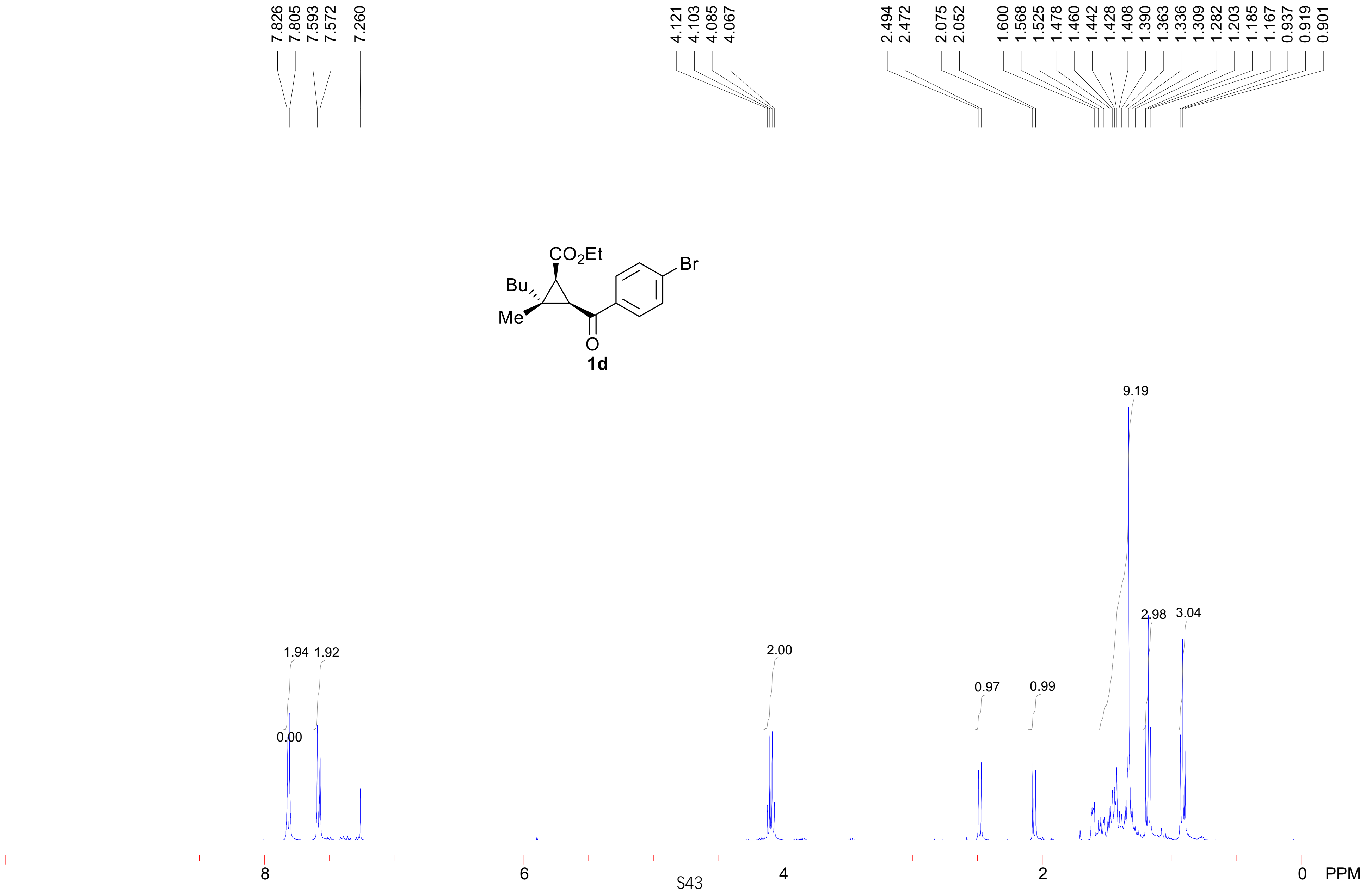
7.826
7.805
7.593
7.572
7.260
4.121
4.103
4.085
4.067
2.494
2.472
2.075
2.052
1.600
1.568
1.525
1.478
1.460
1.442
1.428
1.408
1.390
1.363
1.336
1.309
1.282
1.203
1.185
1.167
0.937
0.919
0.901
CO2Et
Br
Bu
Me
O
1d
9.19
2.98
3.04
1.94
1.92
0.00
2.00
0.97
0.99
8
6
4
2
0
PPM

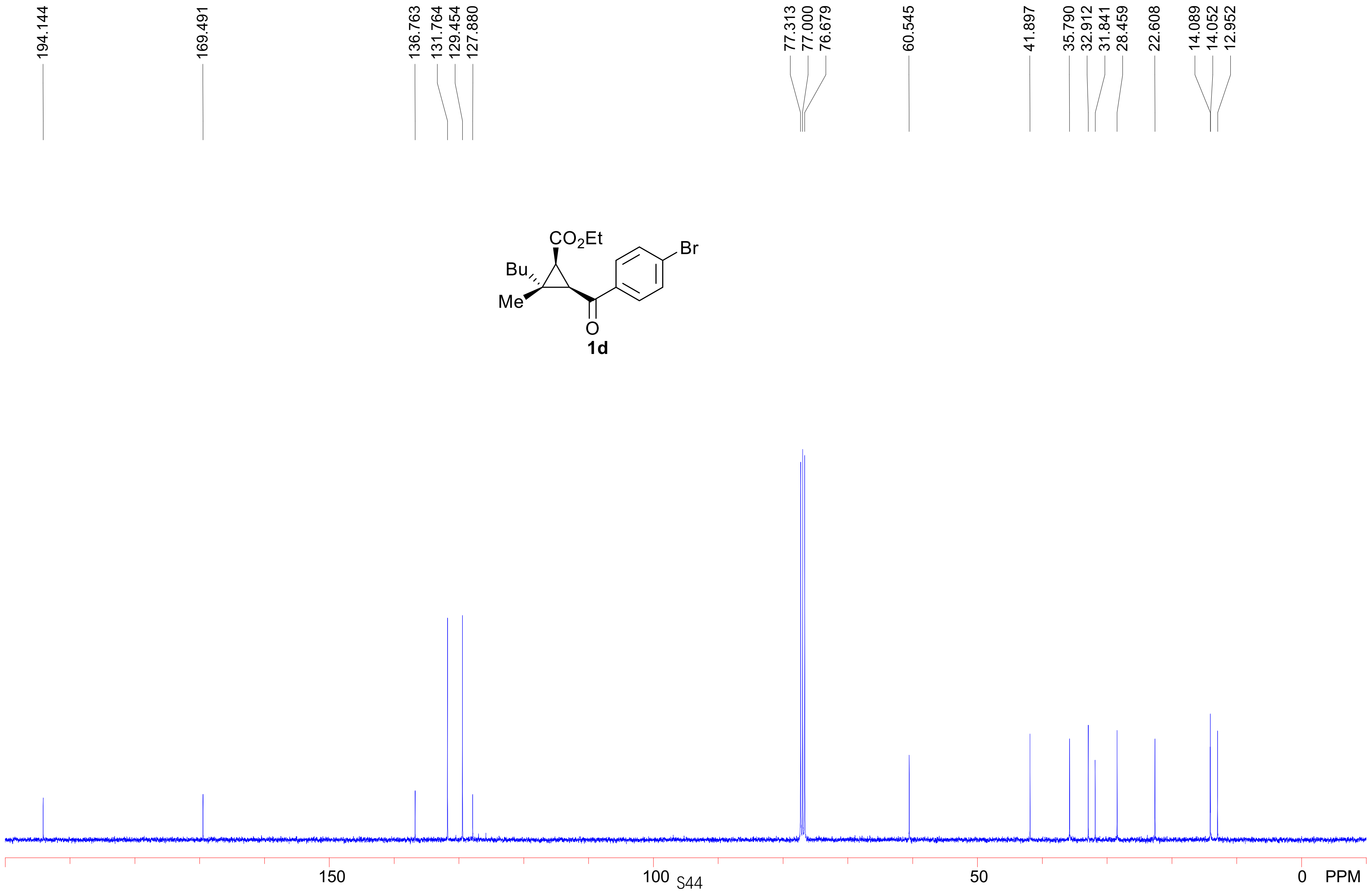
194.144
169.491
136.763
131.764
129.454
127.880
77.313
77.000
76.679
60.545
41.897
35.790
32.912
31.841
28.459
22.608
14.089
14.052
12.952
CO_2Et
Br
Bu
Me
O
1d
150
100
50
0
PPM

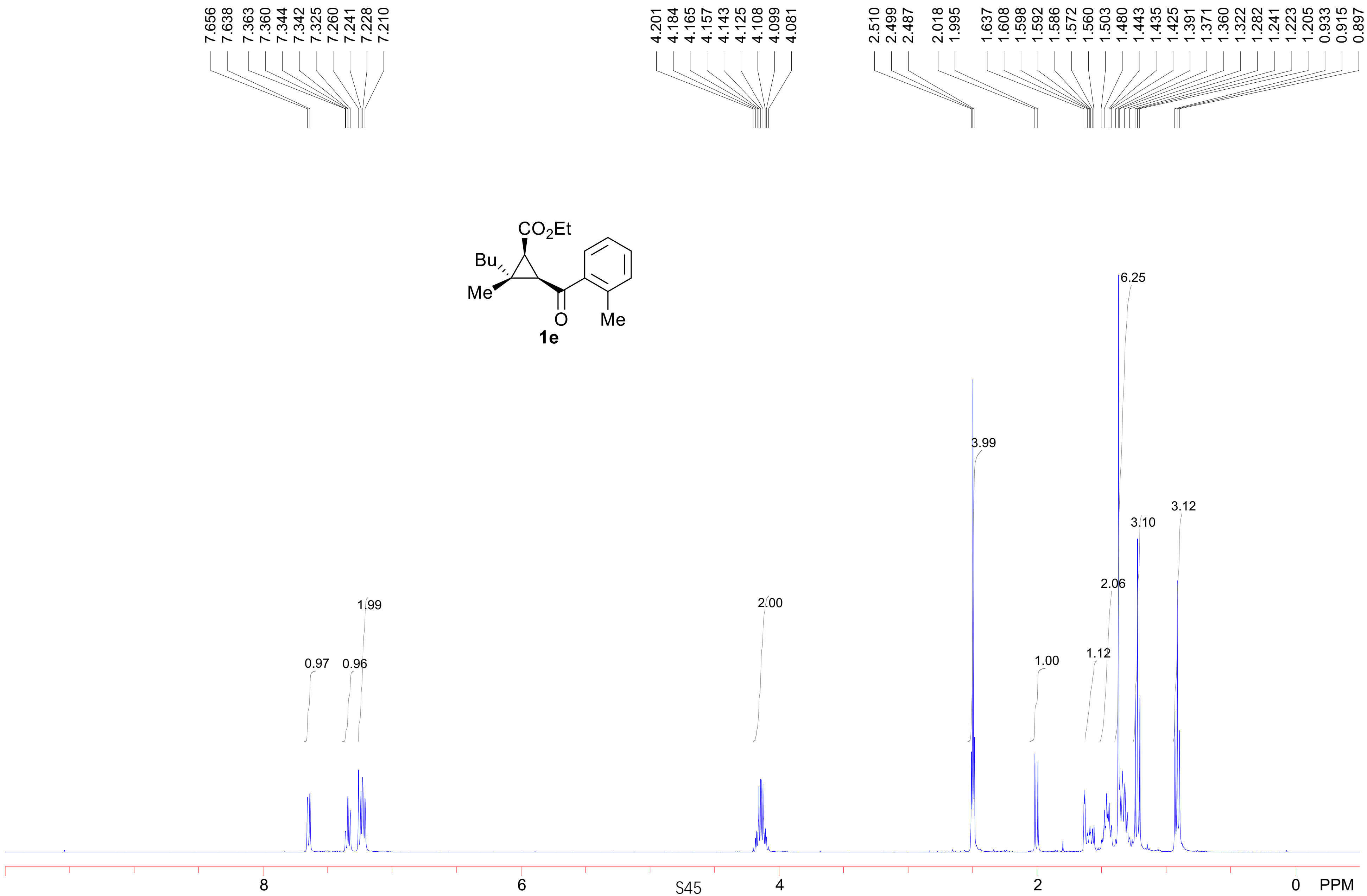
7.656
7.638
7.363
7.360
7.344
7.342
7.325
7.260
7.241
7.228
7.210
4.201
4.184
4.165
4.157
4.143
4.125
4.108
4.099
4.081
2.510
2.499
2.487
2.018
1.995
1.637
1.608
1.598
1.592
1.586
1.572
1.560
1.503
1.480
1.443
1.435
1.425
1.391
1.371
1.360
1.322
1.282
1.241
1.223
1.205
0.933
0.915
0.897
CO2Et
Bu
Me
O
Me
1e
6.25
3.99
3.12
3.10
2.06
1.99
2.00
1.12
1.00
0.97
0.96
8
6
4
2
0
PPM

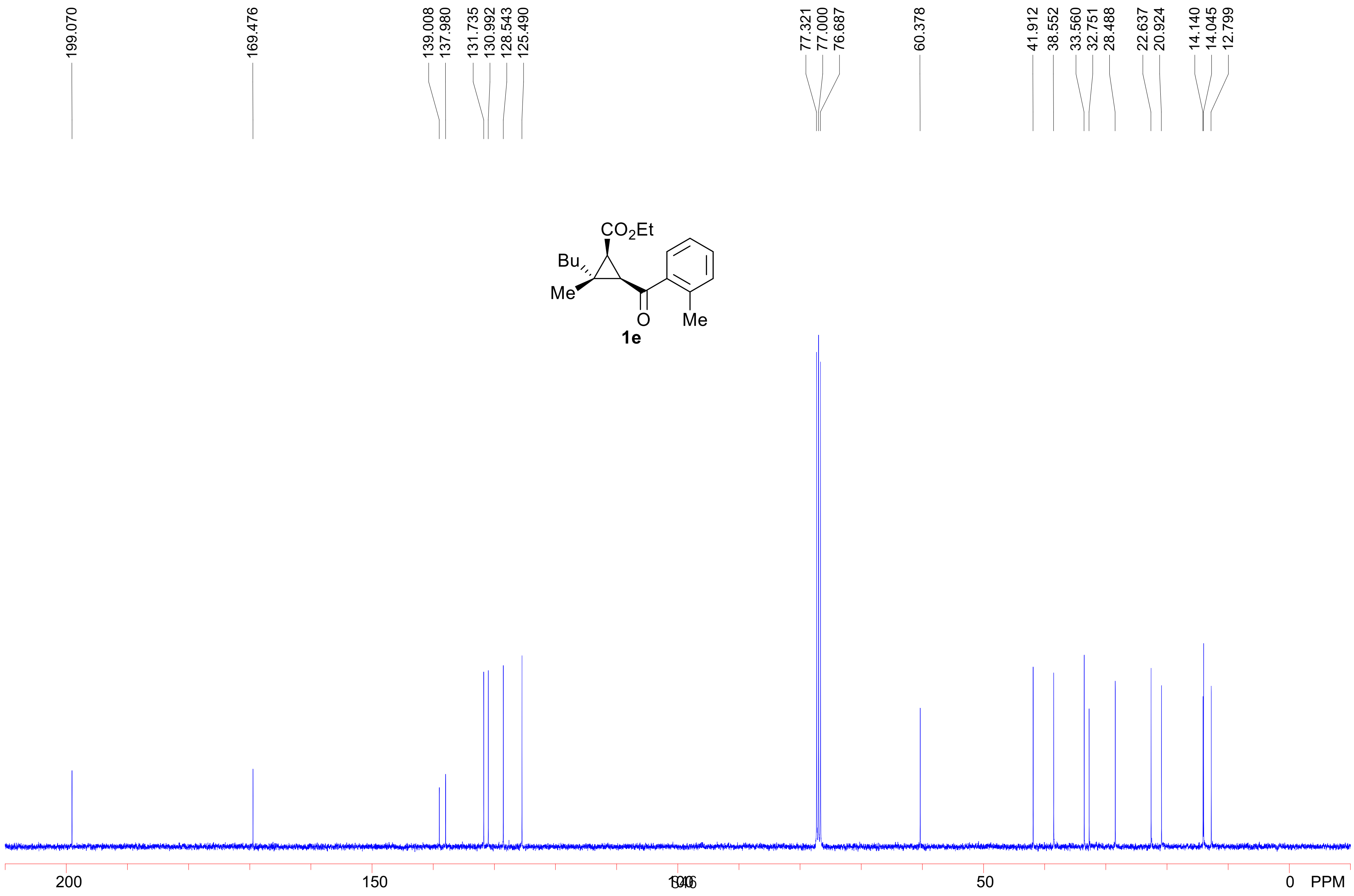
199.070
169.476
139.008
137.980
131.735
130.992
128.543
125.490
77.321
77.000
76.687
60.378
41.912
38.552
33.560
32.751
28.488
22.637
20.924
14.140
14.045
12.799
CO2Et
Bu
Me
O
Me
1e
200
150
50
0
PPM

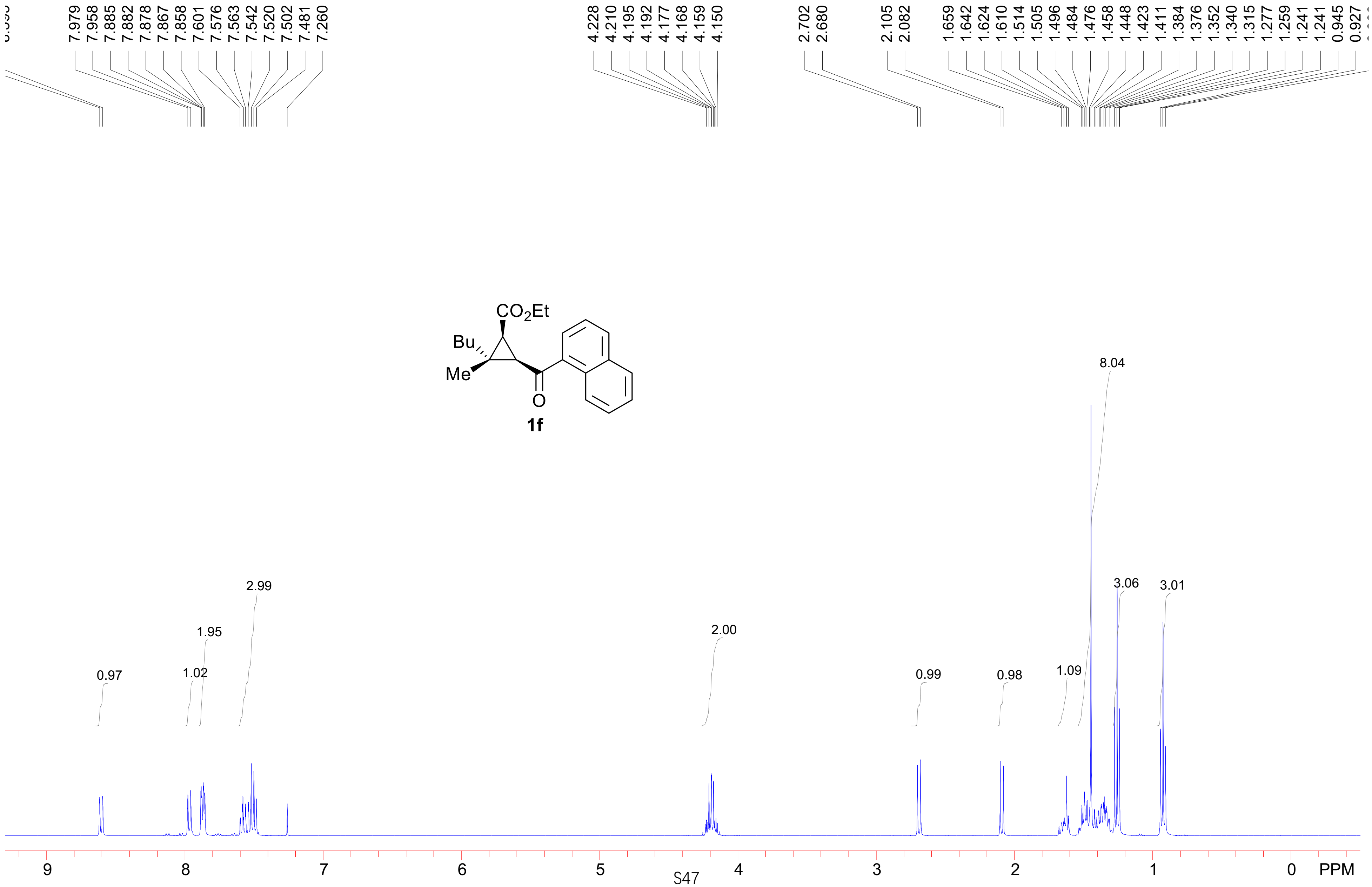
7.979
7.958
7.885
7.882
7.878
7.867
7.858
7.601
7.576
7.563
7.542
7.520
7.502
7.481
7.260
4.228
4.210
4.195
4.192
4.177
4.168
4.159
4.150
2.702
2.680
2.105
2.082
1.659
1.642
1.624
1.610
1.514
1.505
1.496
1.484
1.476
1.458
1.448
1.423
1.411
1.384
1.376
1.352
1.340
1.315
1.277
1.259
1.241
1.241
0.945
0.927
CO2Et
Bu
Me
O
1f
8.04
2.99
1.95
1.02
0.97
2.00
0.99
0.98
1.09
3.06
3.01
9
8
7
6
5
4
3
2
1
0
PPM

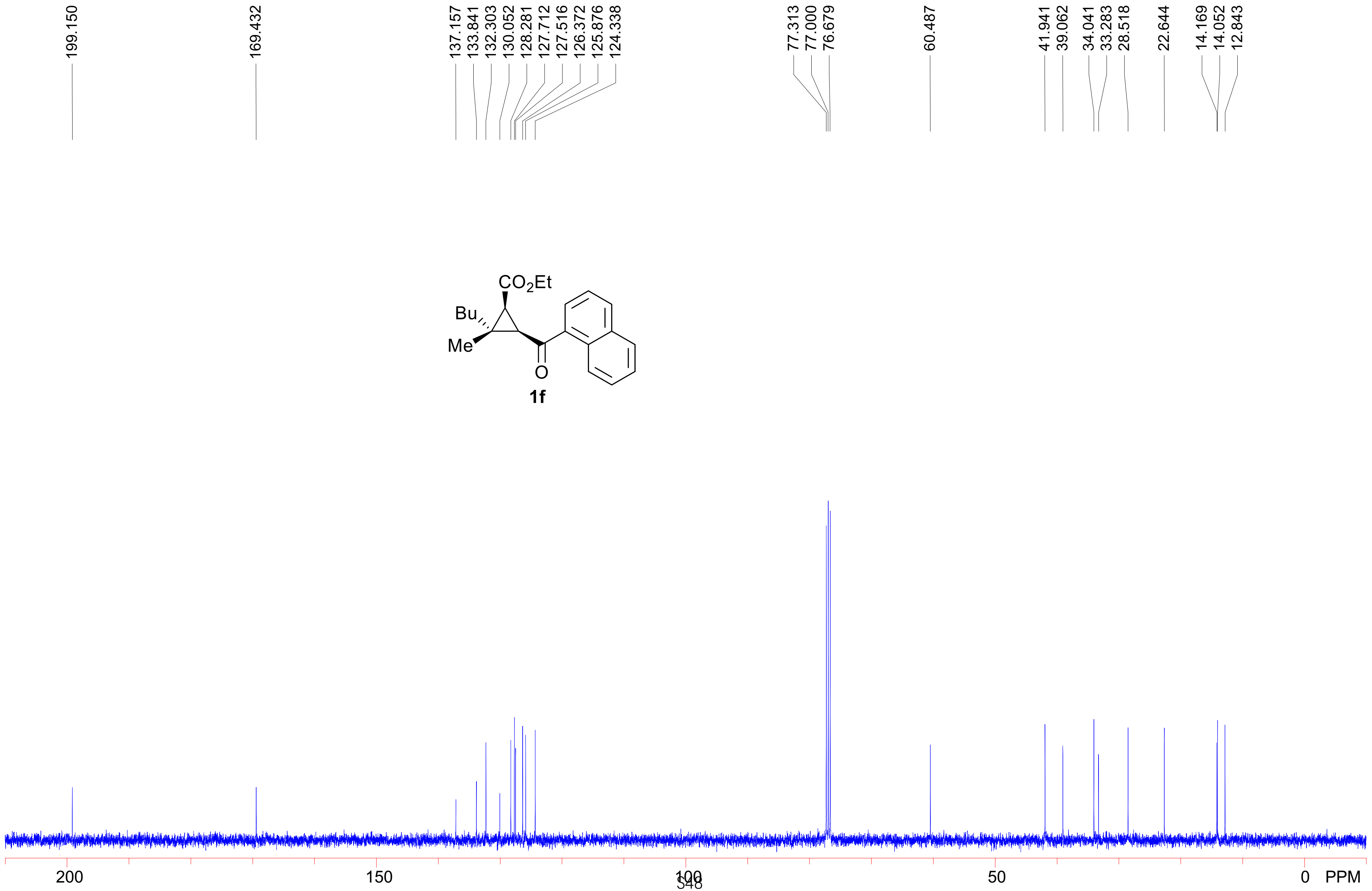
199.150
169.432
137.157
133.841
132.303
130.052
128.281
127.712
127.516
126.372
125.876
124.338
77.313
77.000
76.679
60.487
41.941
39.062
34.041
33.283
28.518
22.644
14.169
14.052
12.843
CO2Et
Bu
Me
O
1f
200
150
100
50
0
PPM

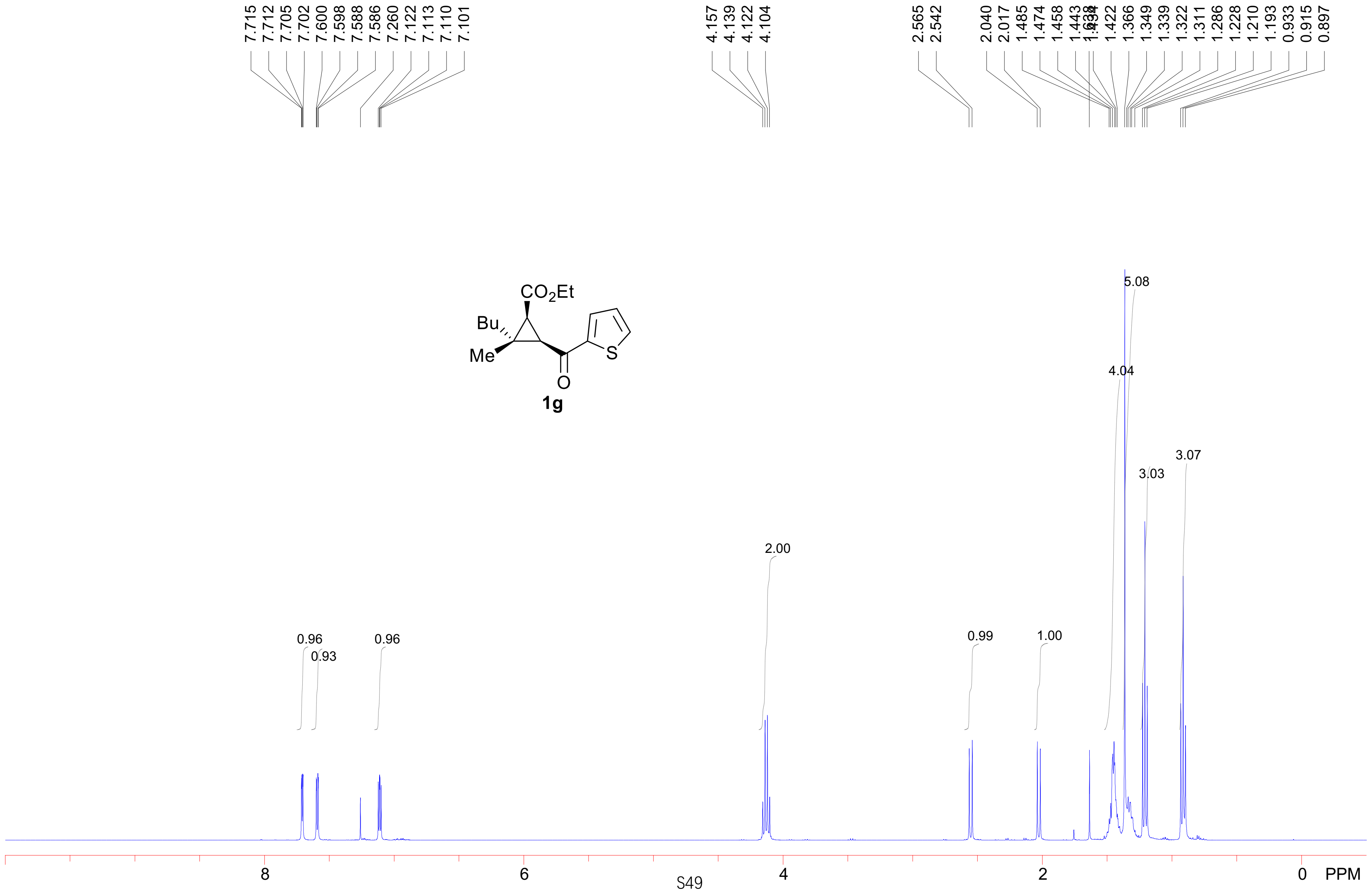
7.715
7.712
7.705
7.702
7.600
7.598
7.588
7.586
7.260
7.122
7.113
7.110
7.101
4.157
4.139
4.122
4.104
2.565
2.542
2.040
2.017
1.485
1.474
1.458
1.443
1.422
1.366
1.349
1.339
1.322
1.311
1.286
1.228
1.210
1.193
0.933
0.915
0.897
CO_2Et
Bu
Me
S
O
1g
5.08
4.04
3.07
3.03
2.00
0.96
0.93
0.96
0.99
1.00
8
6
4
2
0
PPM

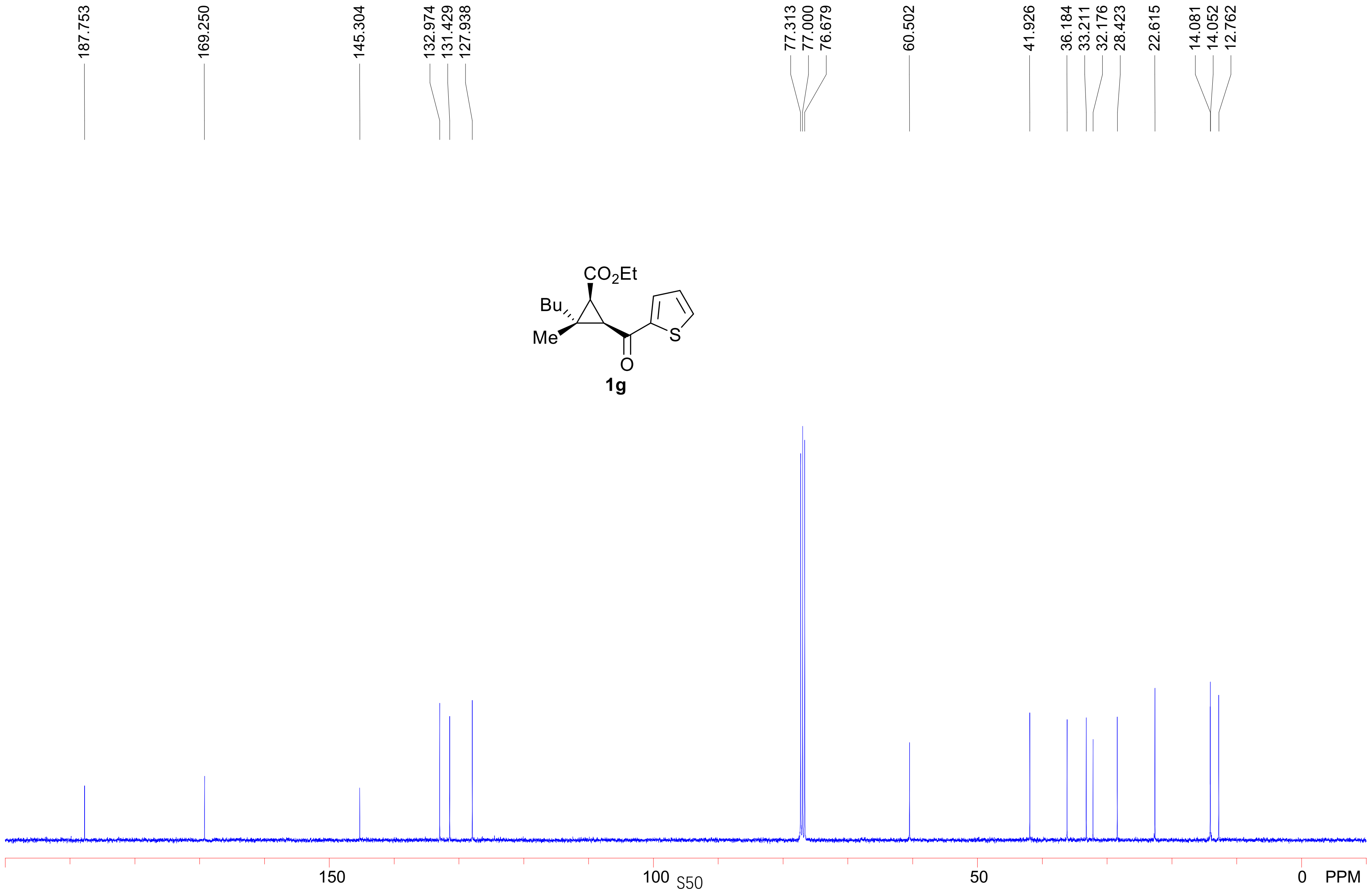
187.753
169.250
145.304
132.974
131.429
127.938
77.313
77.000
76.679
60.502
41.926
36.184
33.211
32.176
28.423
22.615
14.081
14.052
12.762
CO_2Et
Bu
Me
O
S
1g
150
100
50
0
PPM

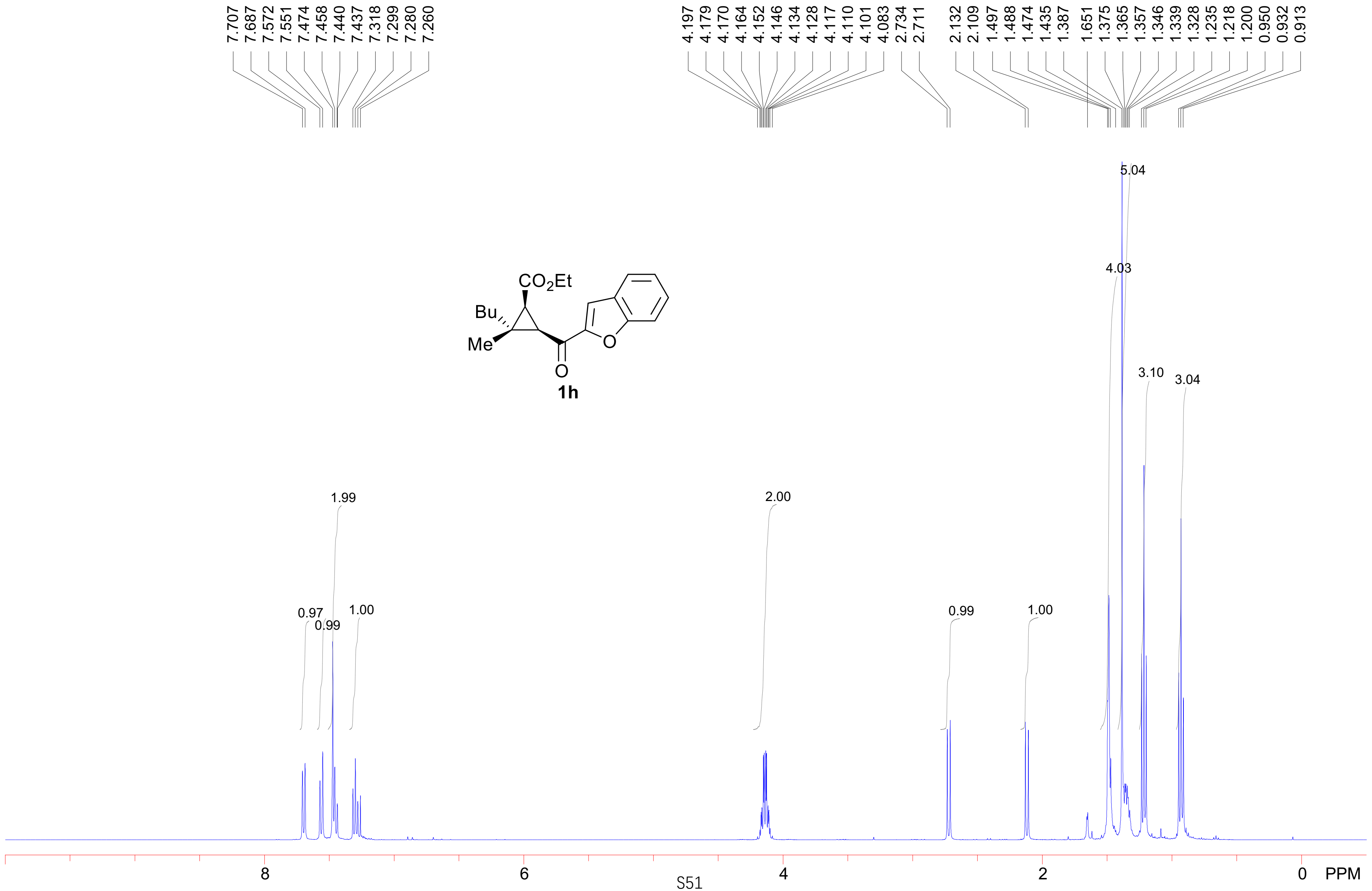
7.707 7.687 7.572 7.551 7.474 7.458 7.440 7.437 7.318 7.299 7.280 7.260
4.197 4.179 4.170 4.164 4.152 4.146 4.134 4.128 4.117 4.110 4.101 4.083 2.734 2.711
2.132 2.109 1.497 1.488 1.474 1.435 1.387 1.651 1.375 1.365 1.357 1.346 1.339 1.328 1.235 1.218 1.200 0.950 0.932 0.913
CO2Et
Bu
Me
O
O
1h
5.04
4.03
3.10
3.04
1.99
2.00
0.97
0.99
1.00
0.99
1.00
8
6
4
2
0
PPM

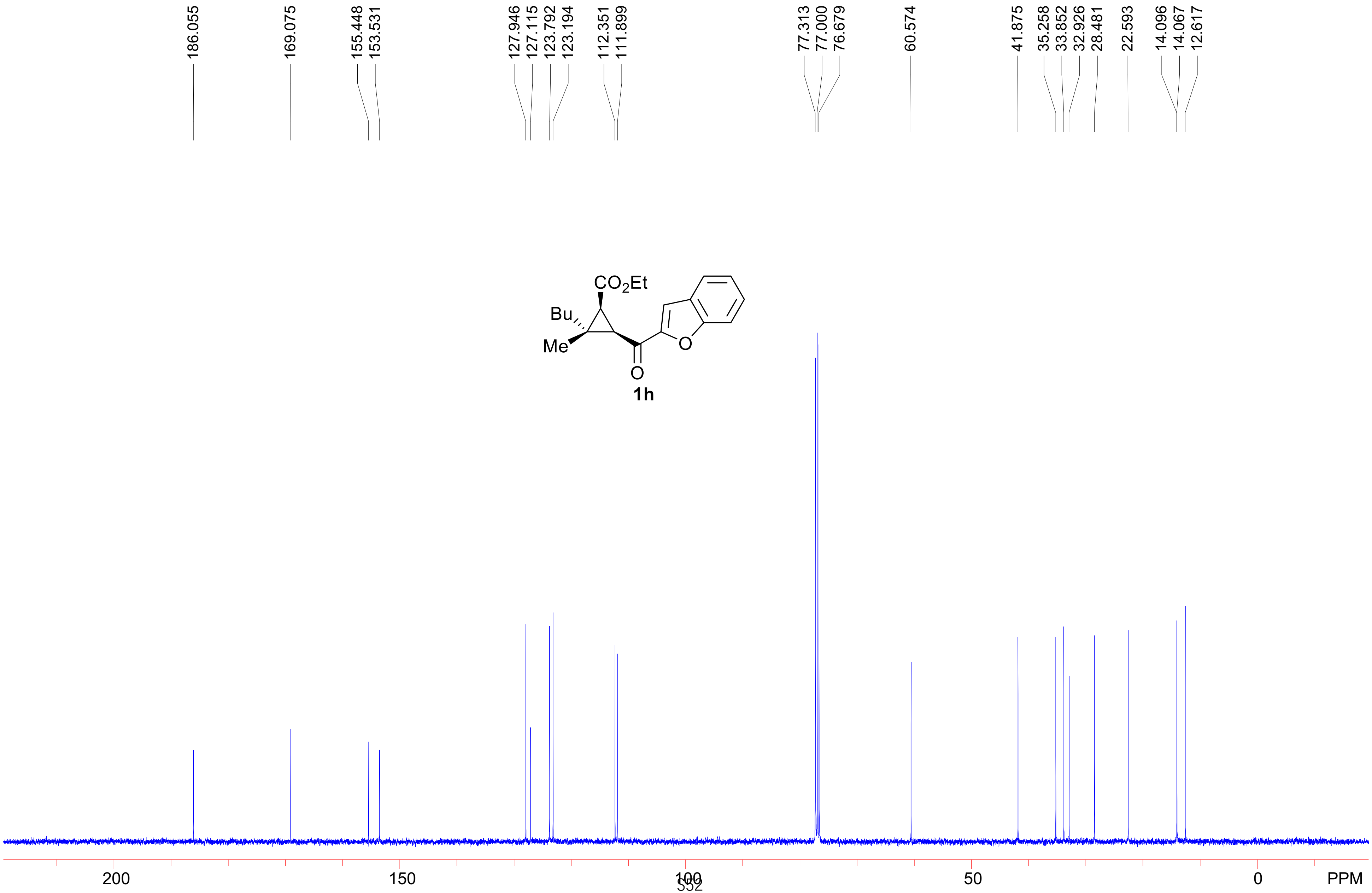
186.055
169.075
155.448
153.531
127.946
127.115
123.792
123.194
112.351
111.899
77.313
77.000
76.679
60.574
41.875
35.258
33.852
32.926
28.481
22.593
14.096
14.067
12.617
CO2Et
Bu
Me
O
O
1h
200
150
100
50
0
PPM

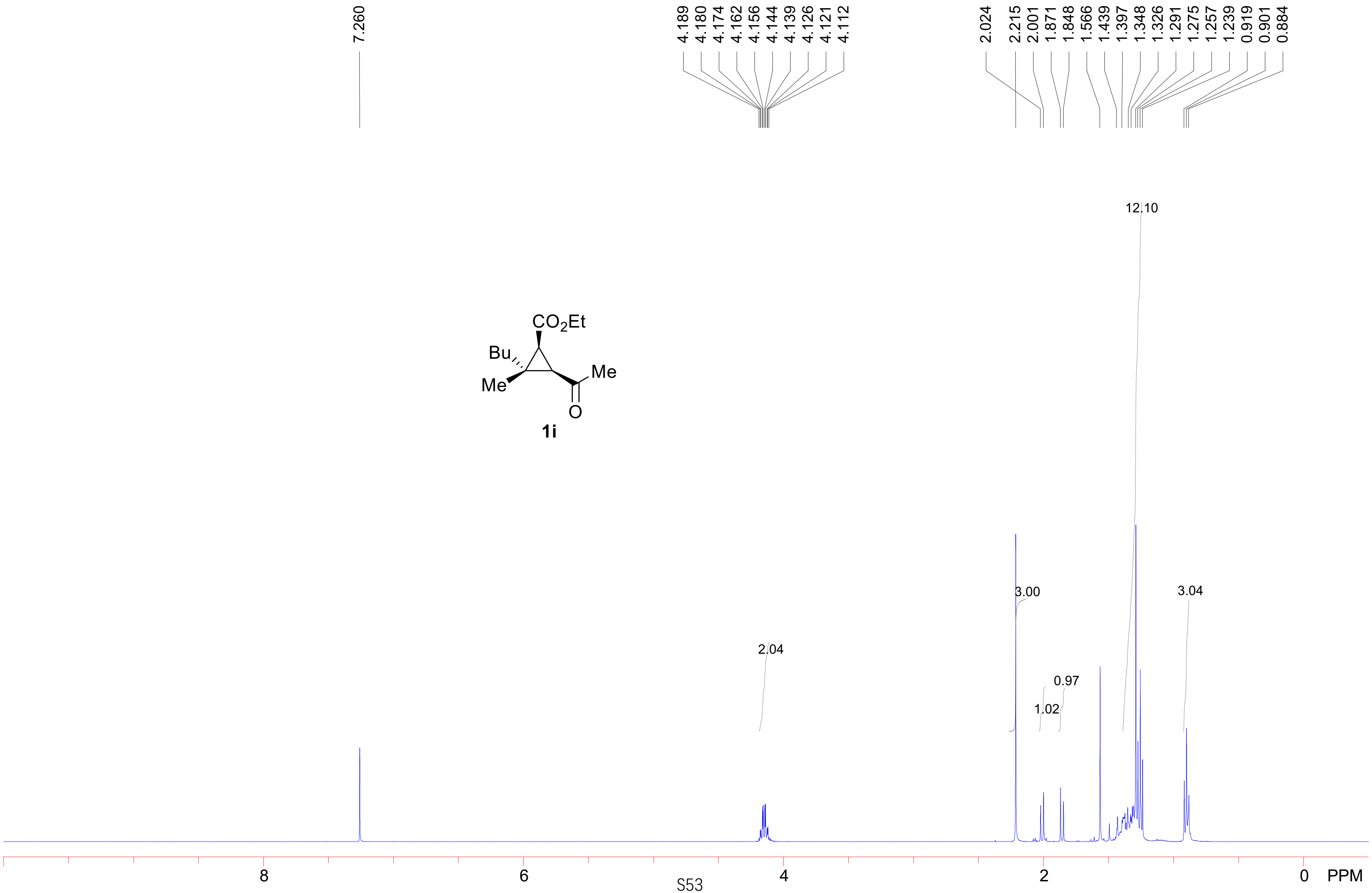

7.260
4.189
4.180
4.174
4.162
4.156
4.144
4.139
4.126
4.121
4.112
2.024
2.215
2.001
1.871
1.848
1.566
1.439
1.397
1.348
1.326
1.291
1.275
1.257
1.239
0.919
0.901
0.884
12.10
1i
3.00
3.04
2.04
0.97
1.02
8
6
4
2
0
PPM

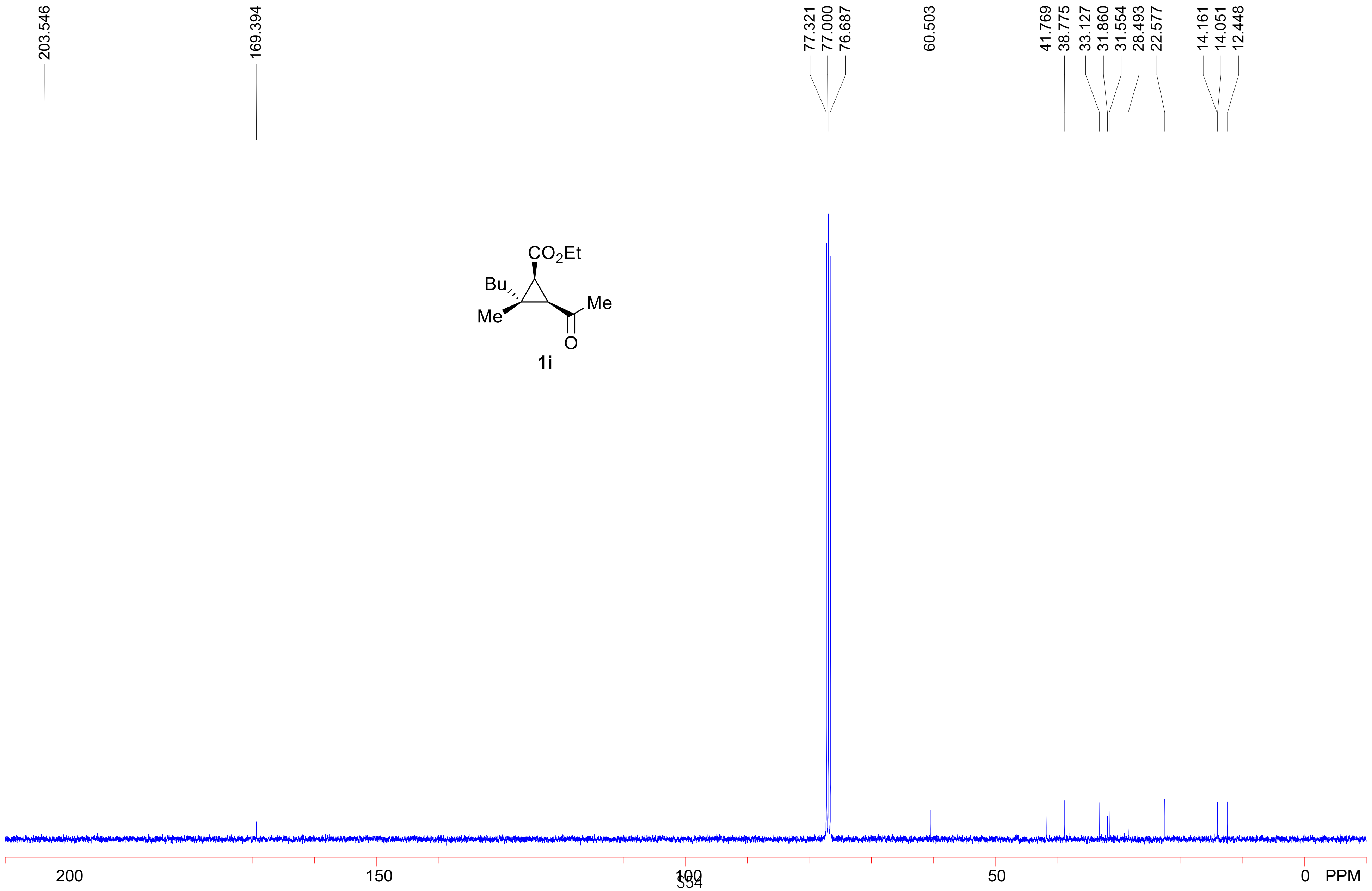
203.546
169.394
77.321
77.000
76.687
60.503
41.769
38.775
33.127
31.860
31.554
28.493
22.577
14.161
14.051
12.448
CO2Et
Bu
Me
Me
O
1i
200
150
100
50
0
PPM

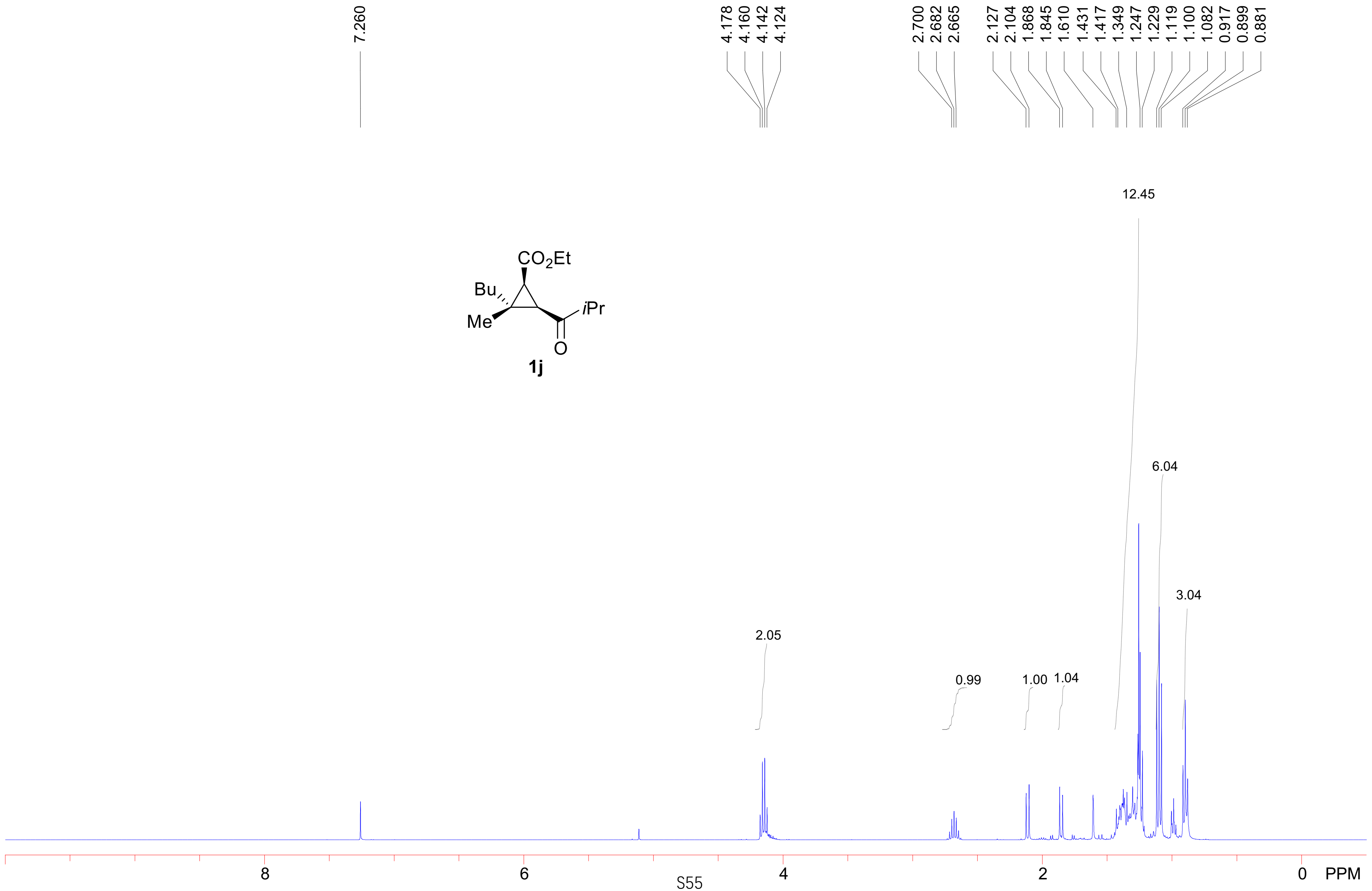
7.260
4.178
4.160
4.142
4.124
2.700
2.682
2.665
2.127
2.104
1.868
1.845
1.610
1.431
1.417
1.349
1.247
1.229
1.119
1.100
1.082
0.917
0.899
0.881
12.45
6.04
3.04
2.05
0.99
1.00
1.04
CO2Et
Bu
Me
iPr
O
1j
8
6
4
2
0
PPM

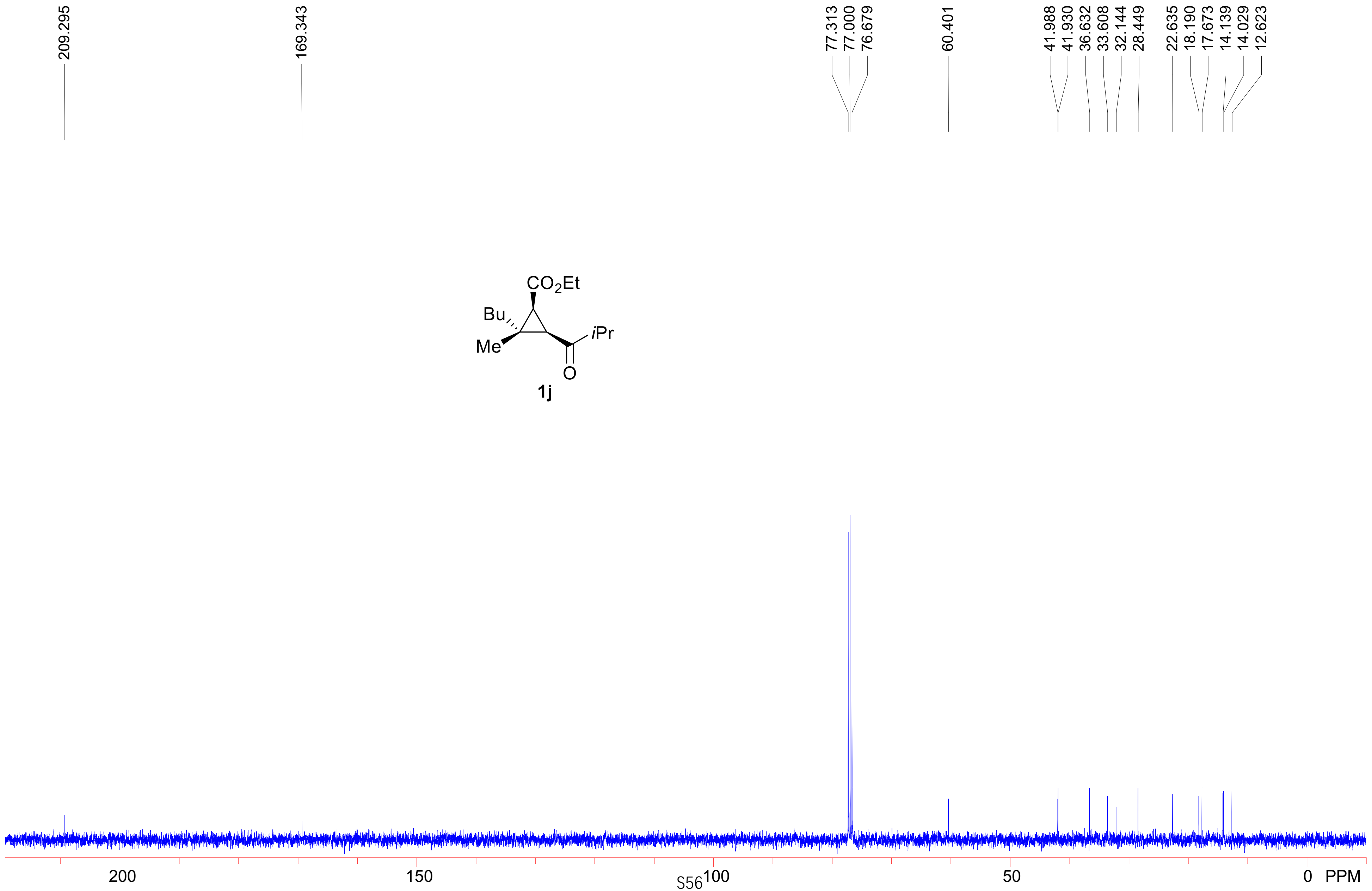
209.295
169.343
77.313
77.000
76.679
60.401
41.988
41.930
36.632
33.608
32.144
28.449
22.635
18.190
17.673
14.139
14.029
12.623
CO2Et
Bu
Me
iPr
O
1j
200
150
100
50
0 PPM

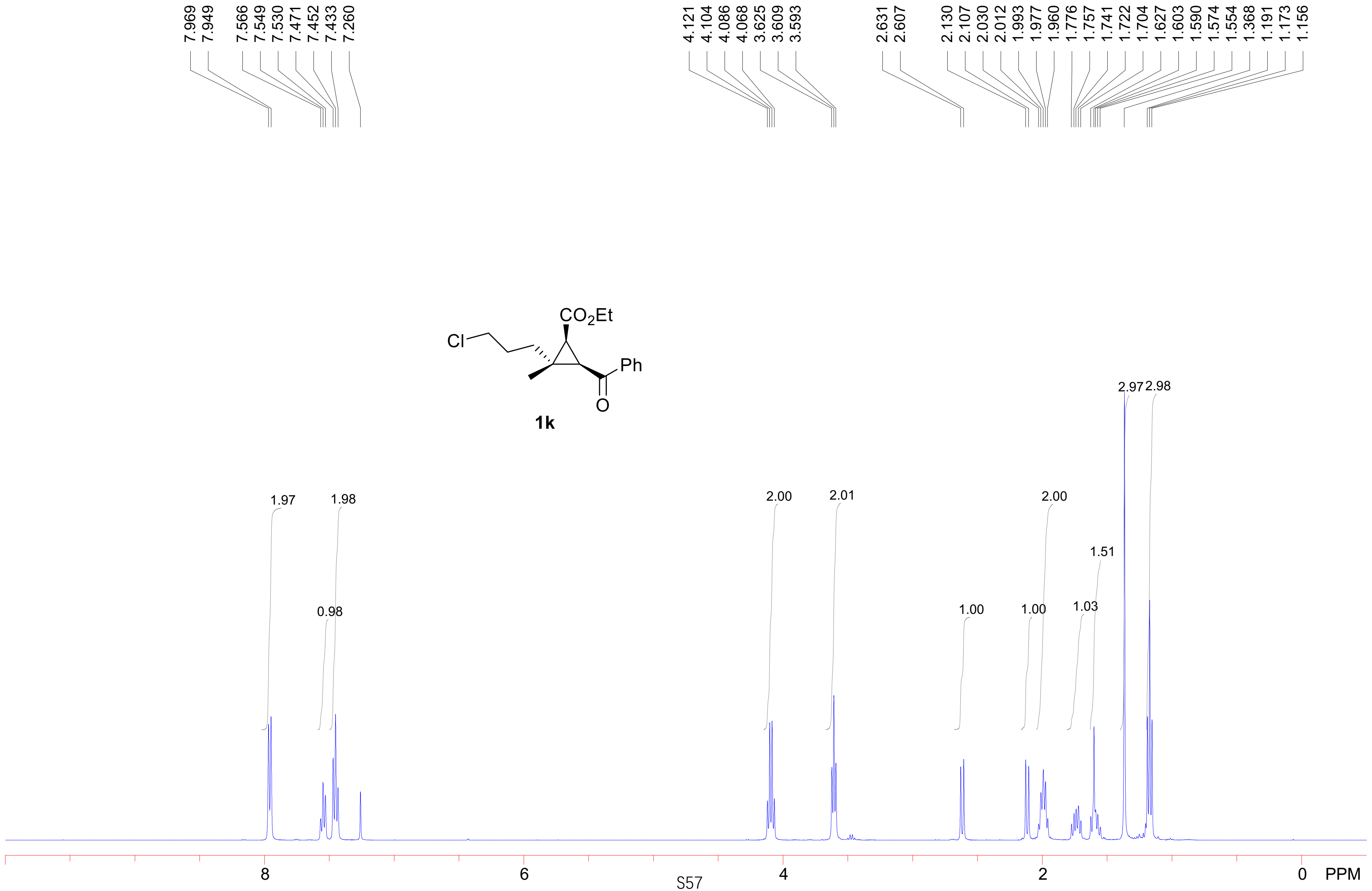
7.969
7.949
7.566
7.549
7.530
7.471
7.452
7.433
7.260
4.121
4.104
4.086
4.068
3.625
3.609
3.593
2.631
2.607
2.130
2.107
2.030
2.012
1.993
1.977
1.960
1.776
1.757
1.741
1.722
1.704
1.627
1.603
1.590
1.574
1.554
1.368
1.191
1.173
1.156
CO_2Et
Cl
Ph
O
1k
1.97
0.98
1.98
2.00
2.01
1.00
1.00
2.00
1.51
1.03
2.97
2.98
8
6
4
2
0
PPM

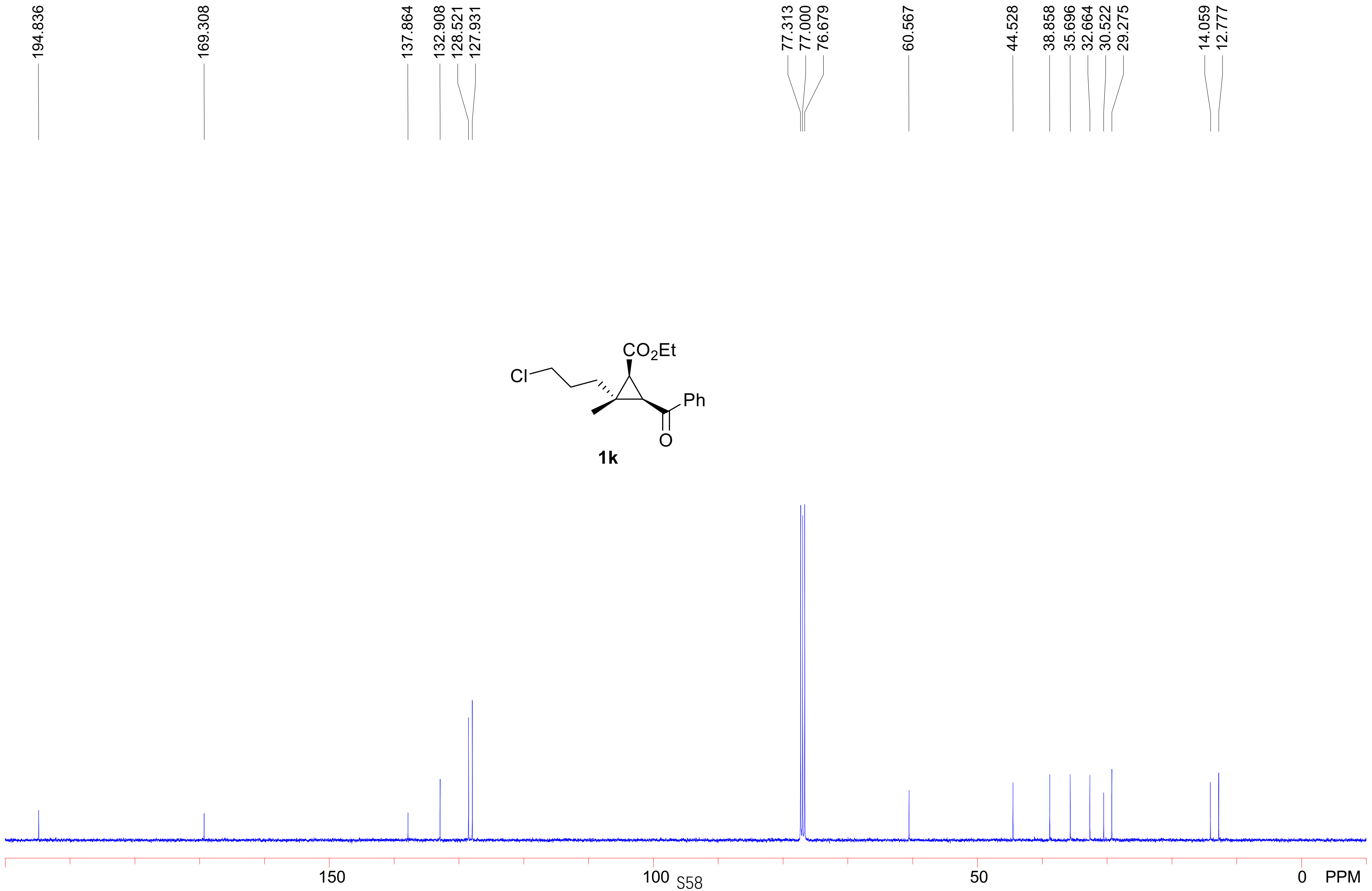

194.836
169.308
137.864
132.908
128.521
127.931
77.313
77.000
76.679
60.567
44.528
38.858
35.696
32.664
30.522
29.275
14.059
12.777
CO2Et
Cl
Ph
O
150
100
50
0
PPM

1k

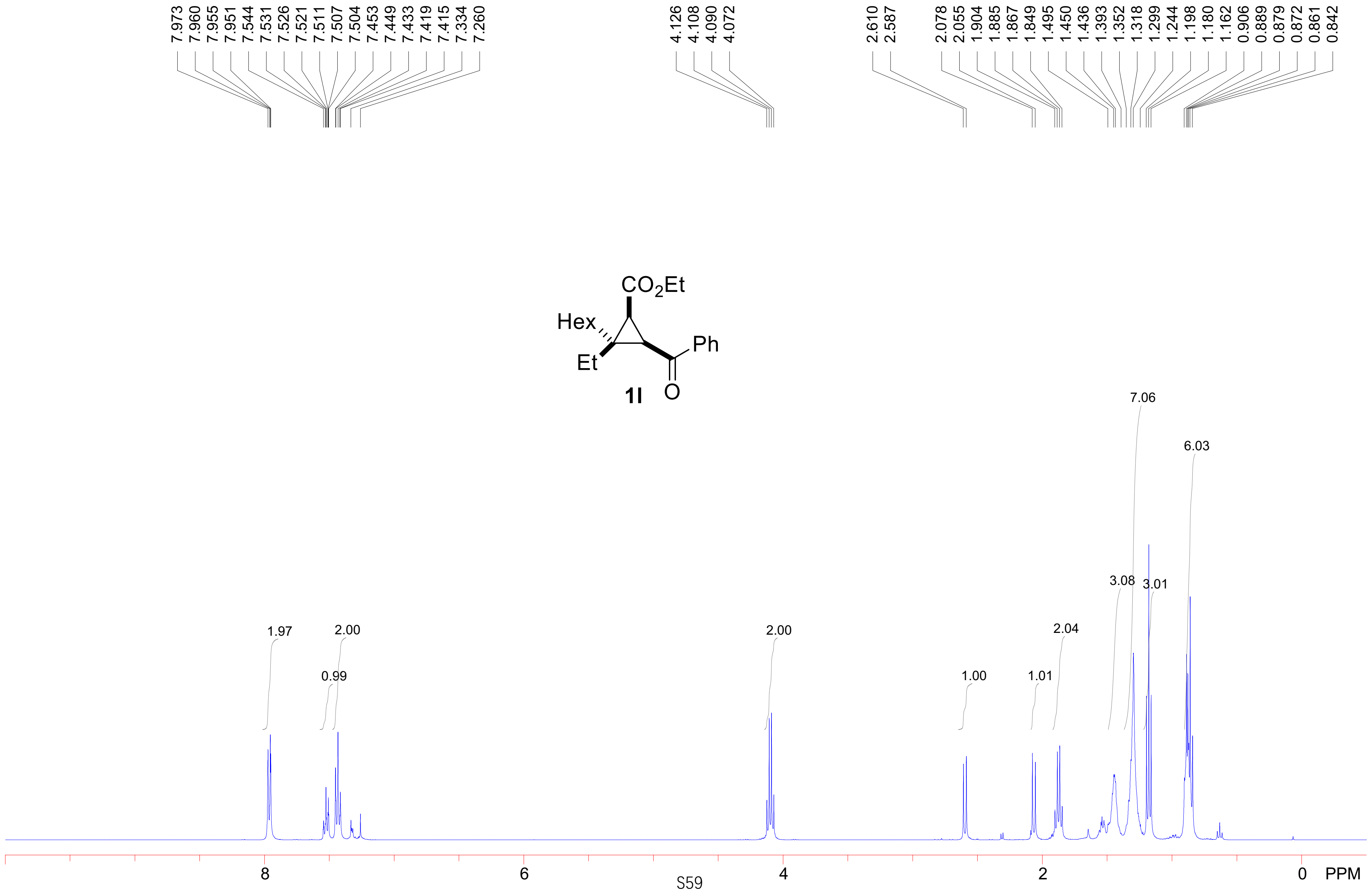
7.973
7.960
7.955
7.951
7.544
7.531
7.526
7.521
7.511
7.507
7.504
7.453
7.449
7.433
7.419
7.415
7.334
7.260
4.126
4.108
4.090
4.072
2.610
2.587
2.078
2.055
1.904
1.885
1.867
1.849
1.495
1.450
1.436
1.393
1.352
1.318
1.299
1.244
1.198
1.180
1.162
0.906
0.889
0.879
0.872
0.861
0.842
CO2Et
Hex
Et
Ph
O
1l
1.97
0.99
2.00
2.00
1.00
1.01
2.04
3.08
7.06
3.01
6.03
8
6
4
2
0
PPM

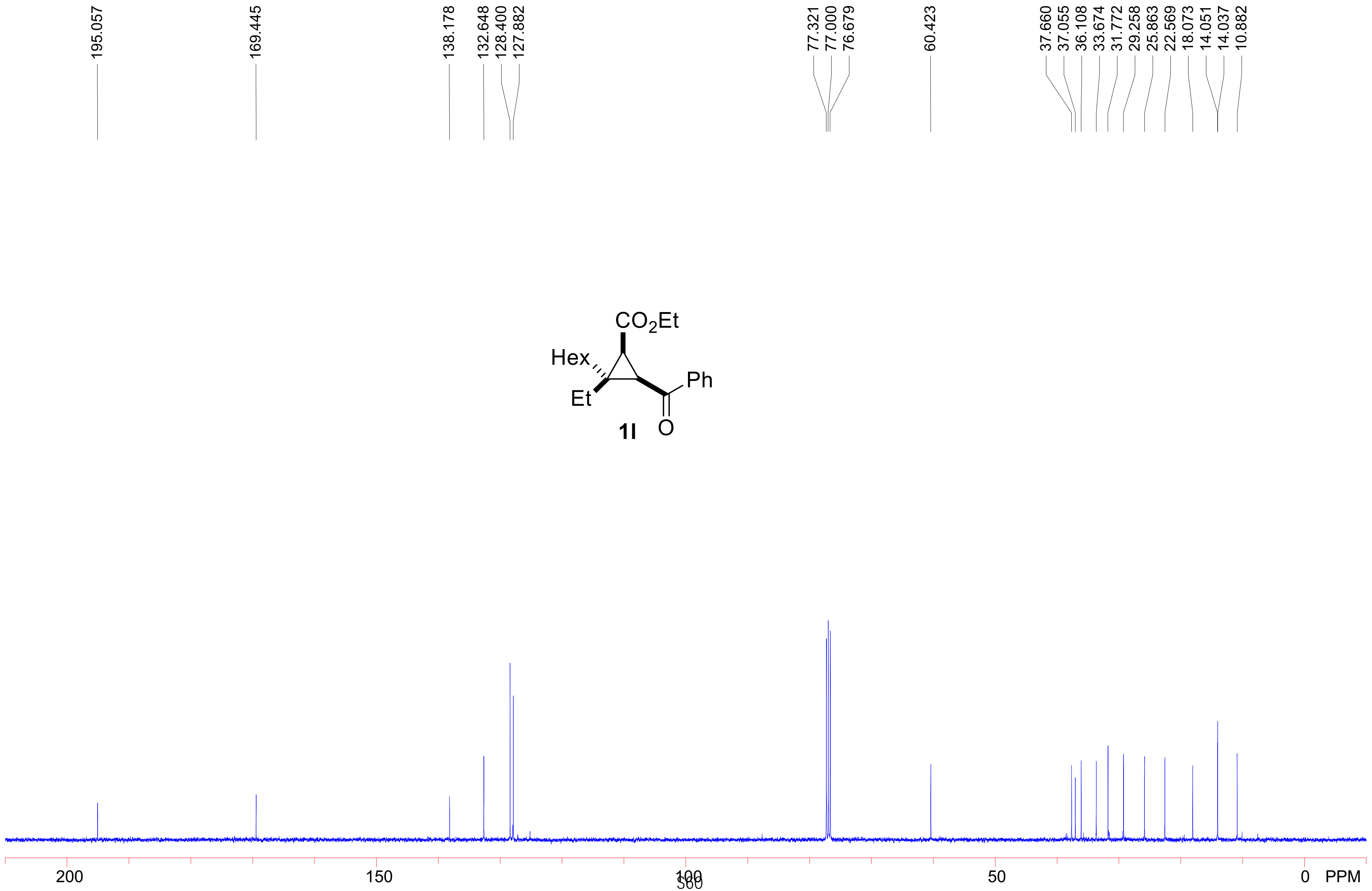
195.057
169.445
138.178
132.648
128.400
127.882
77.321
77.000
76.679
60.423
37.660
37.055
36.108
33.674
31.772
29.258
25.863
22.569
18.073
14.051
14.037
10.882
200
150
100
50
0
PPM
CO_2Et
Hex
Et
Ph
O

1l

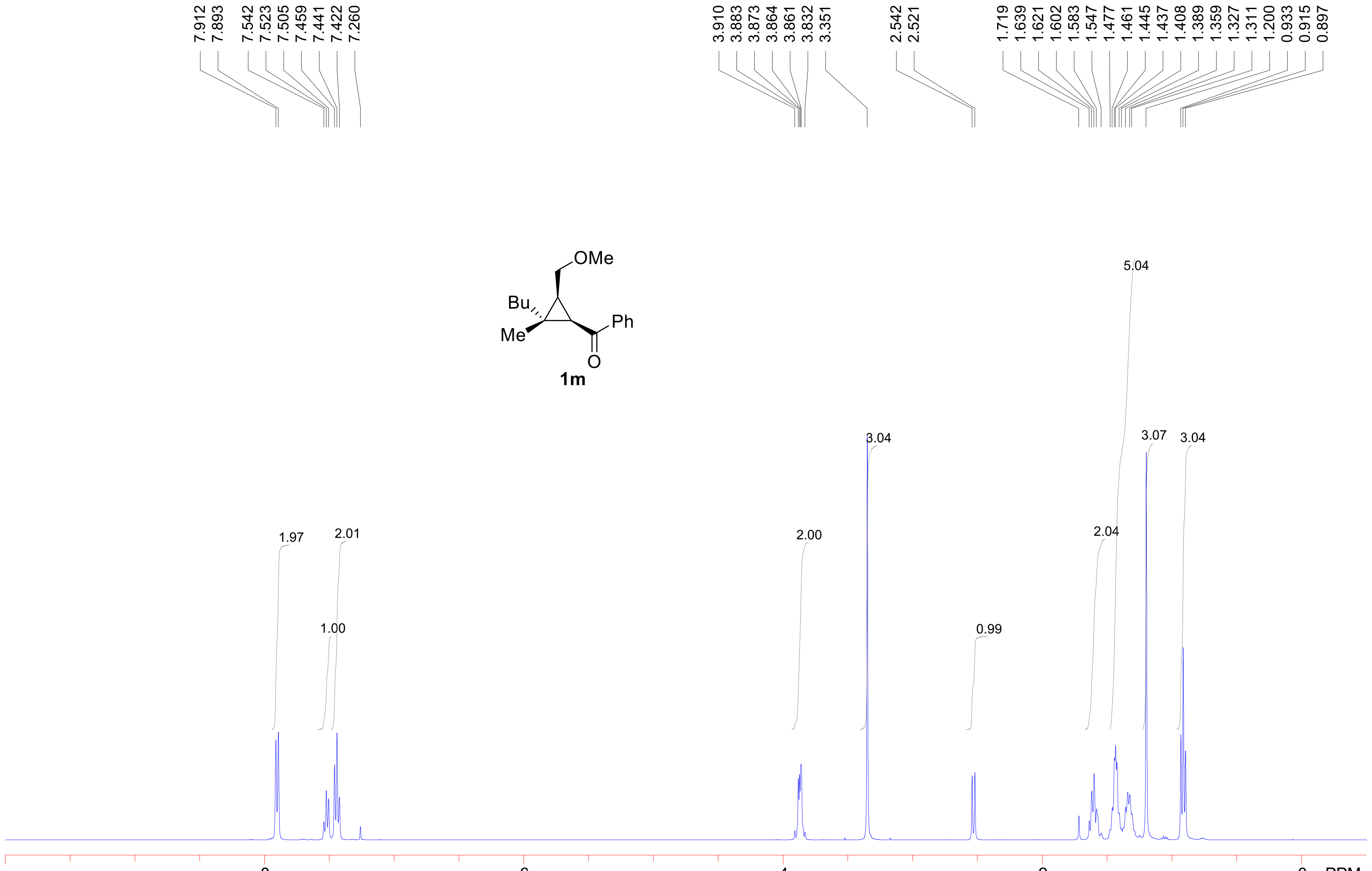
7.912
7.893
7.542
7.523
7.505
7.459
7.441
7.422
7.260
3.910
3.883
3.873
3.864
3.861
3.832
3.351
2.542
2.521
1.719
1.639
1.621
1.602
1.583
1.547
1.477
1.461
1.445
1.437
1.408
1.389
1.359
1.327
1.311
1.200
0.933
0.915
0.897
OMe
Bu
Me
Ph
O
1m
5.04
3.04
3.07
3.04
1.97
2.01
1.00
2.00
2.04
0.99
8
6
4
2
0
PPM

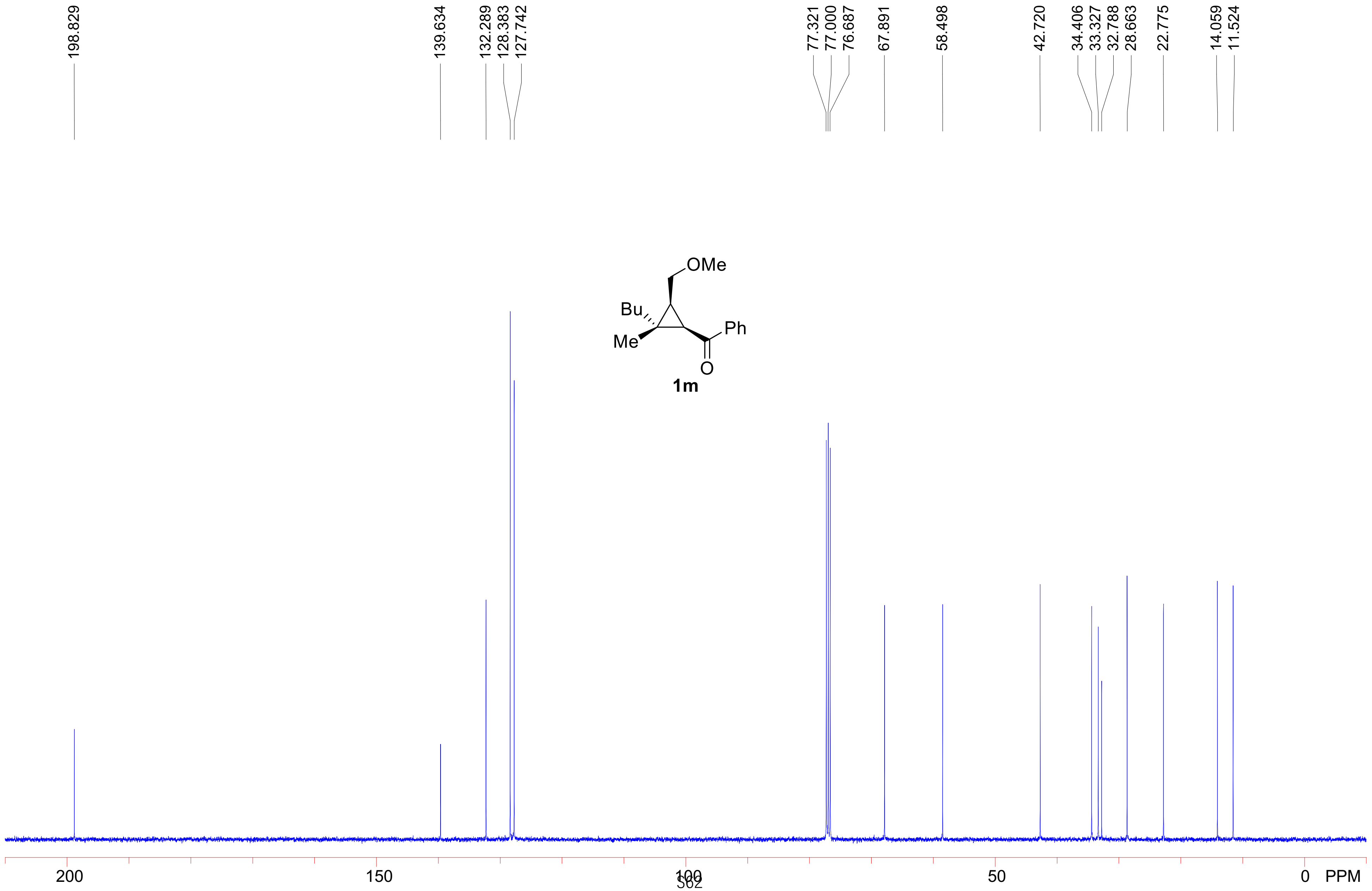
198.829
139.634
132.289
128.383
127.742
77.321
77.000
76.687
67.891
58.498
42.720
34.406
33.327
32.788
28.663
22.775
14.059
11.524
OMe
Bu
Me
Ph
O
1m
200
150
100
50
0
PPM

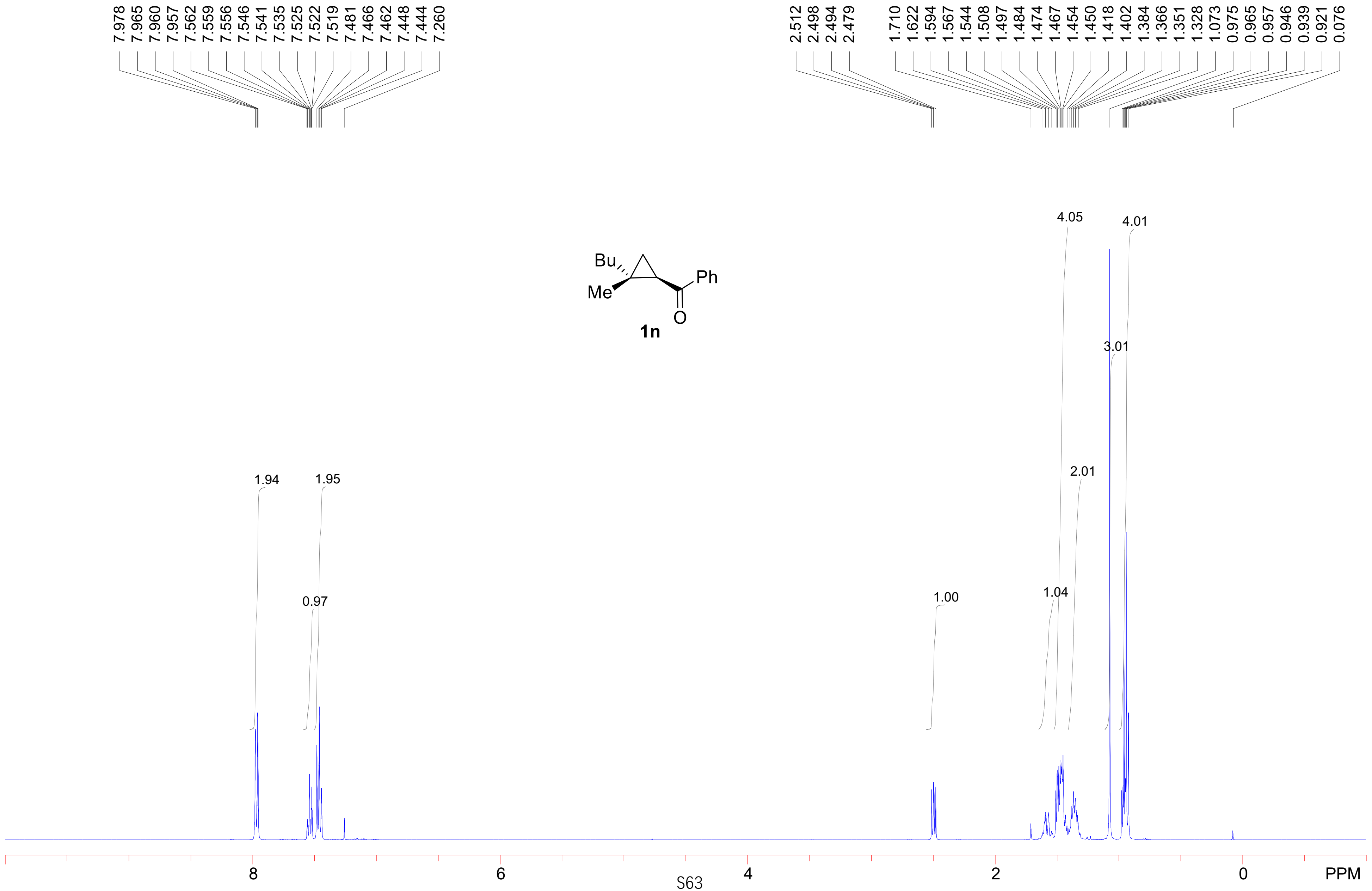
7.978 7.965 7.960 7.957 7.562 7.559 7.556 7.546 7.541 7.535 7.525 7.522 7.519 7.481 7.466 7.462 7.448 7.444 7.260
2.512 2.498 2.494 2.479 1.710 1.622 1.594 1.567 1.544 1.508 1.497 1.484 1.474 1.467 1.454 1.450 1.418 1.402 1.384 1.366 1.351 1.328 1.073 0.975 0.965 0.957 0.946 0.939 0.921 0.076
Bu
Me
Ph
O
1n
1.94
1.95
0.97
1.00
1.04
4.05
2.01
3.01
4.01
8
6
4
2
0
PPM

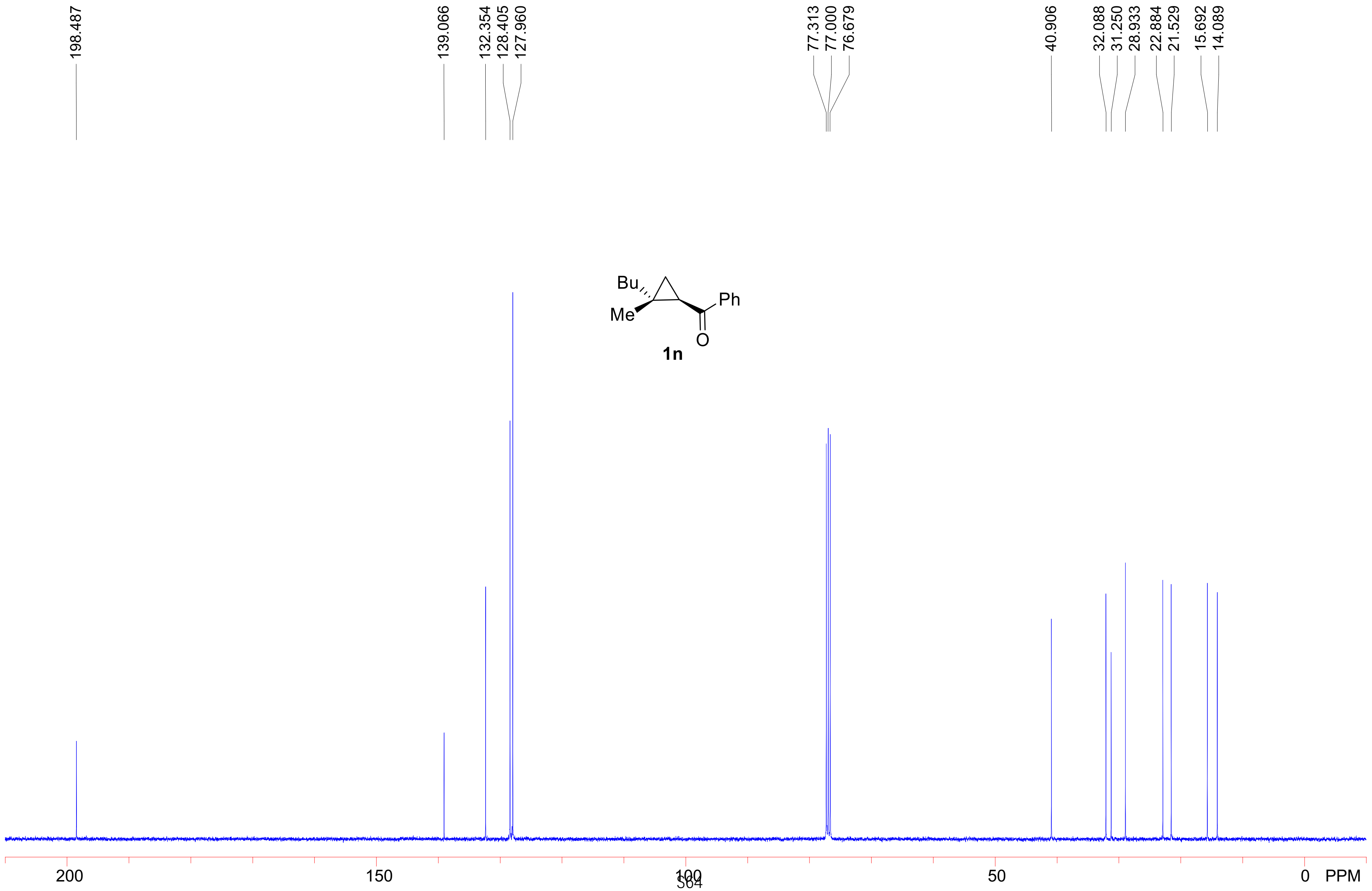
198.487
139.066
132.354
128.405
127.960
77.313
77.000
76.679
40.906
32.088
31.250
28.933
22.884
21.529
15.692
14.089
Bu
Me
Ph
O
1n
200
150
100
50
0
PPM

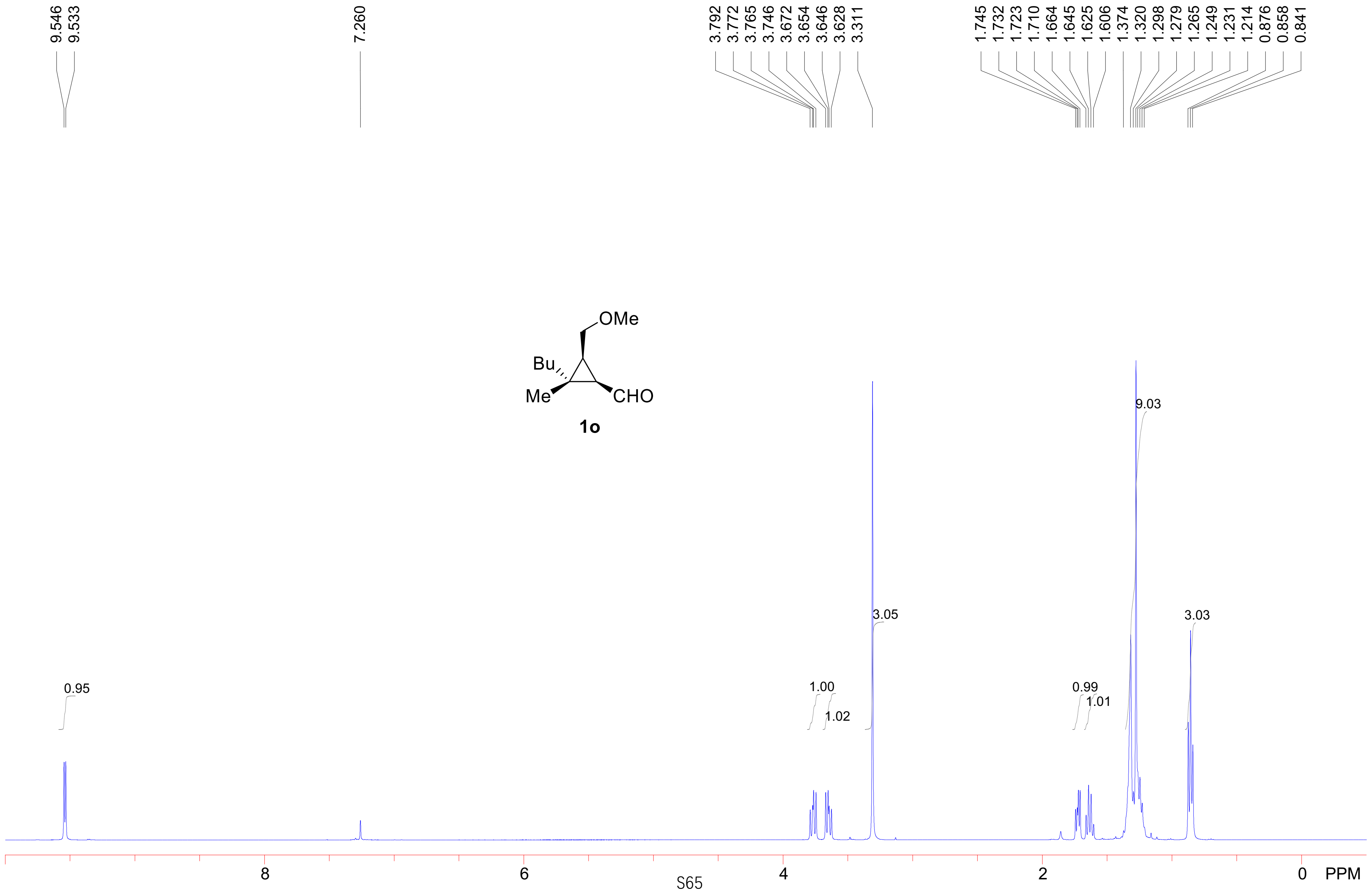
9.546
9.533
7.260
3.792
3.772
3.765
3.746
3.672
3.654
3.646
3.628
3.311
1.745
1.732
1.723
1.710
1.664
1.645
1.625
1.606
1.374
1.320
1.298
1.279
1.265
1.249
1.231
1.214
0.876
0.858
0.841
OMe
Bu
Me
CHO
1o
0.95
1.00
1.02
3.05
0.99
1.01
9.03
3.03
8
6
4
2
0
PPM

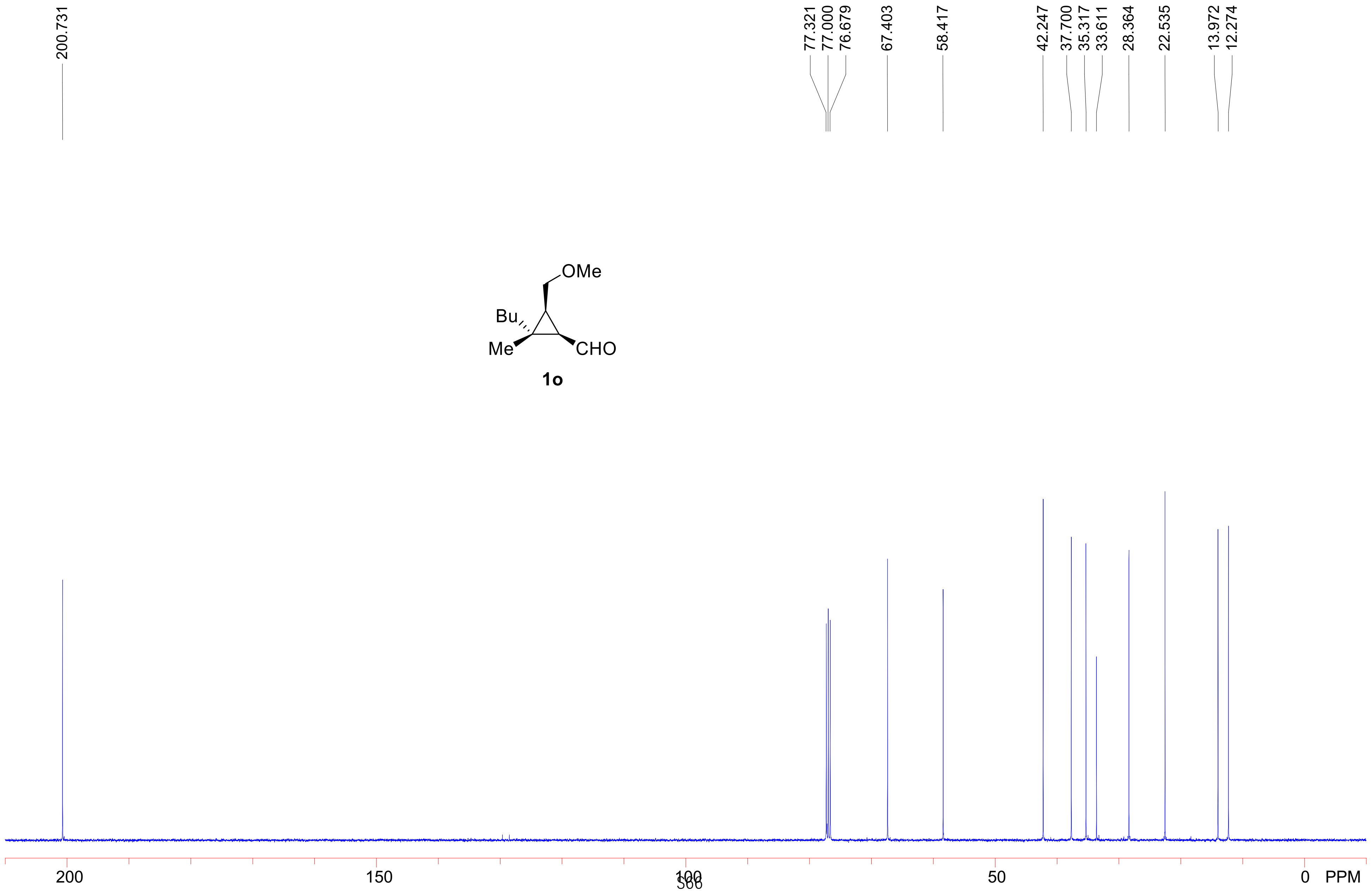

200.731
77.321
77.000
76.679
67.403
58.417
42.247
37.700
35.317
33.611
28.364
22.535
13.972
12.274
OMe
Bu
Me
CHO
1o
200
150
100
50
0
PPM

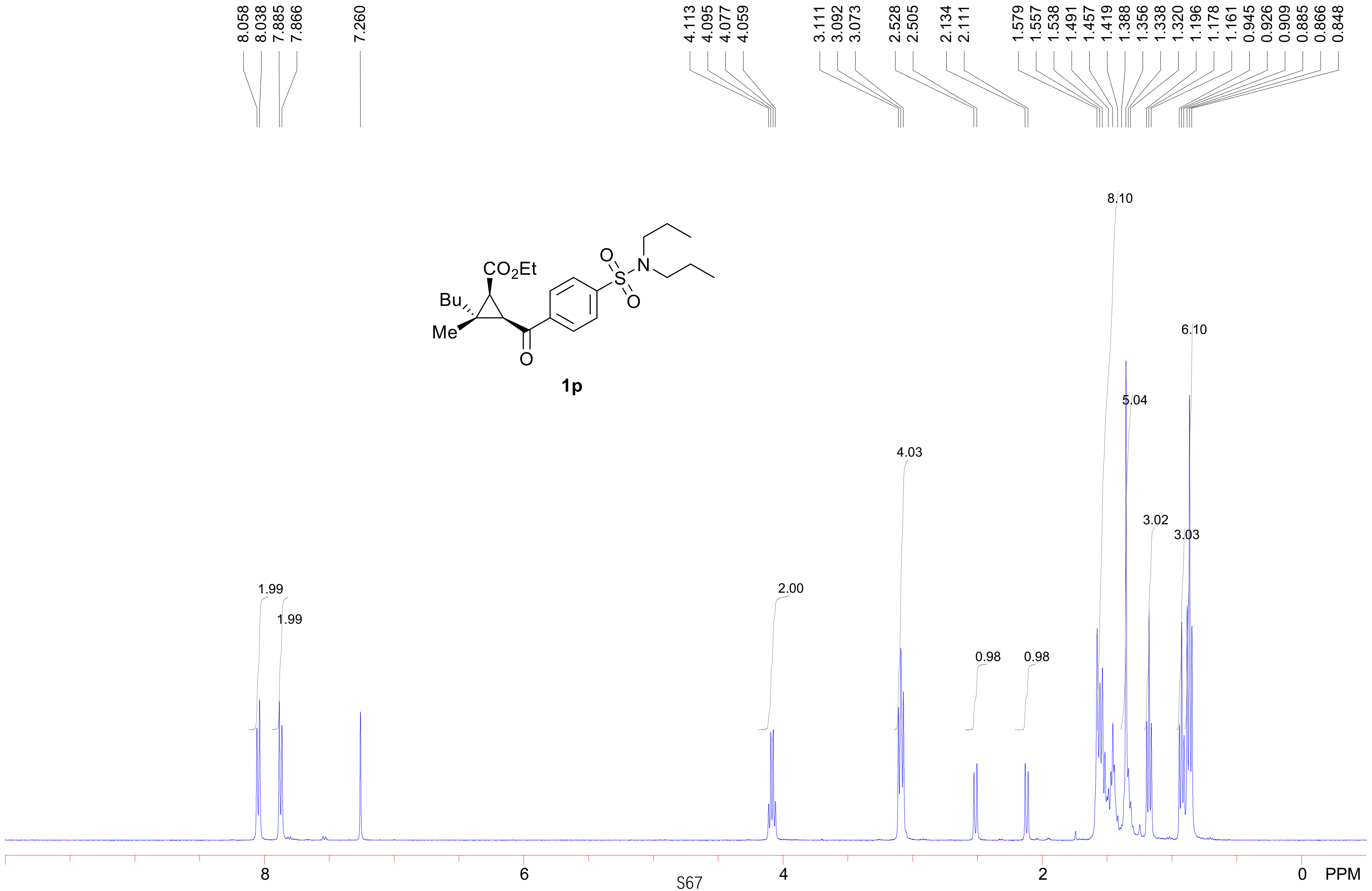
8.058
8.038
7.885
7.866
7.260
4.113
4.095
4.077
4.059
3.111
3.092
3.073
2.528
2.505
2.134
2.111
1.579
1.557
1.538
1.491
1.457
1.419
1.388
1.356
1.338
1.320
1.196
1.178
1.161
0.945
0.926
0.909
0.885
0.866
0.848
CO2Et
Bu
Me
O
S
N
1p
8.10
6.10
5.04
4.03
3.02
3.03
1.99
1.99
2.00
0.98
0.98
8
6
4
2
0
PPM

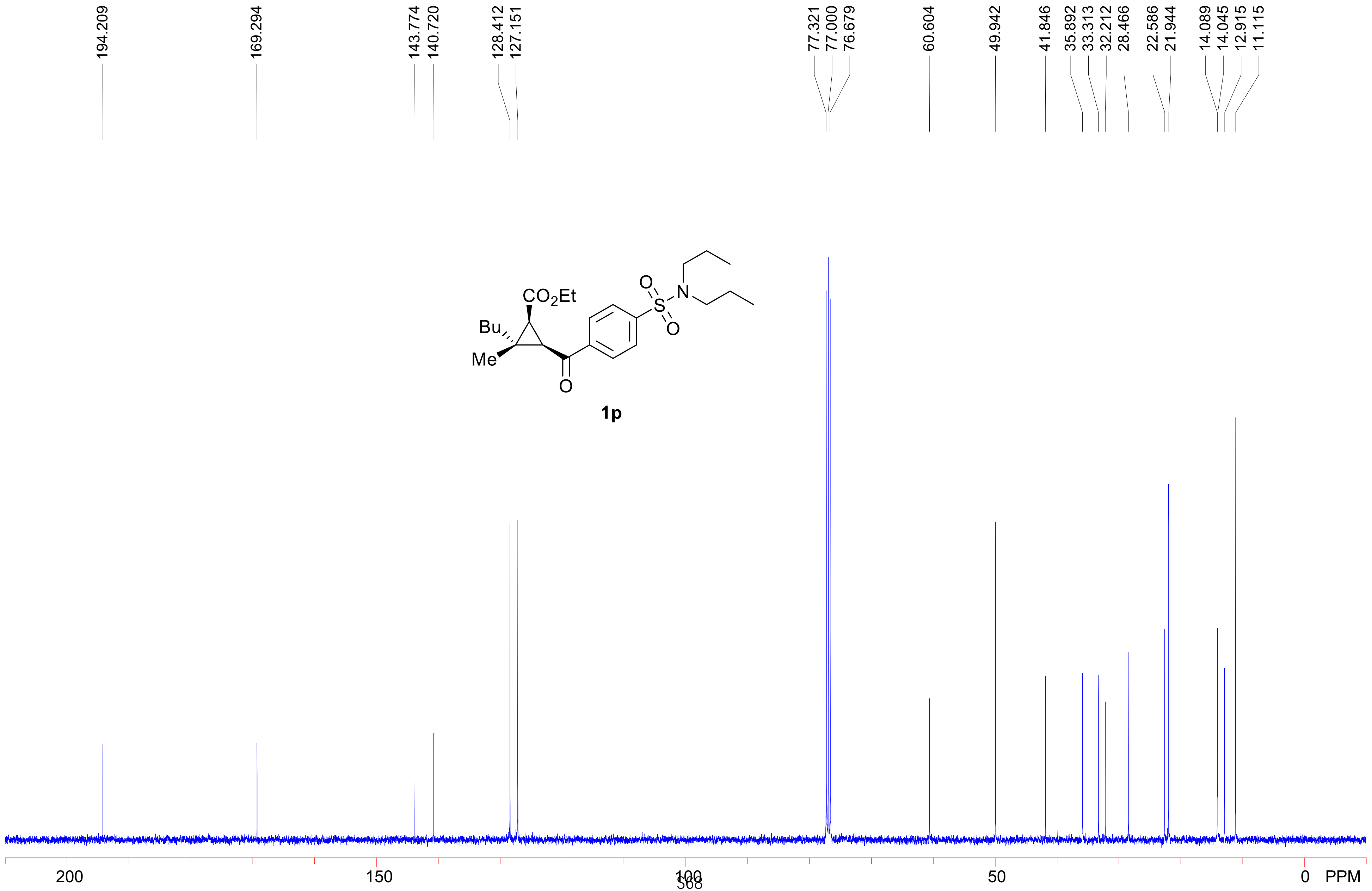
194.209
169.294
143.774
140.720
128.412
127.151
77.321
77.000
76.679
60.604
49.942
41.846
35.892
33.313
32.212
28.466
22.586
21.944
14.089
14.045
12.915
11.115
CO2Et
Bu
Me
O
S
N
1p
200
150
100
50
0
PPM

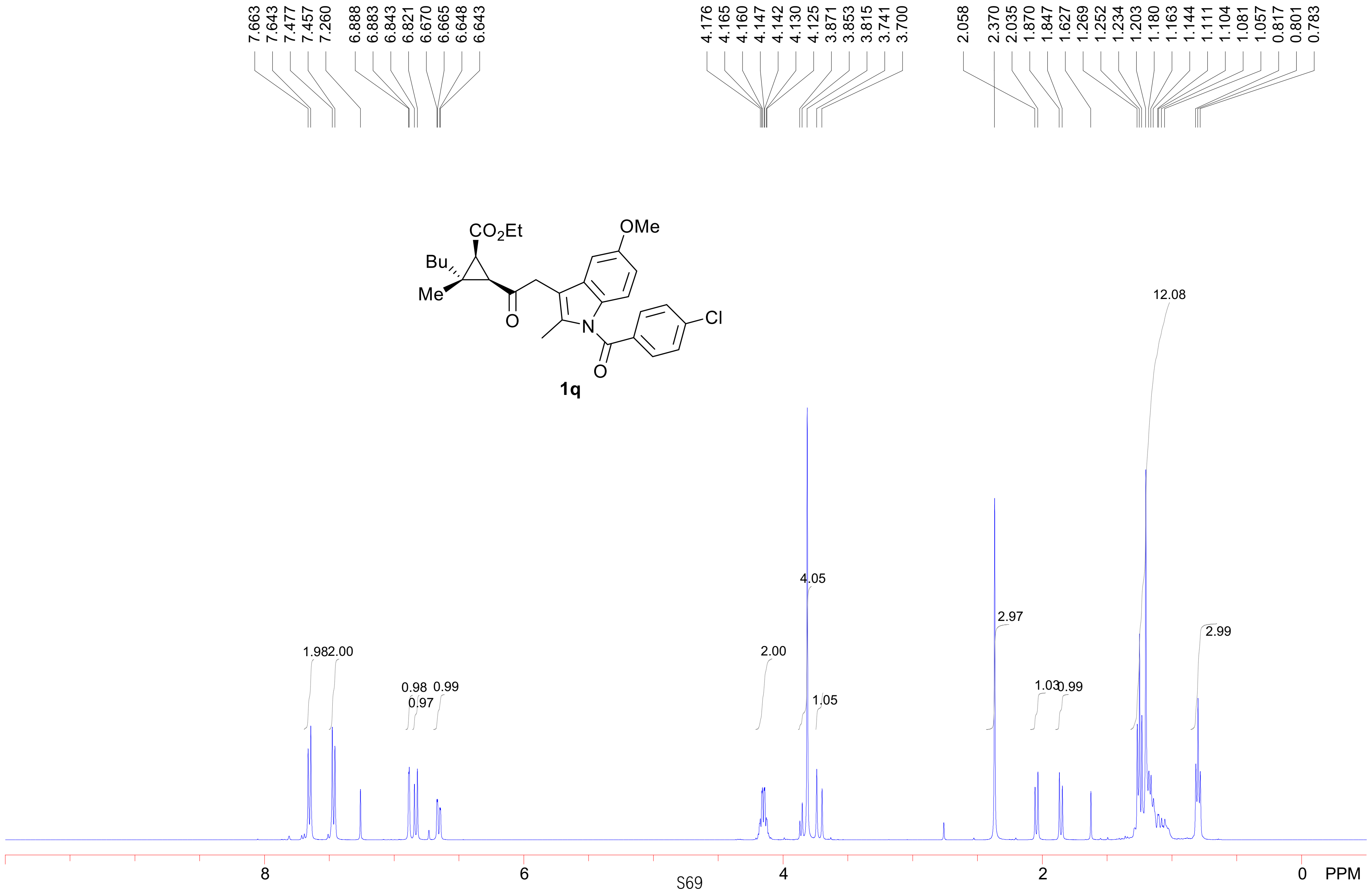

7.663
7.643
7.477
7.457
7.260
6.888
6.883
6.843
6.821
6.670
6.665
6.648
6.643
4.176
4.165
4.160
4.147
4.142
4.130
4.125
3.871
3.853
3.815
3.741
3.700
2.058
2.370
2.035
1.870
1.847
1.627
1.269
1.252
1.234
1.203
1.180
1.163
1.144
1.111
1.104
1.081
1.057
0.817
0.801
0.783
CO2Et
Bu
Me
OMe
O
N
Cl
O
12.08
4.05
2.97
2.99
1.98
2.00
0.98
0.97
0.99
2.00
1.05
1.03
0.99
8
6
4
2
0
PPM

1q

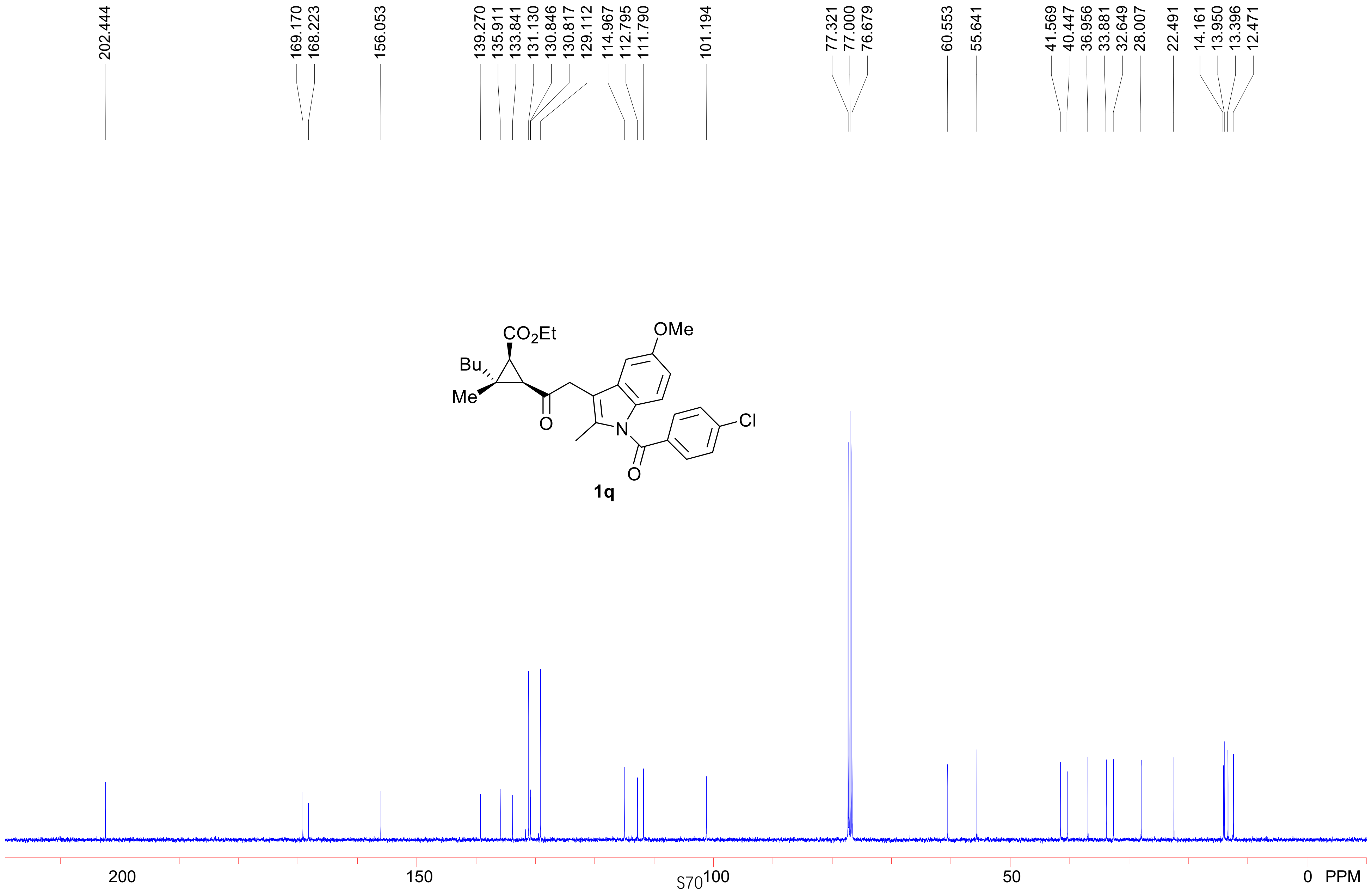

202.444
169.170
168.223
156.053
139.270
135.911
133.841
131.130
130.846
130.817
129.112
114.967
112.795
111.790
101.194
77.321
77.000
76.679
60.553
55.641
41.569
40.447
36.956
33.881
32.649
28.007
22.491
14.161
13.950
13.396
12.471
CO2Et
OMe
Bu
Me
O
N
Cl
O
1q
200
150
100
50
0 PPM

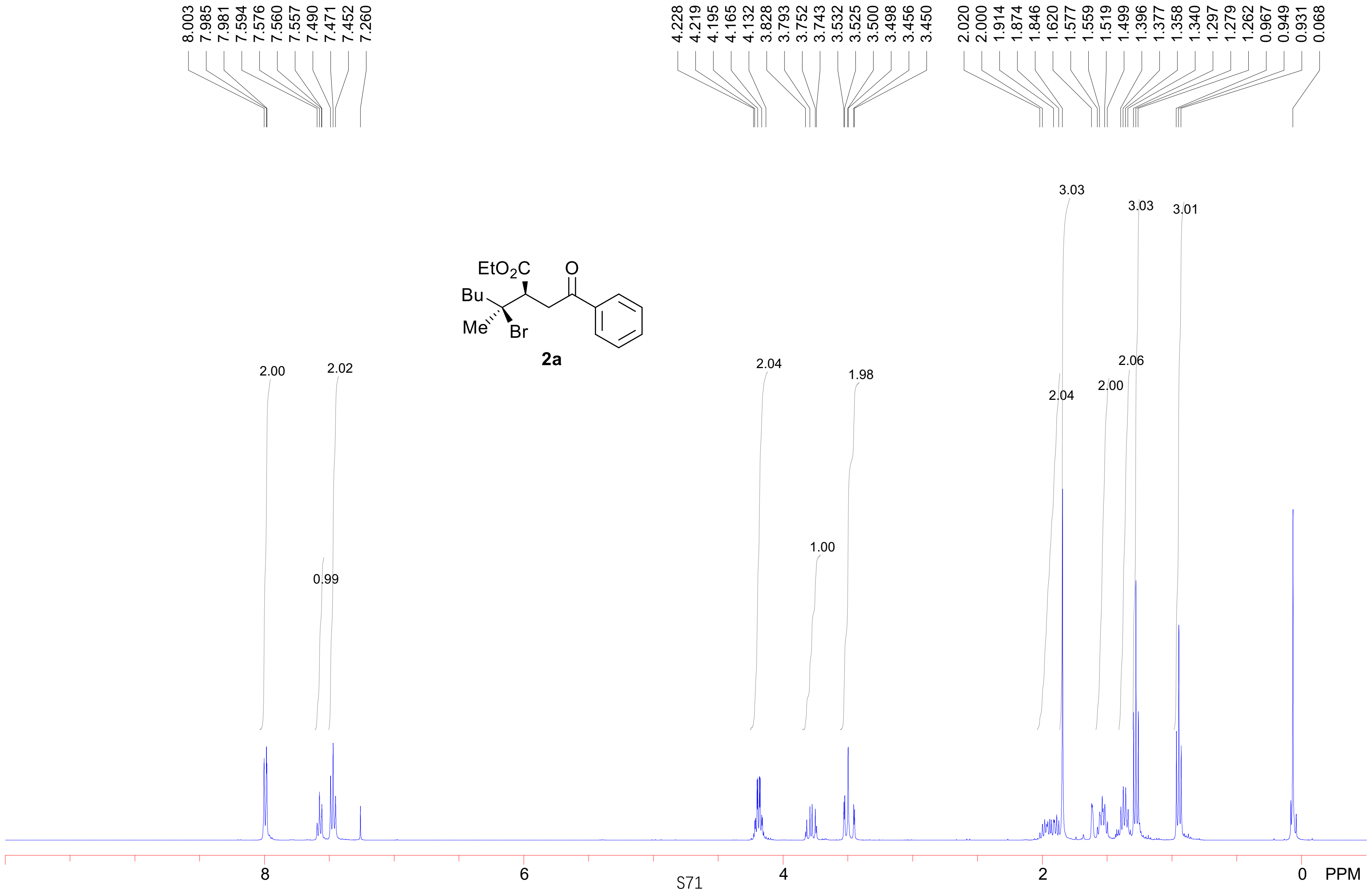
8.003 7.985 7.981 7.594 7.576 7.560 7.557 7.490 7.471 7.452 7.260
4.228 4.219 4.195 4.165 4.132 3.828 3.793 3.752 3.743 3.532 3.525 3.500 3.498 3.456 3.450
2.020 2.000 1.914 1.874 1.846 1.620 1.577 1.559 1.519 1.499 1.396 1.377 1.358 1.340 1.297 1.279 1.262 0.967 0.949 0.931 0.068
EtO2C
O
Bu
Me
Br
2a
2.00
0.99
2.02
2.04
1.00
1.98
2.04
3.03
2.00
2.06
3.03
3.01
8
6
4
2
0
PPM

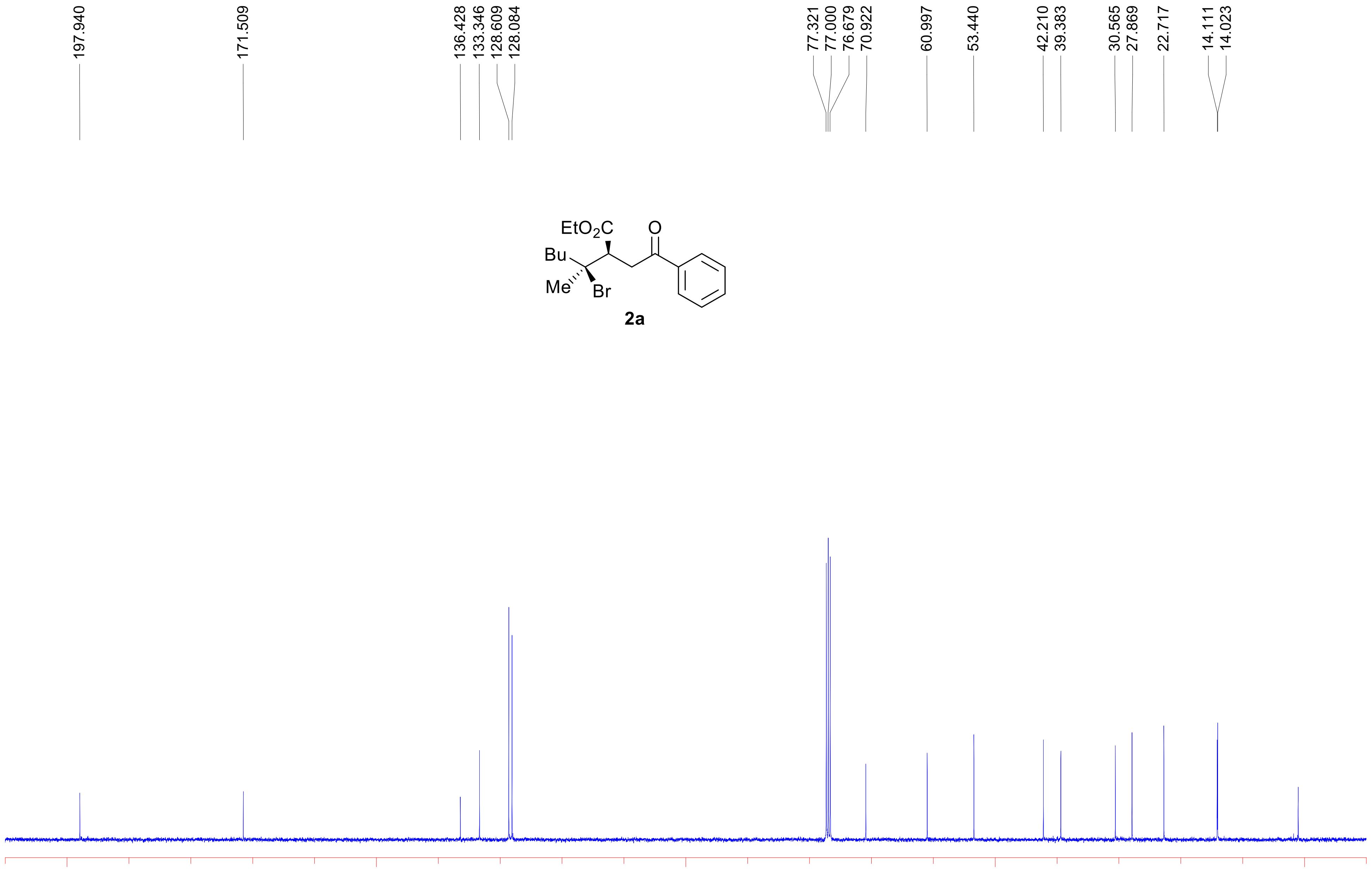
197.940
171.509
136.428
133.346
128.609
128.084
77.321
77.000
76.679
70.922
60.997
53.440
42.210
39.383
30.565
27.869
22.717
14.111
14.023
EtO2C
O
Bu
Me
Br
2a
200
150
100
50
0
PPM

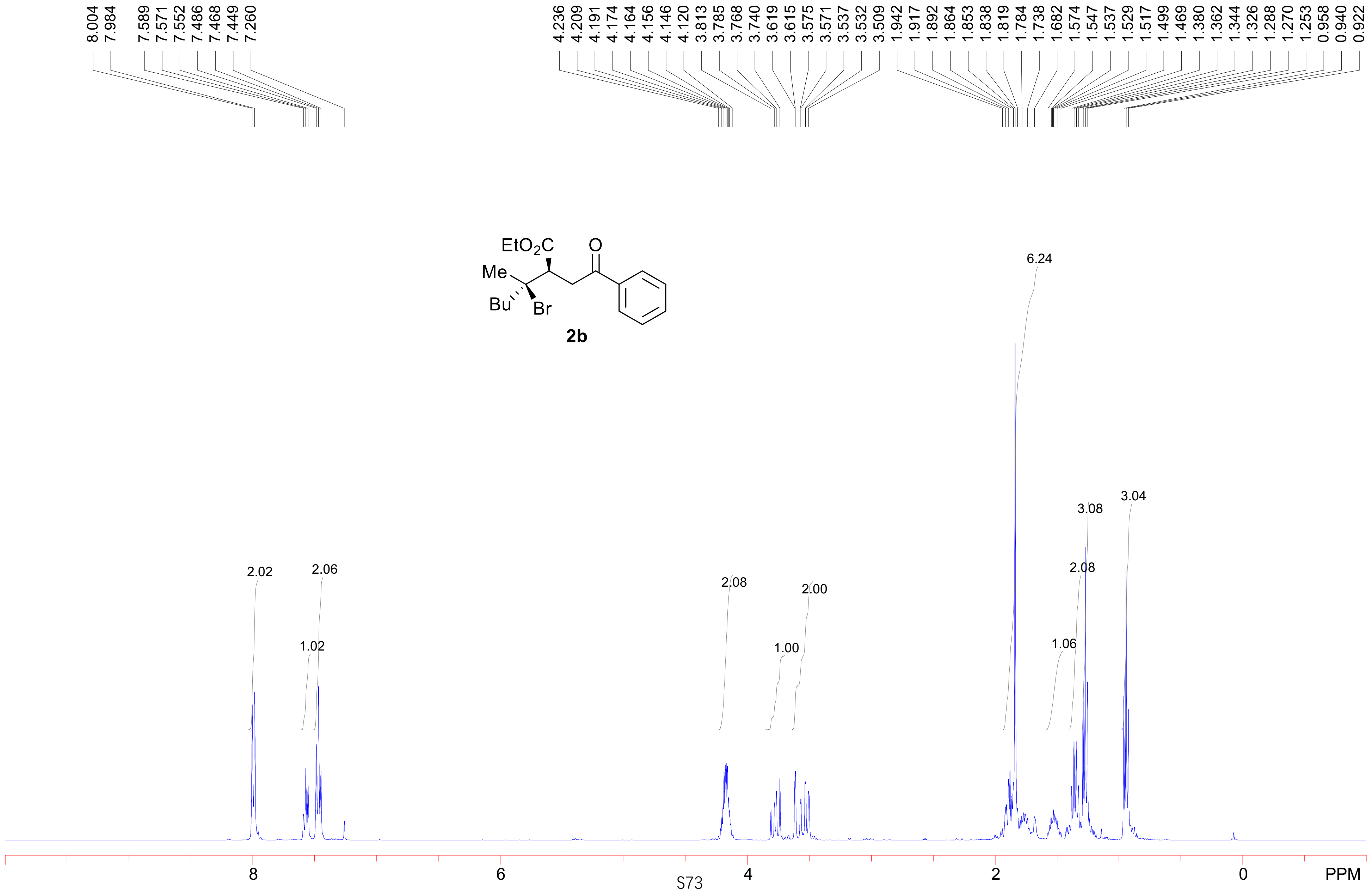
8.004
7.984
7.589
7.571
7.552
7.486
7.468
7.449
7.260
4.236
4.209
4.191
4.174
4.164
4.156
4.146
4.120
3.813
3.785
3.768
3.740
3.619
3.615
3.575
3.571
3.537
3.532
3.509
1.942
1.917
1.892
1.864
1.853
1.838
1.819
1.784
1.738
1.682
1.574
1.547
1.537
1.529
1.517
1.499
1.469
1.380
1.362
1.344
1.326
1.288
1.270
1.253
0.958
0.940
0.922
EtO2C
O
Me
Bu
Br
2b
6.24
3.08
3.04
2.02
2.06
2.08
2.00
2.08
1.02
1.00
1.06
8
6
4
2
0
PPM

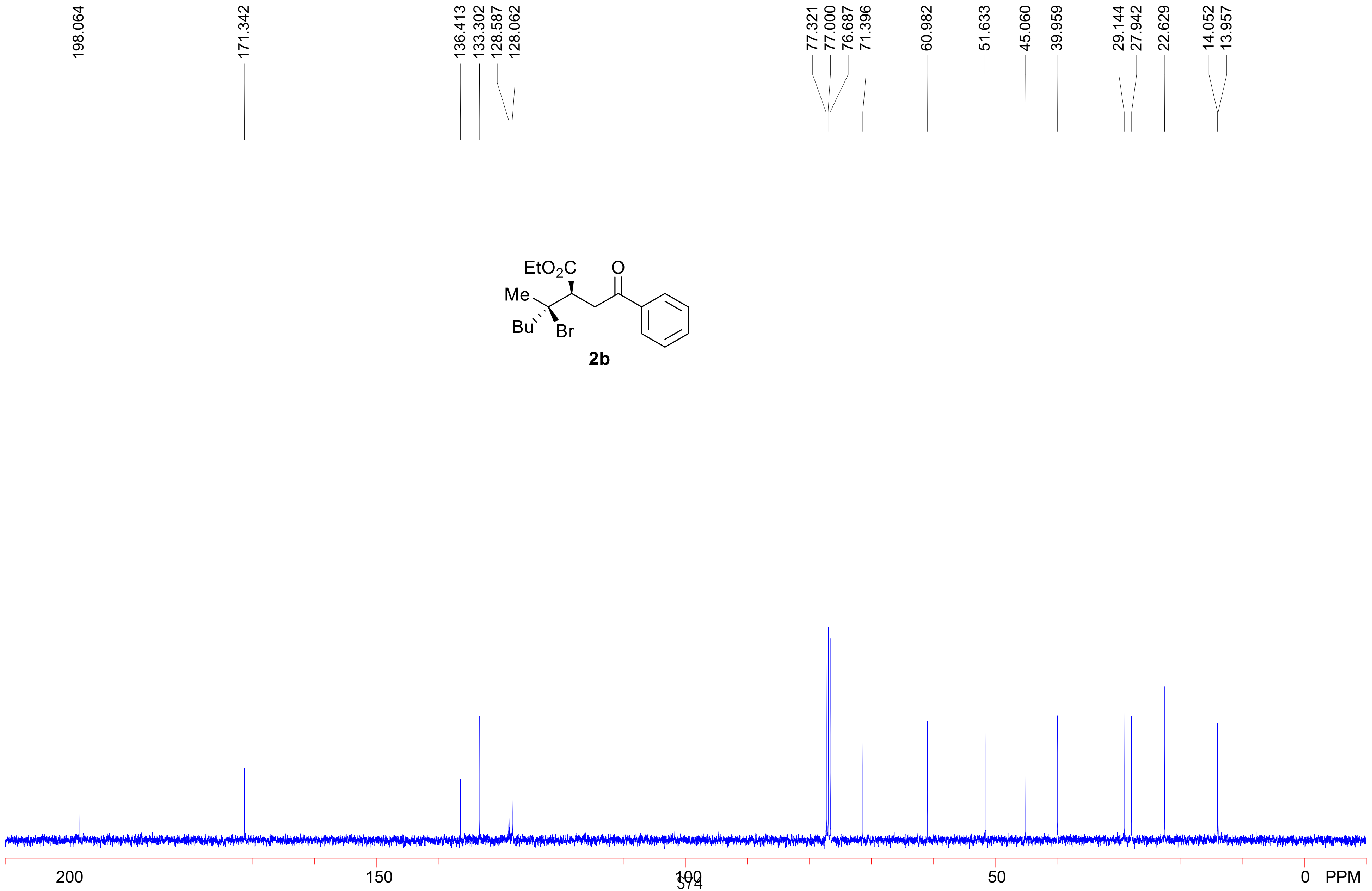
198.064
171.342
136.413
133.302
128.587
128.062
77.321
77.000
76.687
71.396
60.982
51.633
45.060
39.959
29.144
27.942
22.629
14.052
13.957
EtO2C
O
Me
Bu
Br
200
150
100
50
0
PPM

2b

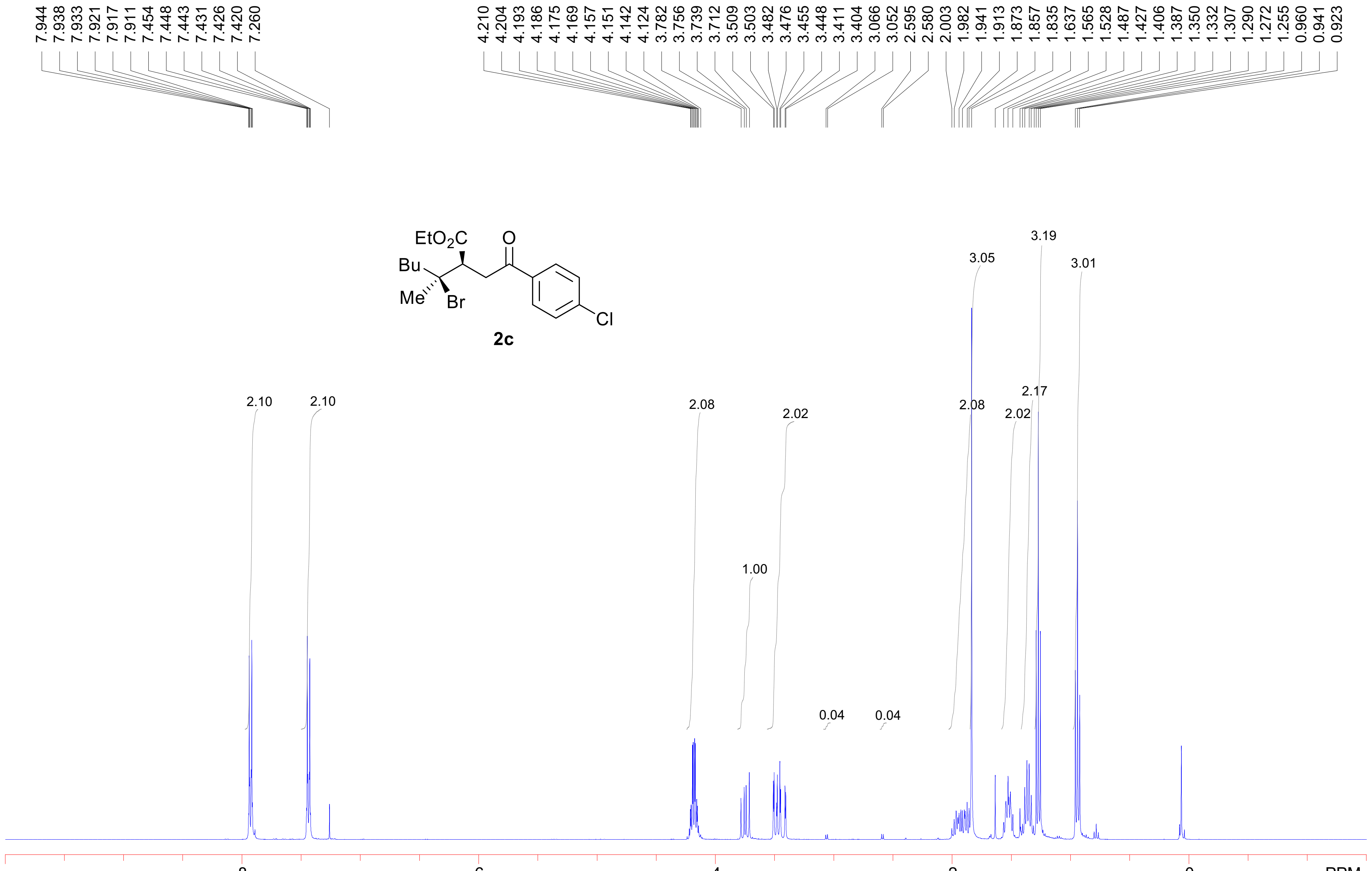
7.944 7.938 7.933 7.921 7.917 7.911 7.454 7.448 7.443 7.431 7.426 7.420 7.260
4.210 4.204 4.193 4.186 4.175 4.169 4.157 4.151 4.142 4.124 3.782 3.756 3.739 3.712 3.509 3.503 3.482 3.476 3.455 3.448 3.411 3.404 3.066 3.052 2.595 2.580 2.003 1.982 1.941 1.913 1.873 1.857 1.835 1.637 1.565 1.528 1.487 1.427 1.406 1.387 1.350 1.332 1.307 1.290 1.272 1.255 0.960 0.941 0.923
EtO2C
O
Bu
Me
Br
Cl
2c
2.10
2.10
2.08
1.00
2.02
0.04
0.04
3.05
2.08
2.02
3.19
2.17
3.01
8
6
4
2
0
PPM

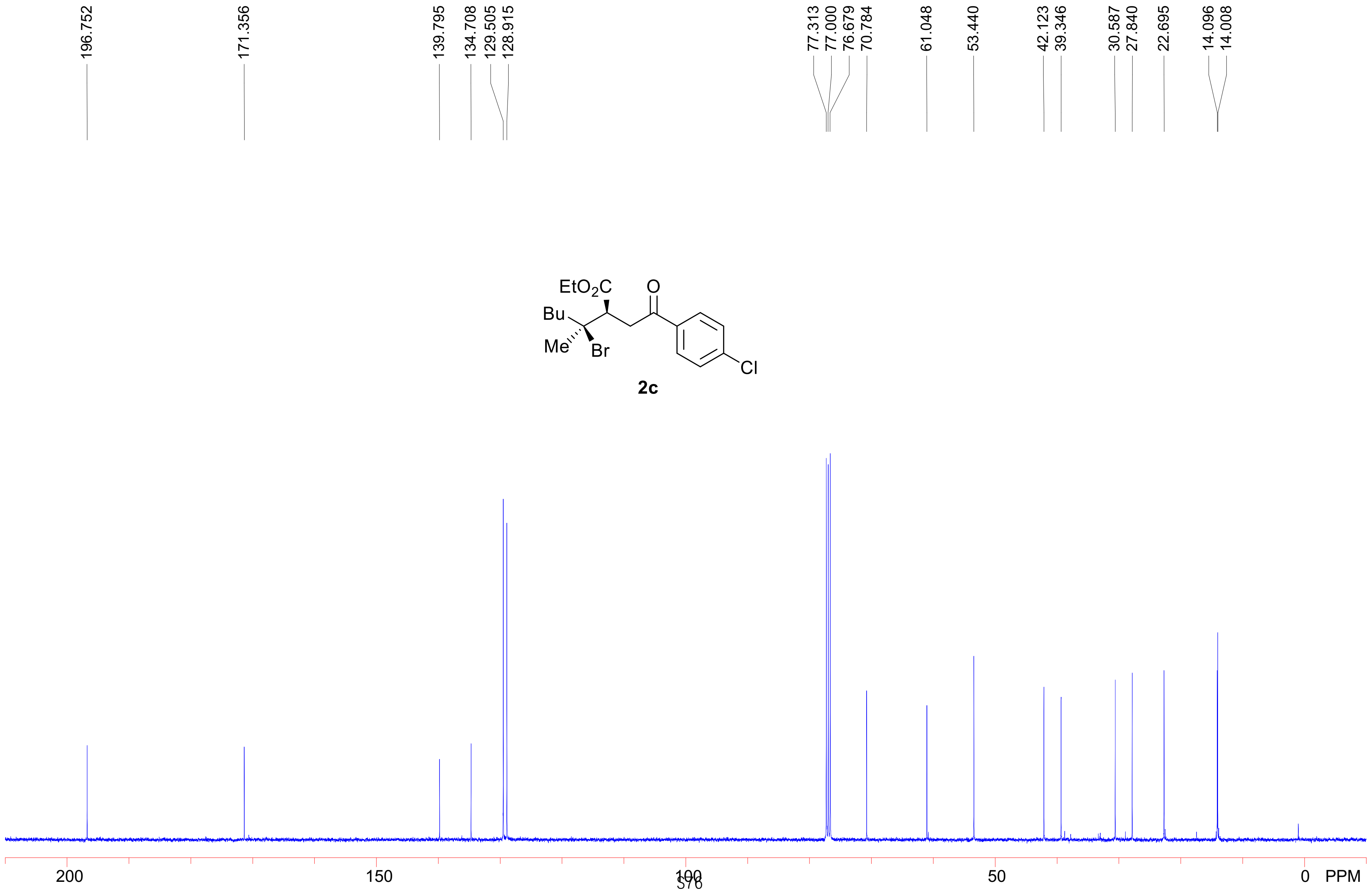
196.752
171.356
139.795
134.708
129.505
128.915
77.313
77.000
76.679
70.784
61.048
53.440
42.123
39.346
30.587
27.840
22.695
14.096
14.008
EtO2C
O
Bu
Me
Br
Cl
2c
200
150
100
50
0
PPM

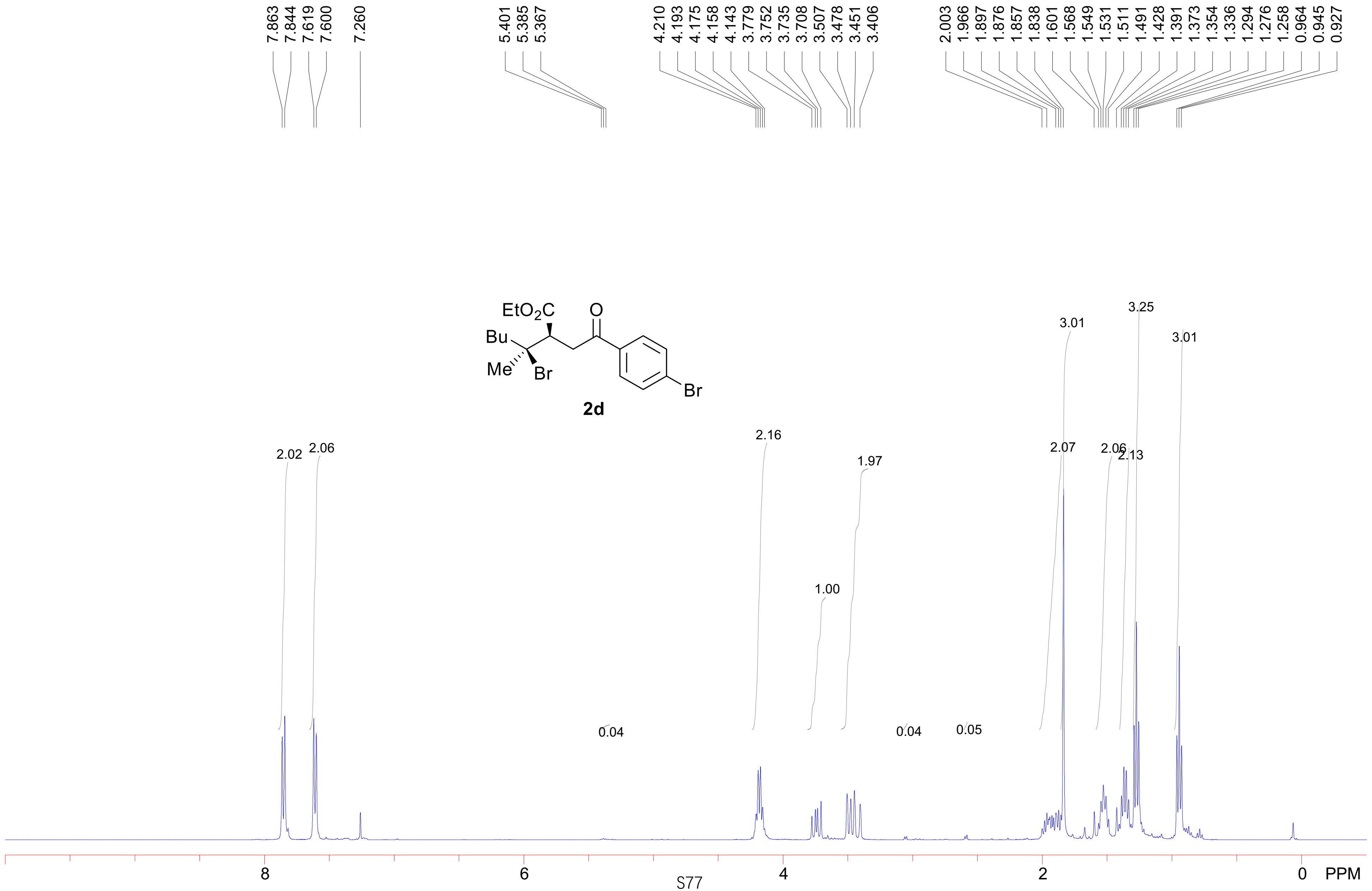
7.863
7.844
7.619
7.600
7.260
5.401
5.385
5.367
4.210
4.193
4.175
4.158
4.143
3.779
3.752
3.735
3.708
3.507
3.478
3.451
3.406
2.003
1.966
1.897
1.876
1.857
1.838
1.601
1.568
1.549
1.531
1.511
1.491
1.428
1.391
1.373
1.354
1.336
1.294
1.276
1.258
0.964
0.945
0.927
EtO2C
O
Bu
Me
Br
Br
2d
2.02
2.06
0.04
2.16
1.00
1.97
0.04
0.05
2.07
3.01
2.06
2.13
3.25
3.01
8
6
4
2
0
PPM

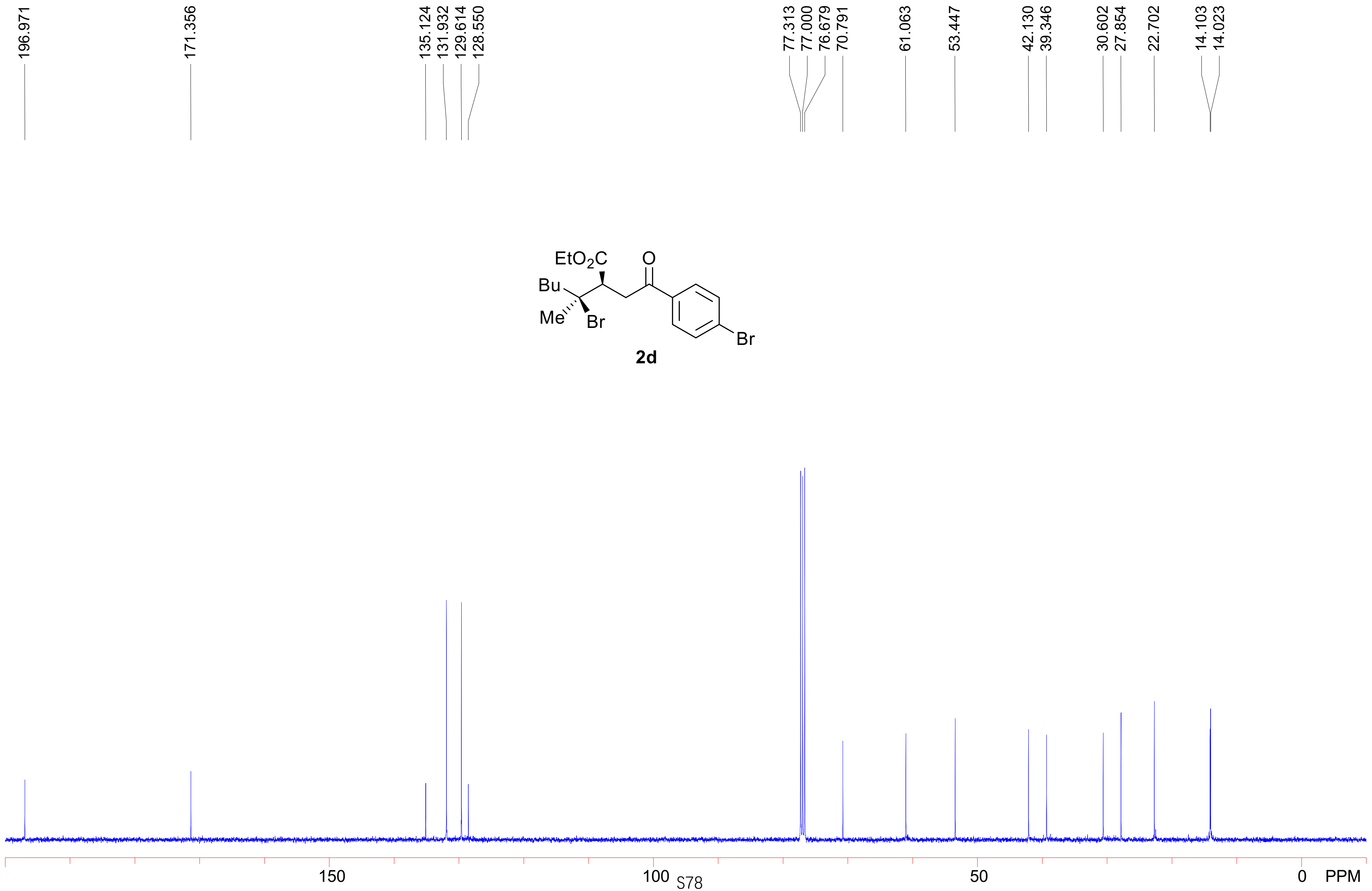
196.971
171.356
135.124
131.932
129.614
128.550
77.313
77.000
76.679
70.791
61.063
53.447
42.130
39.346
30.602
27.854
22.702
14.103
14.023
EtO2C
O
Bu
Me
Br
Br
2d
150
100
50
0
PPM

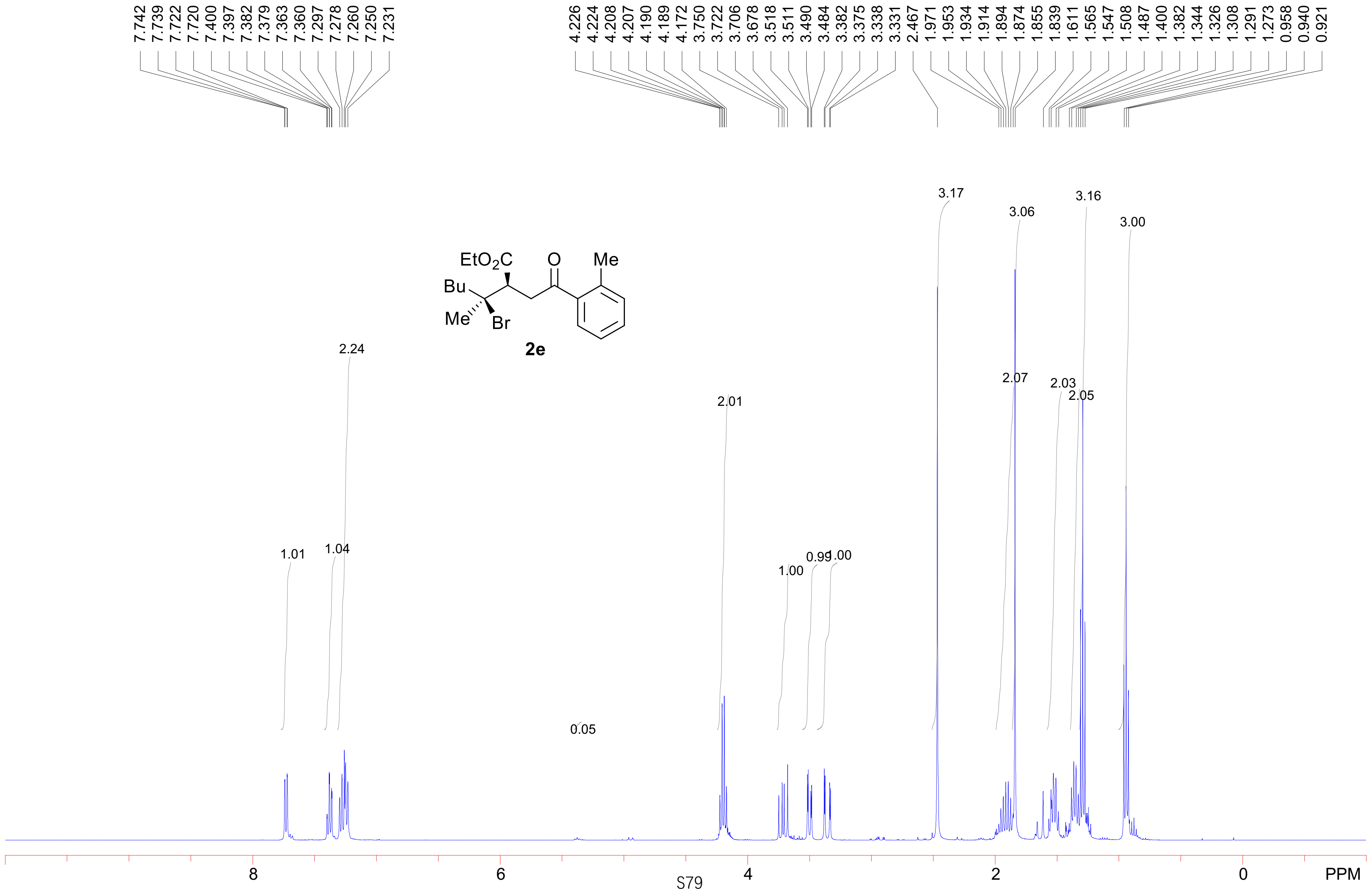
7.742
7.739
7.722
7.720
7.400
7.397
7.382
7.379
7.363
7.360
7.297
7.278
7.260
7.250
7.231
4.226
4.224
4.208
4.207
4.190
4.189
4.172
3.750
3.722
3.706
3.678
3.518
3.511
3.490
3.484
3.382
3.375
3.338
3.331
2.467
1.971
1.953
1.934
1.914
1.894
1.874
1.855
1.839
1.611
1.565
1.547
1.508
1.487
1.400
1.382
1.344
1.326
1.308
1.291
1.273
0.958
0.940
0.921
3.17
3.06
3.16
3.00
EtO2C
O
Me
Bu
Me
Br
2e
2.24
2.07
2.03
2.05
2.01
1.01
1.04
1.00
0.99
1.00
0.05
8
6
4
2
0
PPM

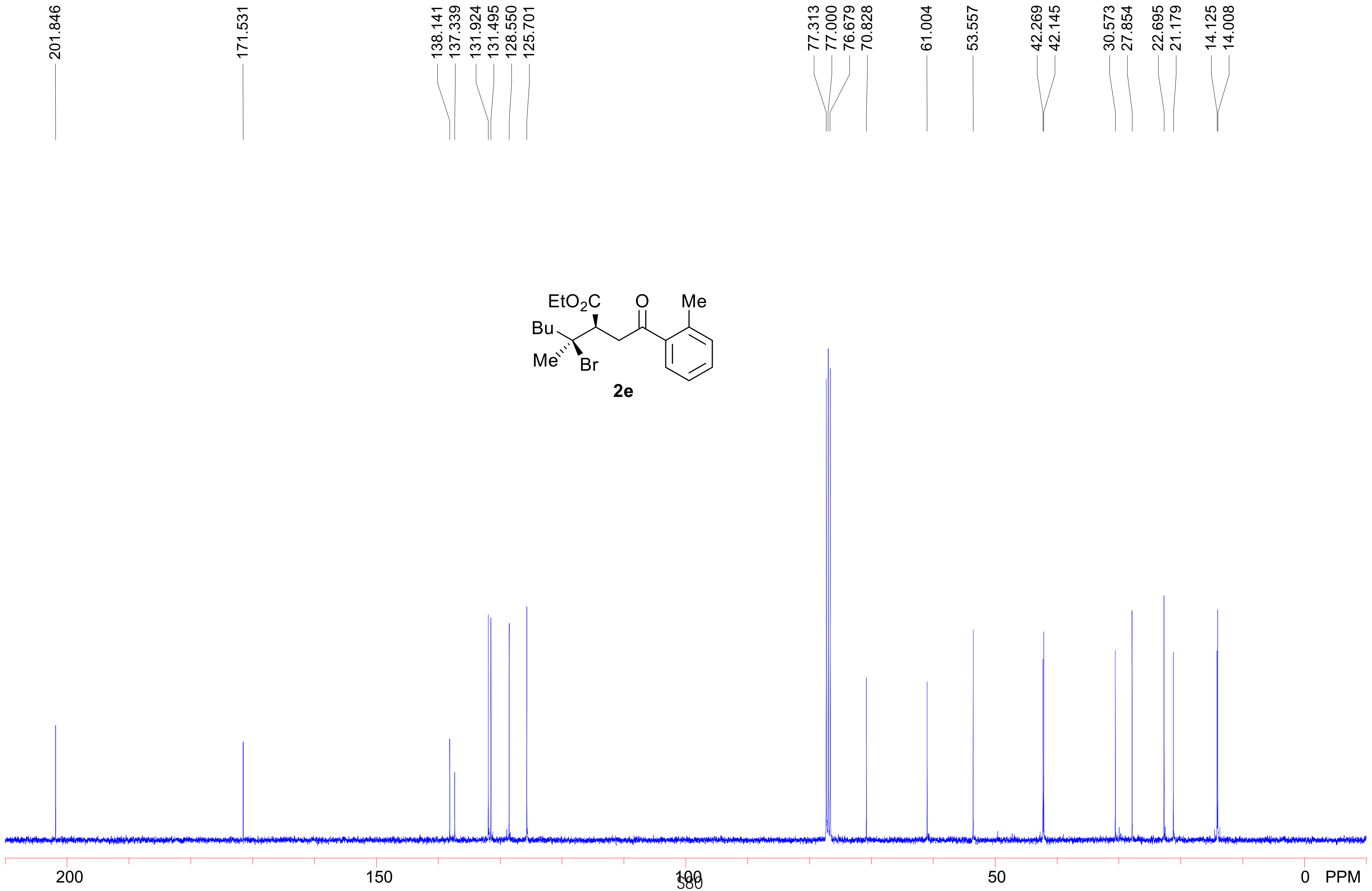
201.846
171.531
138.141
137.339
131.924
131.495
128.550
125.701
77.313
77.000
76.679
70.828
61.004
53.557
42.269
42.145
30.573
27.854
22.695
21.179
14.125
14.008
EtO2C
O
Me
Bu
Me
Br
2e
200
150
100
50
0
PPM

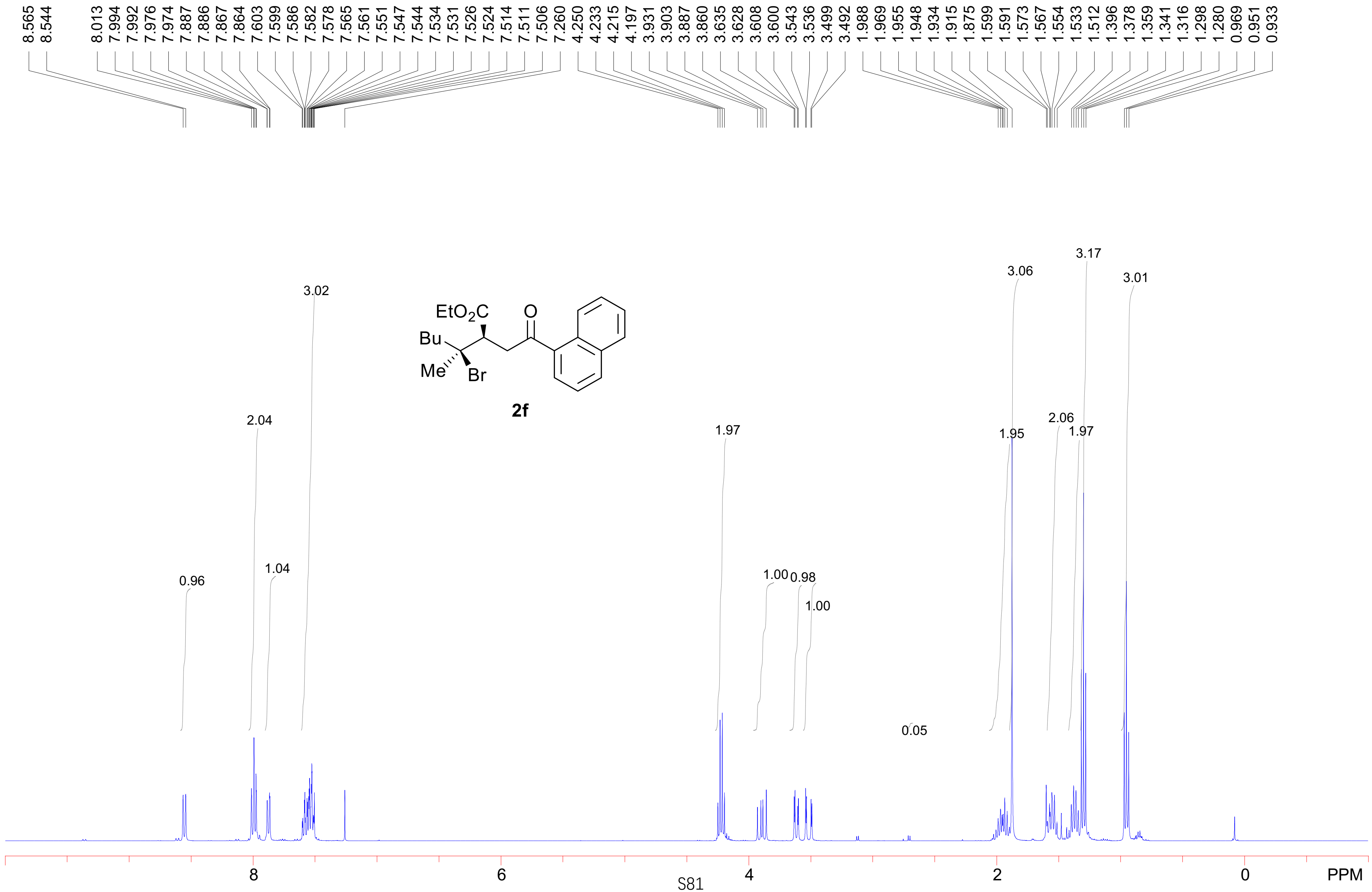
8.565 8.544 8.013 7.994 7.992 7.976 7.974 7.887 7.886 7.867 7.864 7.603 7.599 7.586 7.582 7.578 7.565 7.561 7.551 7.547 7.544 7.534 7.531 7.526 7.524 7.514 7.511 7.506 7.260 4.250 4.233 4.215 4.197 3.931 3.903 3.887 3.860 3.635 3.628 3.608 3.600 3.543 3.536 3.499 3.492 1.988 1.969 1.955 1.948 1.934 1.915 1.875 1.599 1.591 1.573 1.567 1.554 1.533 1.512 1.396 1.378 1.359 1.341 1.316 1.298 1.280 0.969 0.951 0.933
EtO2C
O
Bu
Me
Br
2f
0.96
2.04
1.04
3.02
1.97
1.00
0.98
1.00
0.05
1.95
3.06
2.06
3.17
1.97
3.01
8
6
4
2
0
PPM

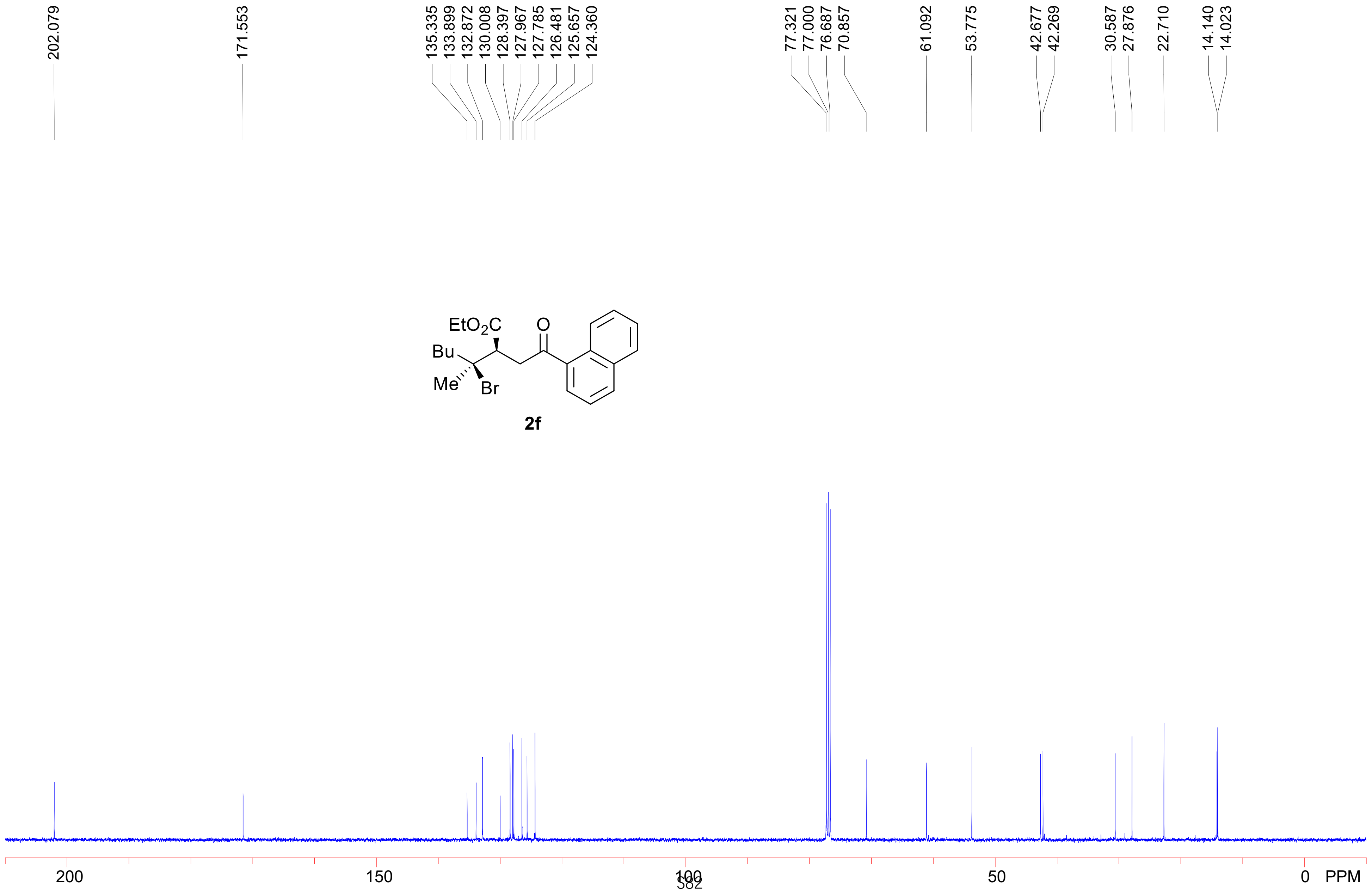
EtO2C
O
Bu
Me
Br
202.079
171.553
135.335
133.899
132.872
130.008
128.397
127.967
127.785
126.481
125.657
124.360
77.321
77.000
76.687
70.857
61.092
53.775
42.677
42.269
30.587
27.876
22.710
14.140
14.023
200
150
100
50
0
PPM

2f

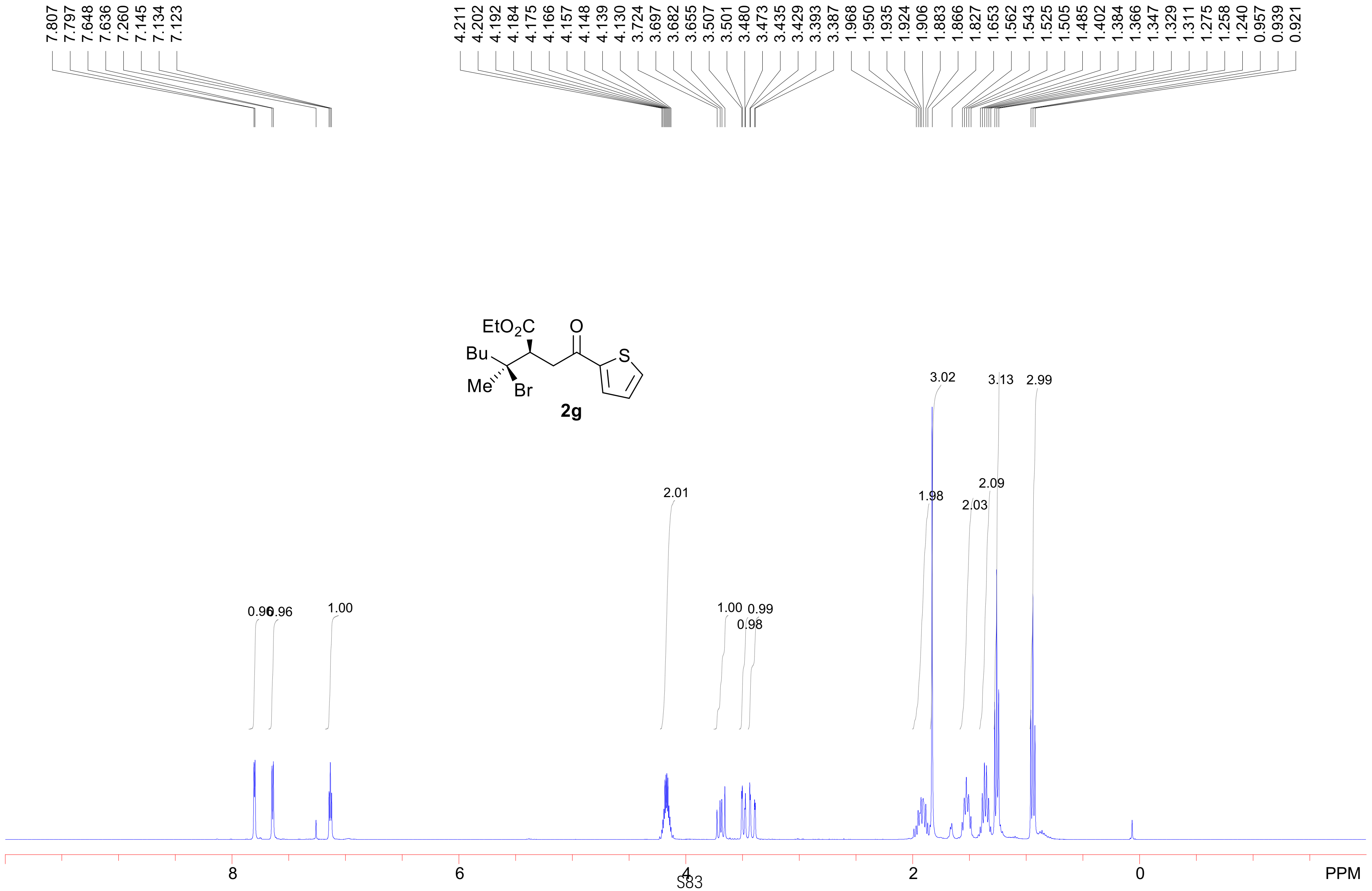
7.807 7.797 7.648 7.636 7.260 7.145 7.134 7.123
4.211 4.202 4.192 4.184 4.175 4.166 4.157 4.148 4.139 4.130 3.724 3.697 3.682 3.655 3.507 3.501 3.480 3.473 3.435 3.429 3.393 3.387 1.968 1.950 1.935 1.924 1.906 1.883 1.866 1.827 1.653 1.562 1.543 1.525 1.505 1.485 1.402 1.384 1.366 1.347 1.329 1.311 1.275 1.258 1.240 0.957 0.939 0.921
EtO_2C
O
S
Bu
Me
Br
2g
0.96 0.96 1.00 2.01 1.00 0.98 0.99 1.98 3.02 2.03 2.09 3.13 2.99
8
6
4
2
0
PPM

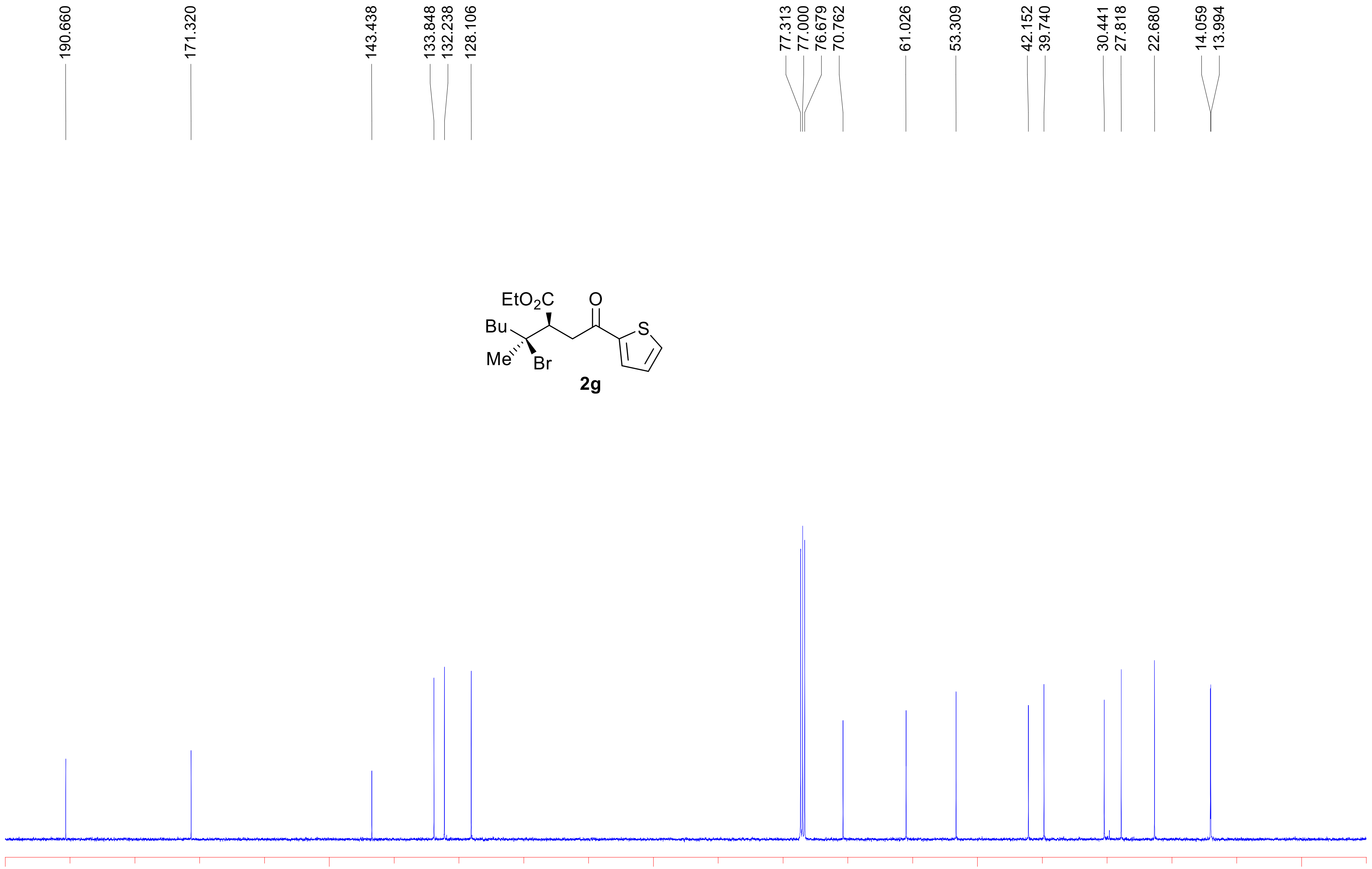
190.660
171.320
143.438
133.848
132.238
128.106
77.313
77.000
76.679
70.762
61.026
53.309
42.152
39.740
30.441
27.818
22.680
14.059
13.994
150
100
50
0
PPM
EtO2C
O
Bu
S
Me
Br

2g

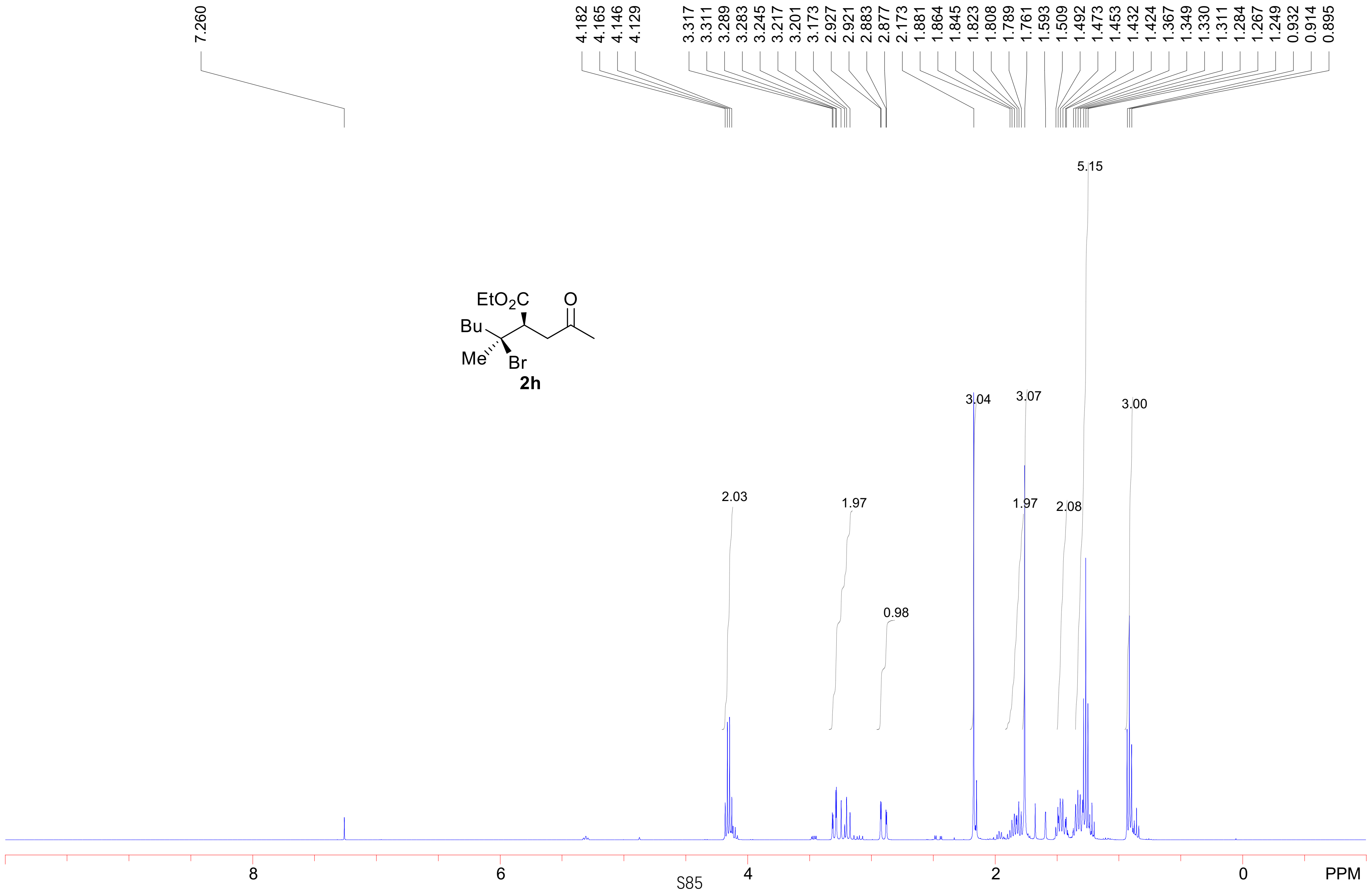
7.260
4.182
4.165
4.146
4.129
3.317
3.311
3.289
3.283
3.245
3.217
3.201
3.173
2.927
2.921
2.883
2.877
2.173
1.881
1.864
1.845
1.823
1.808
1.789
1.761
1.593
1.509
1.492
1.473
1.453
1.432
1.424
1.367
1.349
1.330
1.311
1.284
1.267
1.249
0.932
0.914
0.895
5.15
EtO2C
O
Bu
Me
Br
2h
3.04
3.07
3.00
2.03
1.97
0.98
1.97
2.08
8
6
4
2
0
PPM

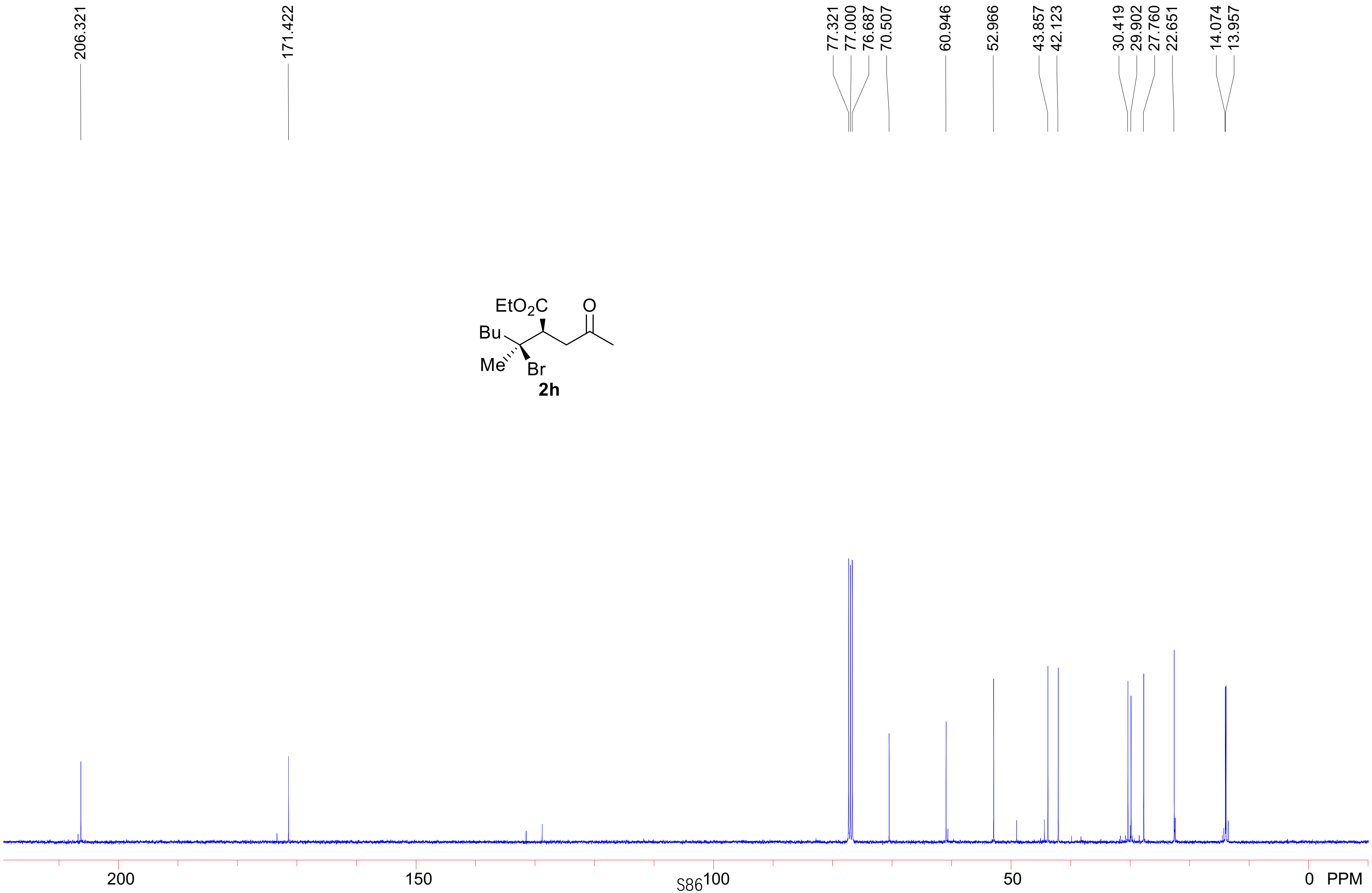
206.321
171.422
77.321
77.000
76.687
70.507
60.946
52.966
43.857
42.123
30.419
29.902
27.760
22.651
14.074
13.957
EtO2C
O
Bu
Me
Br
2h
200
150
100
50
0 PPM

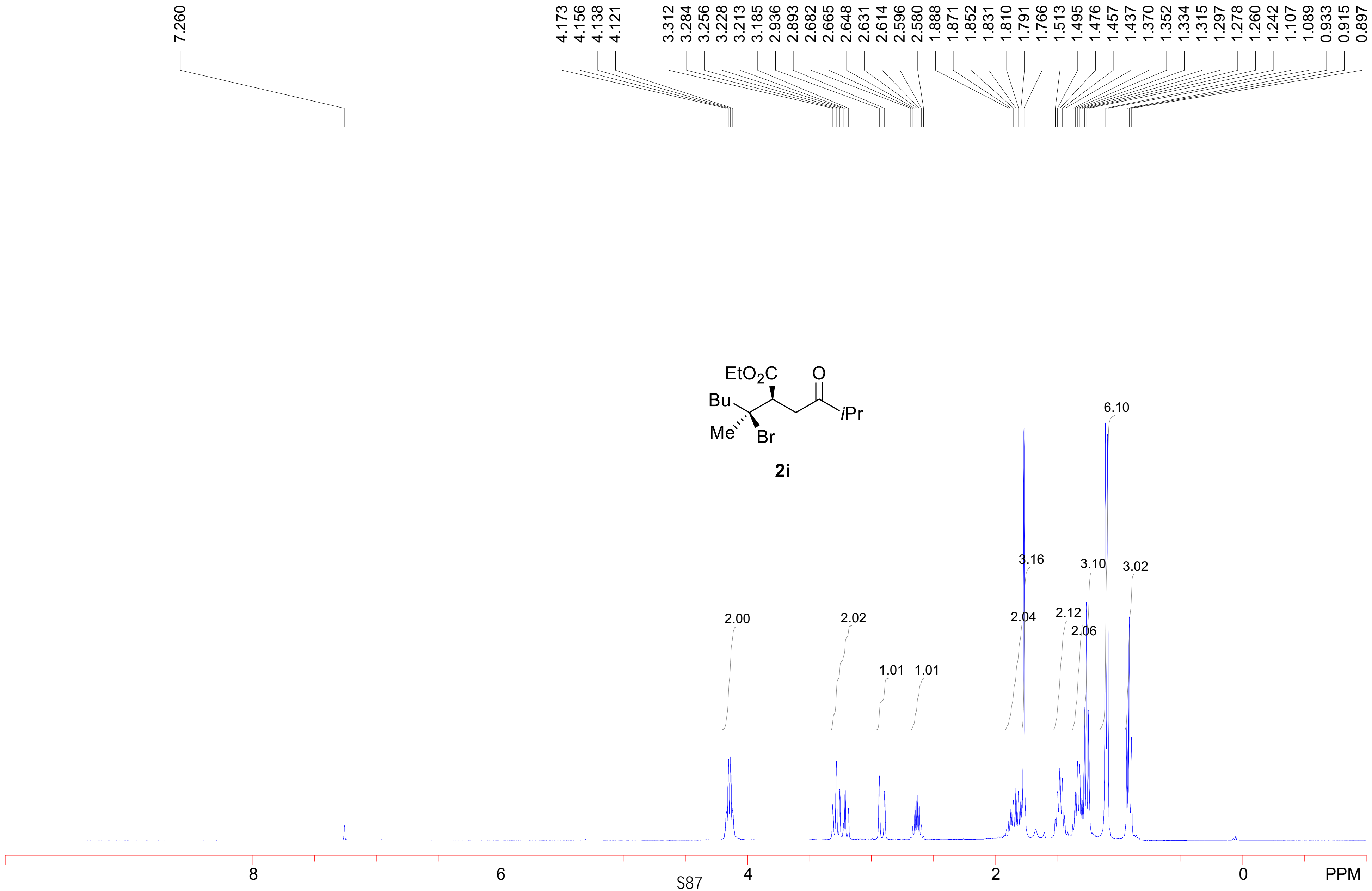
7.260
4.173
4.156
4.138
4.121
3.312
3.284
3.256
3.228
3.213
3.185
2.936
2.893
2.682
2.665
2.648
2.631
2.614
2.596
2.580
1.888
1.871
1.852
1.831
1.810
1.791
1.766
1.513
1.495
1.476
1.457
1.437
1.370
1.352
1.334
1.315
1.297
1.278
1.260
1.242
1.107
1.089
0.933
0.915
0.897
EtO2C
O
Bu
iPr
Me
Br
2i
2.00
2.02
1.01
1.01
2.04
3.16
2.12
2.06
3.10
6.10
3.02
8
6
4
2
0
PPM

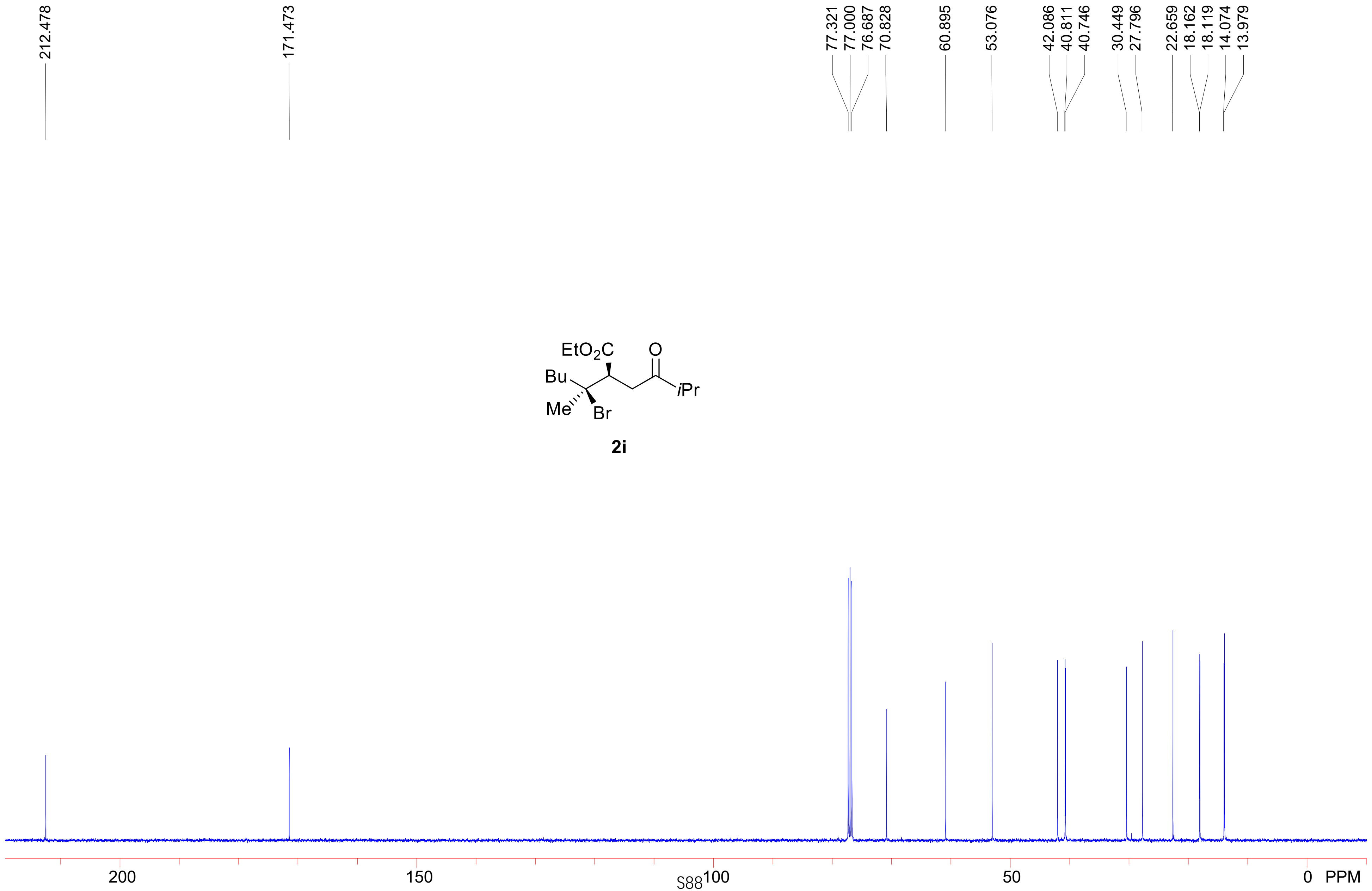
212.478
171.473
77.321
77.000
76.687
70.828
60.895
53.076
42.086
40.811
40.746
30.449
27.796
22.659
18.162
18.119
14.074
13.979
EtO2C
O
Bu
iPr
Me
Br
2i
200
150
100
50
0 PPM

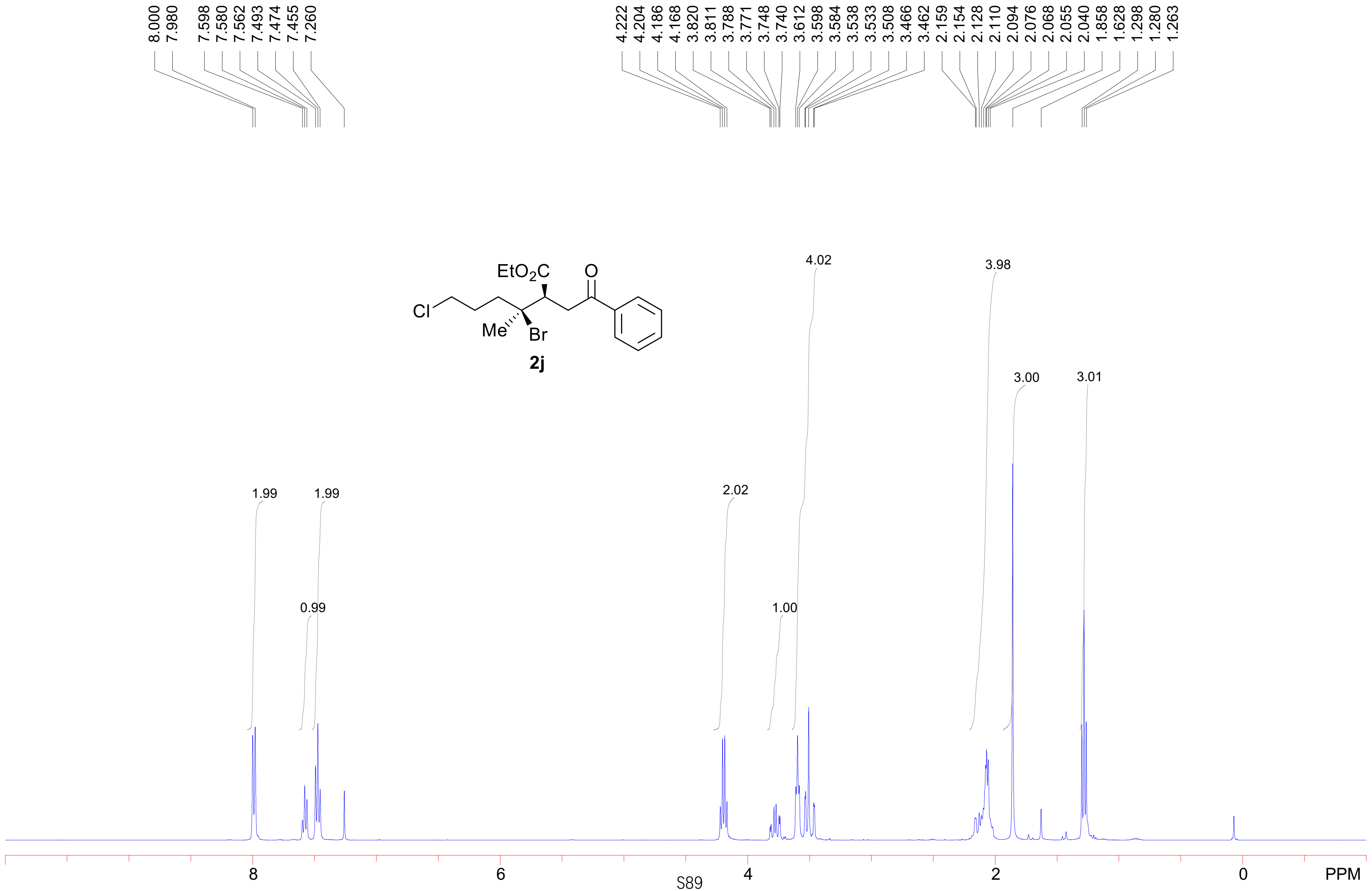
8.000
7.980
7.598
7.580
7.562
7.493
7.474
7.455
7.260
4.222
4.204
4.186
4.168
3.820
3.811
3.788
3.771
3.748
3.740
3.612
3.598
3.584
3.538
3.533
3.508
3.466
3.462
2.159
2.154
2.128
2.110
2.094
2.076
2.068
2.055
2.040
1.858
1.628
1.298
1.280
1.263
EtO2C
O
Cl
Me
Br
2j
1.99
0.99
1.99
2.02
1.00
4.02
3.98
3.00
3.01
8
6
4
2
0
PPM

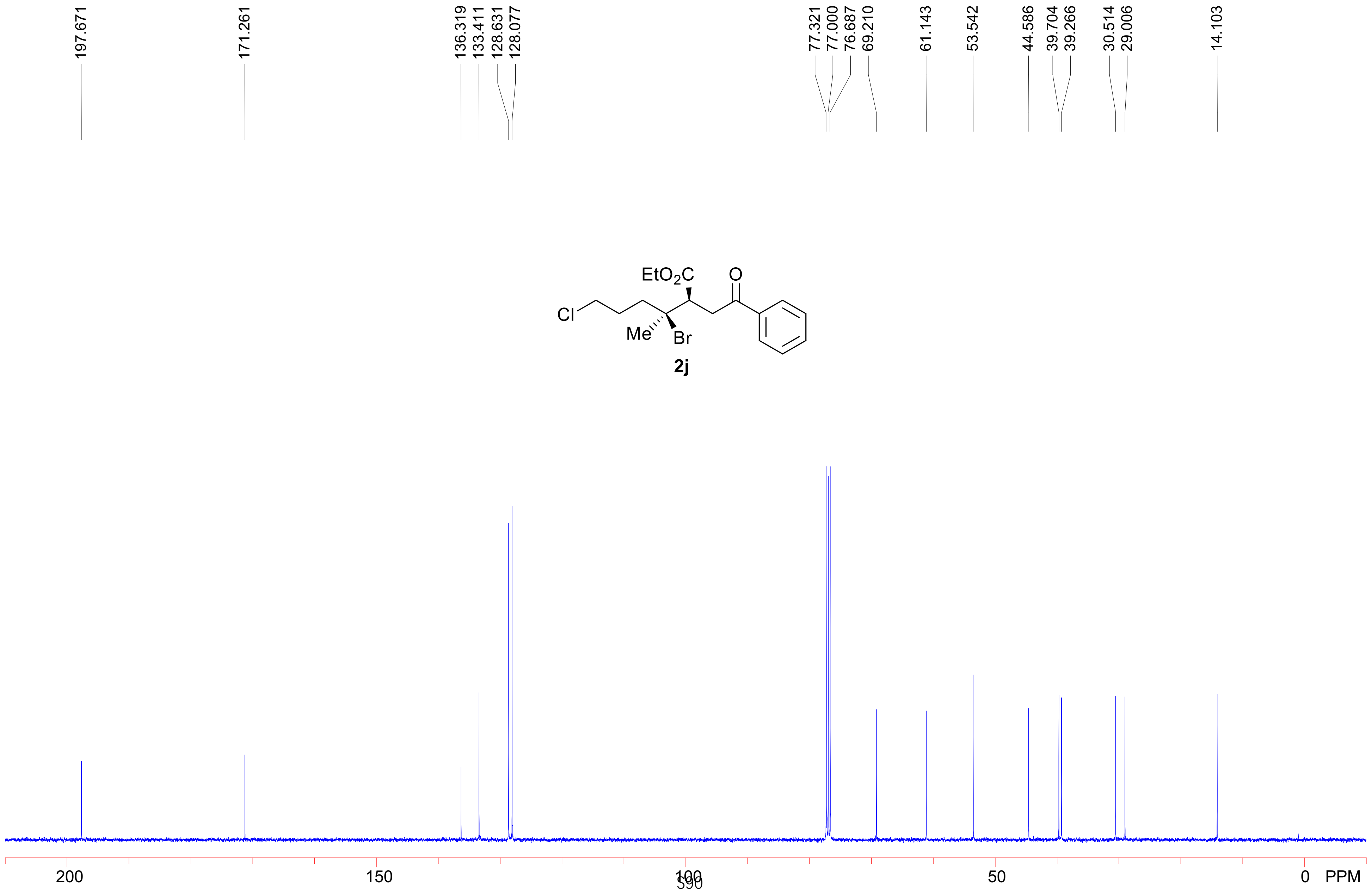
197.671
171.261
136.319
133.411
128.631
128.077
77.321
77.000
76.687
69.210
61.143
53.542
44.586
39.704
39.266
30.514
29.006
14.103
EtO2C
O
Cl
Me
Br
200
150
100
50
0
PPM

2j

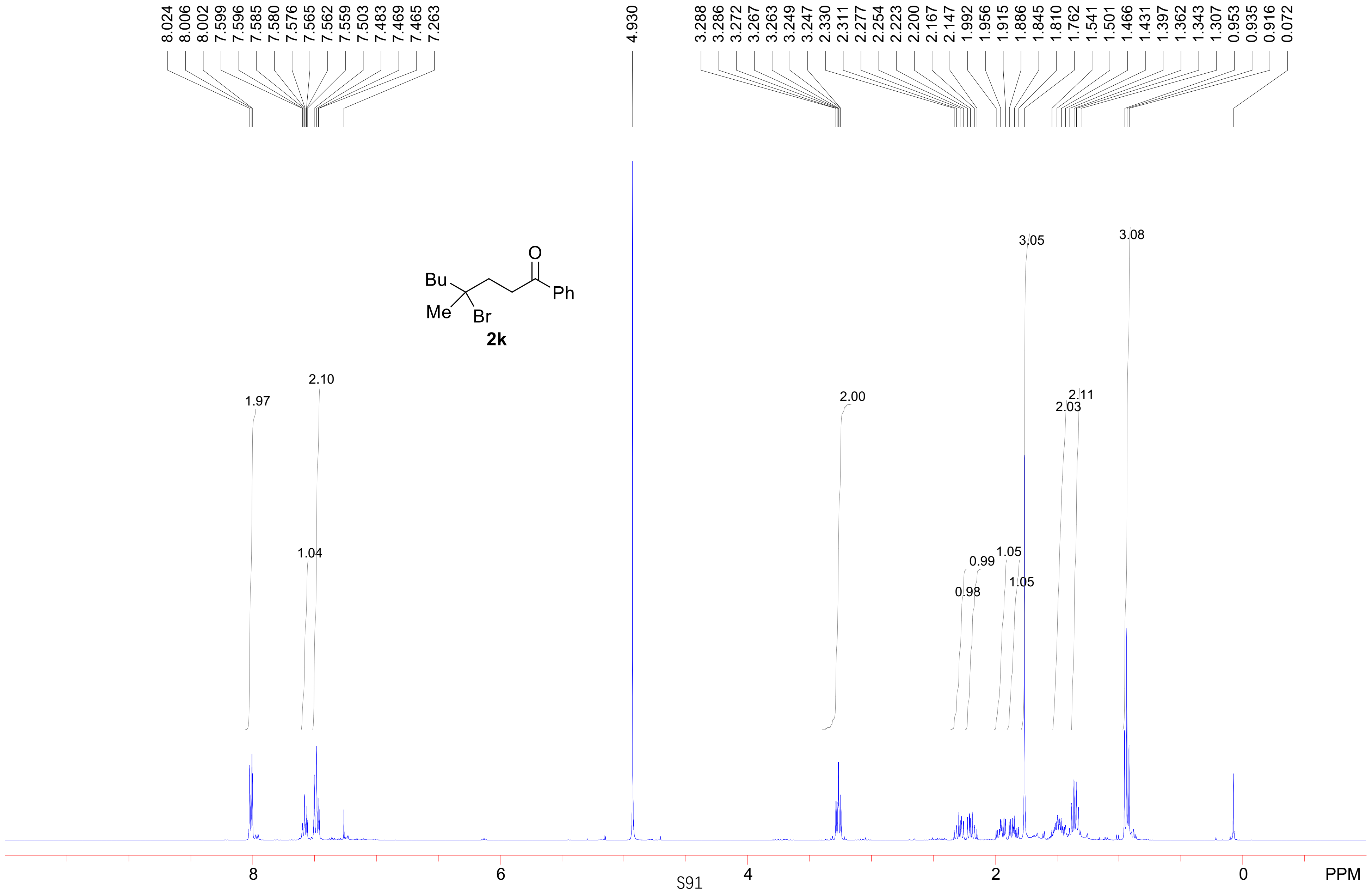

8.024
8.006
8.002
7.599
7.596
7.585
7.580
7.576
7.565
7.562
7.559
7.503
7.483
7.469
7.465
7.263
4.930
3.288
3.286
3.272
3.267
3.263
3.249
3.247
2.330
2.311
2.277
2.254
2.223
2.200
2.167
2.147
1.992
1.956
1.915
1.886
1.845
1.810
1.762
1.541
1.501
1.466
1.431
1.397
1.362
1.343
1.307
0.953
0.935
0.916
0.072
Bu
Me
Br
O
Ph
2k
1.97
2.10
1.04
2.00
0.98
0.99
1.05
1.05
3.05
2.03
2.11
3.08
8
6
4
2
0
PPM

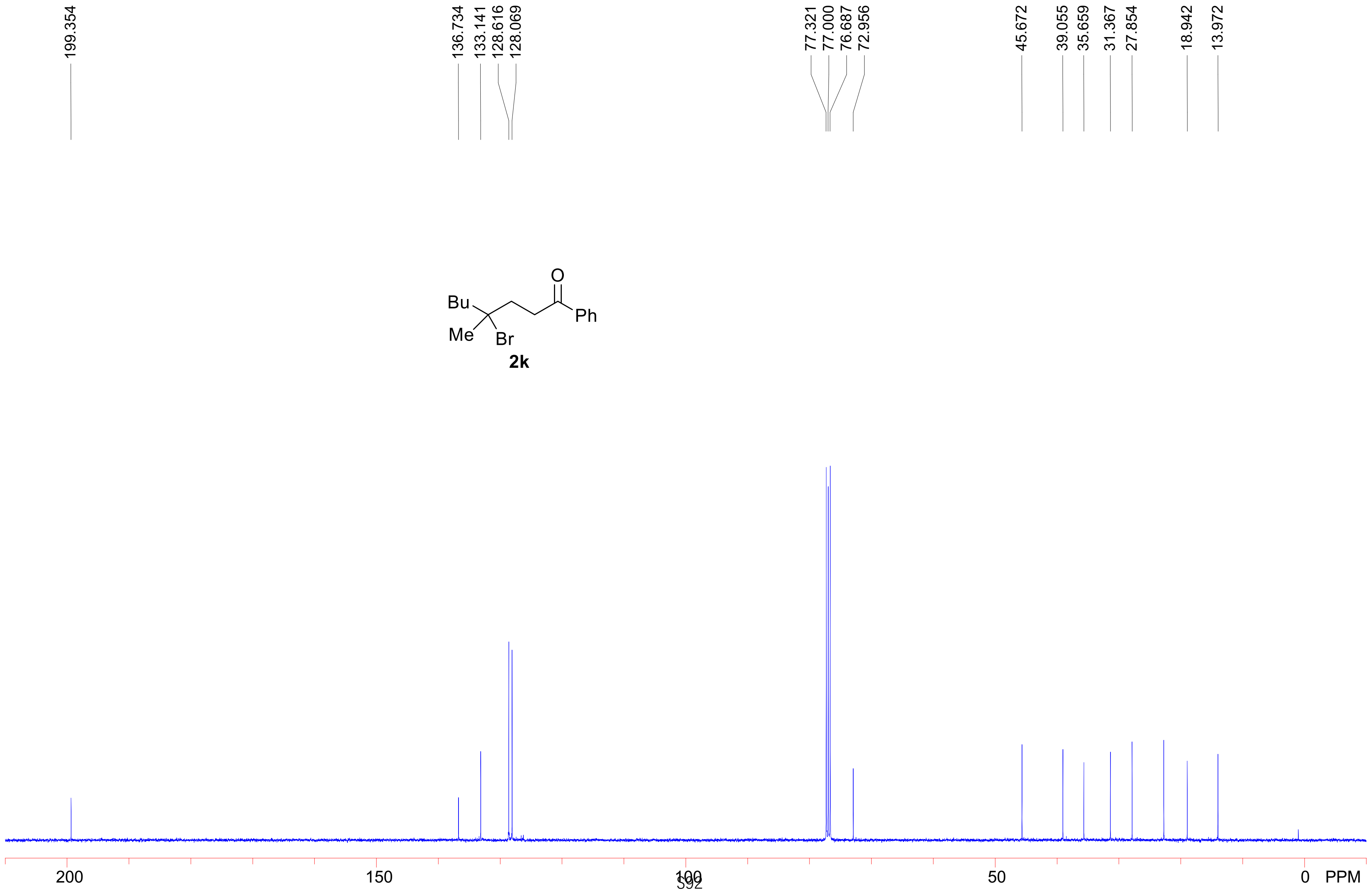
199.354
136.734
133.141
128.616
128.069
77.321
77.000
76.687
72.956
45.672
39.055
35.659
31.367
27.854
18.942
13.972
O
Bu
Ph
Me
Br
2k
200
150
100
50
0
PPM

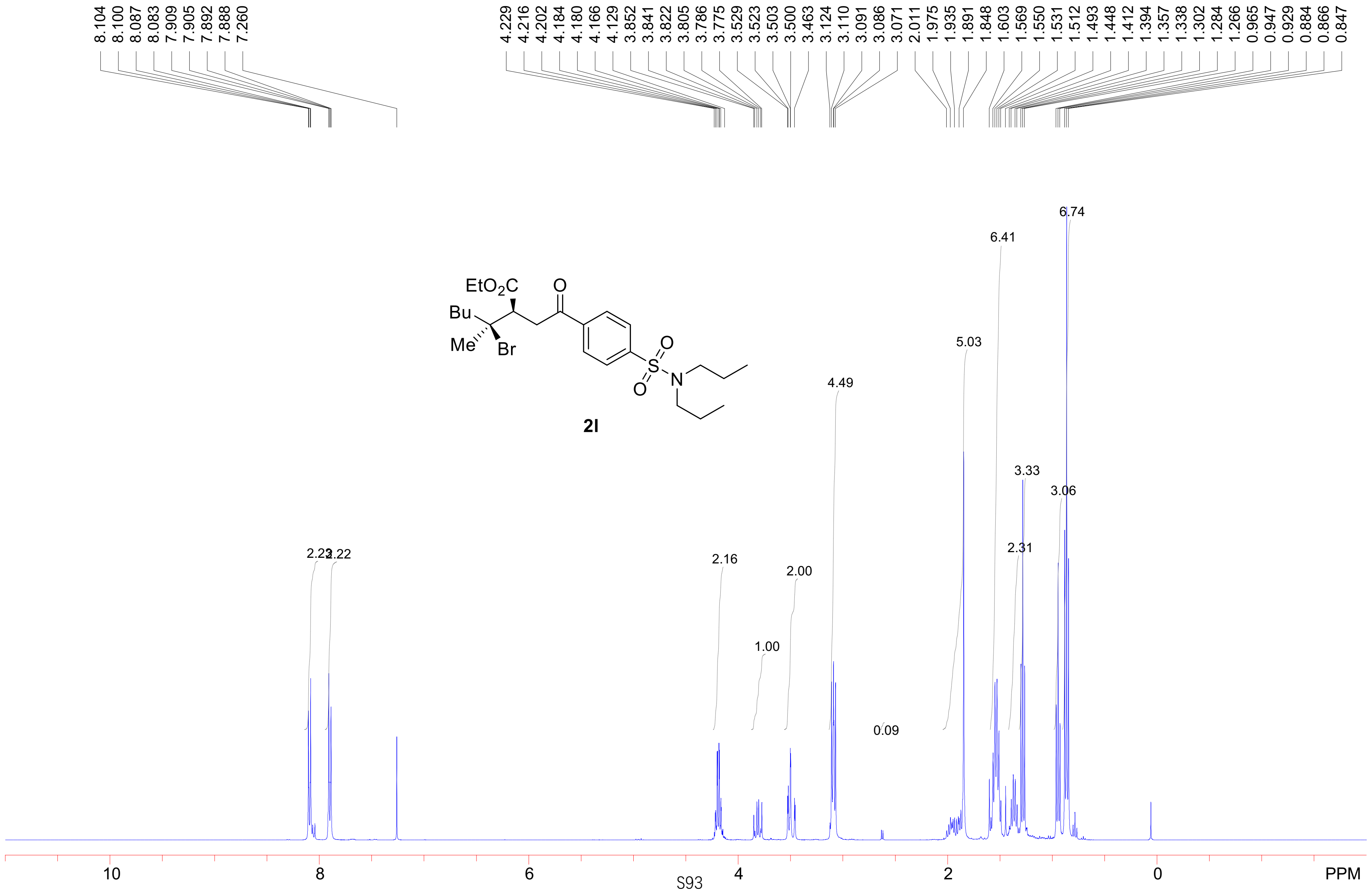
8.104
8.100
8.087
8.083
7.909
7.905
7.892
7.888
7.260
4.229
4.216
4.202
4.184
4.180
4.166
4.129
3.852
3.841
3.822
3.805
3.786
3.775
3.529
3.523
3.503
3.500
3.463
3.124
3.110
3.091
3.086
3.071
2.011
1.975
1.935
1.891
1.848
1.603
1.569
1.550
1.531
1.512
1.493
1.448
1.412
1.394
1.357
1.338
1.302
1.284
1.266
0.965
0.947
0.929
0.884
0.866
0.847
EtO2C
O
Bu
Me
Br
S
O
O
N
2l
2.23
2.22
2.16
1.00
2.00
4.49
0.09
5.03
6.41
2.31
3.33
3.06
6.74
10
8
6
4
2
0
PPM

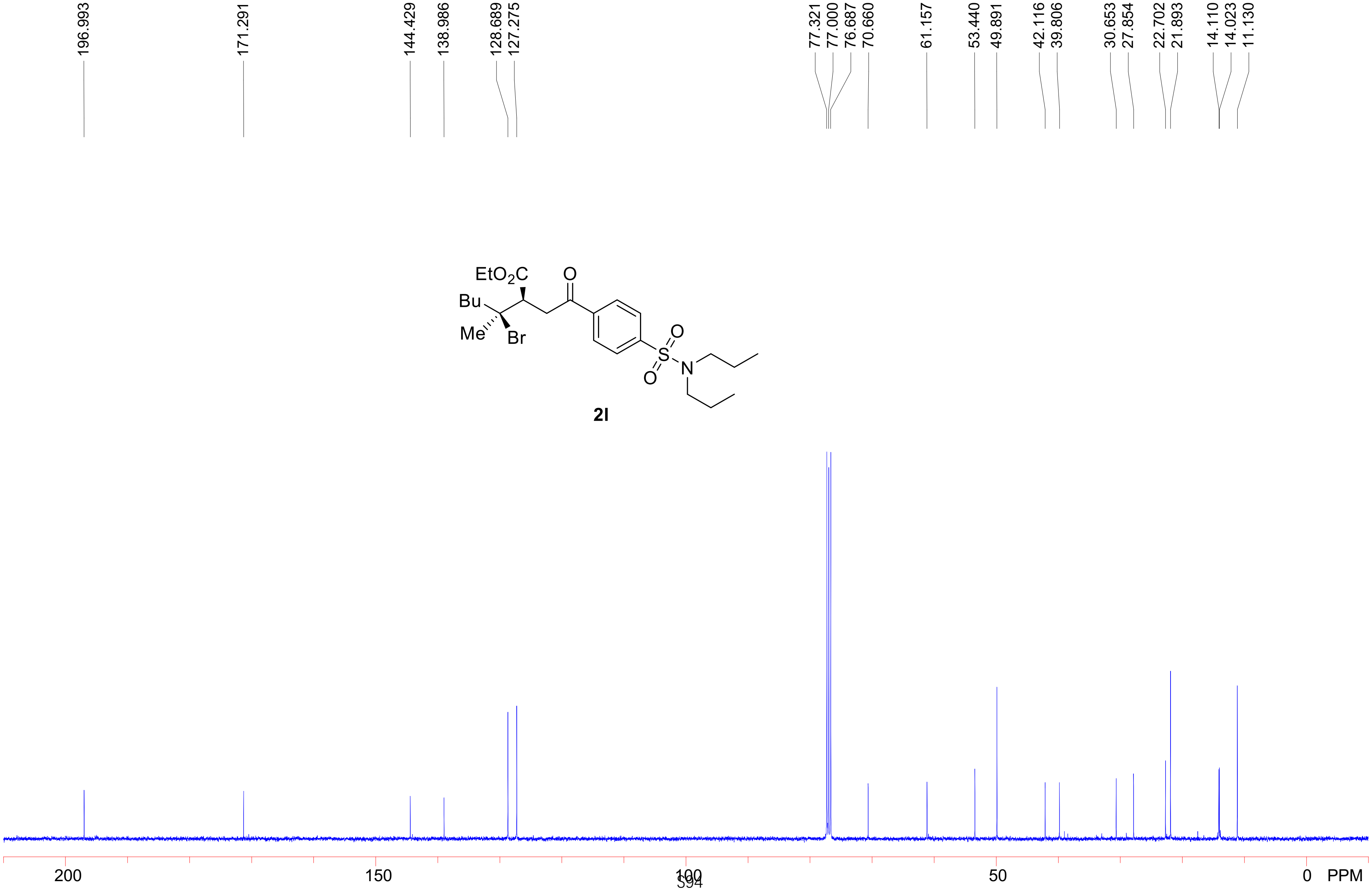
196.993
171.291
144.429
138.986
128.689
127.275
77.321
77.000
76.687
70.660
61.157
53.440
49.891
42.116
39.806
30.653
27.854
22.702
21.893
14.110
14.023
11.130
EtO2C
O
Bu
Me
Br
S
O
O
N
200
150
100
50
0
PPM

2l

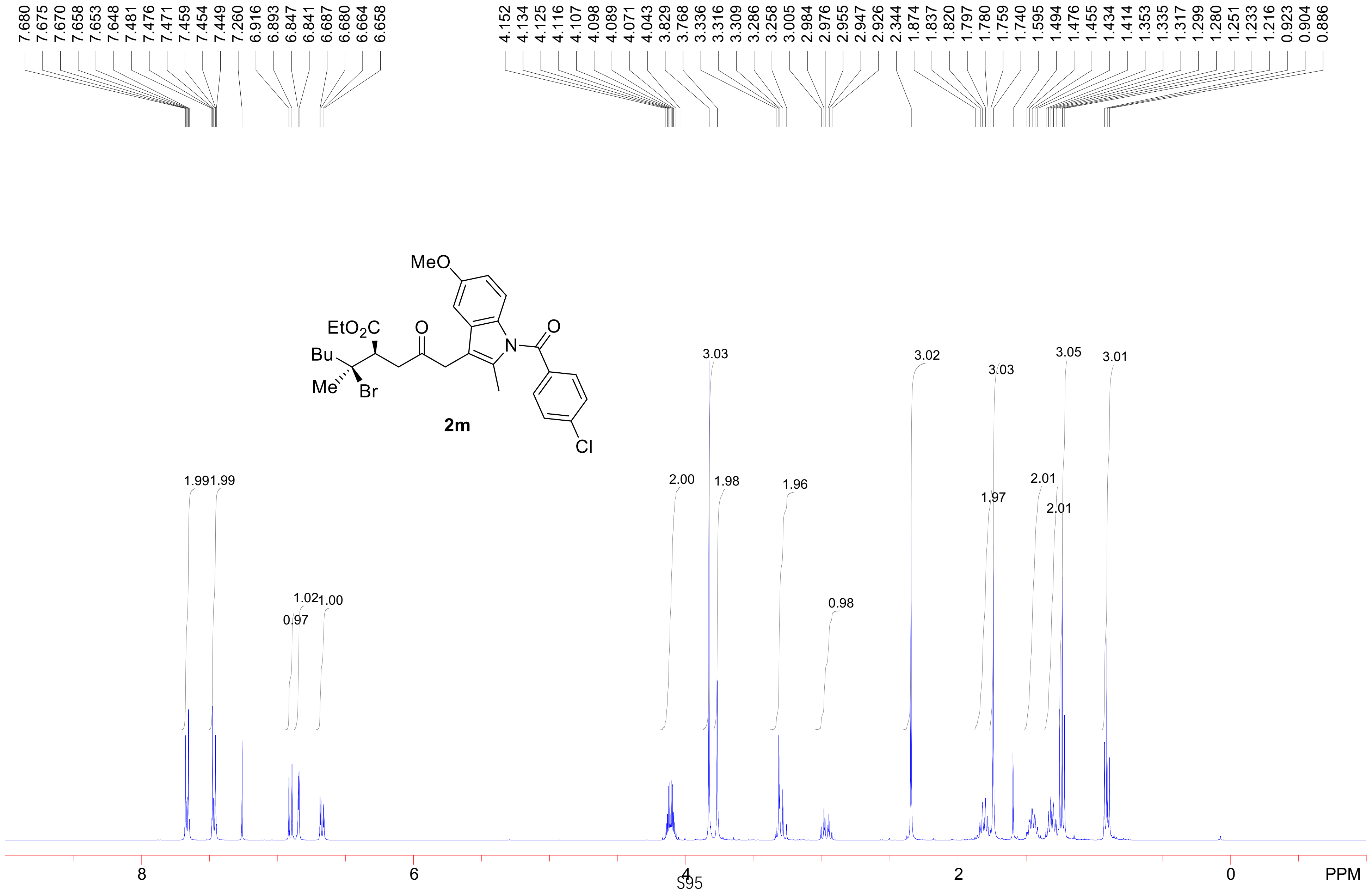
7.680 7.675 7.670 7.658 7.653 7.648 7.481 7.476 7.471 7.459 7.454 7.449 7.260 6.916 6.893 6.847 6.841 6.687 6.680 6.664 6.658
4.152 4.134 4.125 4.116 4.107 4.098 4.089 4.071 4.043 3.829 3.768 3.336 3.316 3.309 3.286 3.258 3.005 2.984 2.976 2.955 2.947 2.926 2.344 1.874 1.837 1.820 1.797 1.780 1.759 1.740 1.595 1.494 1.476 1.455 1.434 1.414 1.353 1.335 1.317 1.299 1.280 1.251 1.233 1.216 0.923 0.904 0.886
MeO
EtO2C
O
O
N
Bu
Me
Br
Cl
2m
1.99 1.99
0.97
1.02 1.00
2.00
3.03
1.98
1.96
0.98
3.02
3.03
1.97
2.01
2.01
3.05
3.01
8
6
4
2
0
PPM

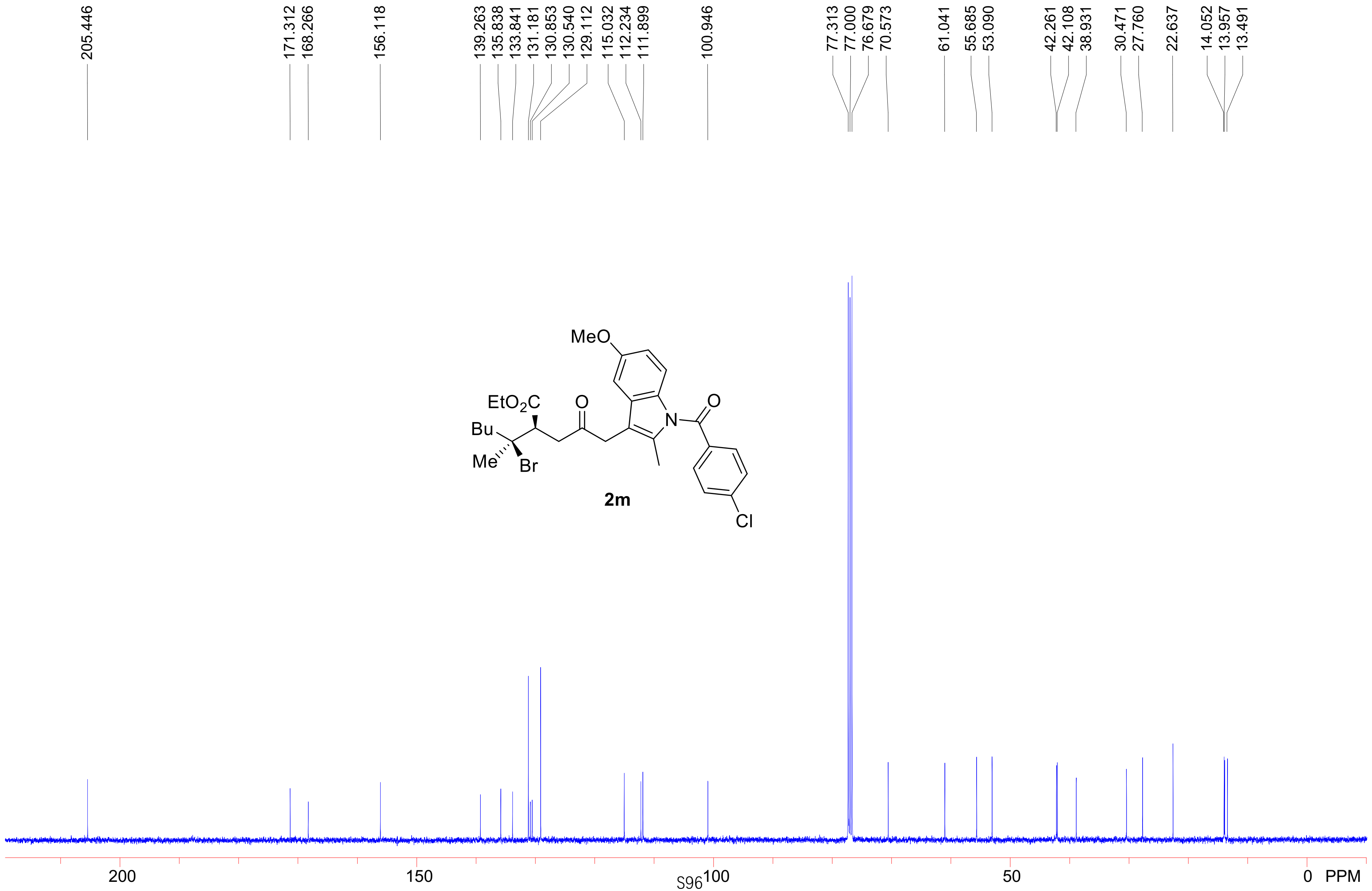
205.446
171.312
168.266
156.118
139.263
135.838
133.841
131.181
130.853
130.540
129.112
115.032
112.234
111.899
100.946
77.313
77.000
76.679
70.573
61.041
55.685
53.090
42.261
42.108
38.931
30.471
27.760
22.637
14.052
13.957
13.491
MeO
EtO_2C
O
O
N
Bu
Me
Br
Cl
2m
200
150
100
50
0 PPM

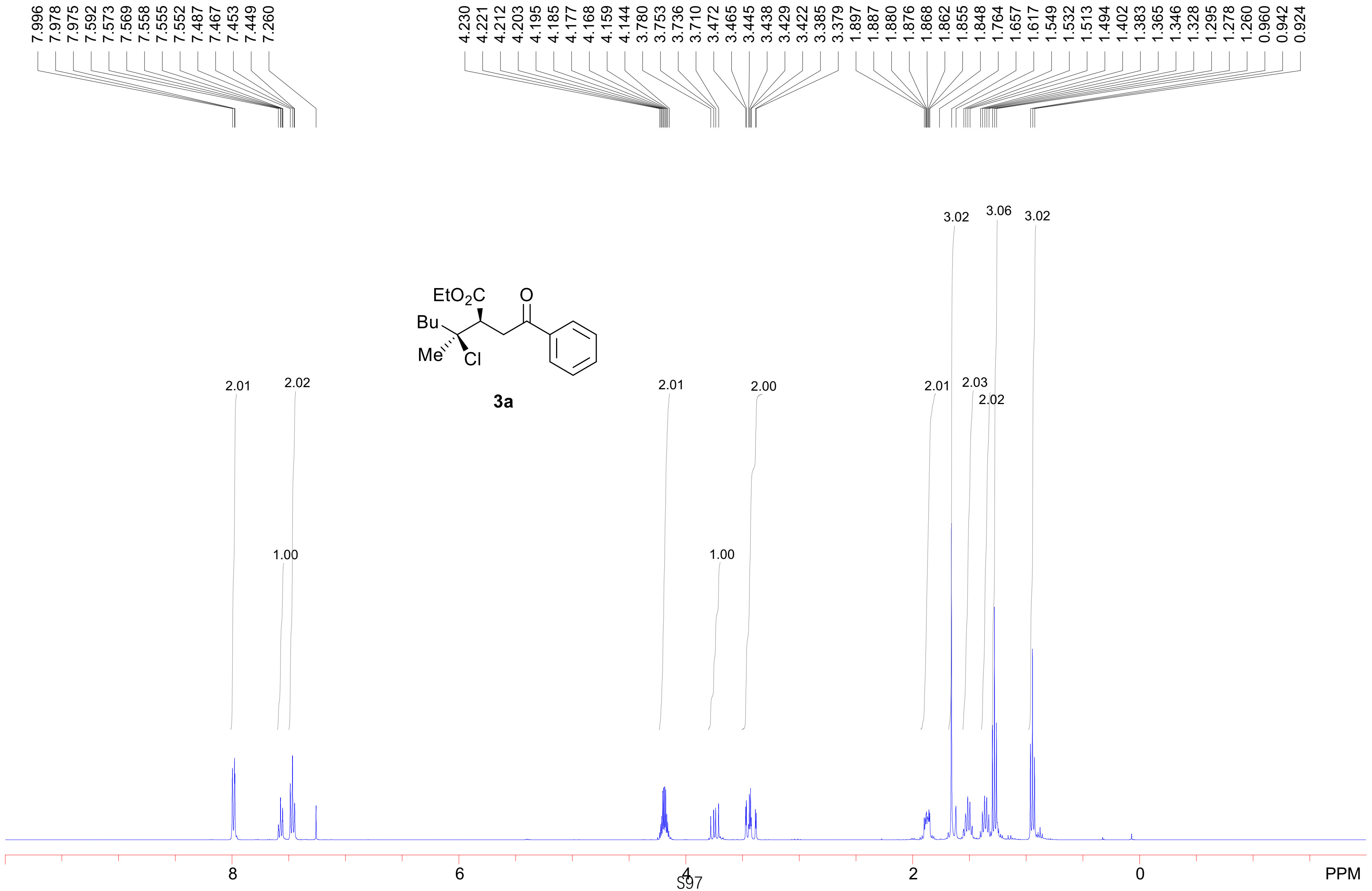
7.996 7.978 7.975 7.592 7.573 7.569 7.558 7.555 7.552 7.487 7.467 7.453 7.449 7.260
4.230 4.221 4.212 4.203 4.195 4.185 4.177 4.168 4.159 4.144 3.780 3.753 3.736 3.710 3.472 3.465 3.445 3.438 3.429 3.422 3.385 3.379
1.897 1.887 1.880 1.876 1.868 1.862 1.855 1.848 1.764 1.657 1.617 1.549 1.532 1.513 1.494 1.402 1.383 1.365 1.346 1.328 1.295 1.278 1.260 0.960 0.942 0.924
3.02
3.06
3.02
EtO2C
O
Bu
Me
Cl
3a
2.01
2.02
1.00
2.01
1.00
2.00
2.01
2.03
2.02
8
6
4
2
0
PPM

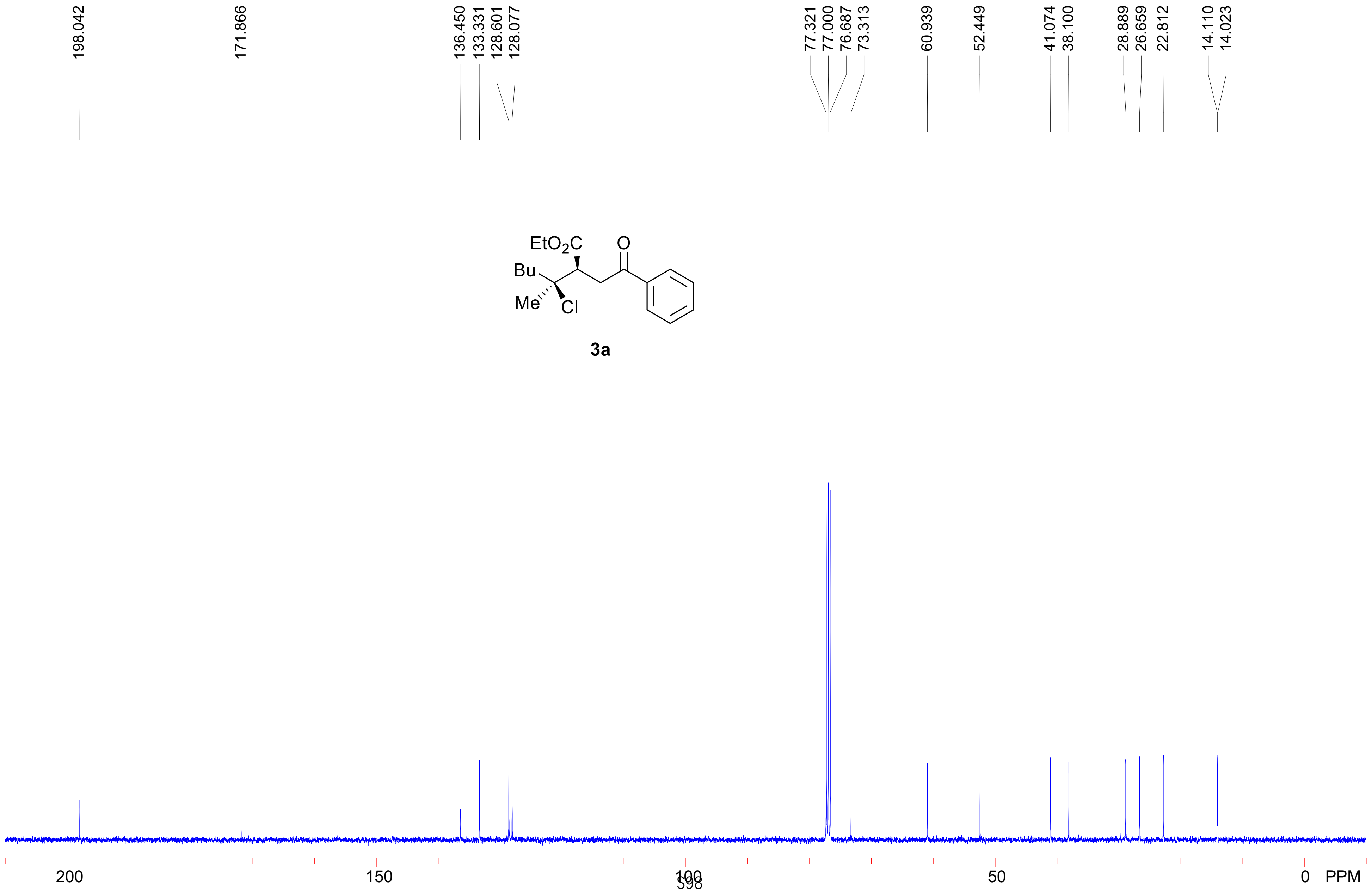
198.042
171.866
136.450
133.331
128.601
128.077
77.321
77.000
76.687
73.313
60.939
52.449
41.074
38.100
28.889
26.659
22.812
14.110
14.023
EtO2C
O
Bu
Me
Cl
3a
200
150
100
50
0
PPM

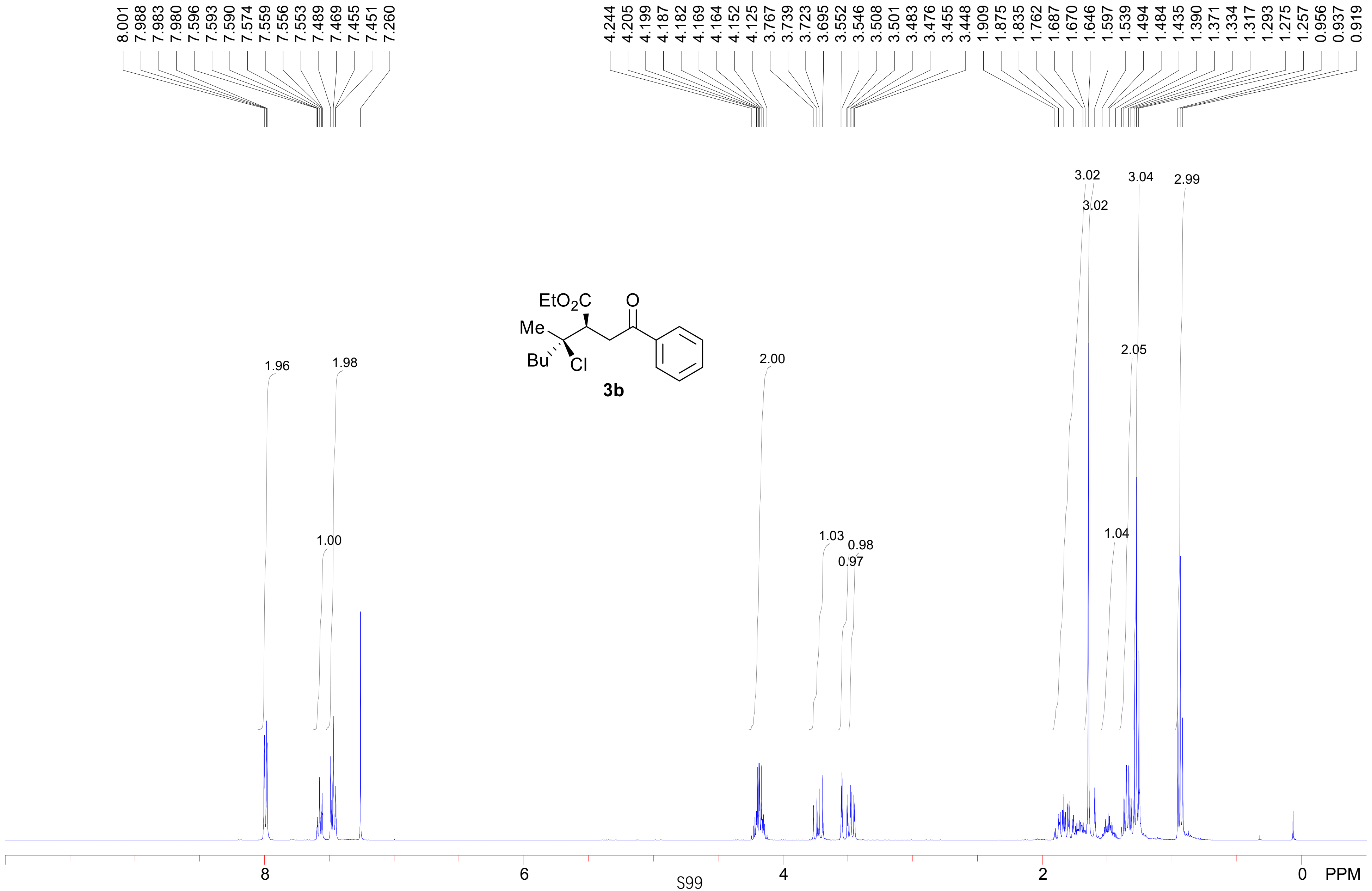
8.001
7.988
7.983
7.980
7.596
7.593
7.590
7.574
7.559
7.556
7.553
7.489
7.469
7.455
7.451
7.260
4.244
4.205
4.199
4.187
4.182
4.169
4.164
4.152
4.125
3.767
3.739
3.723
3.695
3.552
3.546
3.508
3.501
3.483
3.476
3.455
3.448
1.909
1.875
1.835
1.762
1.687
1.670
1.646
1.597
1.539
1.494
1.484
1.435
1.390
1.371
1.334
1.317
1.293
1.275
1.257
0.956
0.937
0.919
EtO2C
O
Me
Bu
Cl
3b
1.96
1.98
1.00
2.00
1.03
0.98
0.97
3.02
3.02
3.04
2.99
2.05
1.04
8
6
4
2
0
PPM

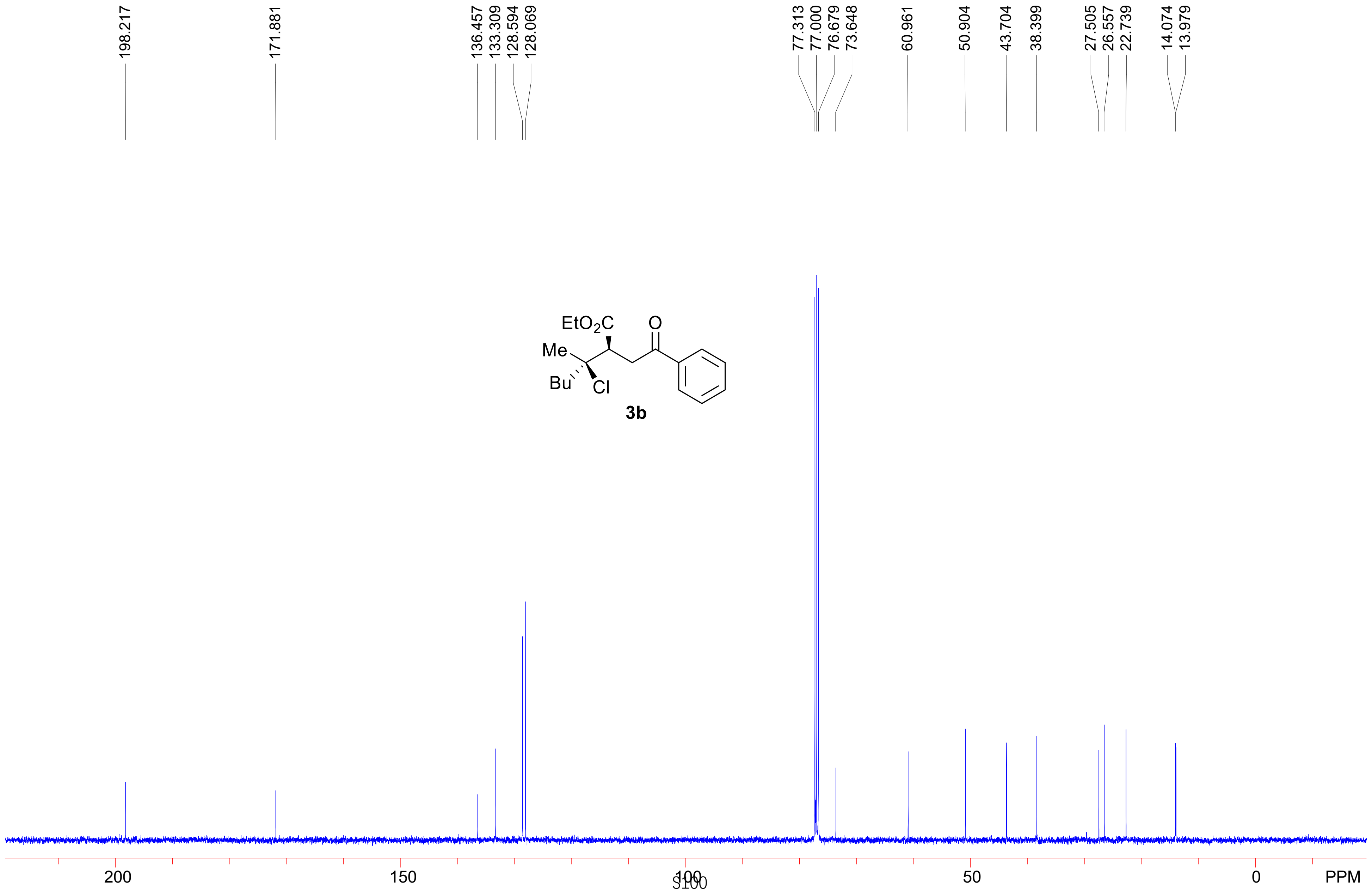
198.217
171.881
136.457
133.309
128.594
128.069
77.313
77.000
76.679
73.648
60.961
50.904
43.704
38.399
27.505
26.557
22.739
14.074
13.979
EtO2C
O
Me
Bu
Cl
3b
200
150
100
50
0
PPM

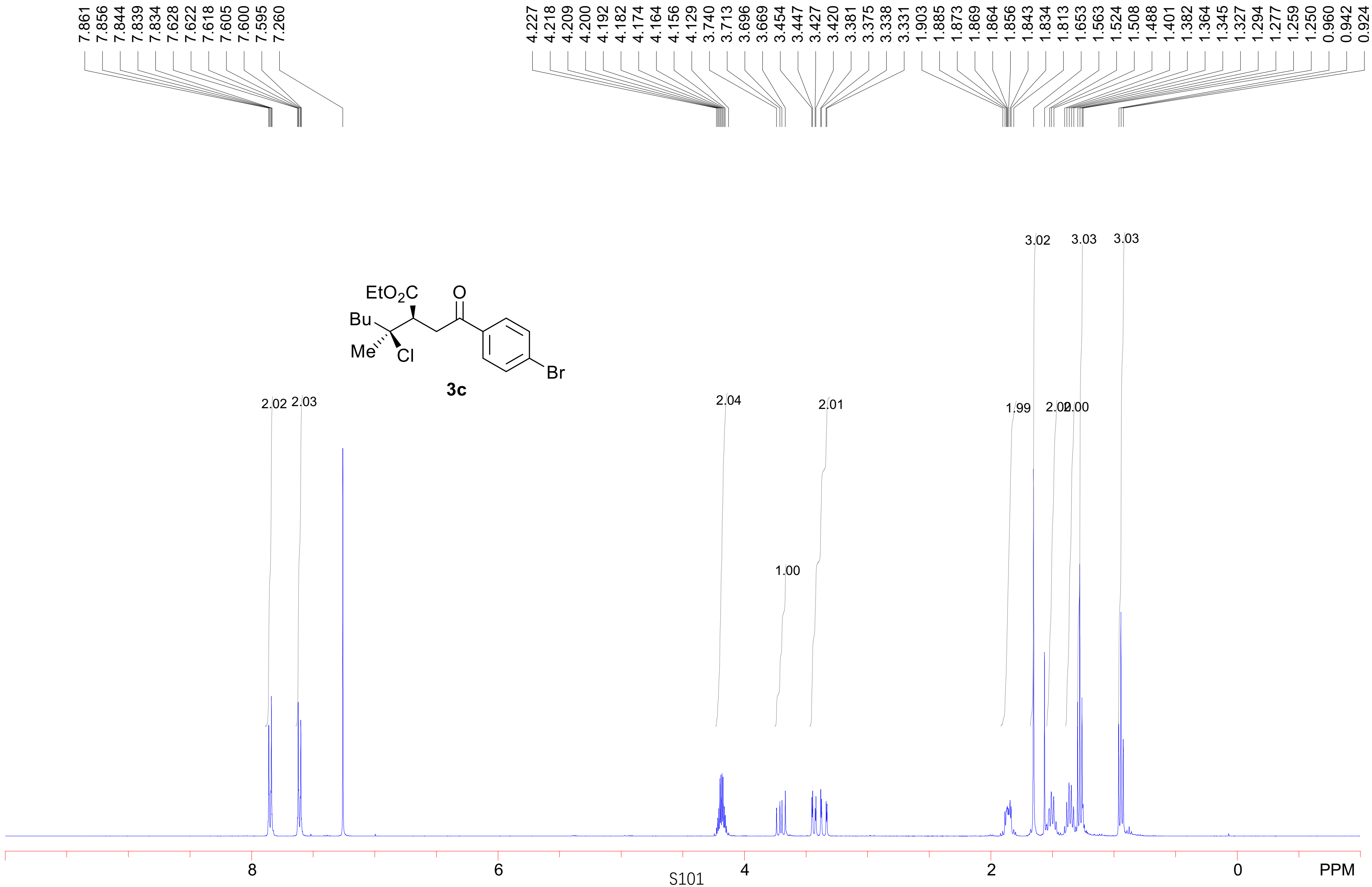

7.861
7.856
7.844
7.839
7.834
7.628
7.622
7.618
7.605
7.600
7.595
7.260
4.227
4.218
4.209
4.200
4.192
4.182
4.174
4.164
4.156
4.129
3.740
3.713
3.696
3.669
3.454
3.447
3.427
3.420
3.381
3.375
3.338
3.331
1.903
1.885
1.873
1.869
1.864
1.856
1.843
1.834
1.813
1.653
1.563
1.524
1.508
1.488
1.401
1.382
1.364
1.345
1.327
1.294
1.277
1.259
1.250
0.960
0.942
0.924
EtO2C
O
Bu
Me
Cl
Br
3c
2.02
2.03
2.04
1.00
2.01
1.99
3.02
2.00
2.00
3.03
3.03
8
6
4
2
0
PPM

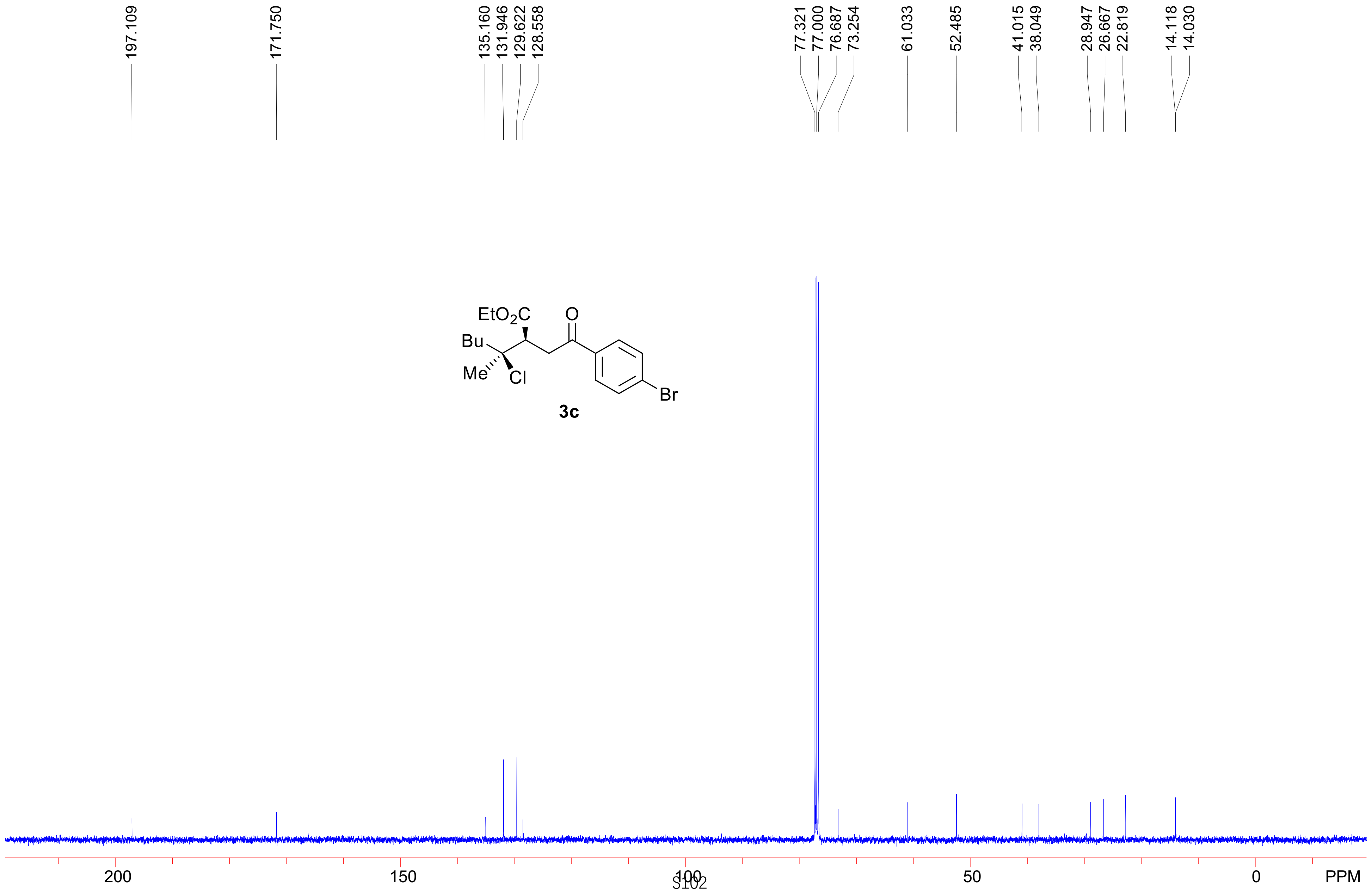
197.109
171.750
135.160
131.946
129.622
128.558
77.321
77.000
76.687
73.254
61.033
52.485
41.015
38.049
28.947
26.667
22.819
14.118
14.030
EtO2C
O
Bu
Me
Cl
Br
3c
200
150
100
50
0
PPM

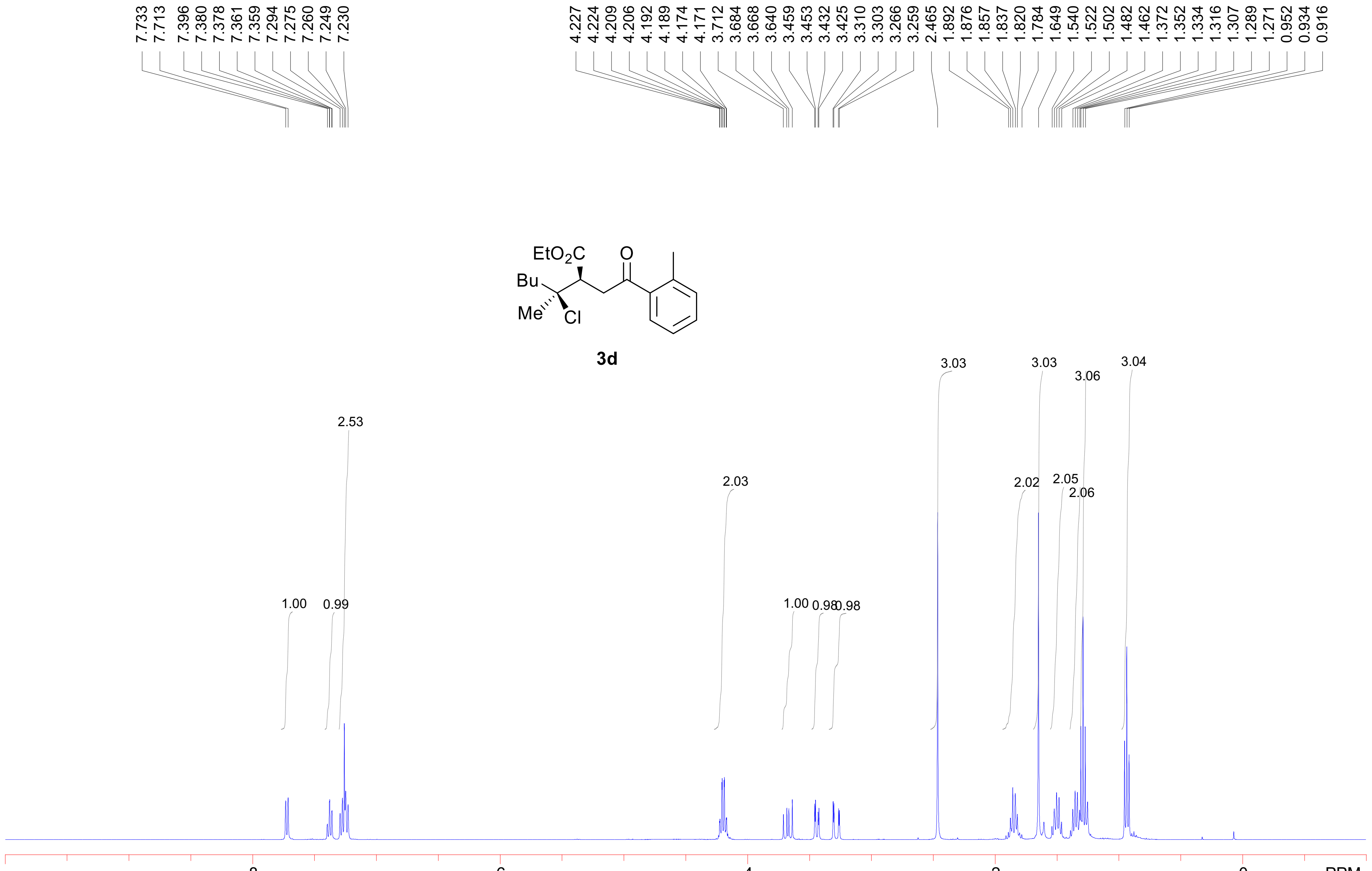
7.733
7.713
7.396
7.380
7.378
7.361
7.359
7.294
7.275
7.260
7.249
7.230
4.227
4.224
4.209
4.206
4.192
4.189
4.174
4.171
3.712
3.684
3.668
3.640
3.459
3.453
3.432
3.425
3.310
3.303
3.266
3.259
2.465
1.892
1.876
1.857
1.837
1.820
1.784
1.649
1.540
1.522
1.502
1.482
1.462
1.372
1.352
1.334
1.316
1.307
1.289
1.271
0.952
0.934
0.916
EtO2C
O
Bu
Me
Cl
3d
1.00
0.99
2.53
2.03
1.00
0.98
0.98
3.03
2.02
3.03
2.05
3.06
2.06
3.04
8
6
4
2
0
PPM

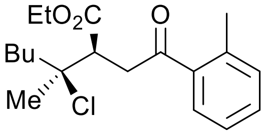

3d

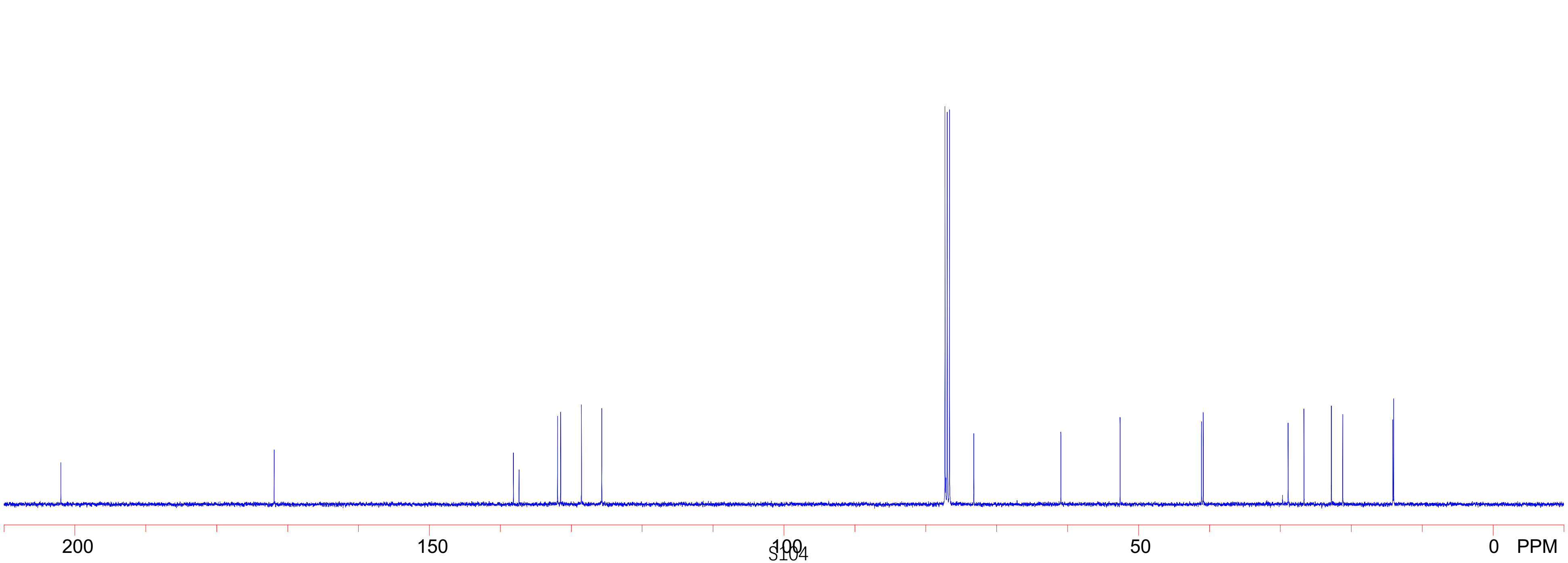

201.977
171.895
138.148
137.354
131.932
131.495
128.558
125.701
77.321
77.000
76.687
73.254
60.968
52.602
41.125
40.877
28.896
26.659
22.797
21.194
14.125
14.016
200
150
100
50
0
PPM

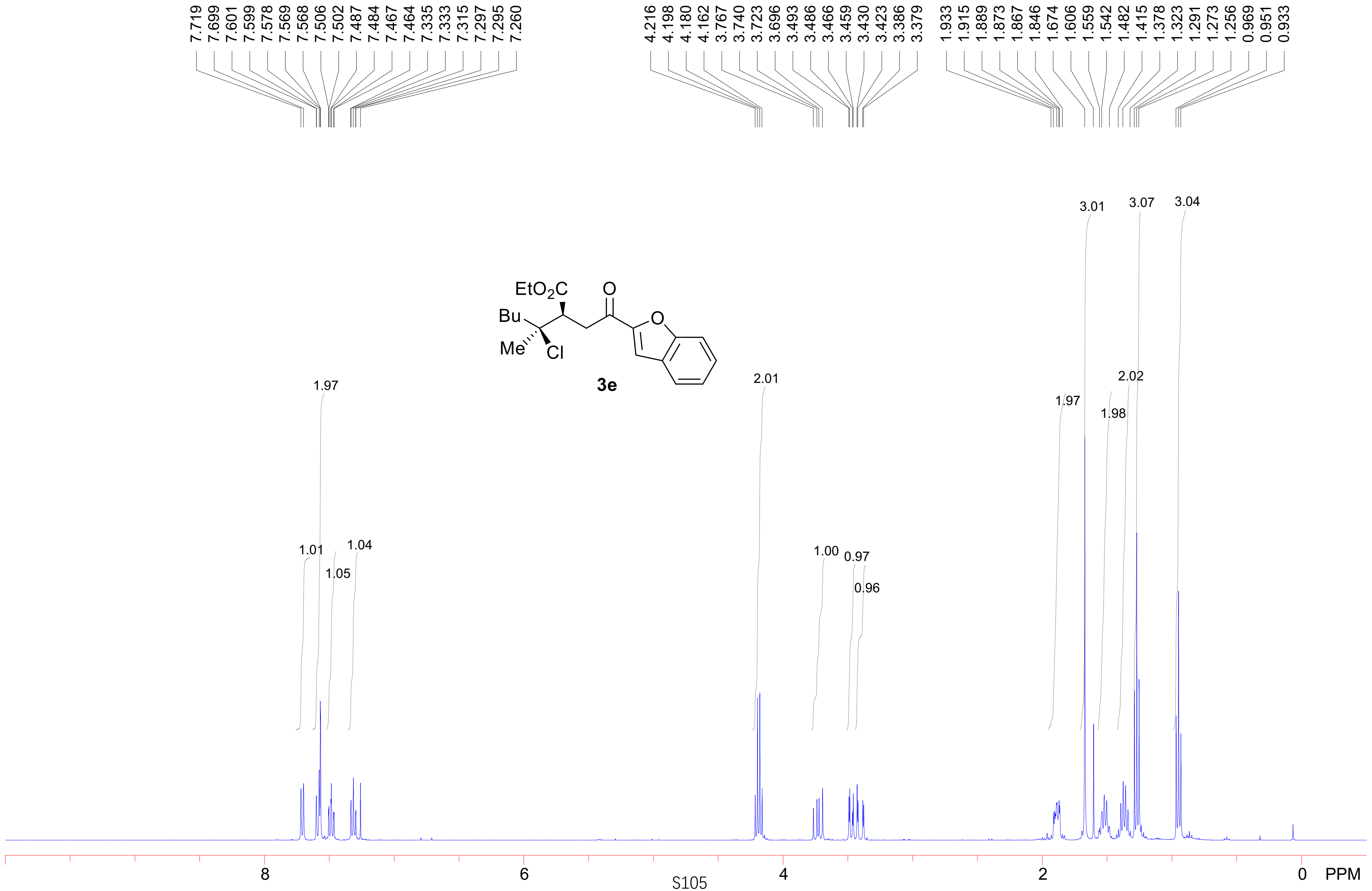
7.719 7.699 7.601 7.599 7.578 7.569 7.568 7.506 7.502 7.487 7.484 7.467 7.464 7.335 7.333 7.315 7.297 7.295 7.260
4.216 4.198 4.180 4.162 3.767 3.740 3.723 3.696 3.493 3.486 3.466 3.459 3.430 3.423 3.386 3.379
1.933 1.915 1.889 1.873 1.867 1.846 1.674 1.606 1.559 1.542 1.482 1.415 1.378 1.323 1.291 1.273 1.256 0.969 0.951 0.933
EtO2C
O
Bu
Me
Cl
O
3e
1.97
1.01
1.05
1.04
2.01
1.00
0.97
0.96
1.97
3.01
1.98
2.02
3.07
3.04
8
6
4
2
0
PPM

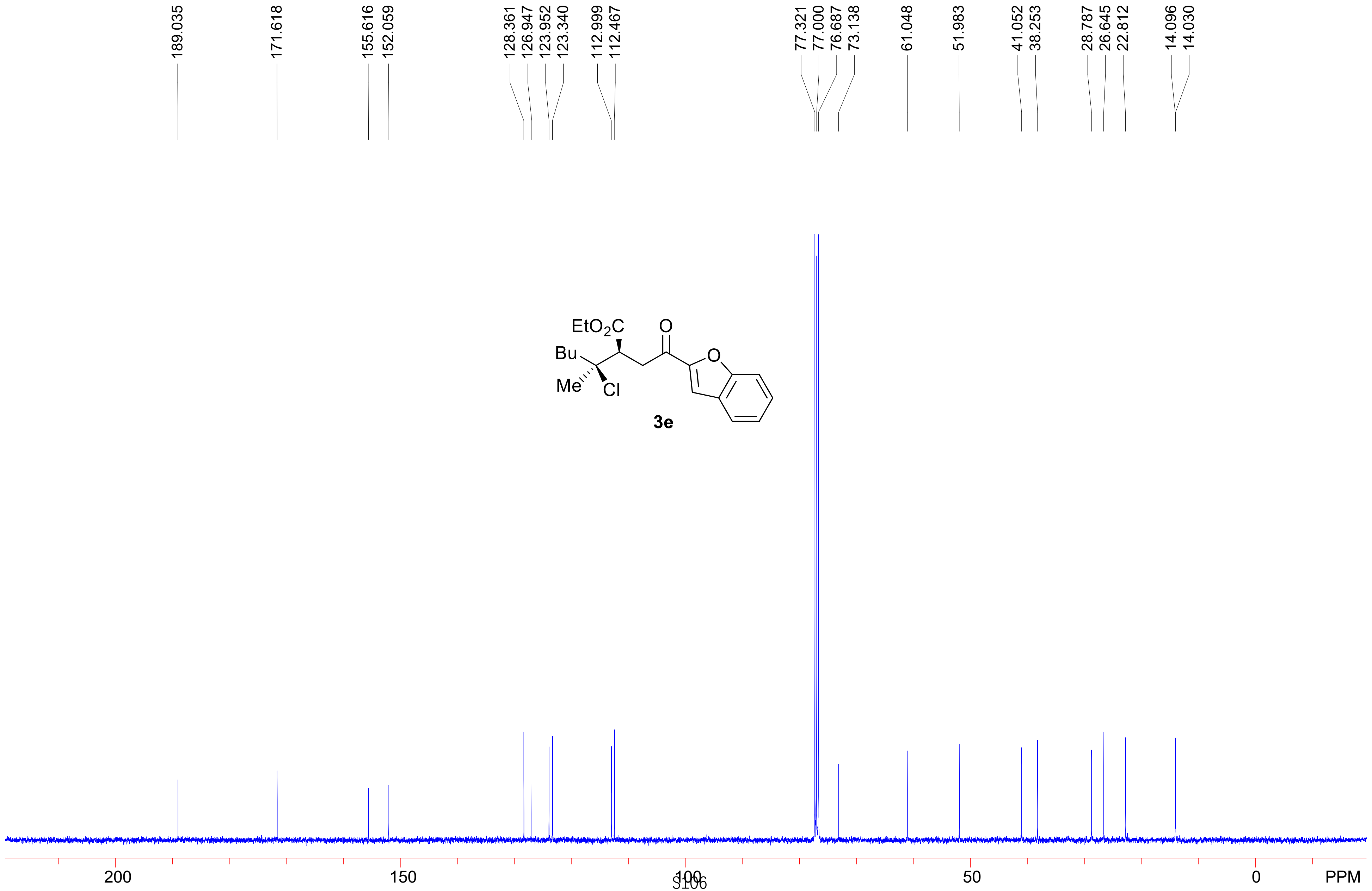
189.035
171.618
155.616
152.059
128.361
126.947
123.952
123.340
112.999
112.467
77.321
77.000
76.687
73.138
61.048
51.983
41.052
38.253
28.787
26.645
22.812
14.096
14.030
EtO2C
O
Bu
O
Me
Cl
3e
200
150
100
50
0
PPM

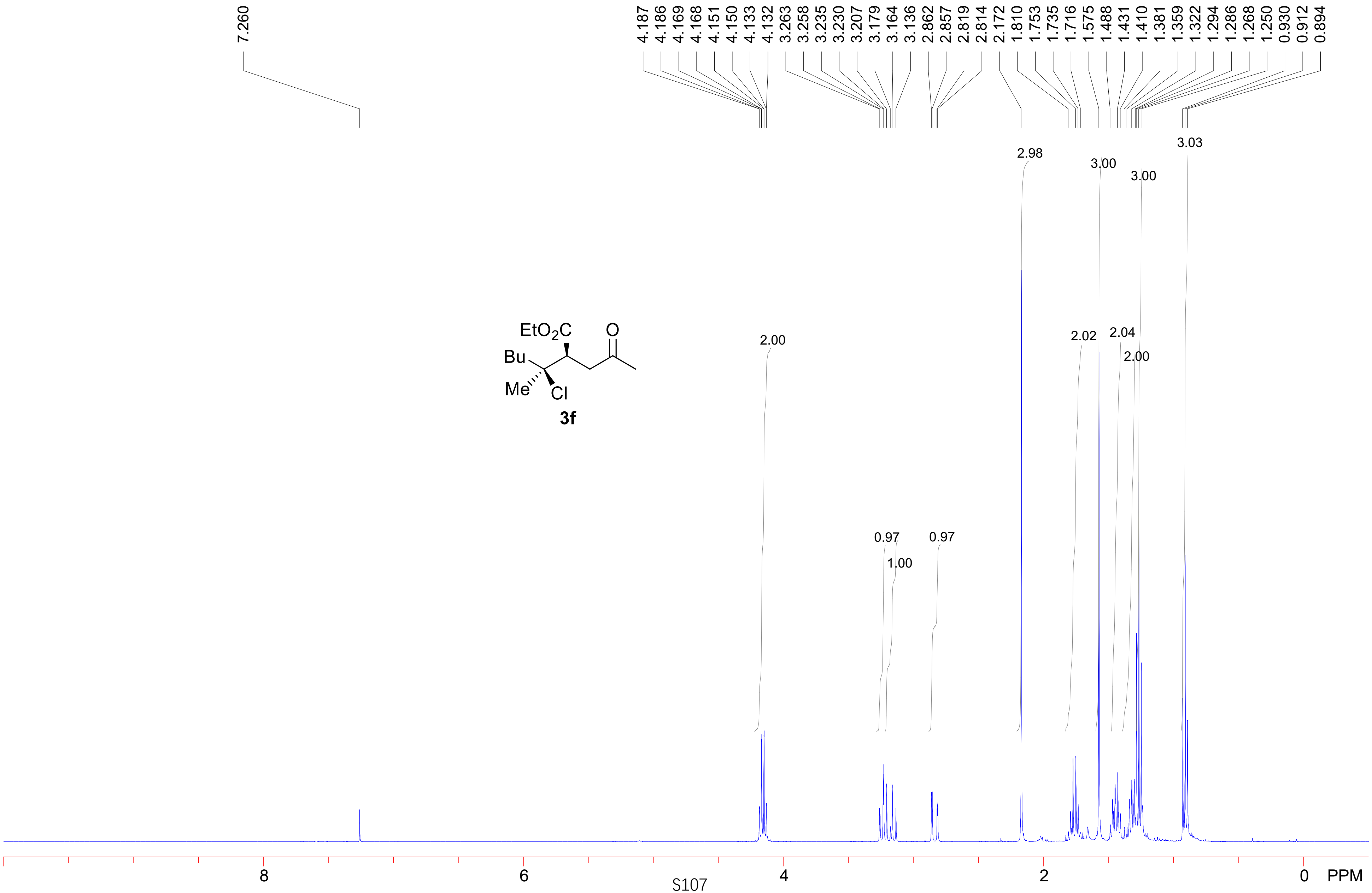
7.260
4.187 4.186 4.169 4.168 4.151 4.150 4.133 4.132 3.263 3.258 3.235 3.230 3.207 3.179 3.164 3.136 2.862 2.857 2.819 2.814 2.172 1.810 1.753 1.735 1.716 1.575 1.488 1.431 1.410 1.381 1.359 1.322 1.294 1.286 1.268 1.250 0.930 0.912 0.894
2.98
3.00
3.00
3.03
EtO2C
O
Bu
Me
Cl
3f
2.00
2.02
2.04
2.00
0.97
1.00
0.97
8
6
4
2
0
PPM

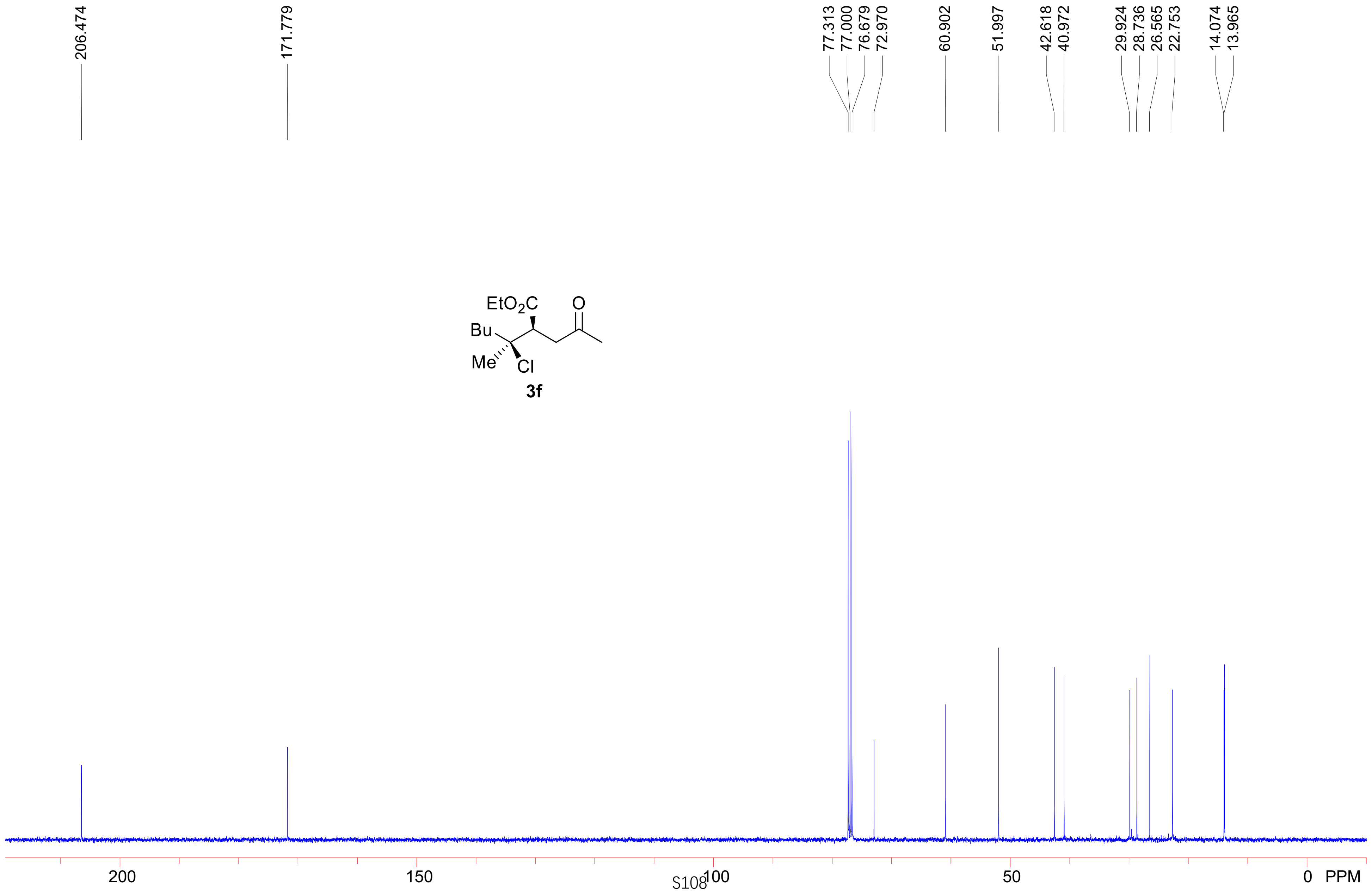
206.474
171.779
77.313
77.000
76.679
72.970
60.902
51.997
42.618
40.972
29.924
28.736
26.565
22.753
14.074
13.965
EtO2C
O
Bu
Me
Cl
3f
200
150
100
50
0
PPM

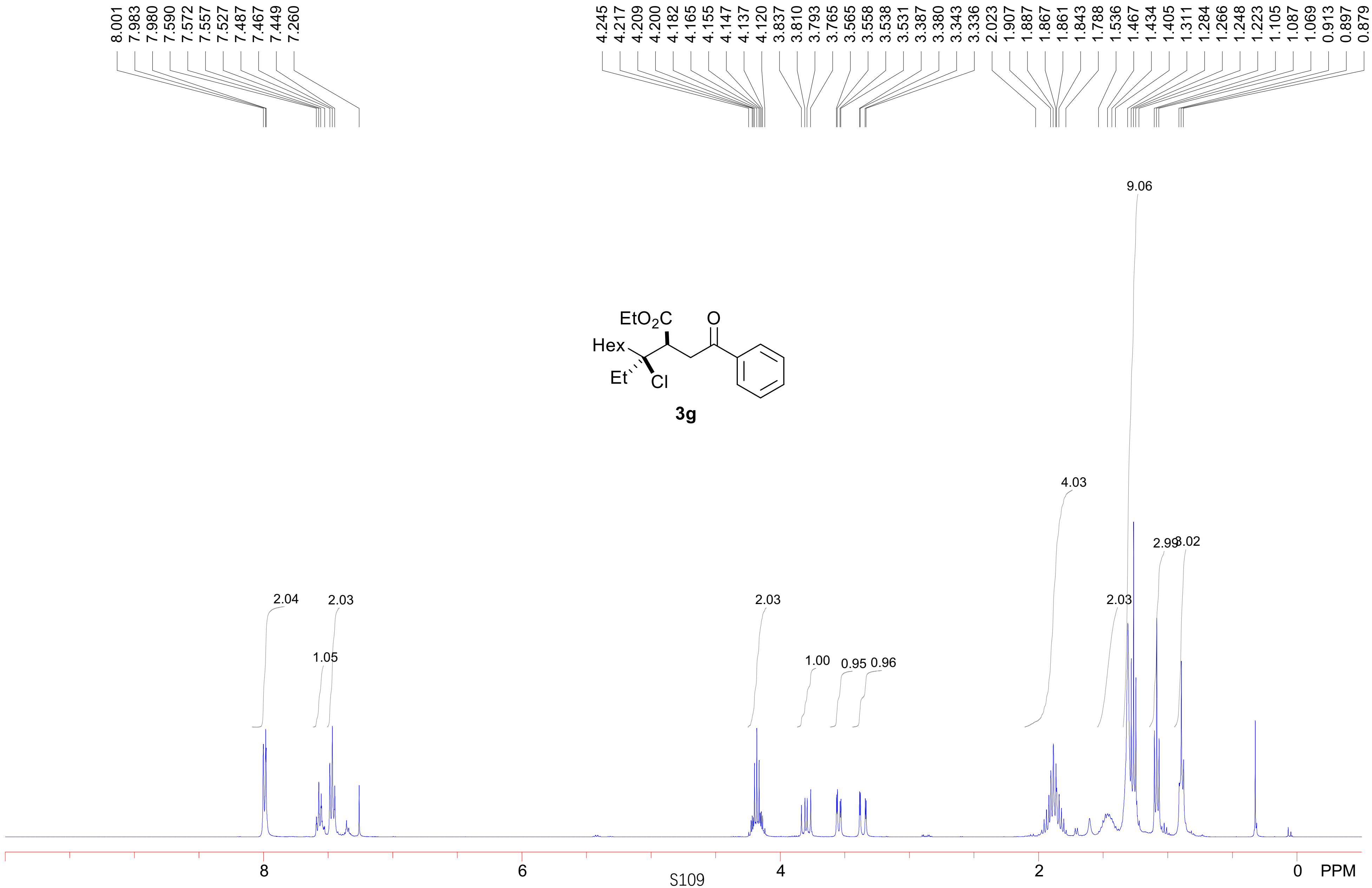
8.001
7.983
7.980
7.590
7.572
7.557
7.527
7.487
7.467
7.449
7.260
4.245
4.217
4.209
4.200
4.182
4.165
4.155
4.147
4.137
4.120
3.837
3.810
3.793
3.765
3.565
3.558
3.538
3.531
3.387
3.380
3.343
3.336
2.023
1.907
1.887
1.867
1.861
1.843
1.788
1.536
1.467
1.434
1.405
1.311
1.284
1.266
1.248
1.223
1.105
1.087
1.069
0.913
0.897
0.879
9.06
EtO2C
O
Hex
Et
Cl
3g
4.03
2.99
3.02
2.04
2.03
1.05
2.03
1.00
0.95
0.96
2.03
8
6
4
2
0
PPM

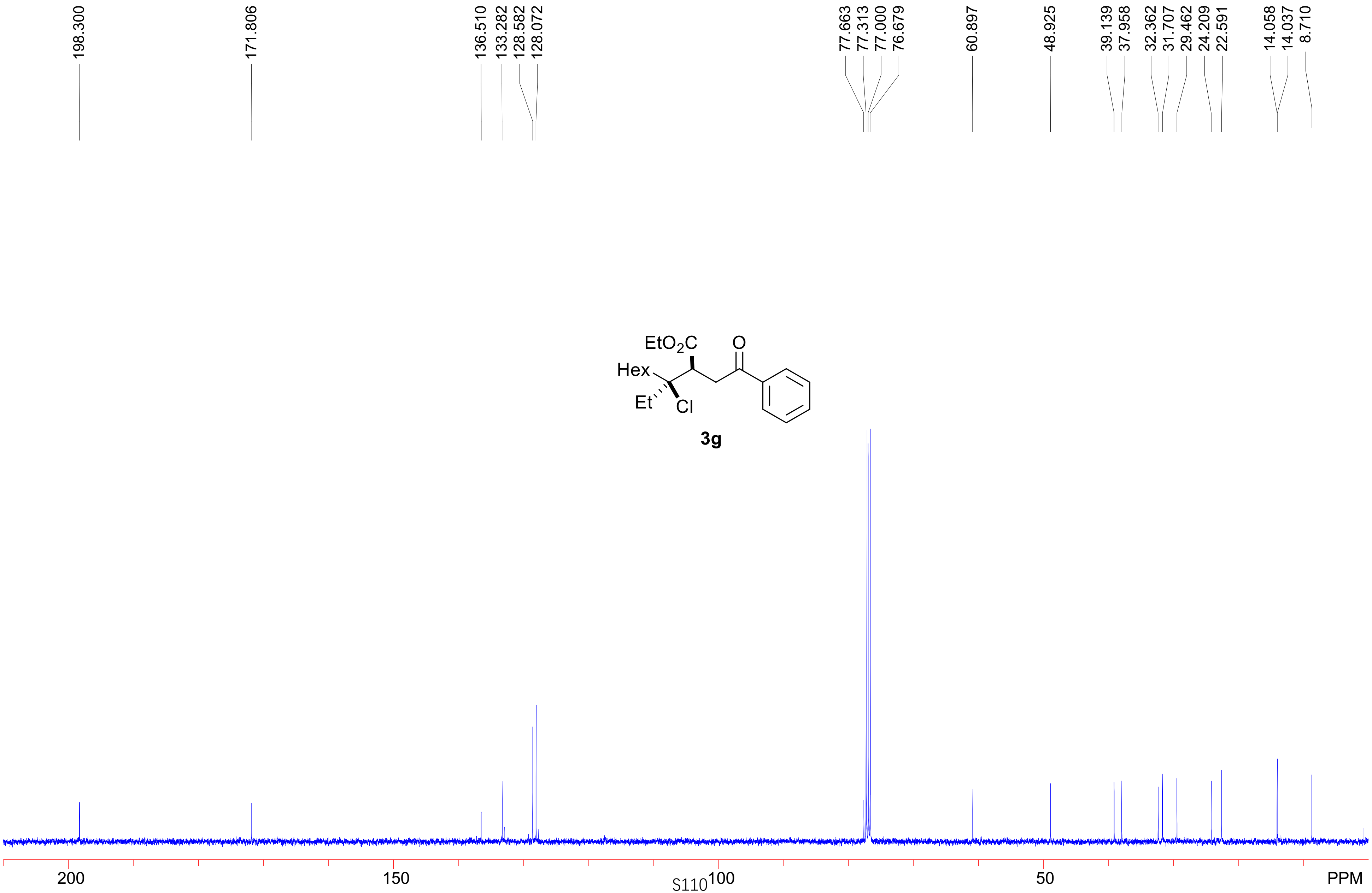
198.300
171.806
136.510
133.282
128.582
128.072
77.663
77.313
77.000
76.679
60.897
48.925
39.139
37.958
32.362
31.707
29.462
24.209
22.591
14.058
14.037
8.710
EtO2C
O
Hex
Et
Cl
3g
200
150
100
50
PPM

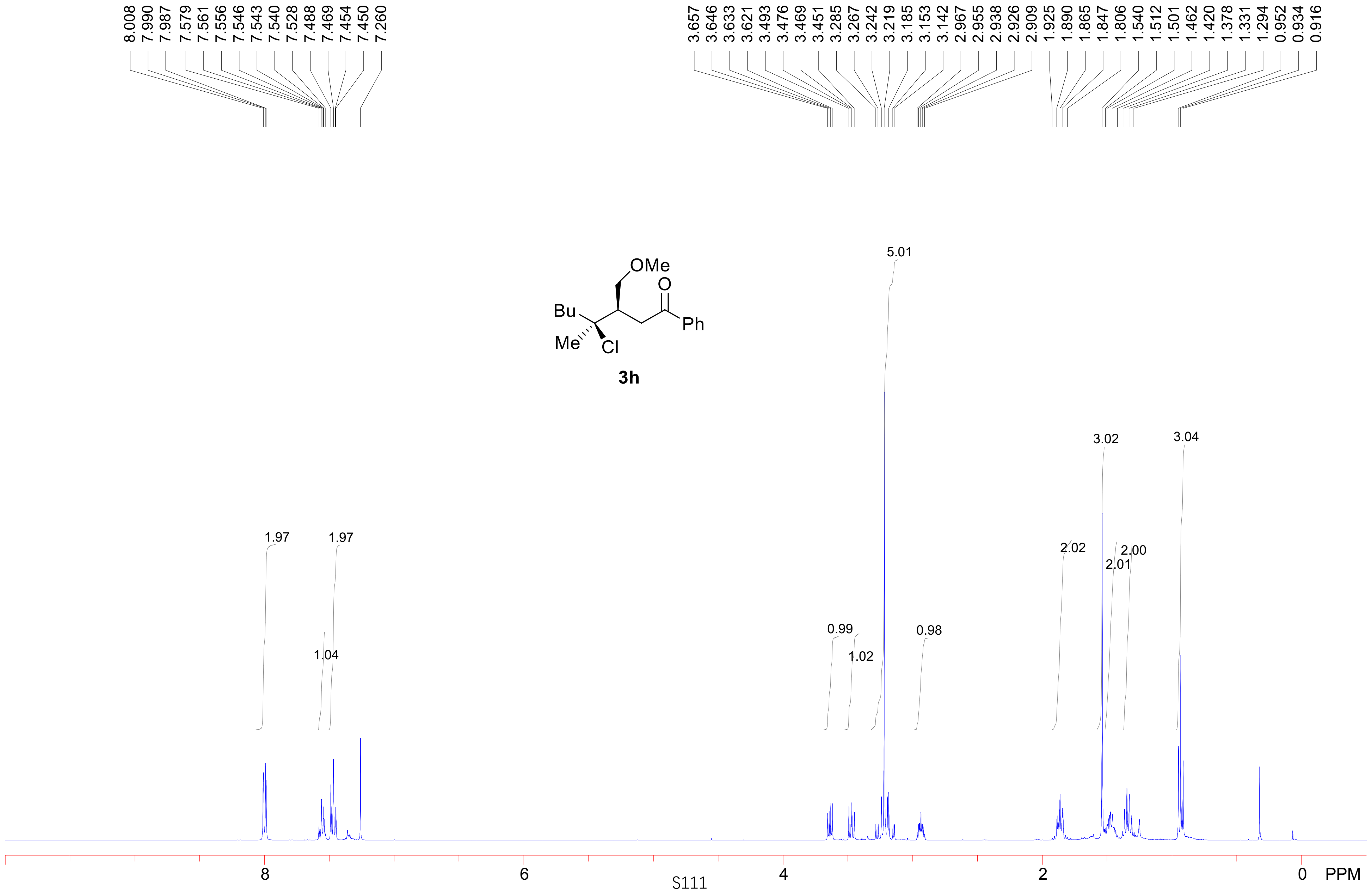
8.008
7.990
7.987
7.579
7.561
7.556
7.546
7.543
7.540
7.528
7.488
7.469
7.454
7.450
7.260
3.657
3.646
3.633
3.621
3.493
3.476
3.469
3.451
3.285
3.267
3.242
3.219
3.185
3.153
3.142
2.967
2.955
2.938
2.926
2.909
1.925
1.890
1.865
1.847
1.806
1.540
1.512
1.501
1.462
1.420
1.378
1.331
1.294
0.952
0.934
0.916
OMe
O
Bu
Ph
Me
Cl
3h
5.01
3.02
3.04
1.97
1.97
1.04
2.02
2.00
2.01
0.99
1.02
0.98
8
6
4
2
0
PPM

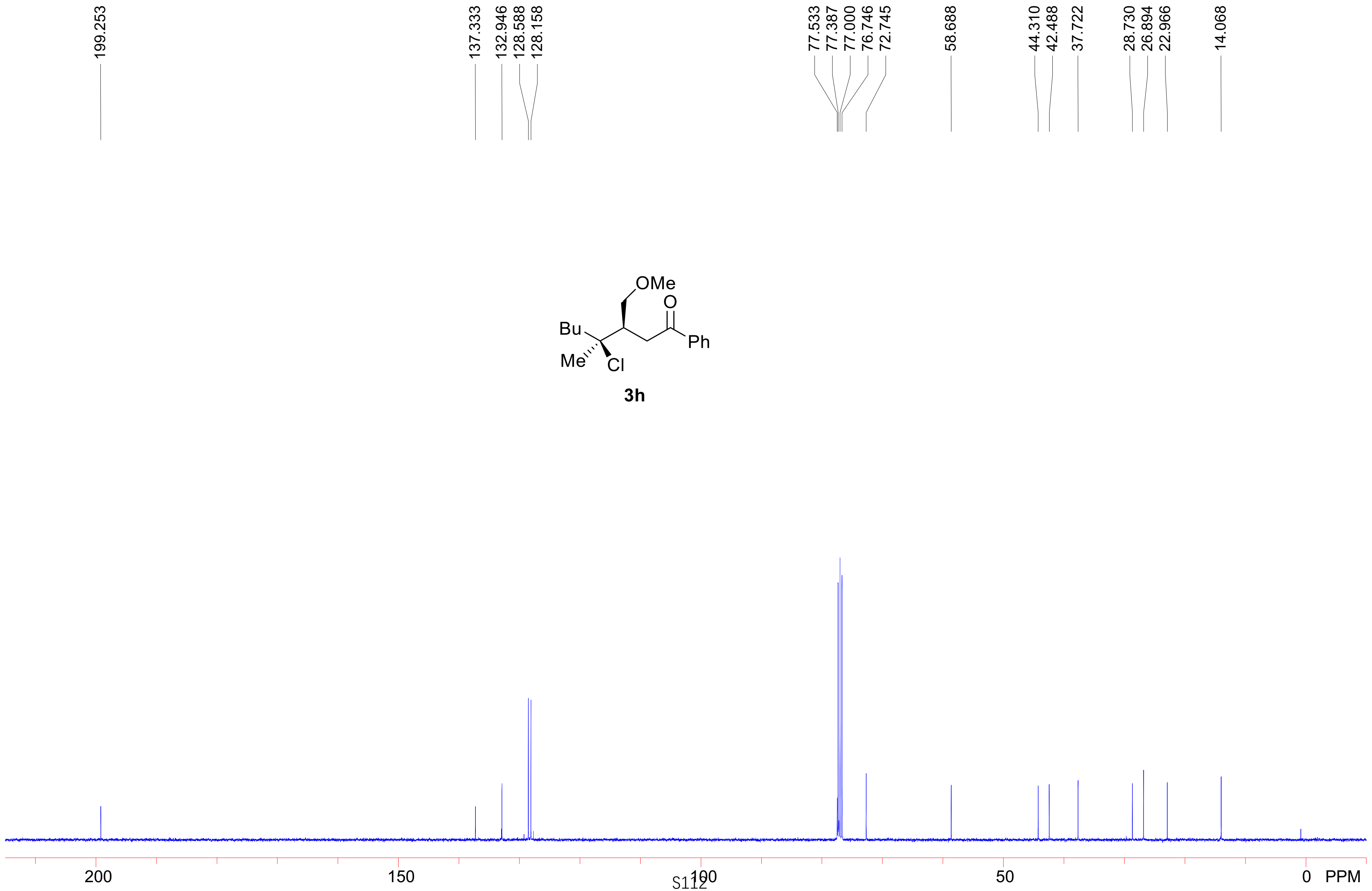
199.253
137.333
132.946
128.588
128.158
77.533
77.387
77.000
76.746
72.745
58.688
44.310
42.488
37.722
28.730
26.894
22.966
14.068
OMe
O
Bu
Ph
Me
Cl
3h
200
150
100
50
0
PPM

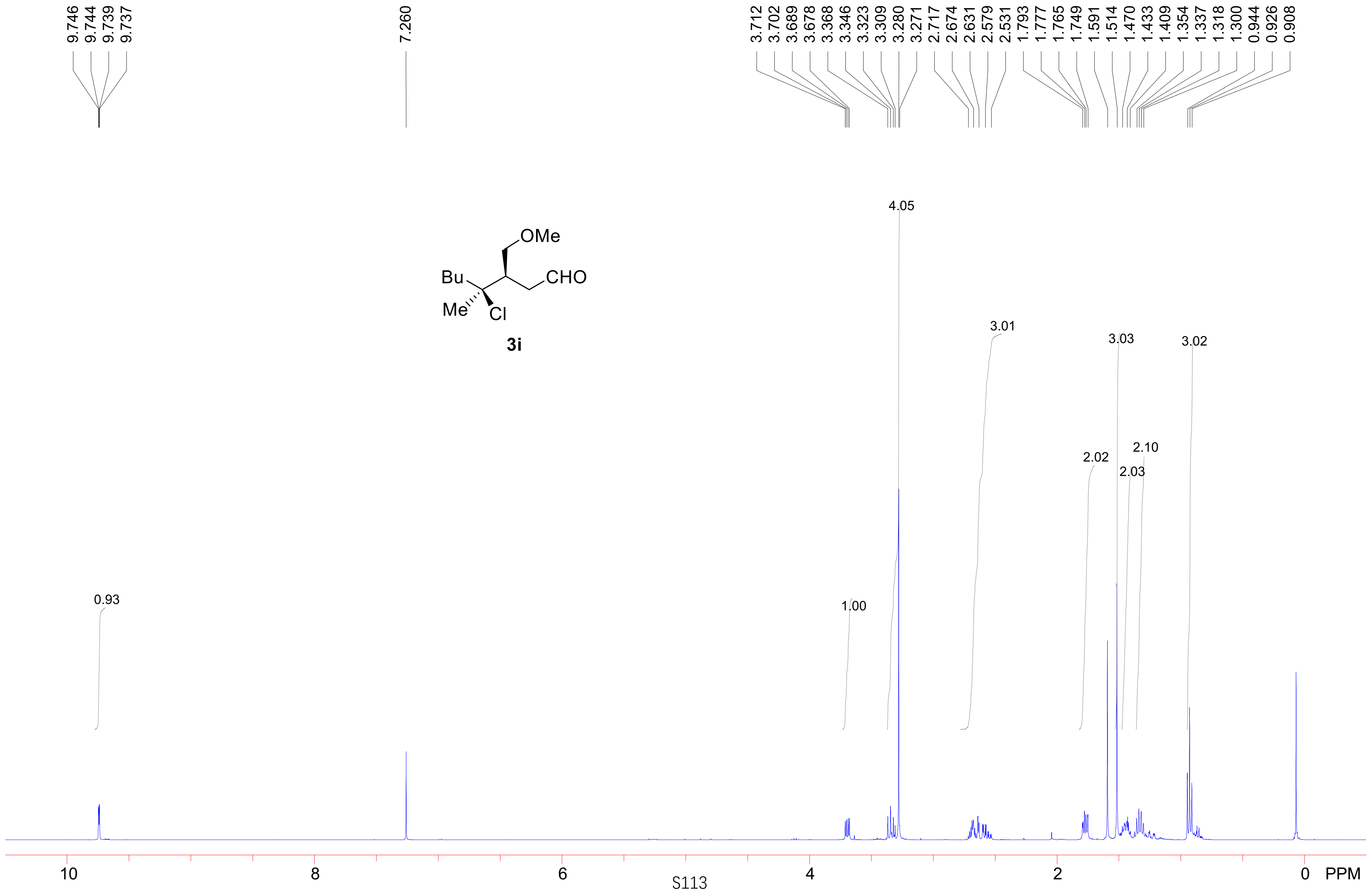
9.746
9.744
9.739
9.737
7.260
3.712
3.702
3.689
3.678
3.368
3.346
3.323
3.309
3.280
3.271
2.717
2.674
2.631
2.579
2.531
1.793
1.777
1.765
1.749
1.591
1.514
1.470
1.433
1.409
1.354
1.337
1.318
1.300
0.944
0.926
0.908
OMe
Bu
CHO
Me
Cl
3i
0.93
1.00
4.05
3.01
2.02
3.03
2.03
2.10
3.02
10
8
6
4
2
0
PPM

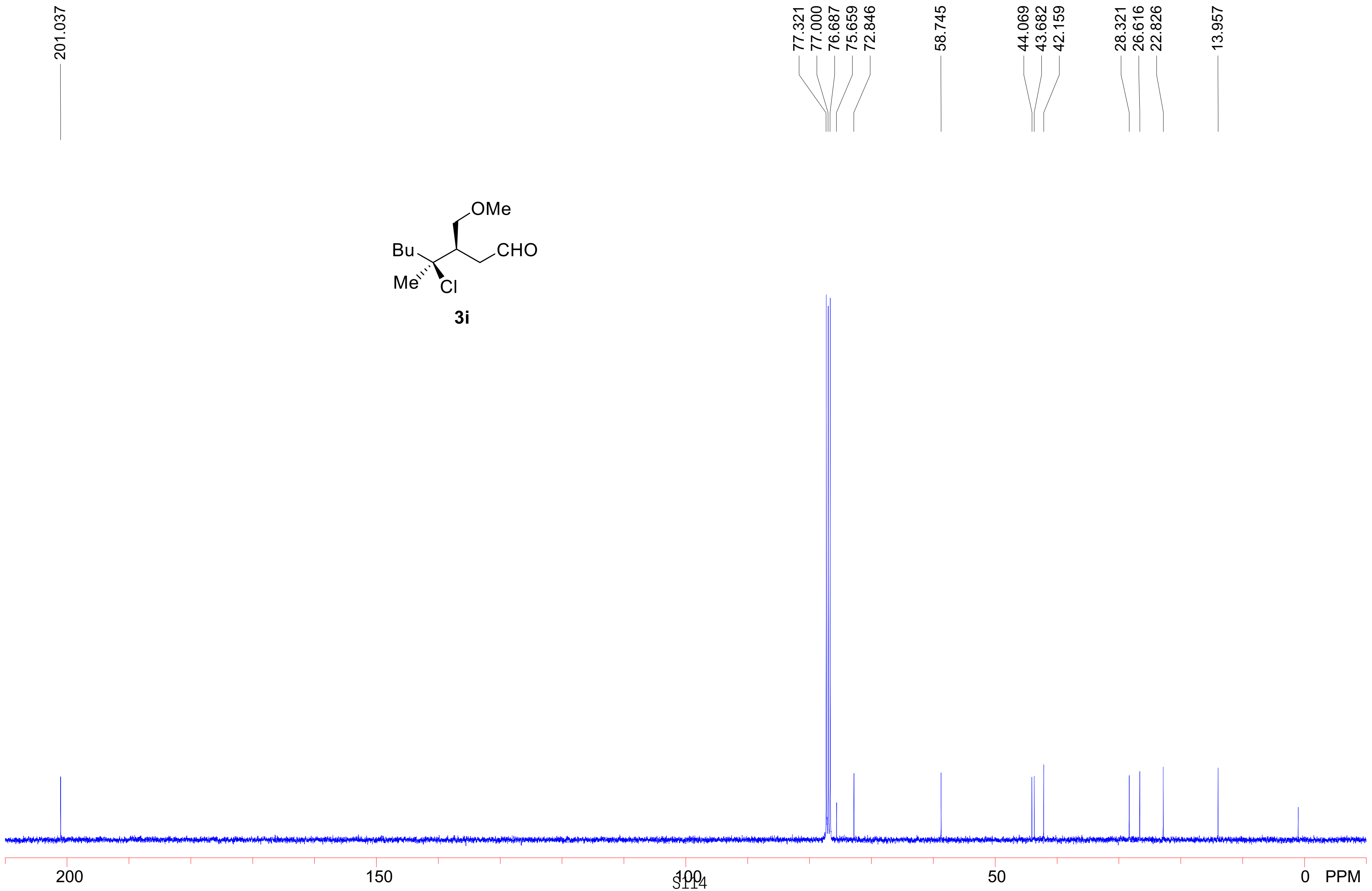
201.037
77.321
77.000
76.687
75.659
72.846
58.745
44.069
43.682
42.159
28.321
26.616
22.826
13.957
OMe
Bu
CHO
Me
Cl
3i
200
150
100
50
0
PPM

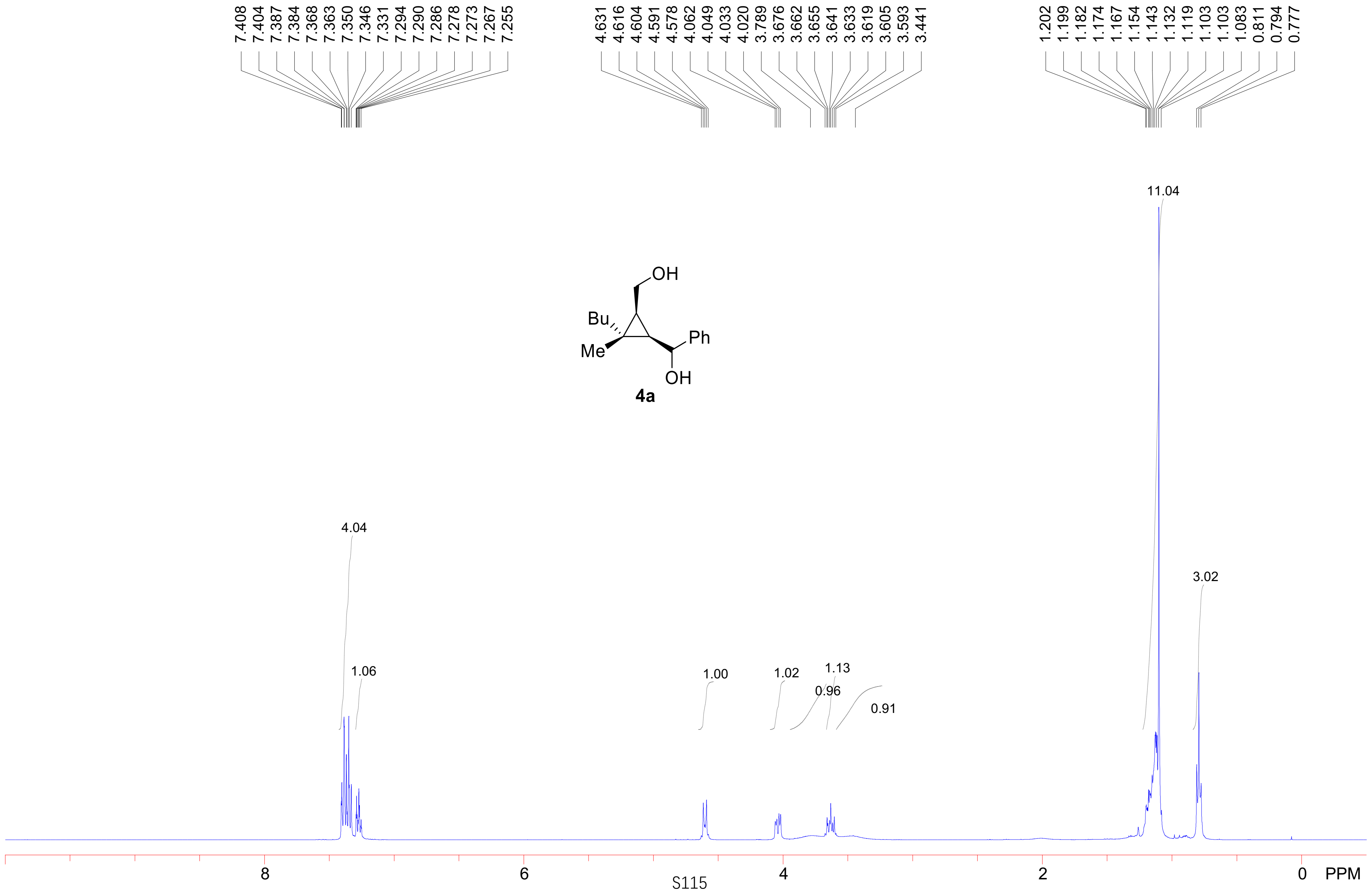
7.408 7.404 7.387 7.384 7.368 7.363 7.350 7.346 7.331 7.294 7.290 7.286 7.278 7.273 7.267 7.255
4.631 4.616 4.604 4.591 4.578 4.062 4.049 4.033 4.020 3.789 3.676 3.662 3.655 3.641 3.633 3.619 3.605 3.593 3.441
1.202 1.199 1.182 1.174 1.167 1.154 1.143 1.132 1.119 1.103 1.103 1.083 0.811 0.794 0.777
OH
Bu
Ph
Me
OH
4a
11.04
4.04
3.02
1.06
1.00
1.02
1.13
0.96
0.91
8
6
4
2
0
PPM

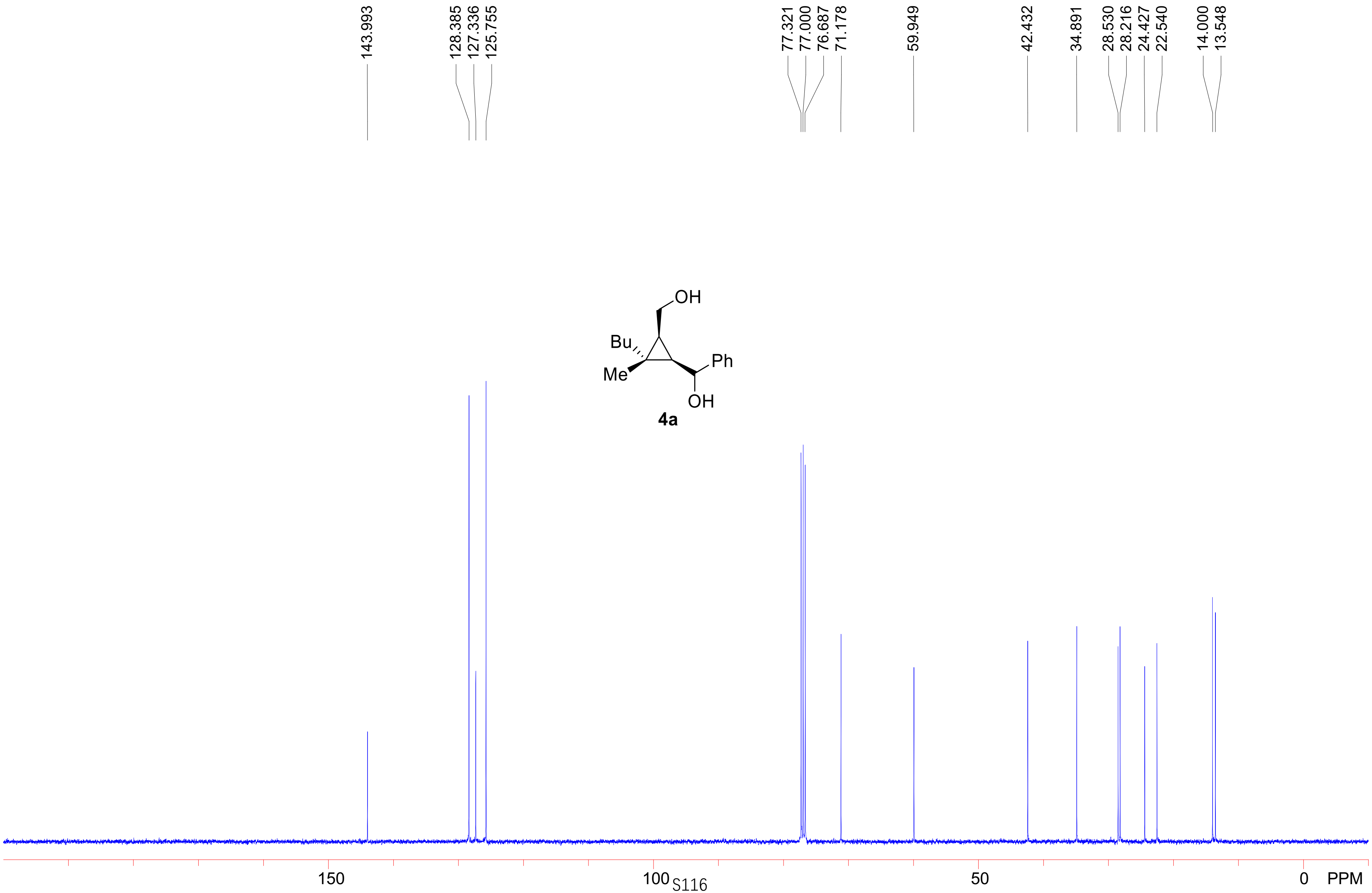
143.993
128.385
127.336
125.755
77.321
77.000
76.687
71.178
59.949
42.432
34.891
28.530
28.216
24.427
22.540
14.000
13.548
OH
Bu
Ph
Me
OH
4a
150
100
50
0
PPM

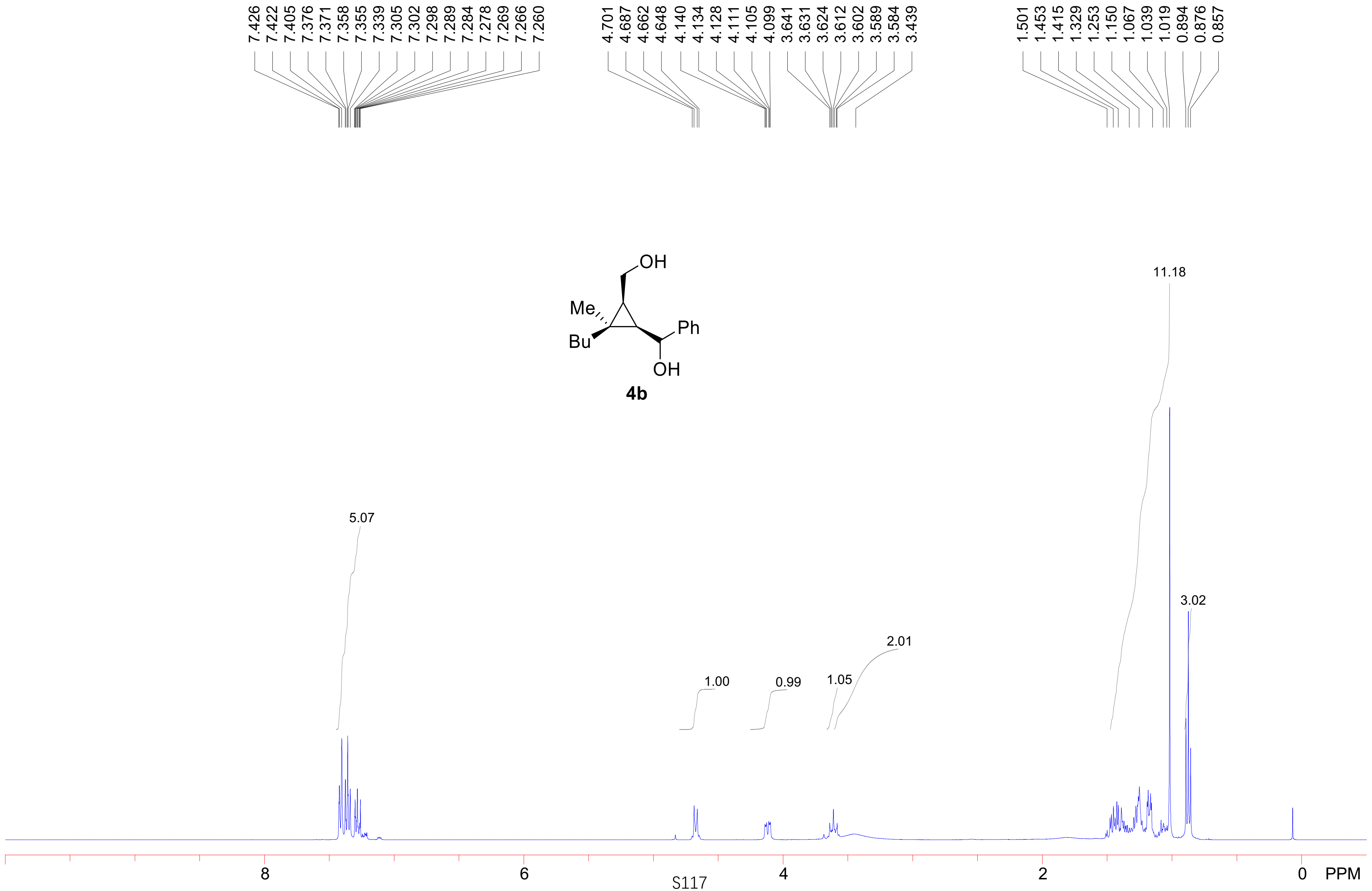
7.426 7.422 7.405 7.376 7.371 7.358 7.355 7.339 7.305 7.302 7.298 7.289 7.284 7.278 7.269 7.266 7.260
4.701 4.687 4.662 4.648 4.140 4.134 4.128 4.111 4.105 4.099 3.641 3.631 3.624 3.612 3.602 3.589 3.584 3.439
1.501 1.453 1.415 1.329 1.253 1.150 1.067 1.039 1.019 0.894 0.876 0.857
OH
Me
Ph
Bu
OH
4b
11.18
5.07
3.02
2.01
1.00
0.99
1.05
8
6
4
2
0
PPM

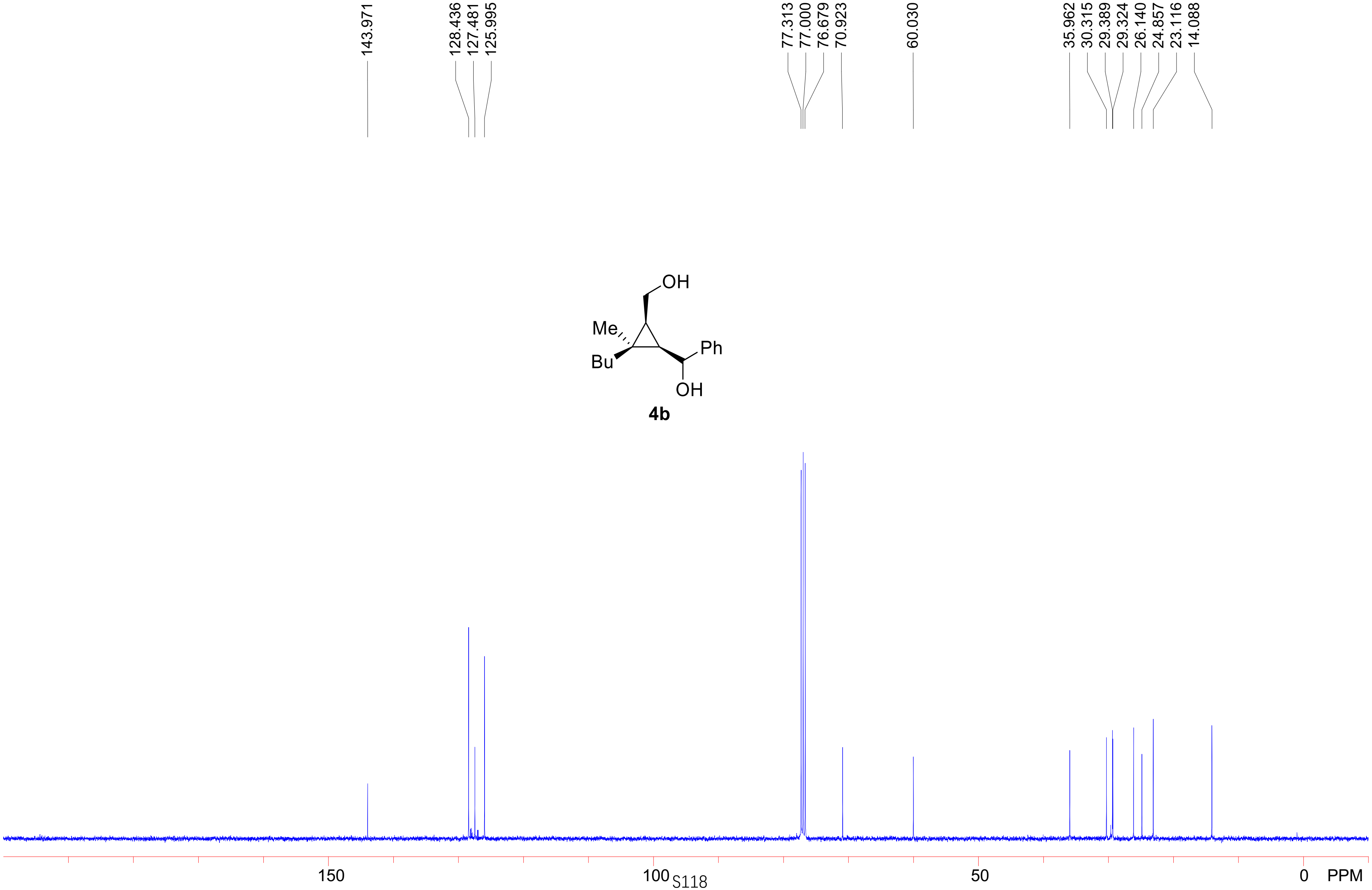
143.971
128.436
127.481
125.995
77.313
77.000
76.679
70.923
60.030
35.962
30.315
29.389
29.324
26.140
24.857
23.116
14.088
OH
Me
Ph
Bu
OH
4b
150
100
50
0
PPM

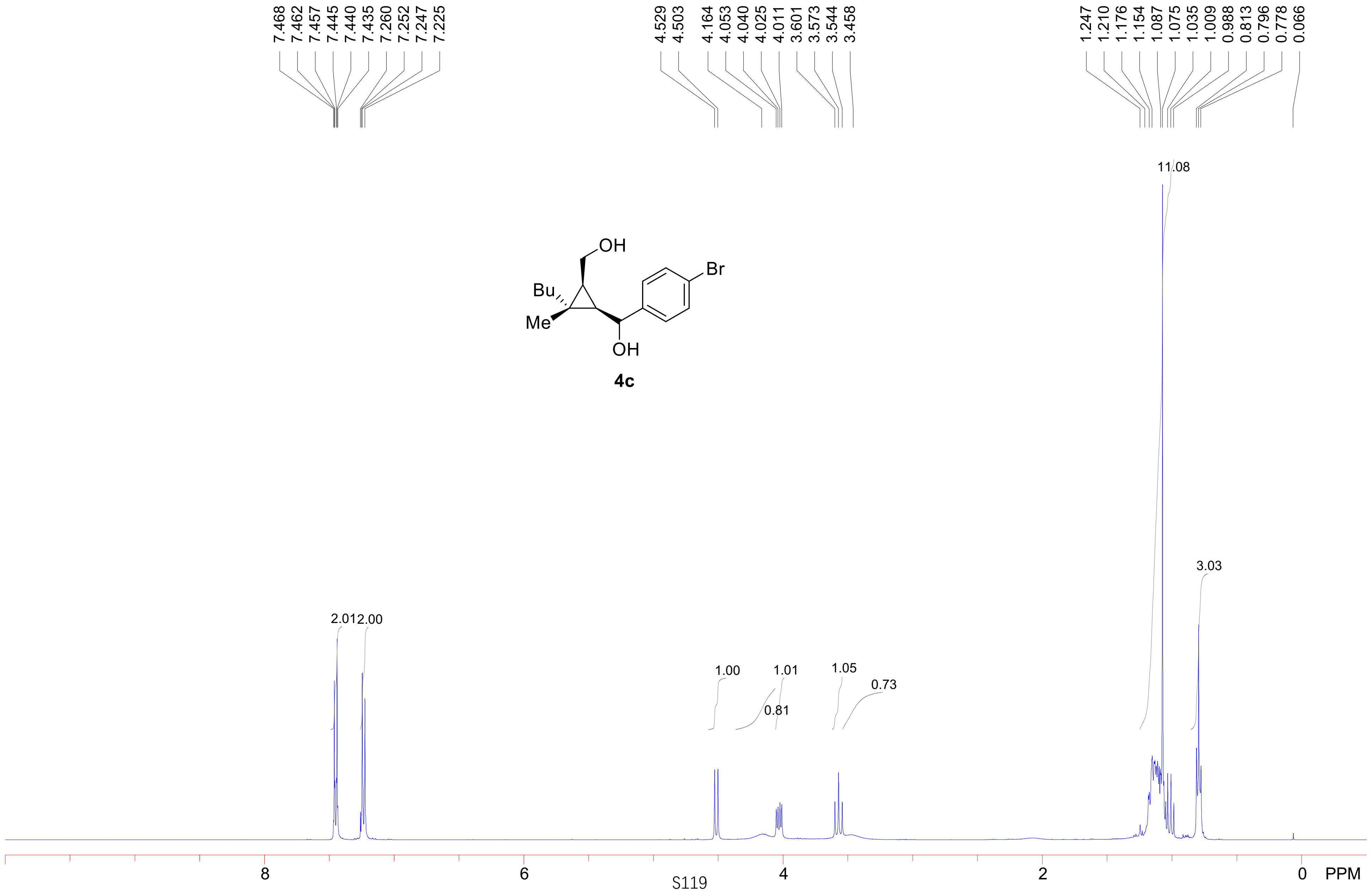
7.468
7.462
7.457
7.445
7.440
7.435
7.260
7.252
7.247
7.225
4.529
4.503
4.164
4.053
4.040
4.025
4.011
3.601
3.573
3.544
3.458
1.247
1.210
1.176
1.154
1.087
1.075
1.035
1.009
0.988
0.813
0.796
0.778
0.066
OH
Br
Bu
Me
OH
4c
11.08
3.03
2.01
2.00
1.00
1.01
0.81
1.05
0.73
8
6
4
2
0
PPM

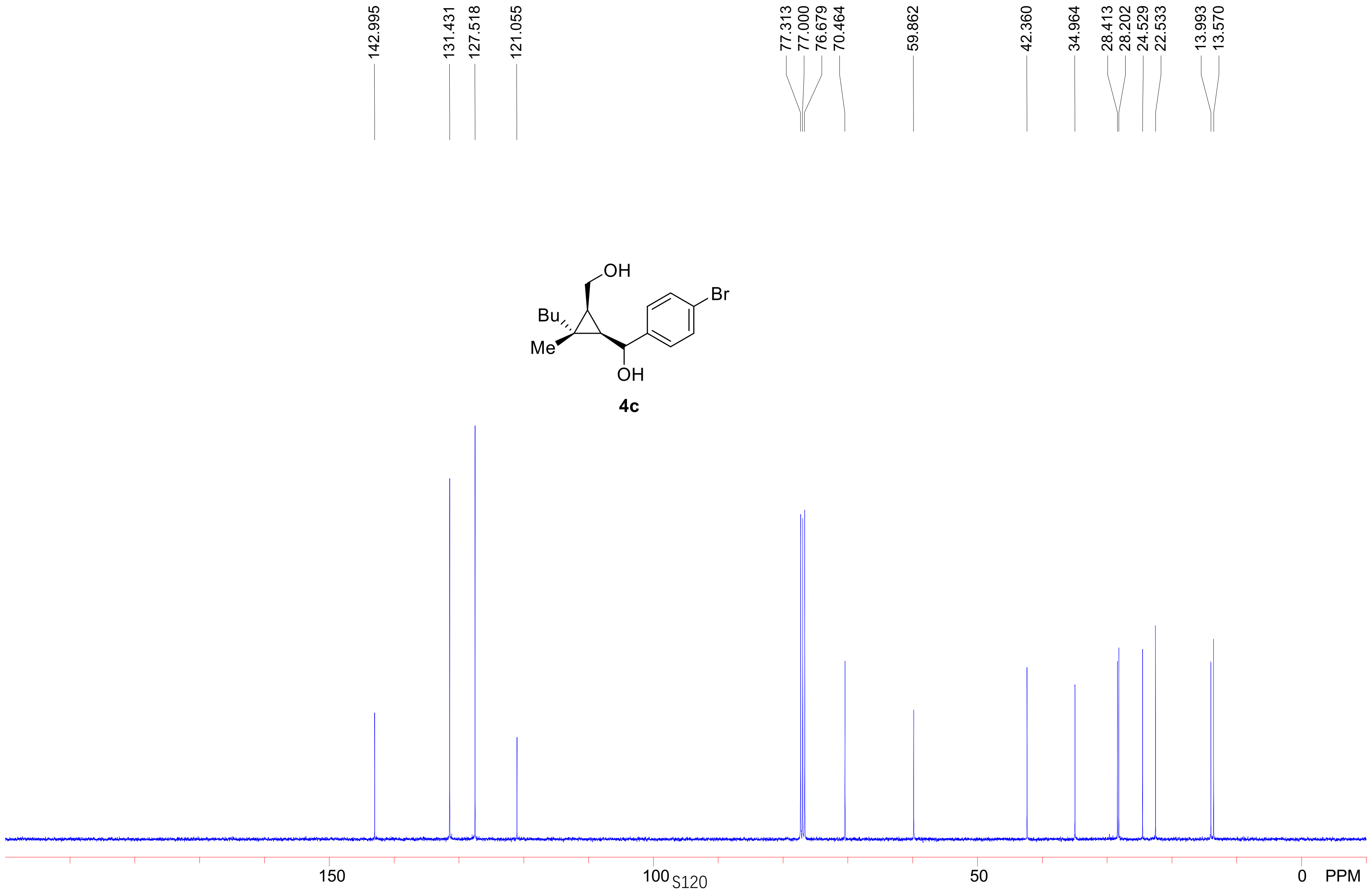
142.995
131.431
127.518
121.055
77.313
77.000
76.679
70.464
59.862
42.360
34.964
28.413
28.202
24.529
22.533
13.993
13.570
OH
Br
Bu
Me
OH
4c
150
100
50
0
PPM

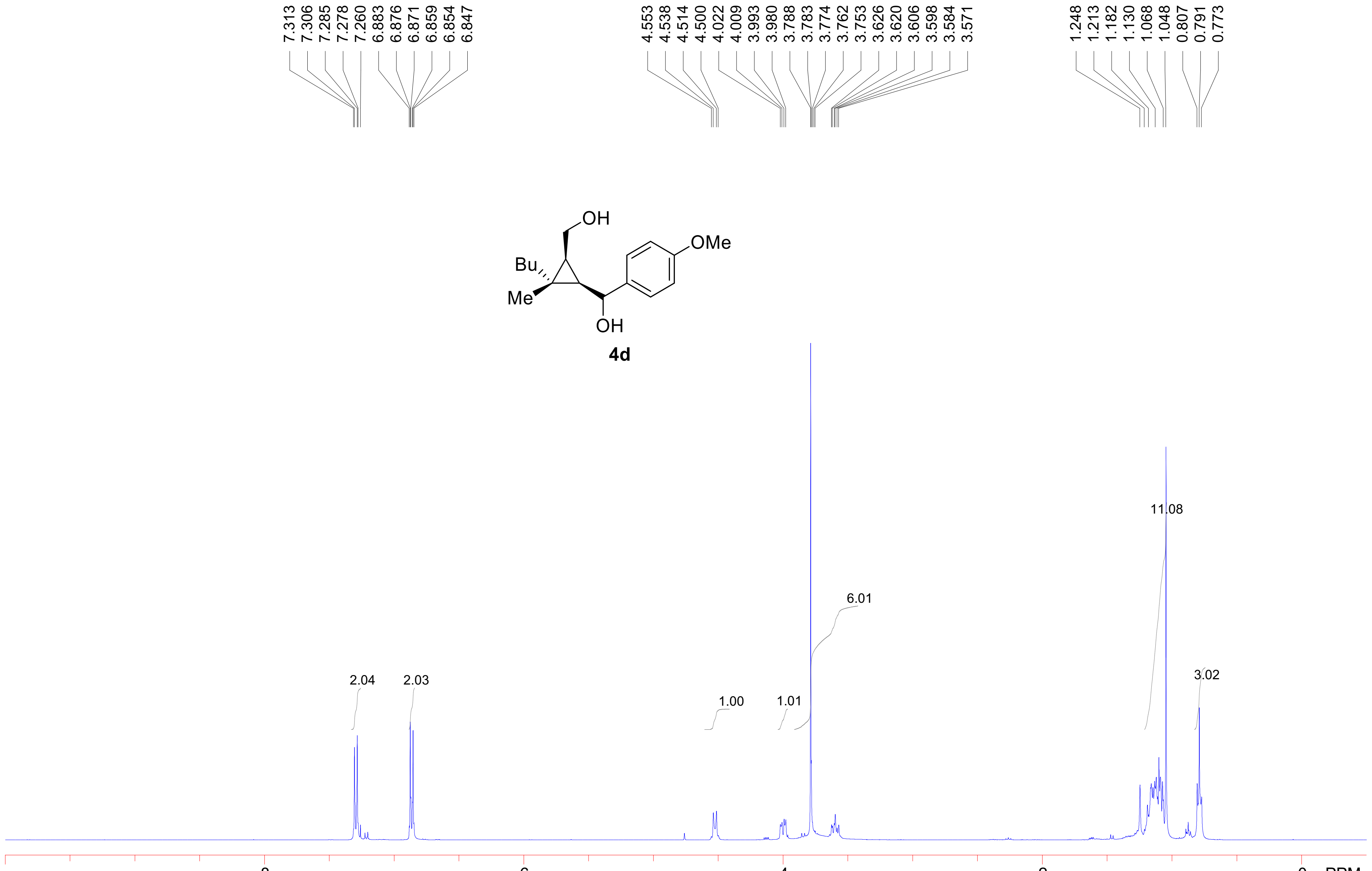
7.313
7.306
7.285
7.278
7.260
6.883
6.876
6.871
6.859
6.854
6.847
4.553
4.538
4.514
4.500
4.022
4.009
3.993
3.980
3.788
3.783
3.774
3.762
3.753
3.626
3.620
3.606
3.598
3.584
3.571
1.248
1.213
1.182
1.130
1.068
1.048
0.807
0.791
0.773
OH
OMe
Bu
Me
OH
4d
11.08
6.01
2.04
2.03
1.00
1.01
3.02
8
6
4
2
0
PPM

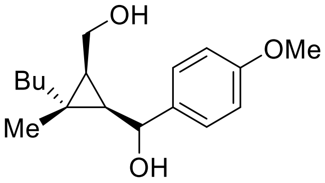
OH
OMe
Bu
Me
OH

4d

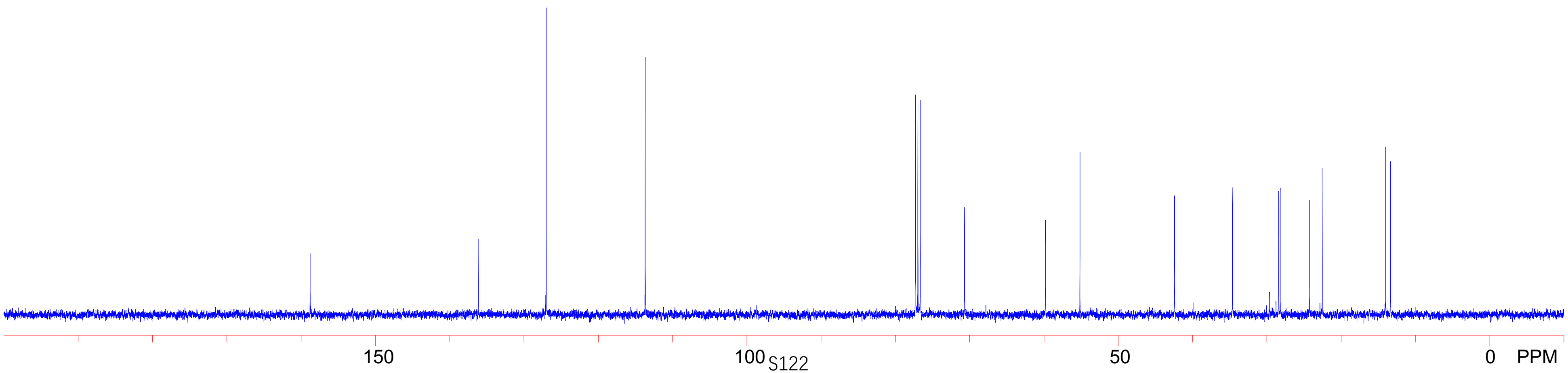
158.777
136.160
127.008
113.681
77.321
77.000
76.687
70.726
59.833
55.177
42.432
34.650
28.413
28.202
24.274
22.533
13.993
13.374
150
100
50
0
PPM

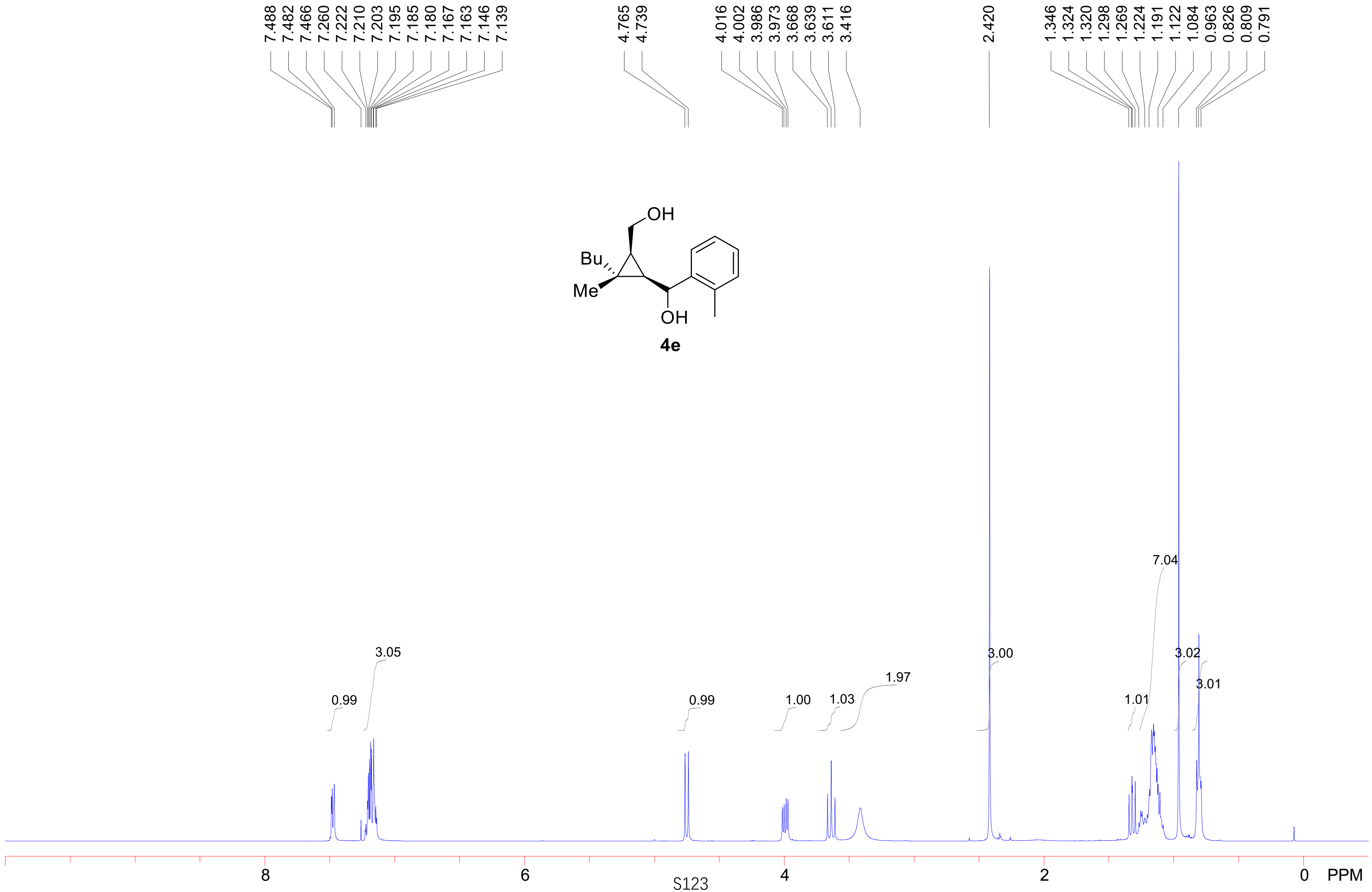
7.488 7.482 7.466 7.260 7.222 7.210 7.203 7.195 7.185 7.180 7.167 7.163 7.146 7.139
4.765 4.739
4.016 4.002 3.986 3.973 3.668 3.639 3.611 3.416
2.420
1.346 1.324 1.320 1.298 1.269 1.224 1.191 1.122 1.084 0.963 0.826 0.809 0.791
OH
Bu
Me
OH
4e
0.99
3.05
0.99
1.00
1.03
1.97
3.00
1.01
7.04
3.02
3.01
8
6
4
2
0
PPM

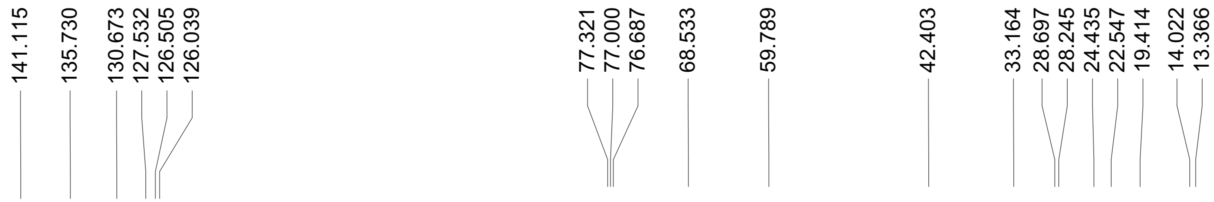
141.115
135.730
130.673
127.532
126.505
126.039
77.321
77.000
76.687
68.533
59.789
42.403
33.164
28.697
28.245
24.435
22.547
19.414
14.022
13.366

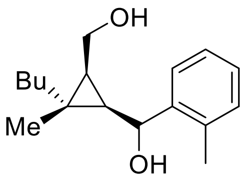
OH
Bu
Me
OH

4e

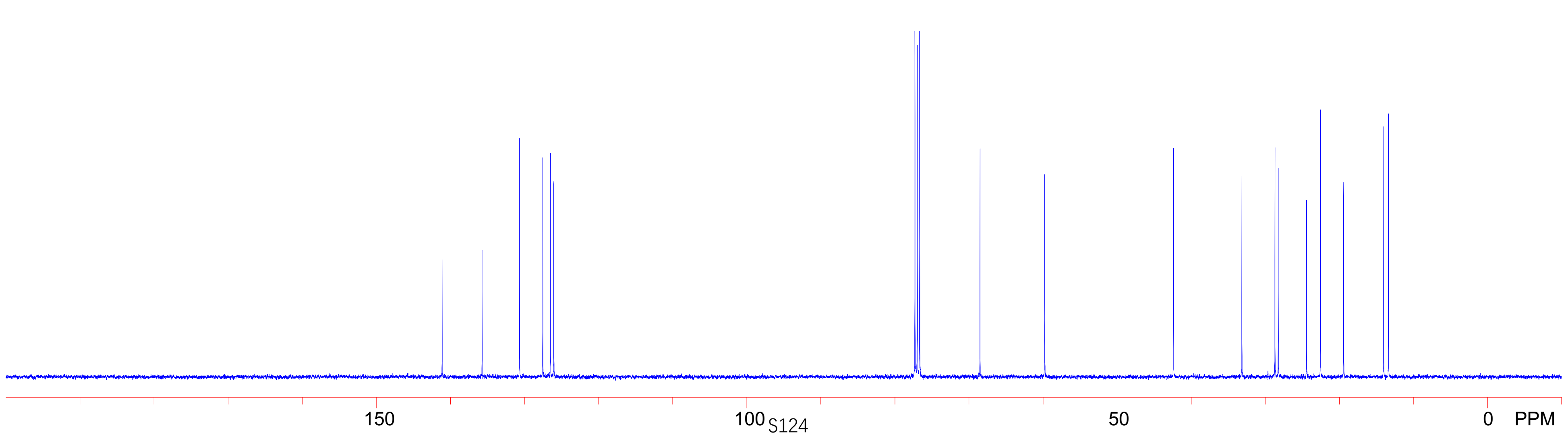
150
100
50
0
PPM

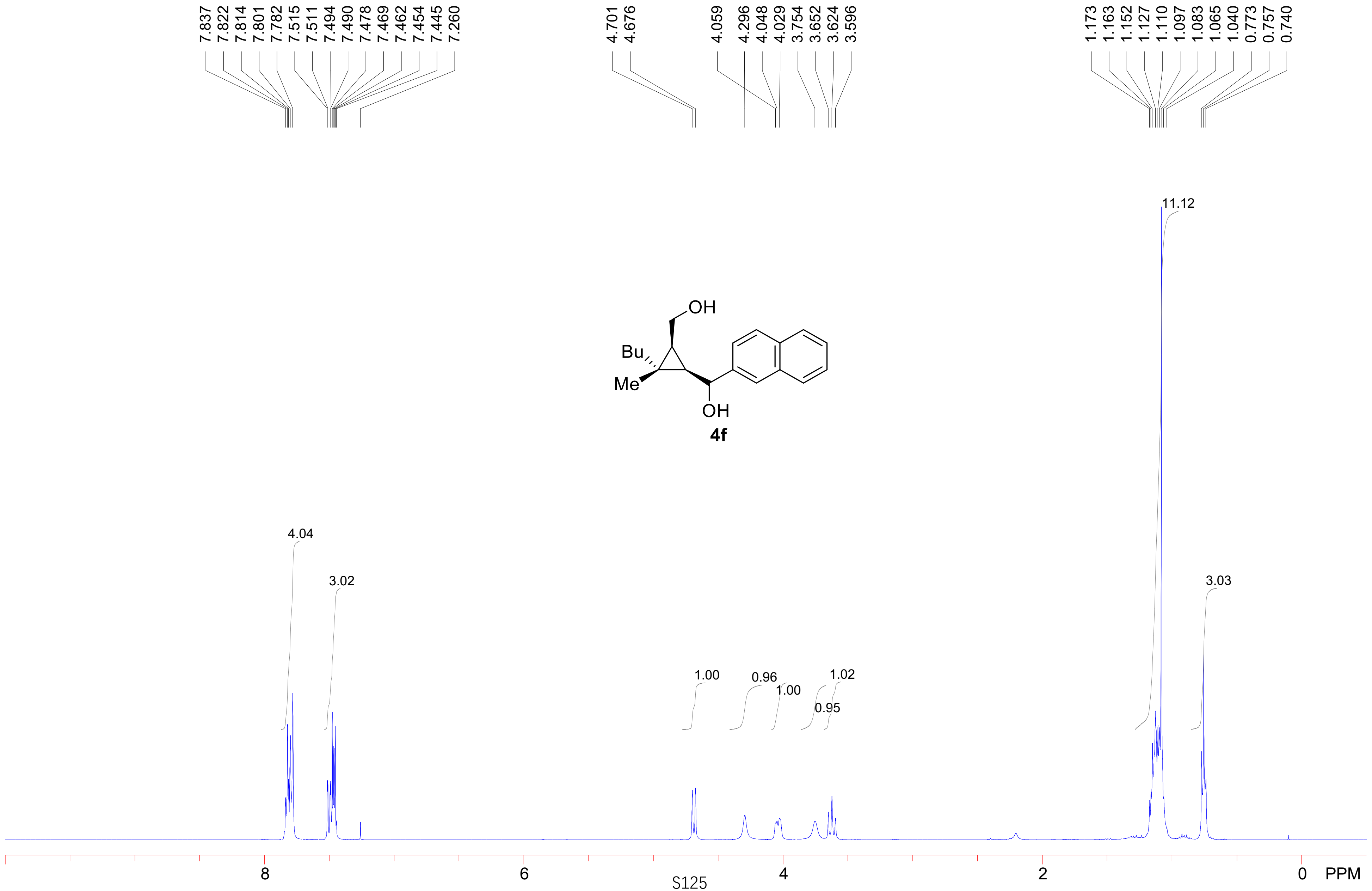
7.837 7.822 7.814 7.801 7.782 7.515 7.511 7.494 7.490 7.478 7.469 7.462 7.454 7.445 7.260
4.701 4.676
4.059 4.296 4.048 4.029 3.754 3.652 3.624 3.596
1.173 1.163 1.152 1.127 1.110 1.097 1.083 1.065 1.040 0.773 0.757 0.740
OH
Bu
Me
OH
4f
11.12
4.04
3.02
3.03
1.00
0.96
1.00
1.02
0.95
8
6
4
2
0
PPM

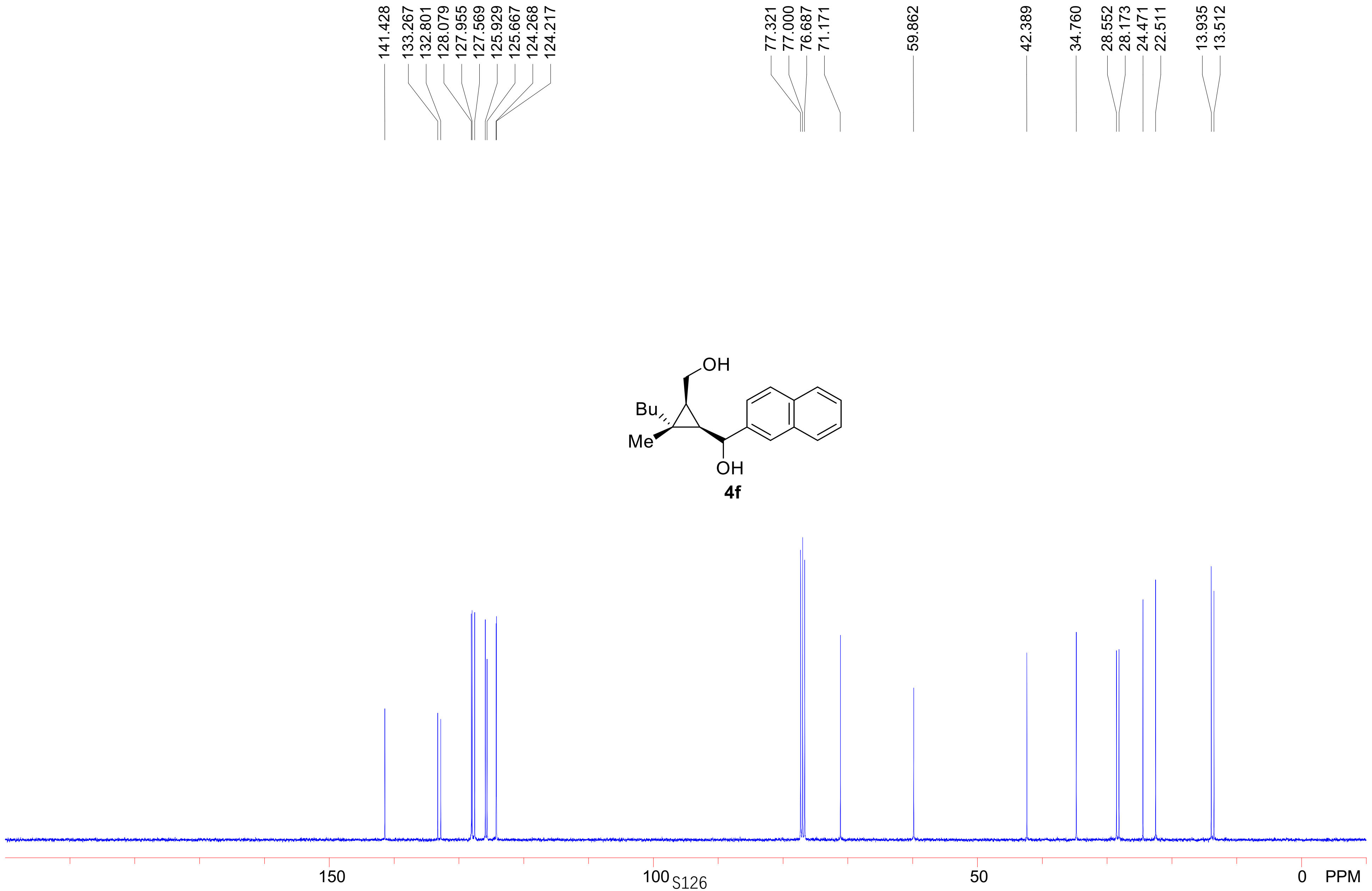
141.428
133.267
132.801
128.079
127.955
127.569
125.929
125.667
124.268
124.217
77.321
77.000
76.687
71.171
59.862
42.389
34.760
28.552
28.173
24.471
22.511
13.935
13.512
OH
Bu
Me
OH
4f
150
100
50
0
PPM

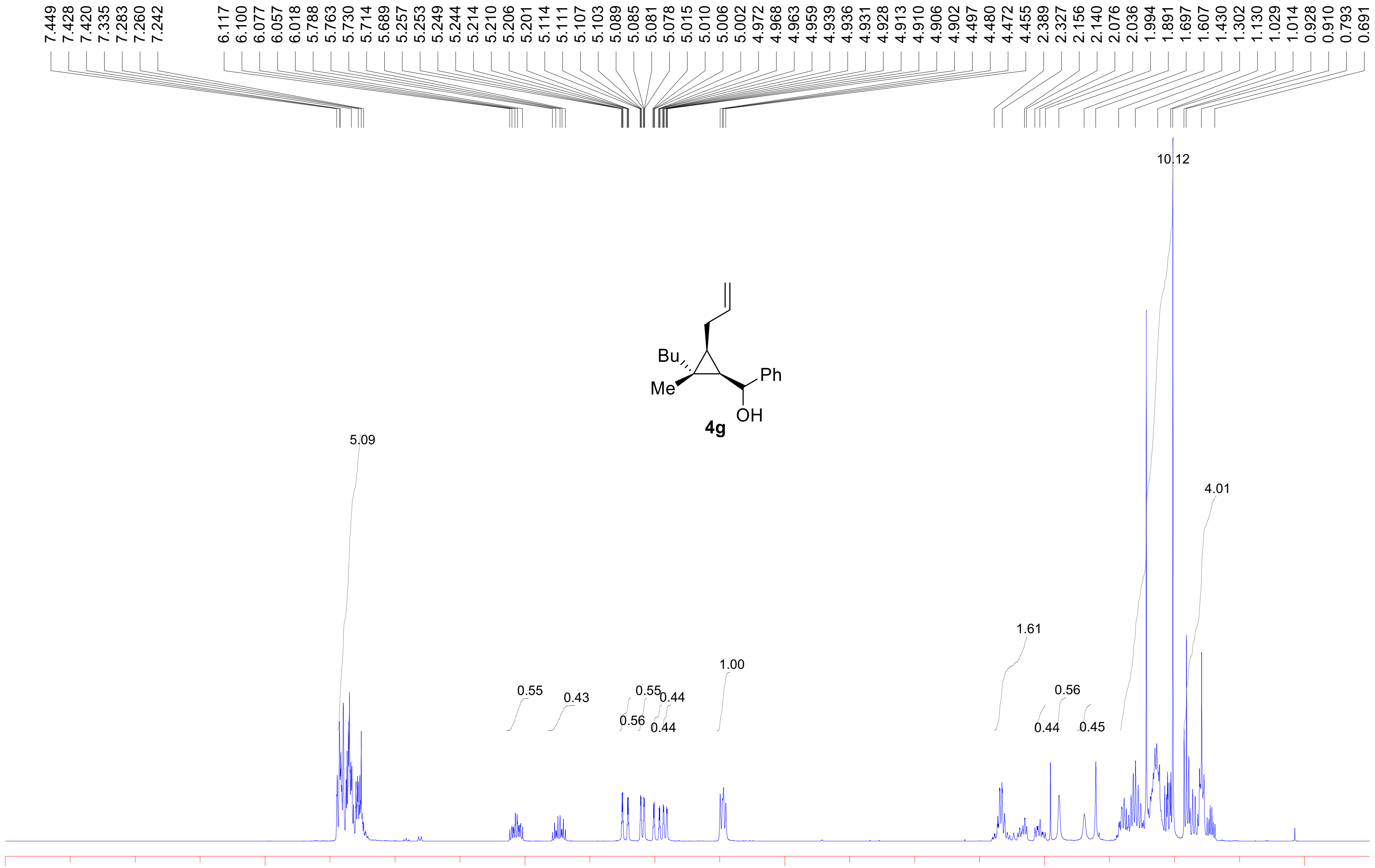
7.449 7.428 7.420 7.335 7.283 7.260 7.242
6.117 6.100 6.077 6.057 6.018 5.788 5.763 5.730 5.714 5.689 5.257 5.253 5.249 5.244 5.214 5.210 5.206 5.201 5.114 5.111 5.107 5.103 5.089 5.085 5.081 5.078 5.015 5.010 5.006 5.002 4.972 4.968 4.963 4.959 4.939 4.936 4.931 4.928 4.913 4.910 4.906 4.902 4.497 4.480 4.472 4.455 2.389 2.327 2.156 2.140 2.076 2.036 1.994 1.891 1.697 1.607 1.430 1.302 1.130 1.029 1.014 0.928 0.910 0.793 0.691
10.12
5.09
4.01
1.61
1.00
0.55
0.43
0.55
0.44
0.56
0.44
0.56
0.44
0.45
Bu
Me
Ph
OH
4g
8
6
4
2
0
PPM

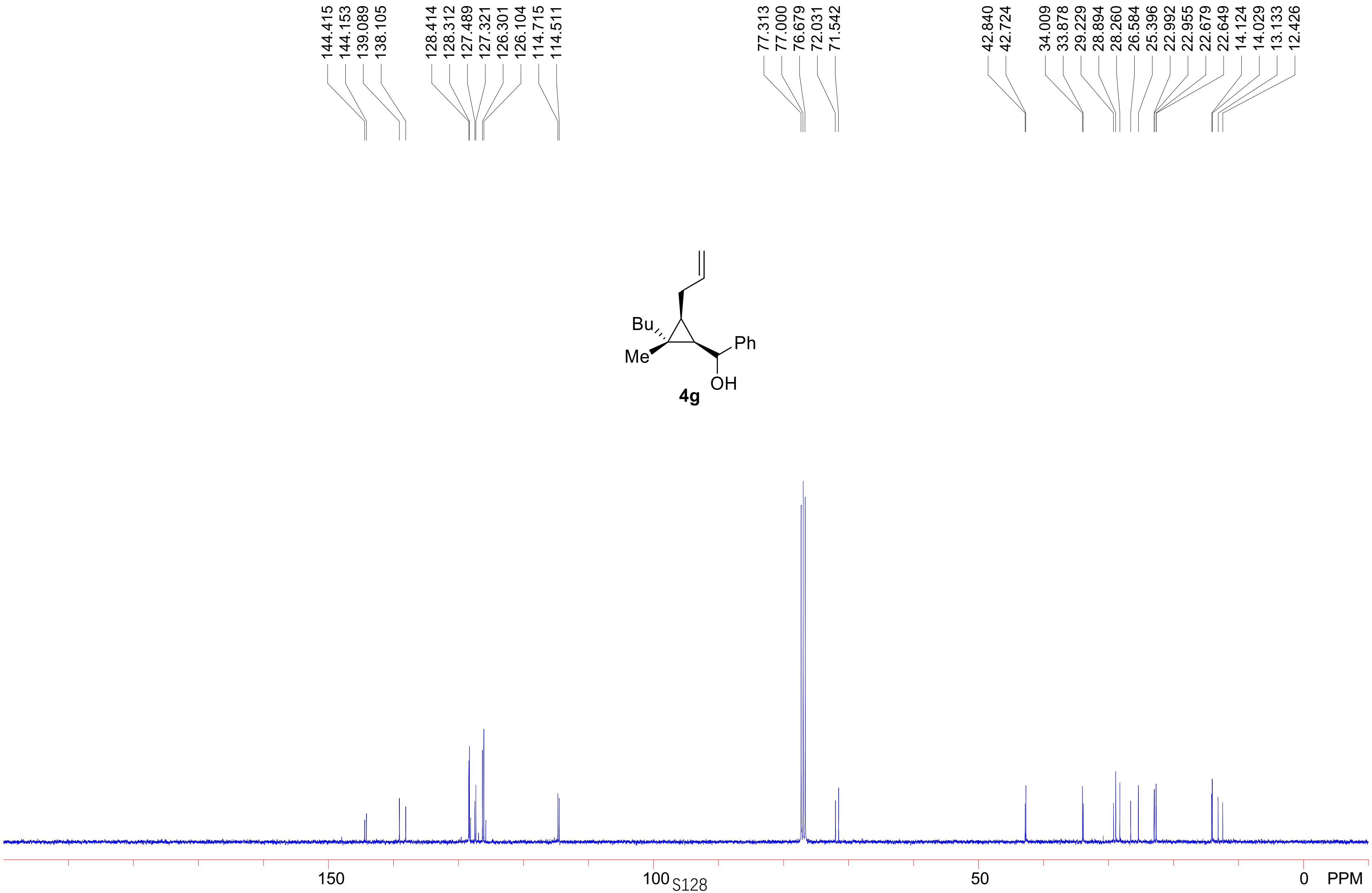
144.415
144.153
139.089
138.105
128.414
128.312
127.489
127.321
126.301
126.104
114.715
114.511
77.313
77.000
76.679
72.031
71.542
42.840
42.724
34.009
33.878
29.229
28.894
28.260
26.584
25.396
22.992
22.955
22.679
22.649
14.124
14.029
13.133
12.426
Bu
Me
Ph
OH
4g
150
100
50
0
PPM

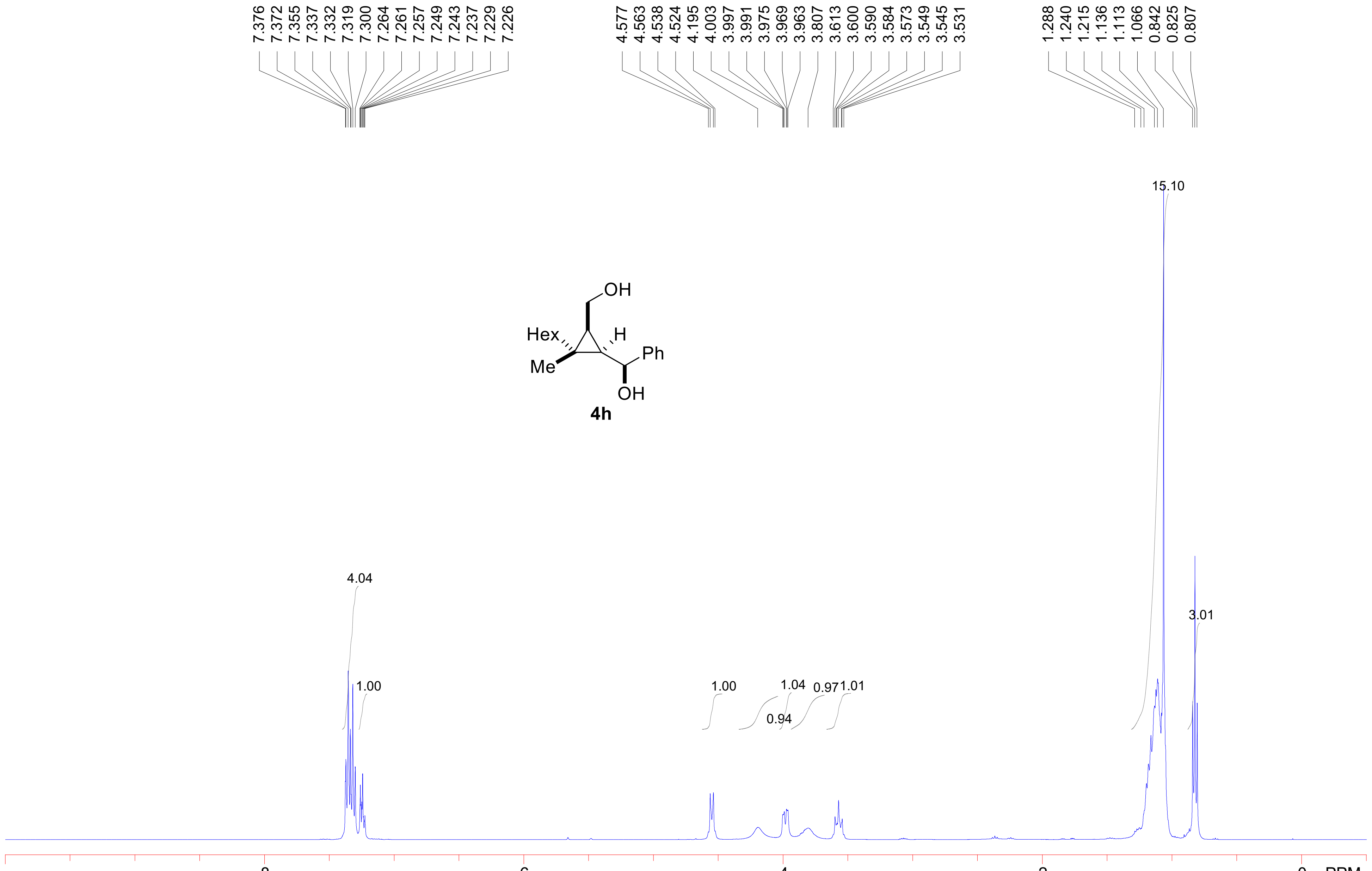
7.376
7.372
7.355
7.337
7.332
7.319
7.300
7.264
7.261
7.257
7.249
7.243
7.237
7.229
7.226
4.577
4.563
4.538
4.524
4.195
4.003
3.997
3.991
3.975
3.969
3.963
3.807
3.613
3.600
3.590
3.584
3.573
3.549
3.545
3.531
1.288
1.240
1.215
1.136
1.113
1.066
0.842
0.825
0.807
15.10
OH
Hex
H
Ph
Me
OH
4h
4.04
1.00
1.00
1.04
0.94
0.97
1.01
3.01
8
6
4
2
0
PPM

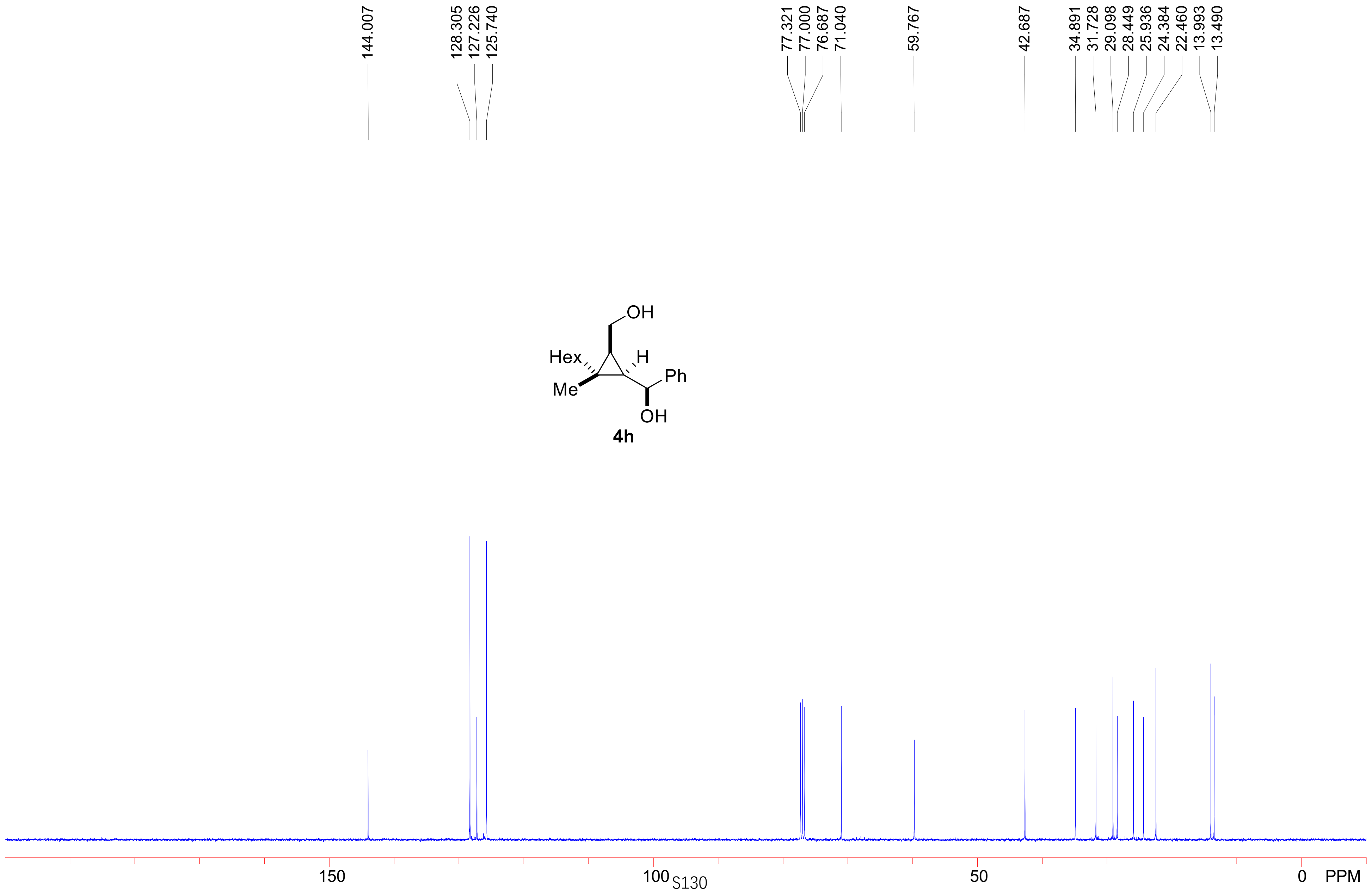
144.007
128.305
127.226
125.740
77.321
77.000
76.687
71.040
59.767
42.687
34.891
31.728
29.098
28.449
25.936
24.384
22.460
13.993
13.490
OH
Hex
H
Ph
Me
OH
4h
150
100
50
0
PPM

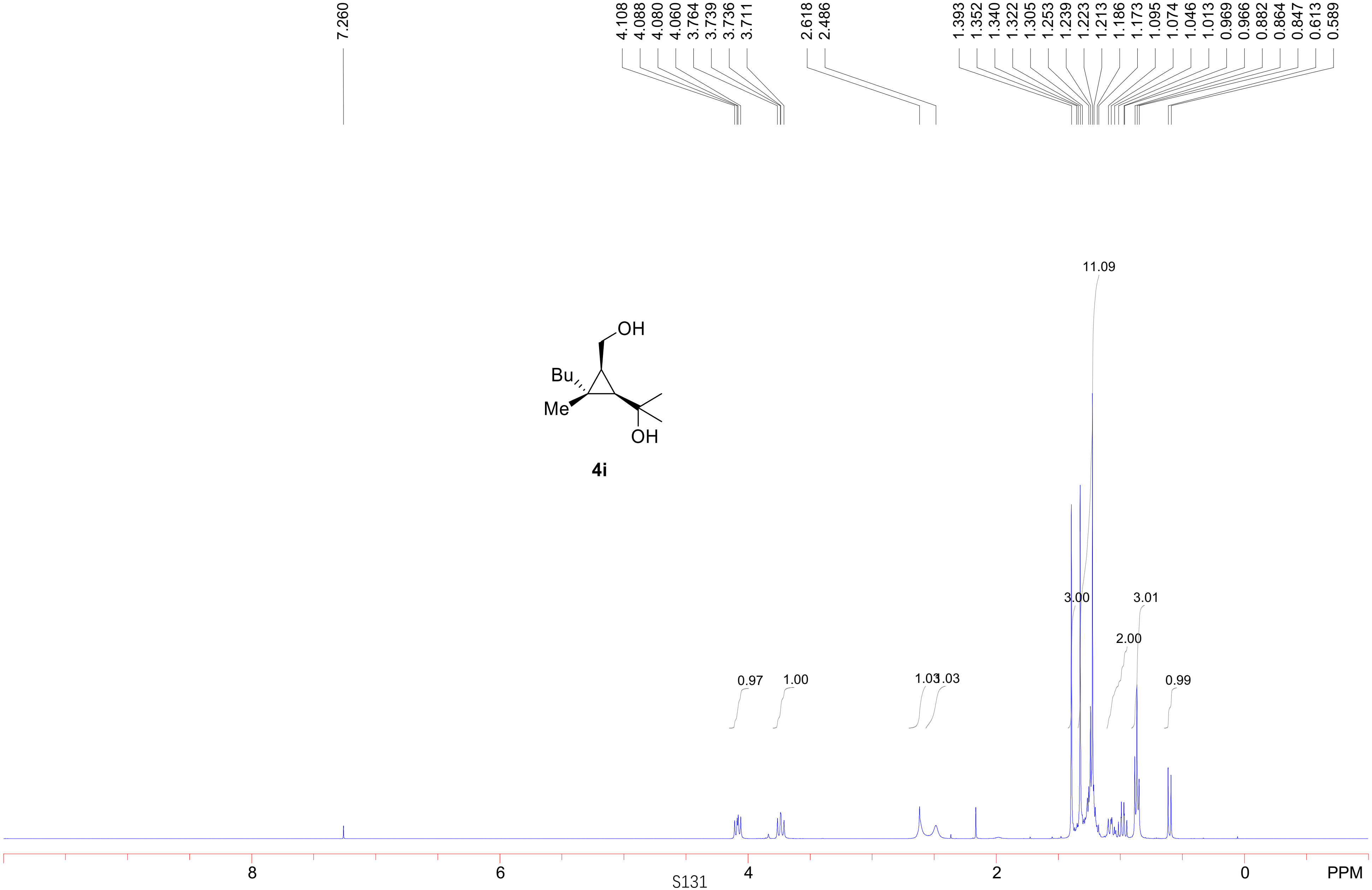
7.260
4.108
4.088
4.080
4.060
3.764
3.739
3.736
3.711
2.618
2.486
1.393
1.352
1.340
1.322
1.305
1.253
1.239
1.223
1.213
1.186
1.173
1.095
1.074
1.046
1.013
0.969
0.966
0.882
0.864
0.847
0.613
0.589
OH
Bu
Me
OH
4i
11.09
3.00
3.01
2.00
0.97
1.00
1.03
1.03
0.99
8
6
4
2
0
PPM

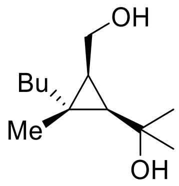
OH
Bu
Me
OH

4i

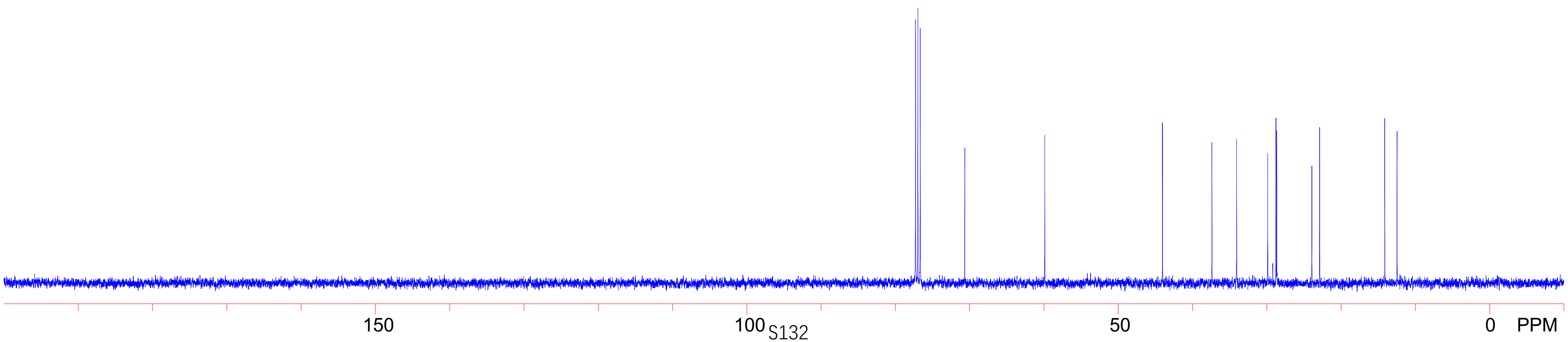
77.321
77.000
76.679
70.690
59.913
44.057
37.397
34.075
29.885
28.785
28.675
23.939
22.890
14.124
12.477
150
100
50
0
PPM

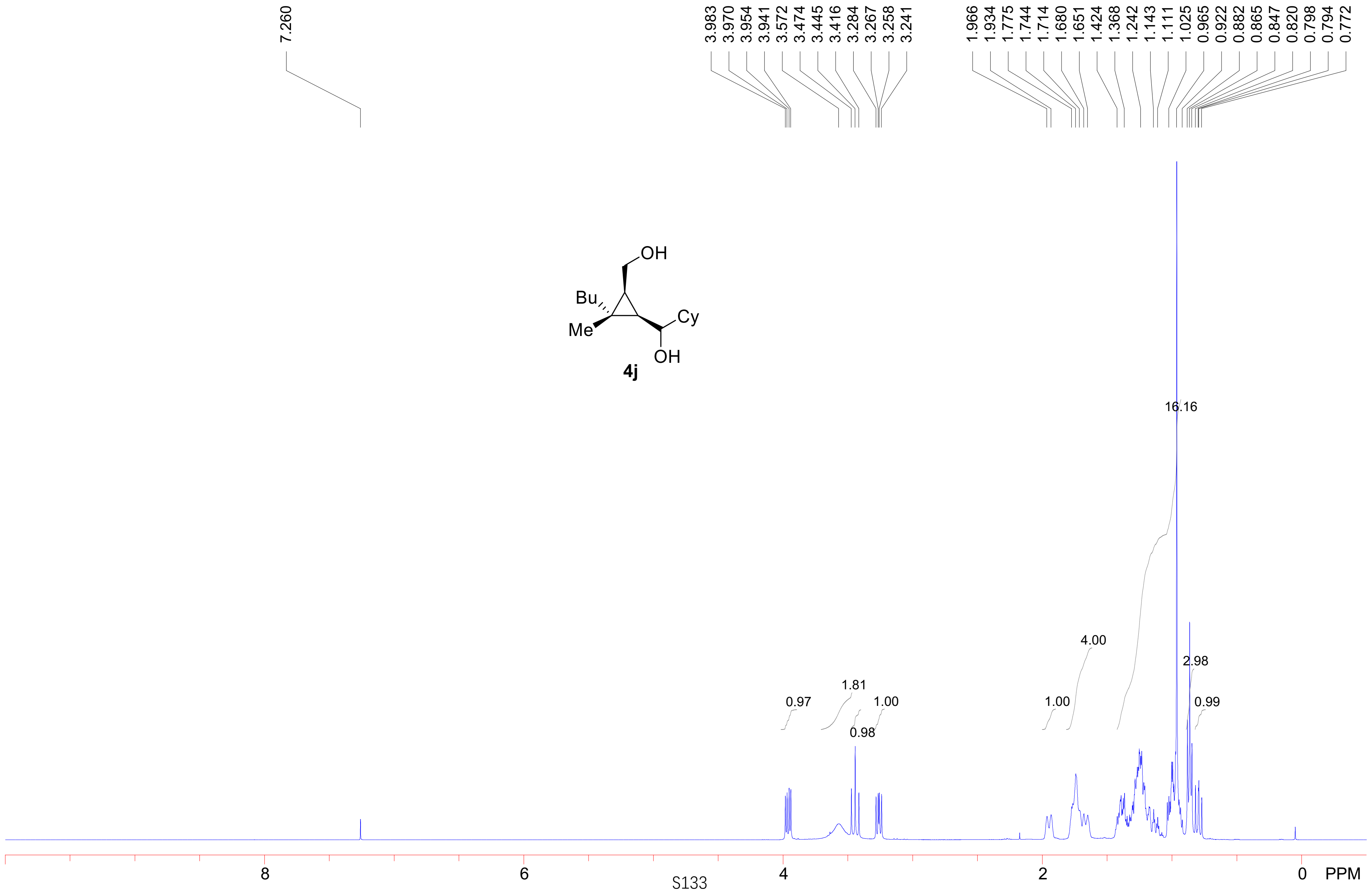
7.260
3.983
3.970
3.954
3.941
3.572
3.474
3.445
3.416
3.284
3.267
3.258
3.241
1.966
1.934
1.775
1.744
1.714
1.680
1.651
1.424
1.368
1.242
1.143
1.111
1.025
0.965
0.922
0.882
0.865
0.847
0.820
0.798
0.794
0.772
OH
Bu
Cy
Me
OH
4j
16.16
4.00
2.98
1.81
0.97
1.00
0.99
1.00
0.98
8
6
4
2
0
PPM

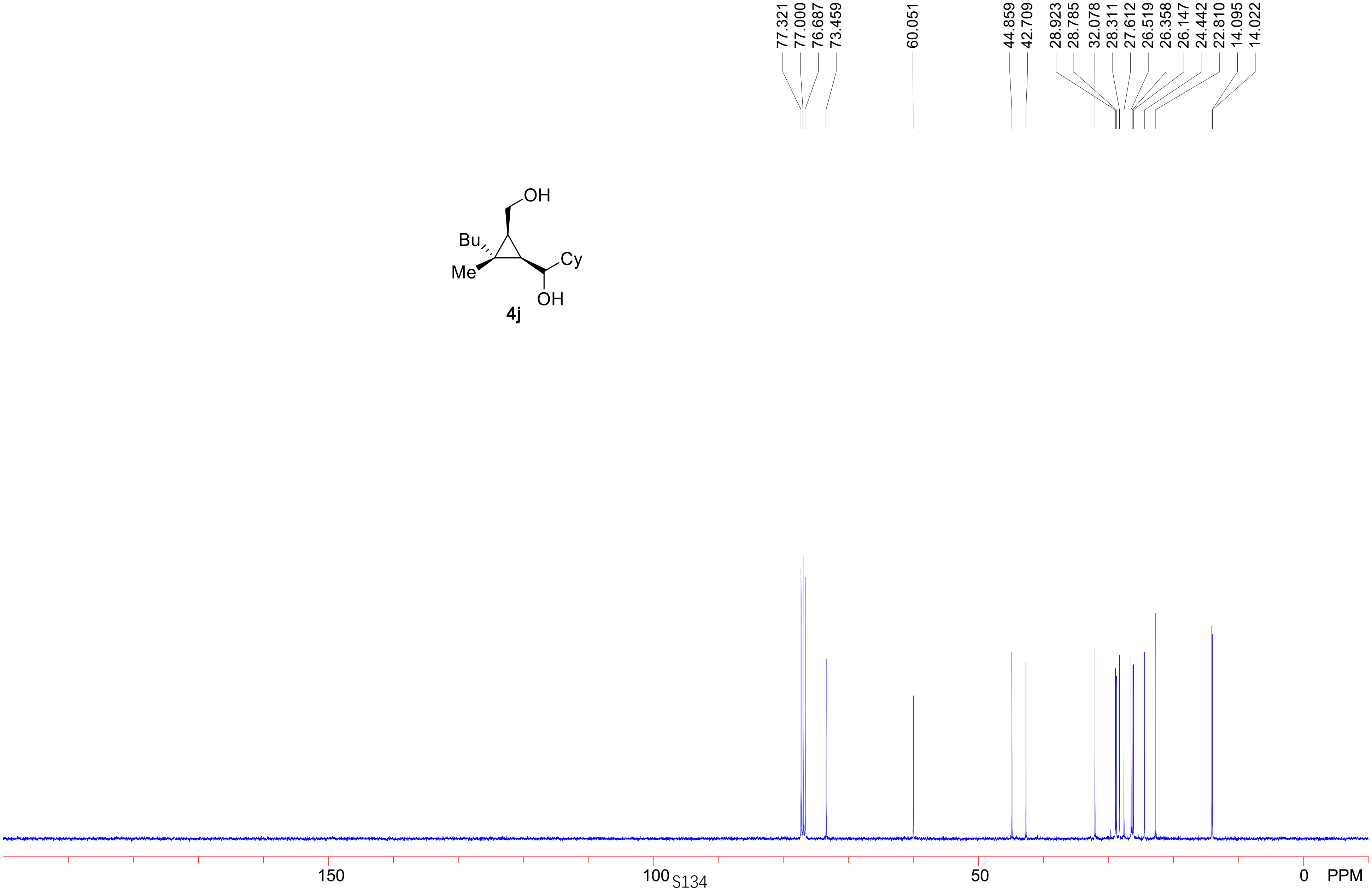
77.321
77.000
76.687
73.459
60.051
44.859
42.709
28.923
28.785
32.078
28.311
27.612
26.519
26.358
26.147
24.442
22.810
14.095
14.022
OH
Bu
Me
Cy
OH
150
100
50
0
PPM

4j

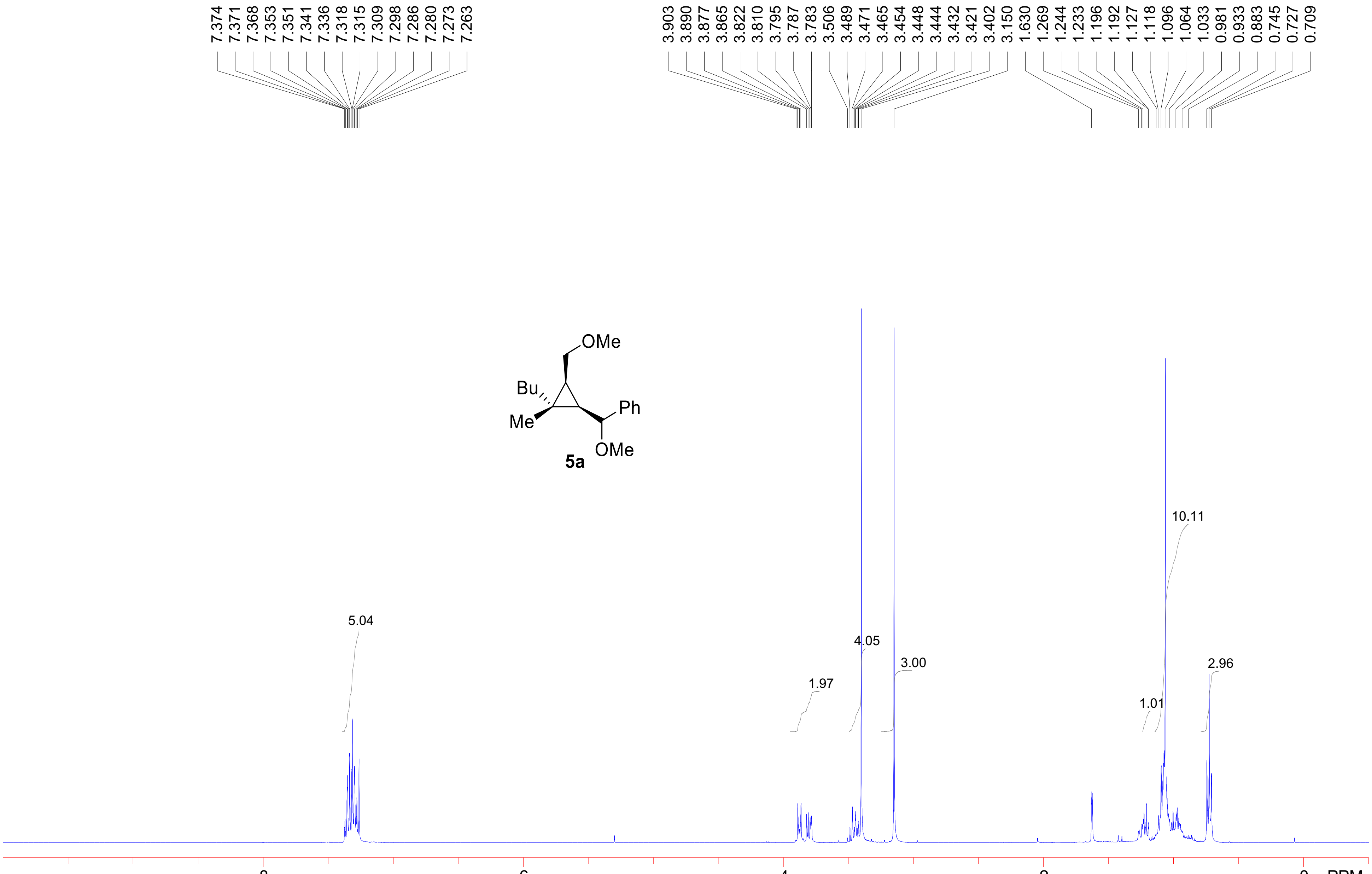
7.374
7.371
7.368
7.353
7.351
7.341
7.336
7.318
7.315
7.309
7.298
7.286
7.280
7.273
7.263
3.903
3.890
3.877
3.865
3.822
3.810
3.795
3.787
3.783
3.506
3.489
3.471
3.465
3.454
3.448
3.444
3.432
3.421
3.402
3.150
1.630
1.269
1.244
1.233
1.196
1.192
1.127
1.118
1.096
1.064
1.033
0.981
0.933
0.883
0.745
0.727
0.709
OMe
Bu
Ph
Me
OMe
5a
5.04
1.97
4.05
3.00
1.01
10.11
2.96
8
6
4
2
0
PPM

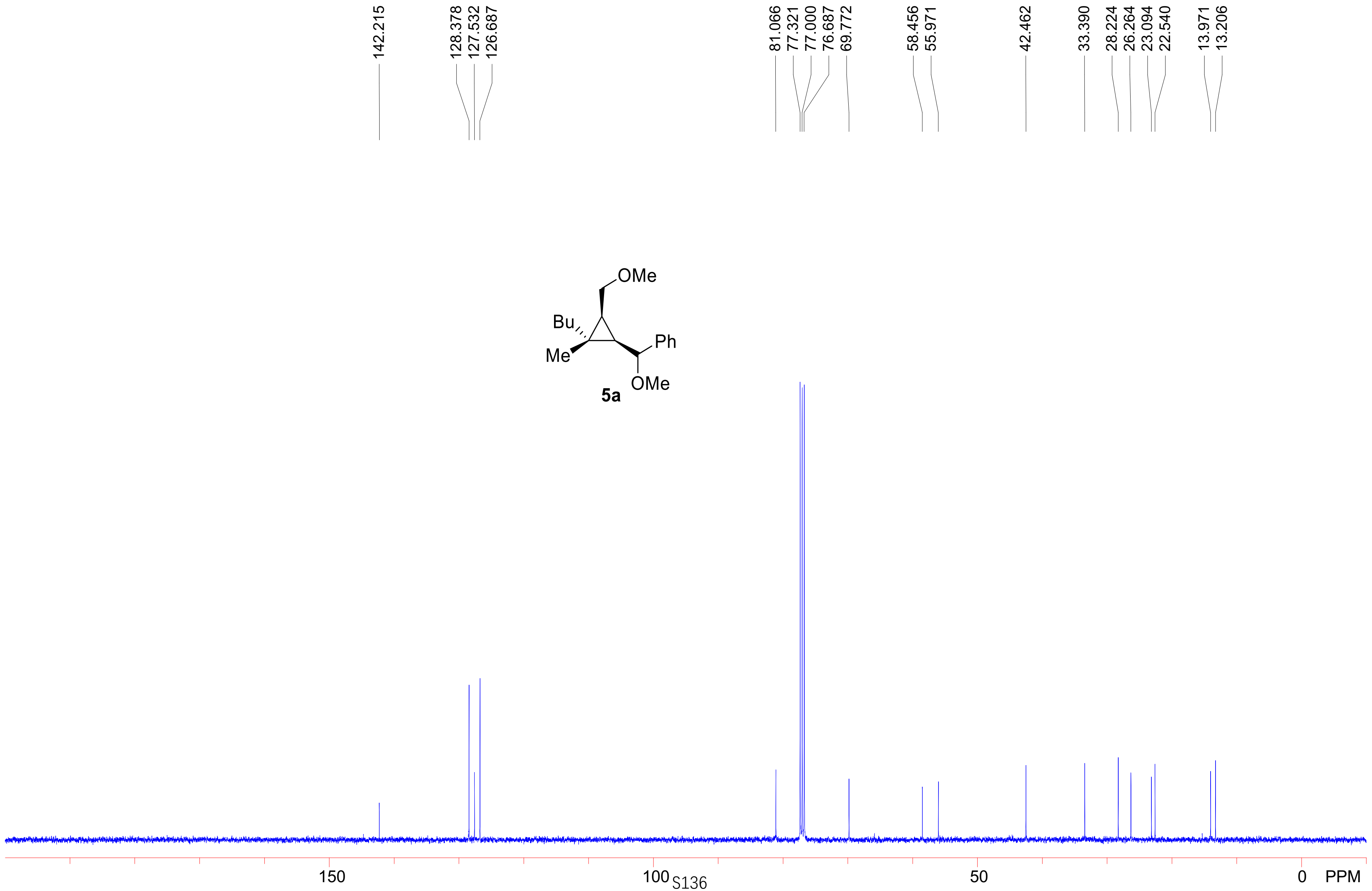
142.215
128.378
127.532
126.687
81.066
77.321
77.000
76.687
69.772
58.456
55.971
42.462
33.390
28.224
26.264
23.094
22.540
13.971
13.206
OMe
Bu
Ph
Me
OMe
5a
150
100
50
0
PPM

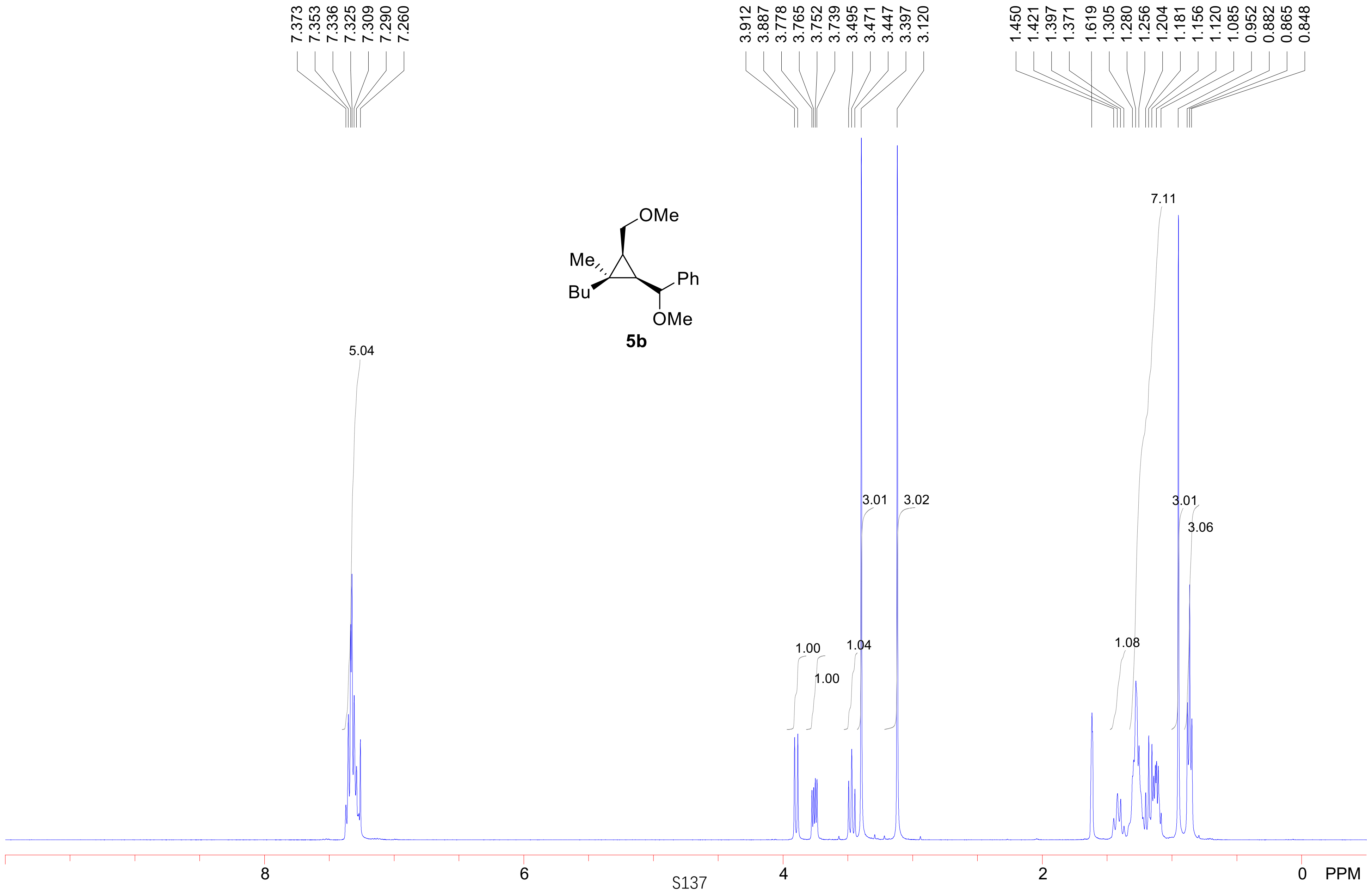
7.373
7.353
7.336
7.325
7.309
7.290
7.260
3.912
3.887
3.778
3.765
3.752
3.739
3.495
3.471
3.447
3.397
3.120
1.450
1.421
1.397
1.371
1.619
1.305
1.280
1.256
1.204
1.181
1.156
1.120
1.085
0.952
0.882
0.865
0.848
OMe
Me
Bu
Ph
OMe
5b
5.04
1.00
1.00
1.04
3.01
3.02
1.08
7.11
3.01
3.06
8
6
4
2
0
PPM

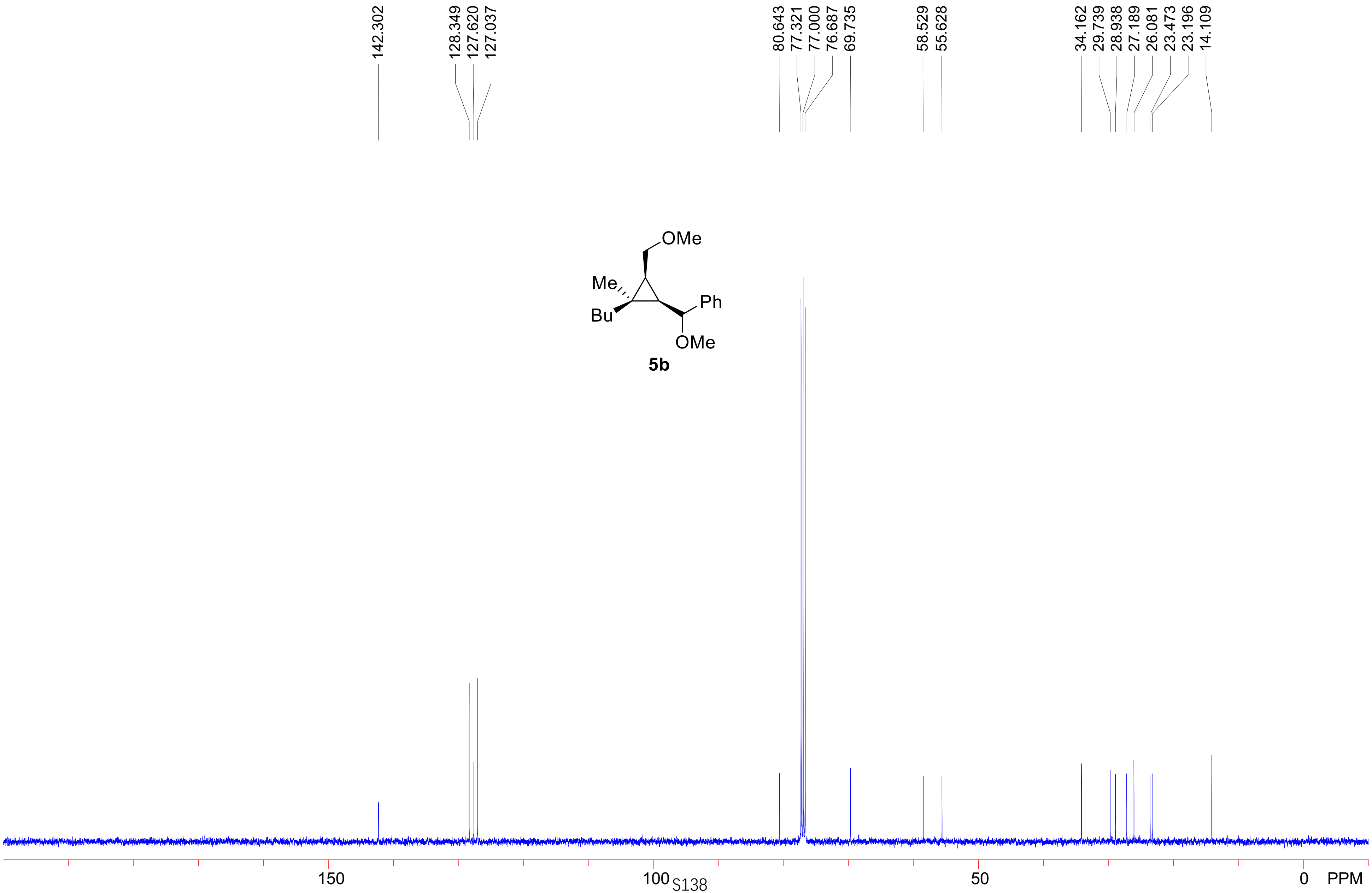
142.302
128.349
127.620
127.037
80.643
77.321
77.000
76.687
69.735
58.529
55.628
34.162
29.739
28.938
27.189
26.081
23.473
23.196
14.109
OMe
Me
Bu
Ph
OMe
5b
150
100
50
0
PPM

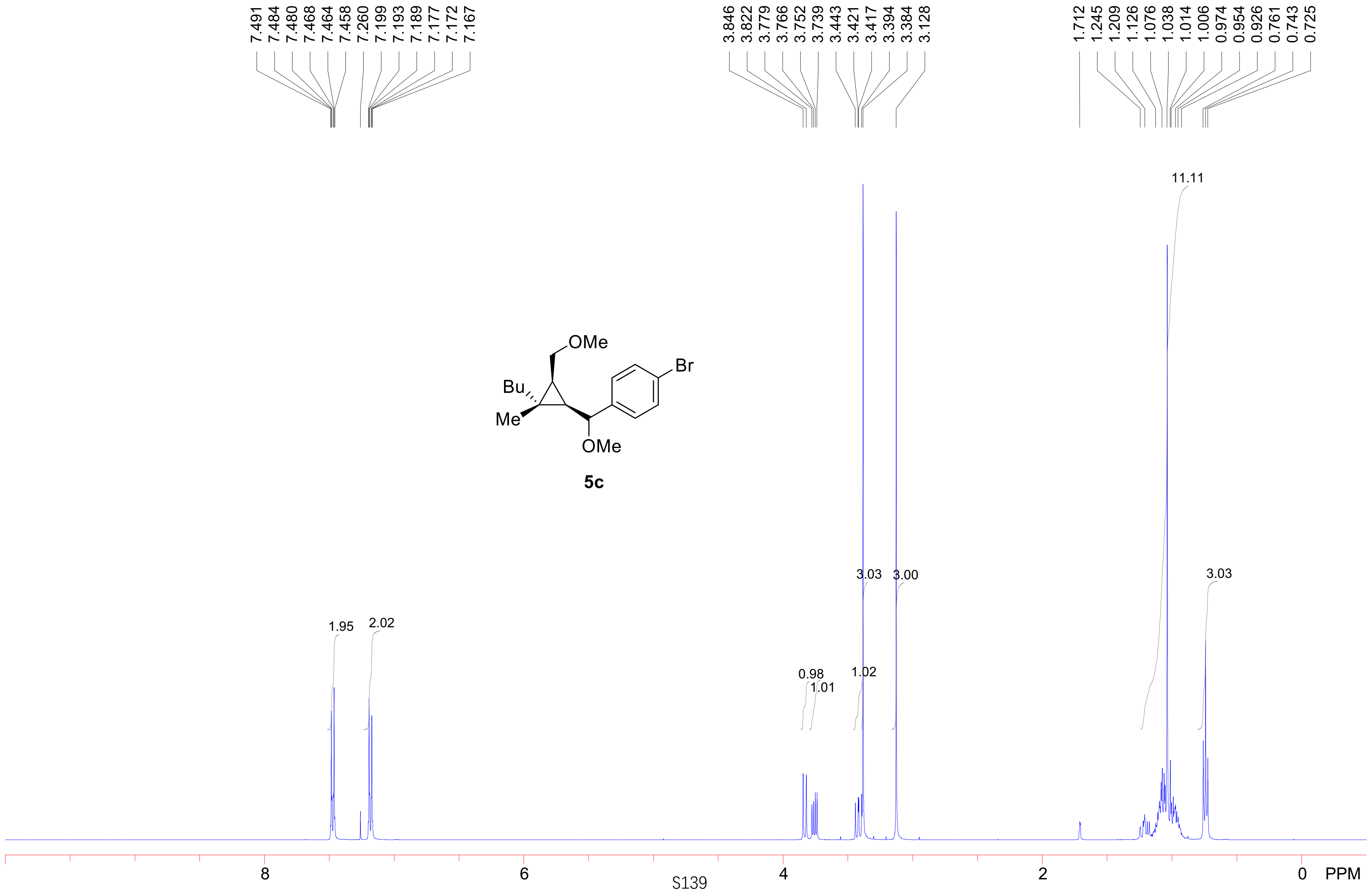
7.491
7.484
7.480
7.468
7.464
7.458
7.260
7.199
7.193
7.189
7.177
7.172
7.167
3.846
3.822
3.779
3.766
3.752
3.739
3.443
3.421
3.417
3.394
3.384
3.128
1.712
1.245
1.209
1.126
1.076
1.038
1.014
1.006
0.974
0.954
0.926
0.761
0.743
0.725
OMe
Br
Bu
Me
OMe
5c
11.11
3.03
3.00
3.03
1.95
2.02
0.98
1.01
1.02
8
6
4
2
0
PPM

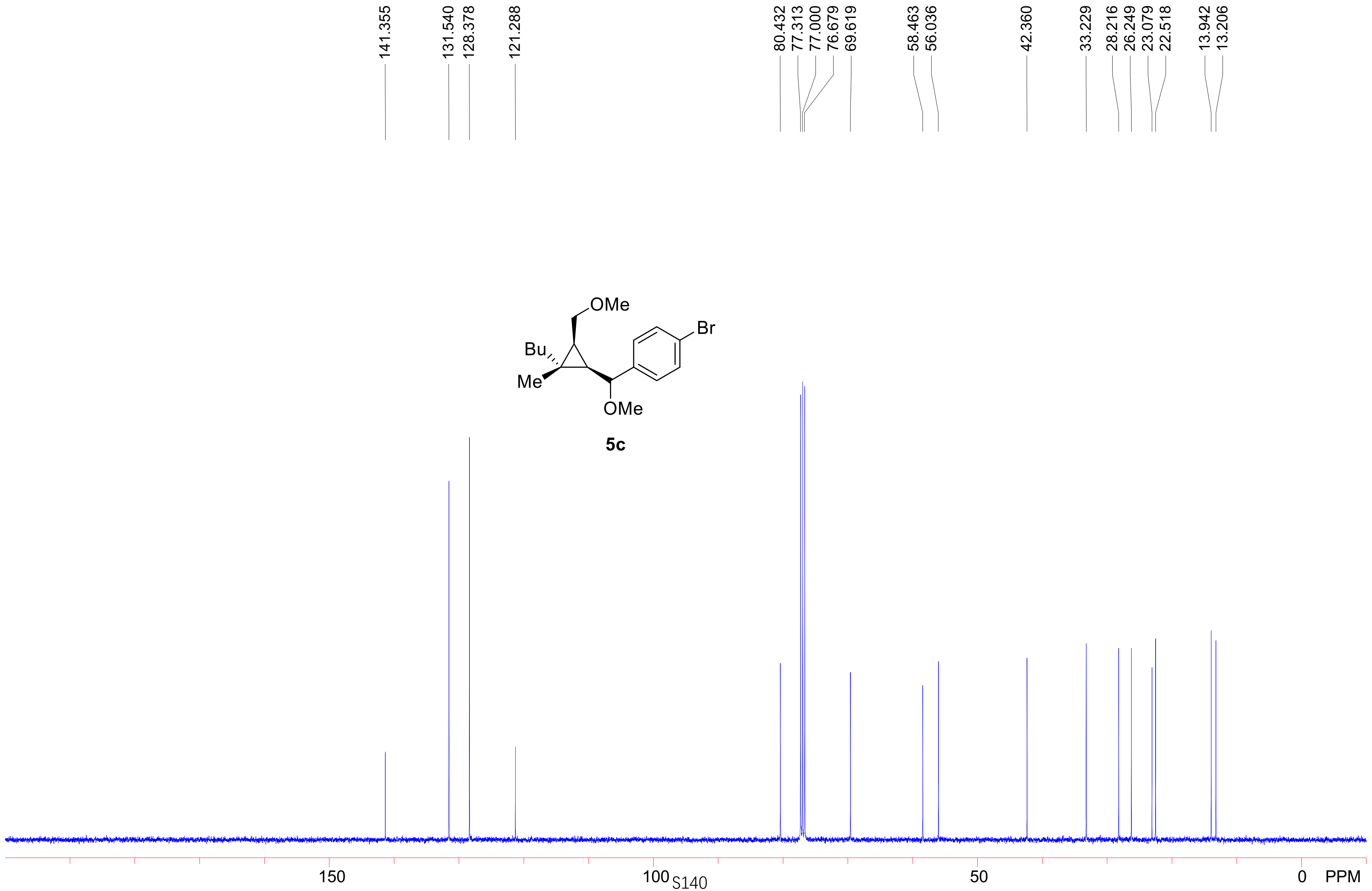
141.355
131.540
128.378
121.288
80.432
77.313
77.000
76.679
69.619
58.463
56.036
42.360
33.229
28.216
26.249
23.079
22.518
13.942
13.206
OMe
Br
Bu
Me
OMe
5c
150
100
50
0
PPM

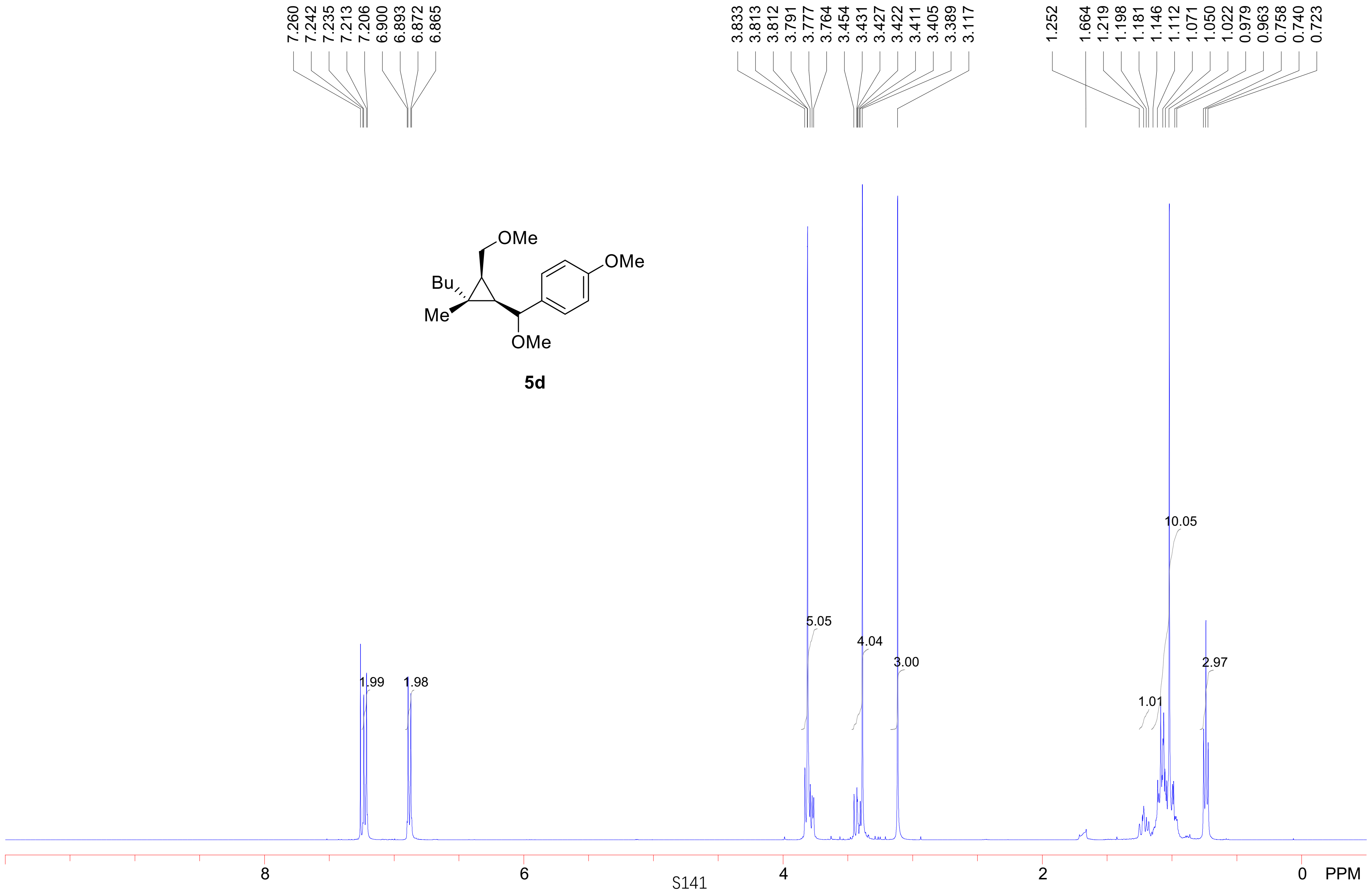
7.260
7.242
7.235
7.213
7.206
6.900
6.893
6.872
6.865
3.833
3.813
3.812
3.791
3.777
3.764
3.454
3.431
3.427
3.422
3.411
3.405
3.389
3.117
1.252
1.664
1.219
1.198
1.181
1.146
1.112
1.071
1.050
1.022
0.979
0.963
0.758
0.740
0.723
OMe
OMe
Bu
Me
OMe
5d
1.99
1.98
5.05
4.04
3.00
10.05
1.01
2.97
8
6
4
2
0
PPM

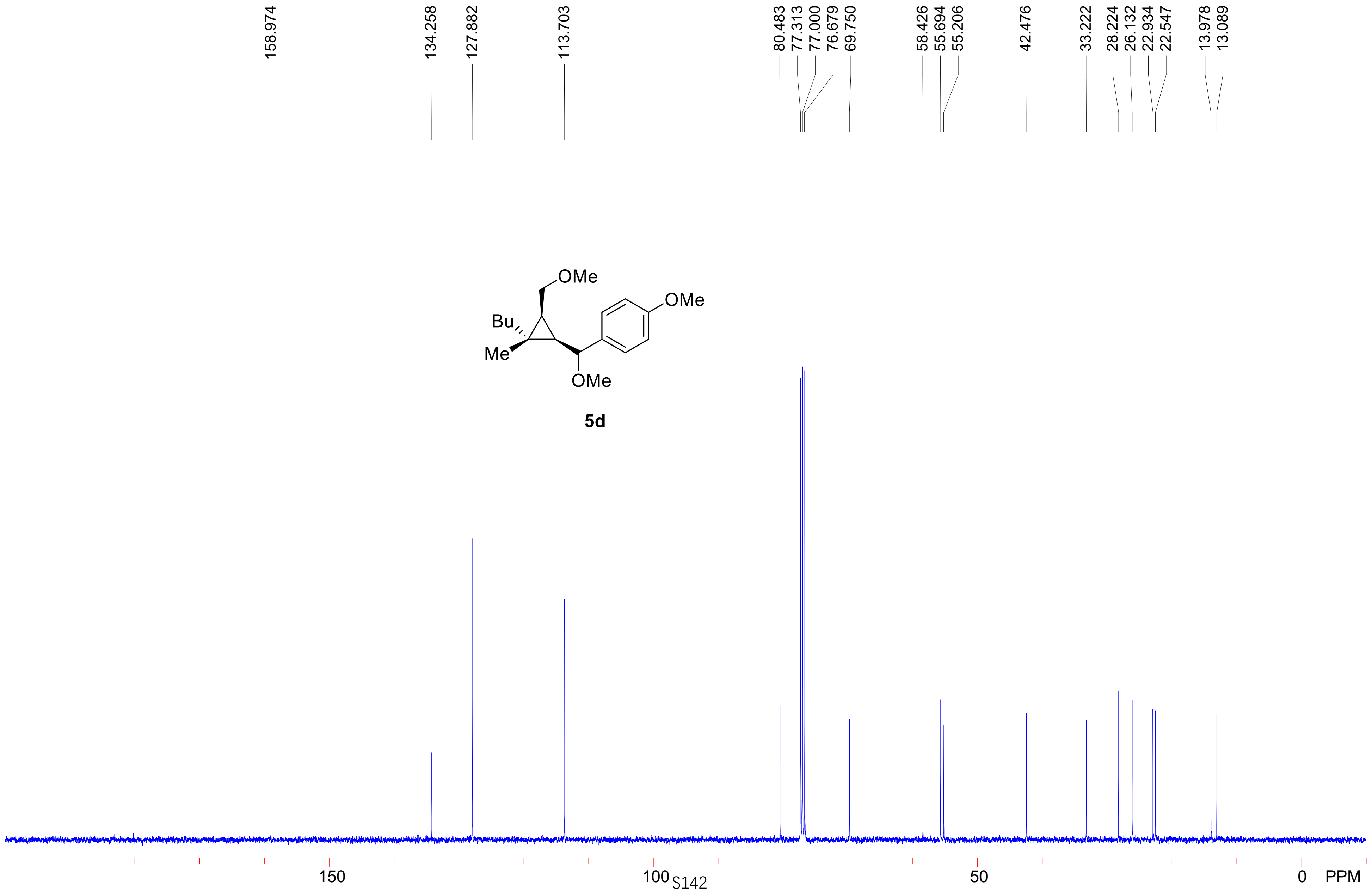
OMe
OMe
Bu
Me
OMe
158.974
134.258
127.882
113.703
80.483
77.313
77.000
76.679
69.750
58.426
55.694
55.206
42.476
33.222
28.224
26.132
22.934
22.547
13.978
13.089
150
100
50
0
PPM

5d

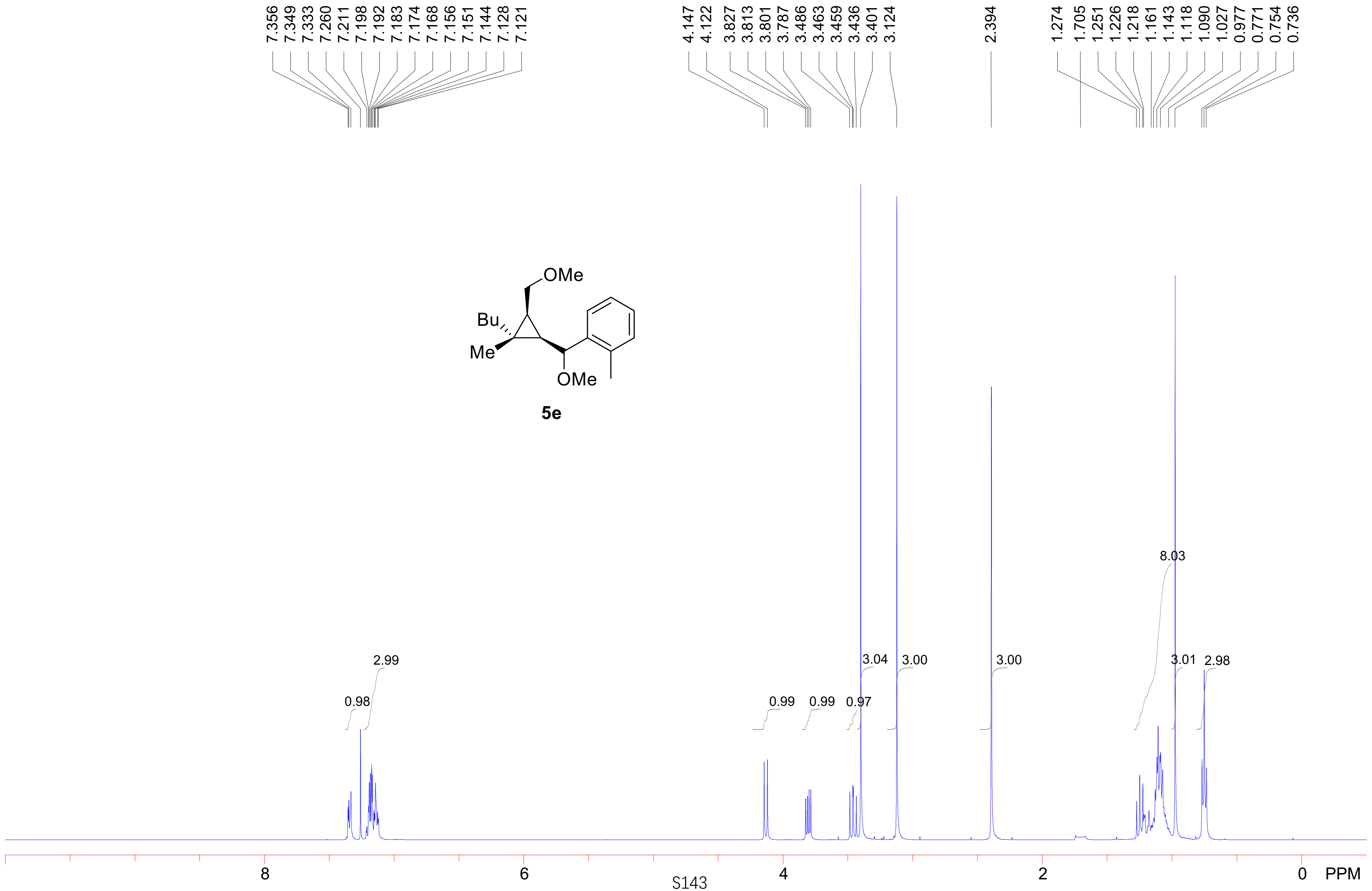
7.356
7.349
7.333
7.260
7.211
7.198
7.192
7.183
7.174
7.168
7.156
7.151
7.144
7.128
7.121
4.147
4.122
3.827
3.813
3.801
3.787
3.486
3.463
3.459
3.436
3.401
3.124
2.394
1.274
1.705
1.251
1.226
1.218
1.161
1.143
1.118
1.090
1.027
0.977
0.771
0.754
0.736
OMe
Bu
Me
OMe
5e
0.98
2.99
0.99
0.99
0.97
3.04
3.00
3.00
8.03
3.01
2.98
8
6
4
2
0
PPM

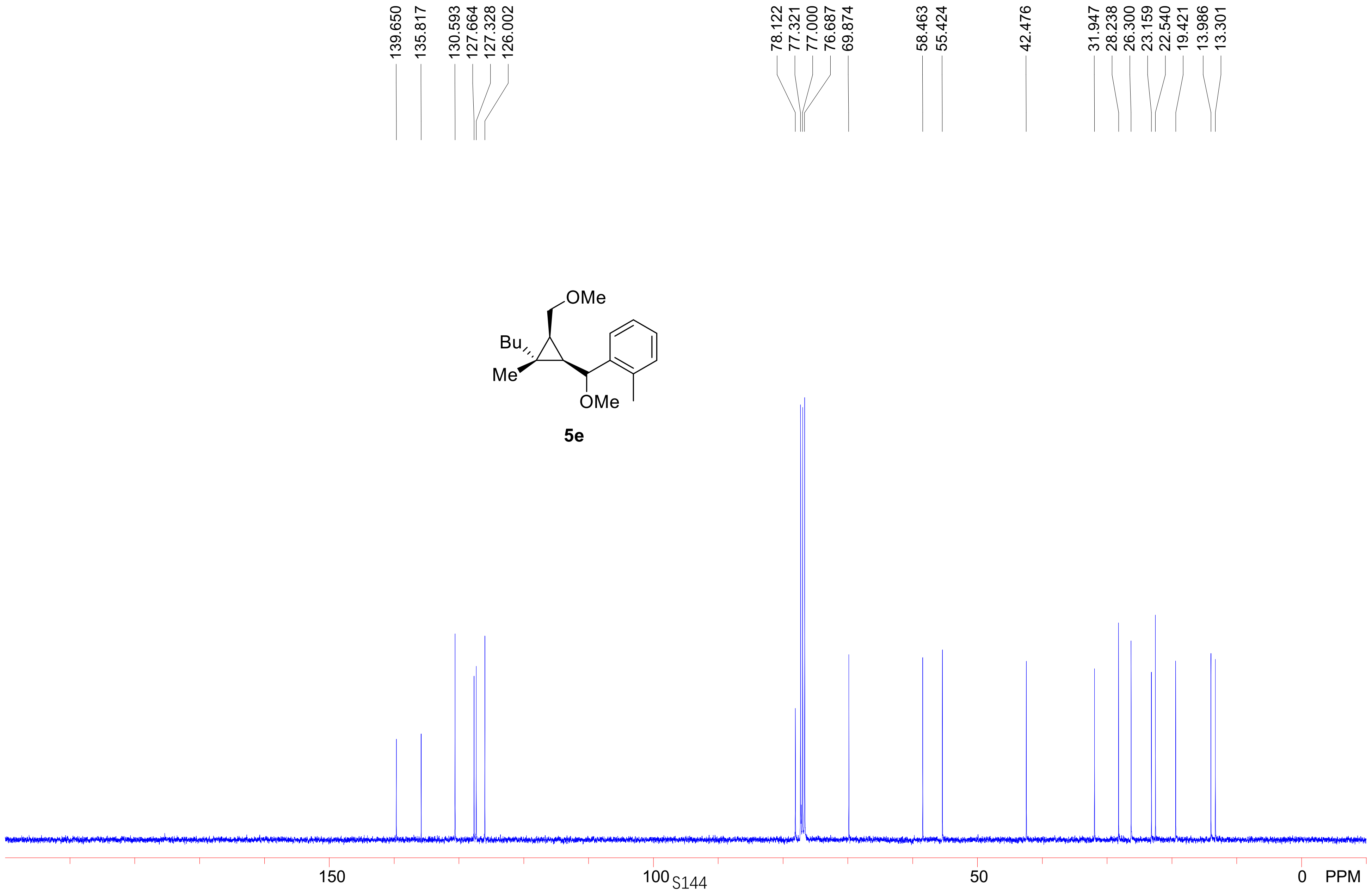
139.650
135.817
130.593
127.664
127.328
126.002
78.122
77.321
77.000
76.687
69.874
58.463
55.424
42.476
31.947
28.238
26.300
23.159
22.540
19.421
13.986
13.301
OMe
Bu
Me
OMe
5e
150
100
50
0
PPM

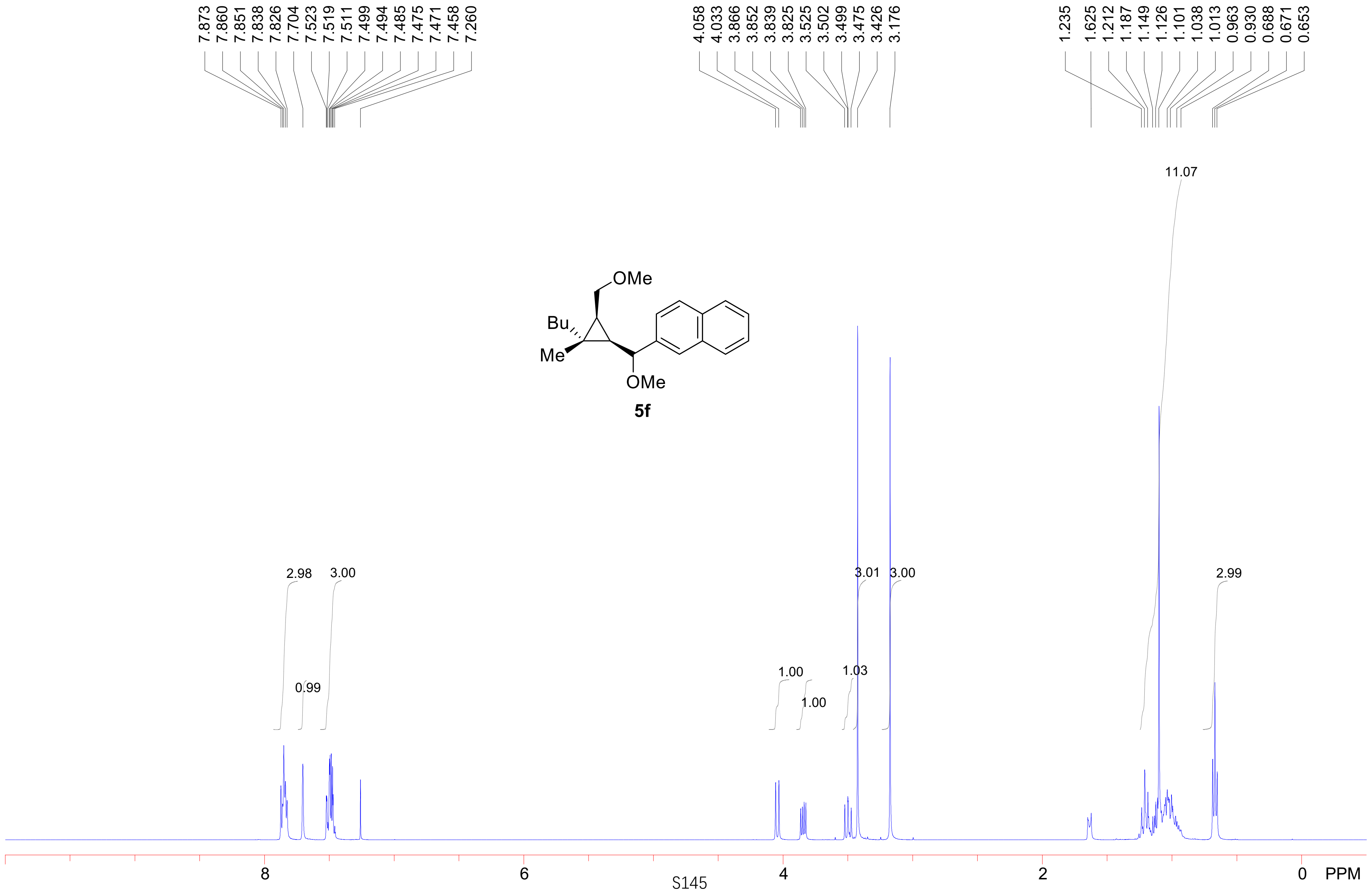
7.873
7.860
7.851
7.838
7.826
7.704
7.523
7.519
7.511
7.499
7.494
7.485
7.475
7.471
7.458
7.260
4.058
4.033
3.866
3.852
3.839
3.825
3.525
3.502
3.499
3.475
3.426
3.176
1.235
1.625
1.212
1.187
1.149
1.126
1.101
1.038
1.013
0.963
0.930
0.688
0.671
0.653
11.07
OMe
Bu
Me
OMe
5f
2.98
3.00
0.99
1.00
1.00
1.03
3.01
3.00
2.99
8
6
4
2
0
PPM

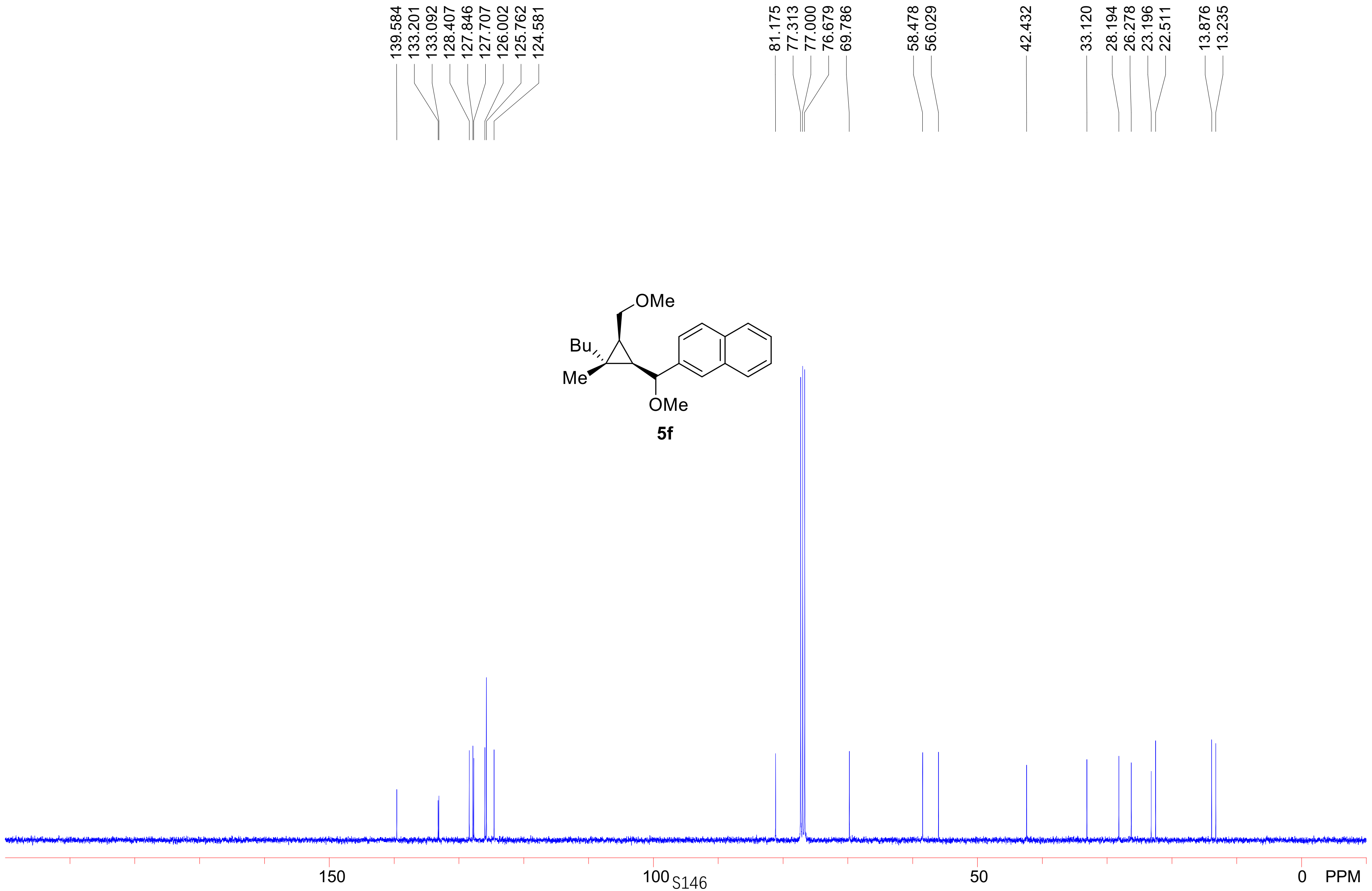

139.584
133.201
133.092
128.407
127.846
127.707
126.002
125.762
124.581
81.175
77.313
77.000
76.679
69.786
58.478
56.029
42.432
33.120
28.194
26.278
23.196
22.511
13.876
13.235
OMe
Bu
Me
OMe
5f
150
100
50
0
PPM

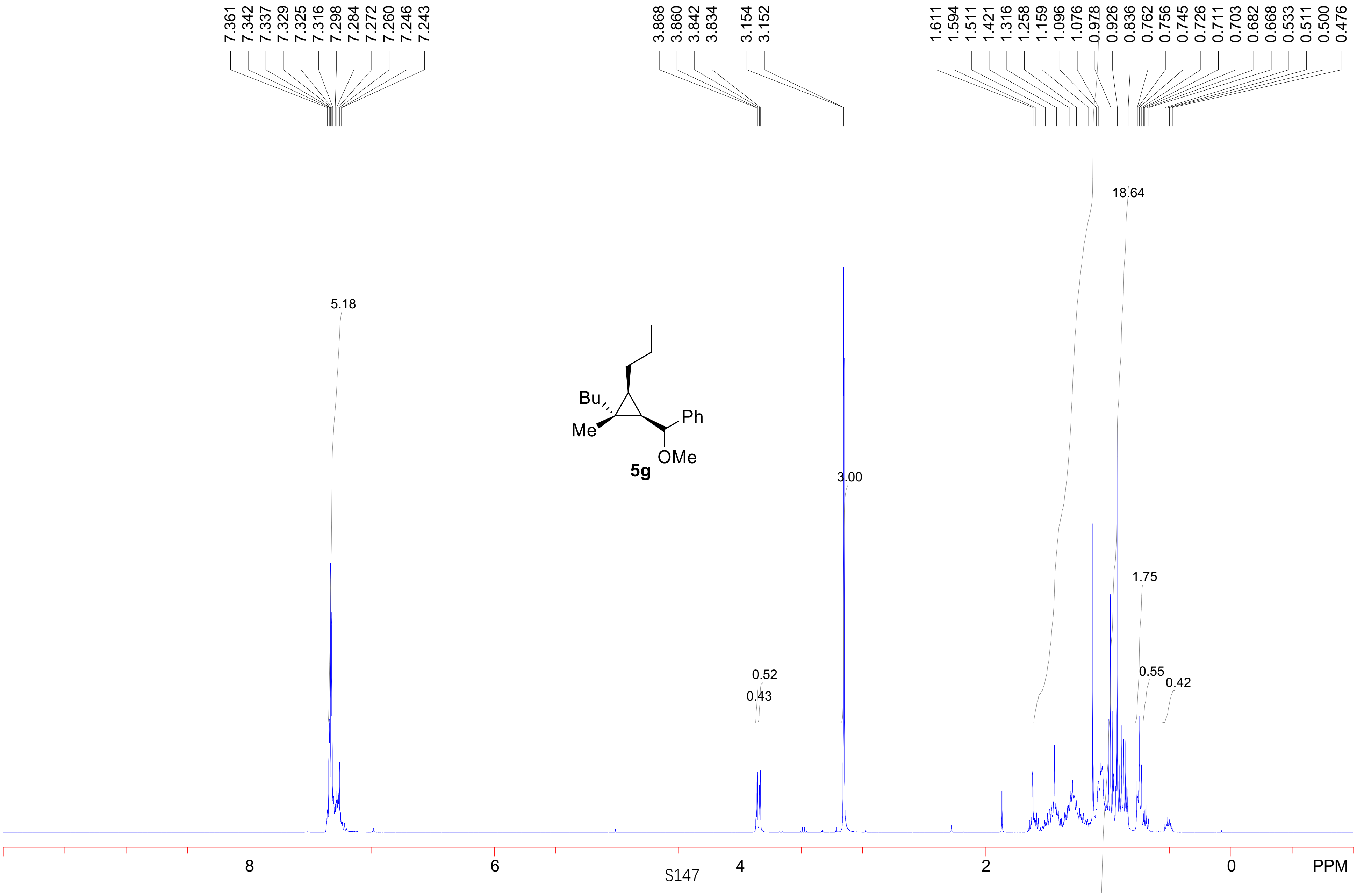
7.361
7.342
7.337
7.329
7.325
7.316
7.298
7.284
7.272
7.260
7.246
7.243
3.868
3.860
3.842
3.834
3.154
3.152
1.611
1.594
1.511
1.421
1.316
1.258
1.159
1.096
1.076
0.978
0.926
0.836
0.762
0.756
0.745
0.726
0.711
0.703
0.682
0.668
0.533
0.511
0.500
0.476
5.18
0.52
0.43
3.00
18.64
1.75
0.55
0.42
Bu
Me
Ph
OMe
5g
8
6
4
2
0
PPM

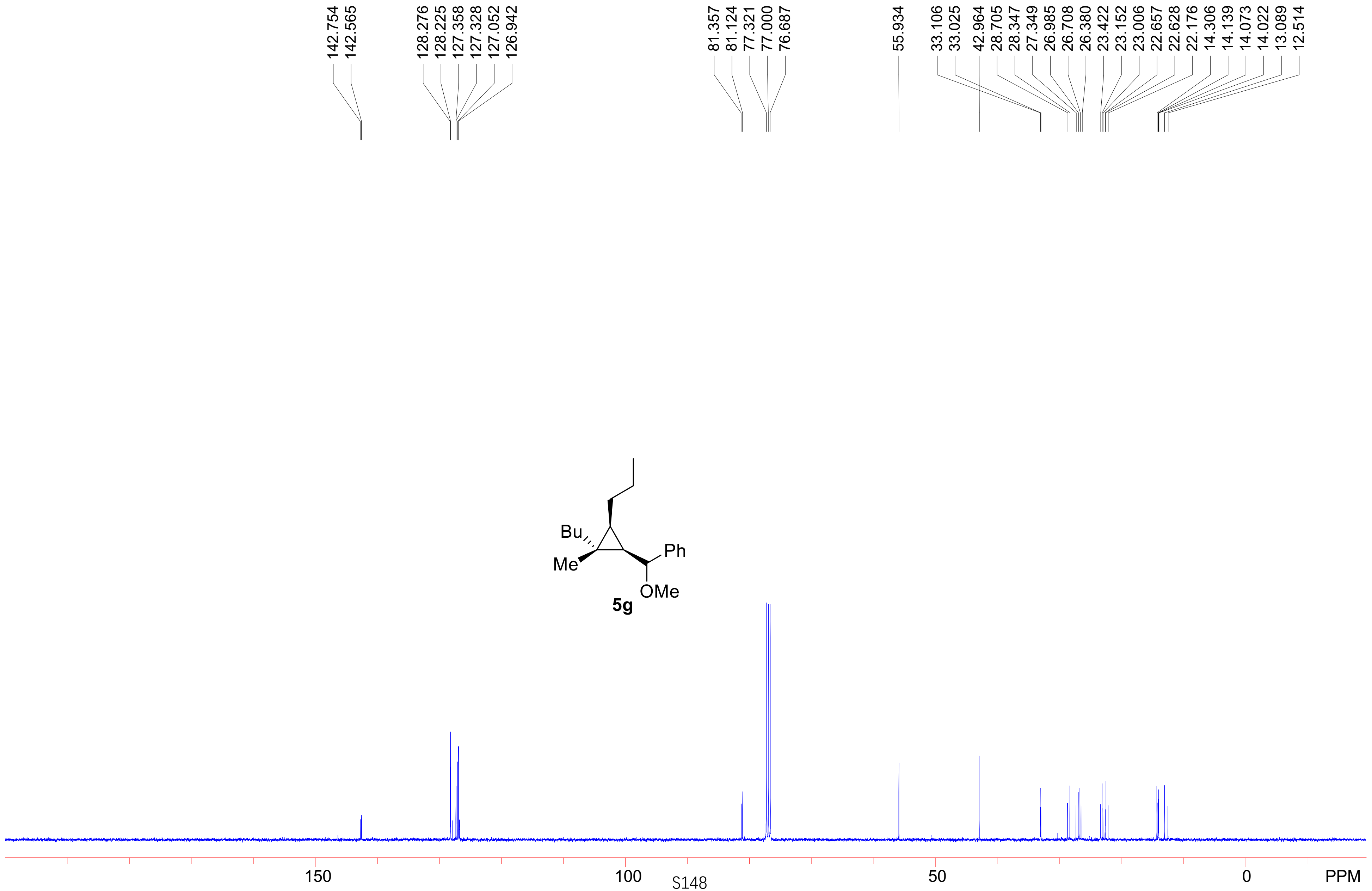
142.754
142.565
128.276
128.225
127.358
127.328
127.052
126.942
81.357
81.124
77.321
77.000
76.687
55.934
33.106
33.025
42.964
28.705
28.347
27.349
26.985
26.708
26.380
23.422
23.152
23.006
22.657
22.628
22.176
14.306
14.139
14.073
14.022
13.089
12.514
Bu
Me
Ph
OMe
5g
150
100
50
0
PPM

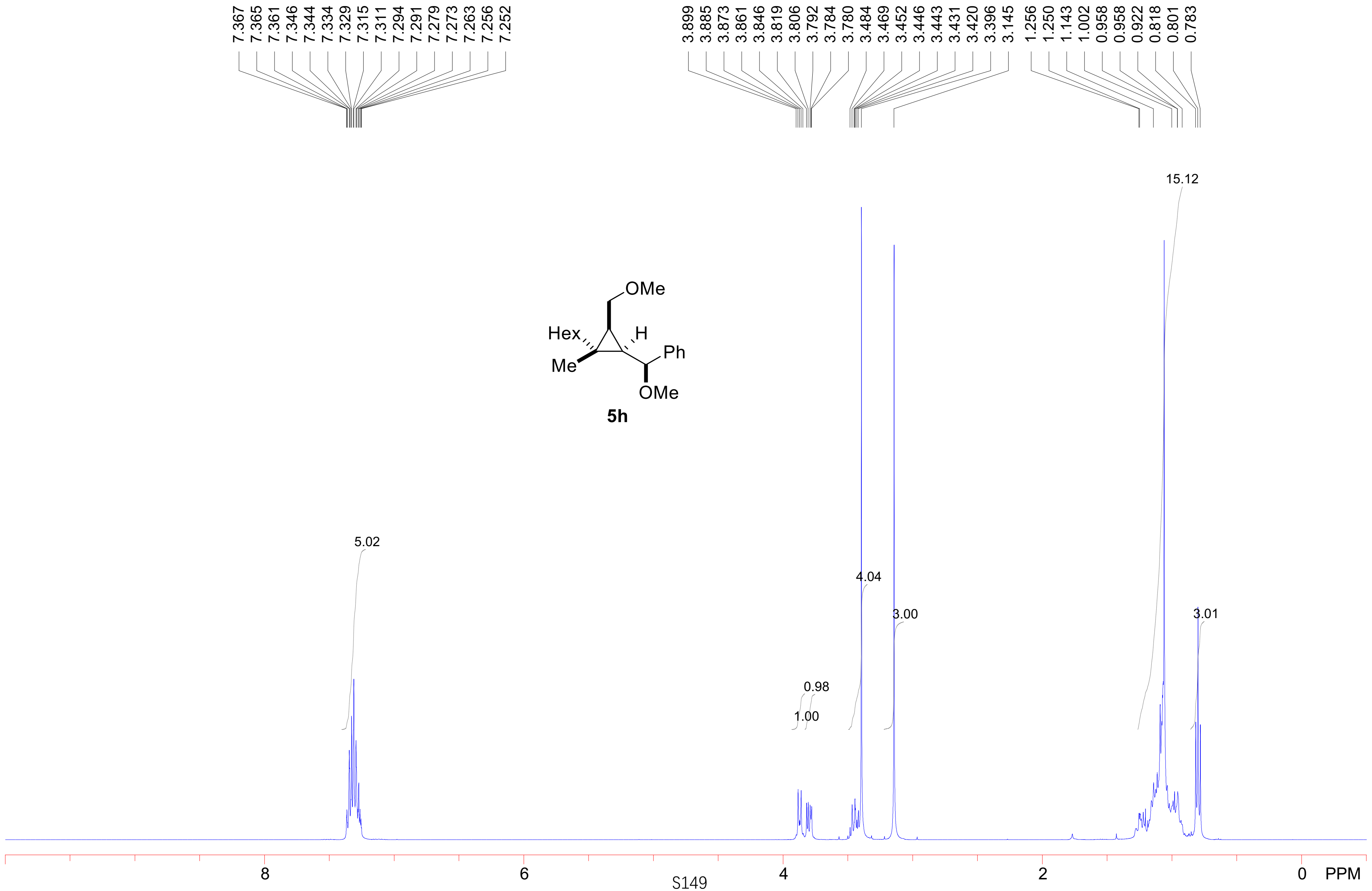

7.367
7.365
7.361
7.346
7.344
7.334
7.329
7.315
7.311
7.294
7.291
7.279
7.273
7.263
7.256
7.252
3.899
3.885
3.873
3.861
3.846
3.819
3.806
3.792
3.784
3.780
3.484
3.469
3.452
3.446
3.443
3.431
3.420
3.396
3.145
1.256
1.250
1.143
1.002
0.958
0.958
0.922
0.818
0.801
0.783
15.12
OMe
Hex
H
Ph
Me
OMe
5h
5.02
4.04
3.00
3.01
0.98
1.00
8
6
4
2
0
PPM

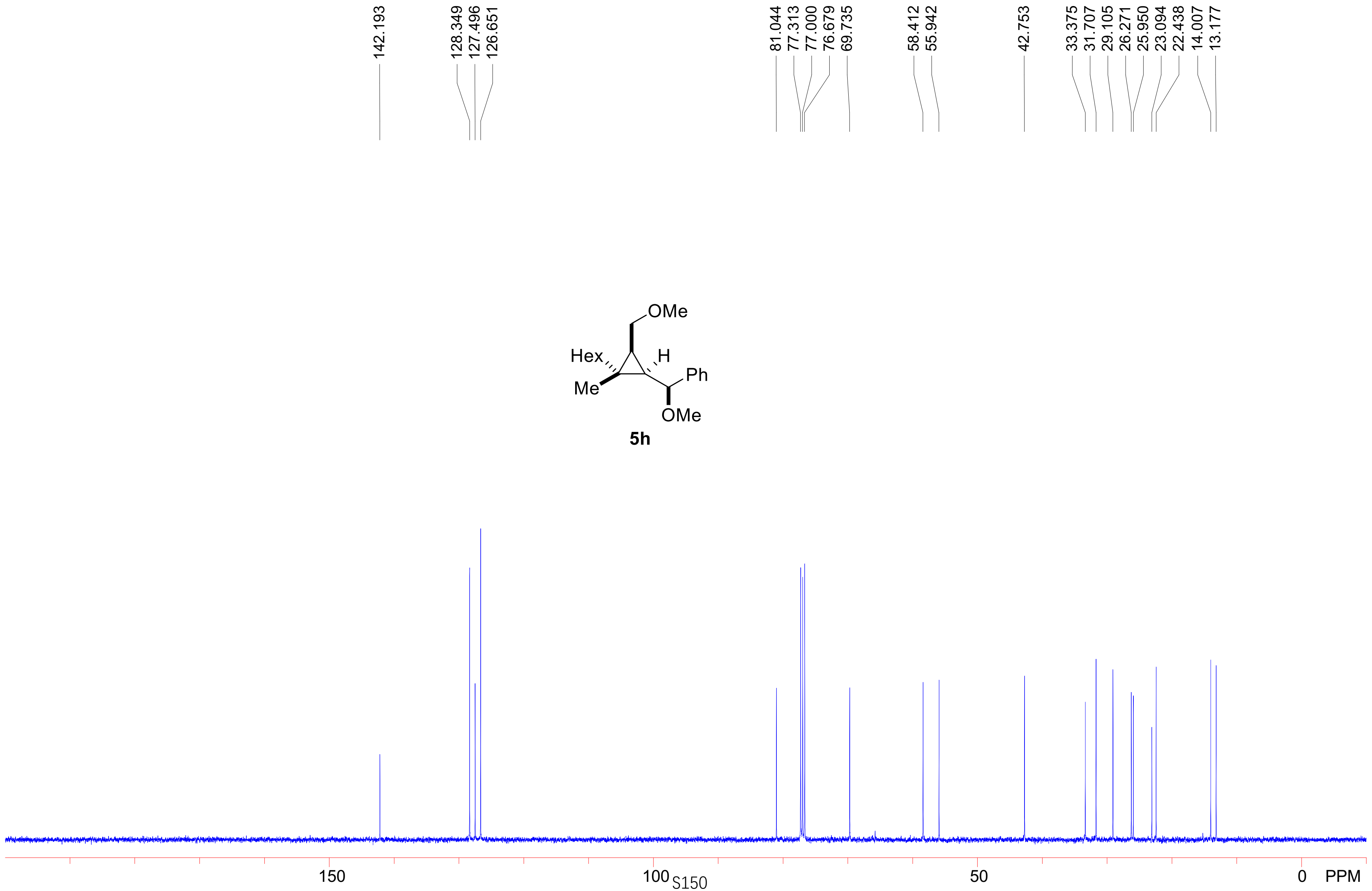
142.193
128.349
127.496
126.651
81.044
77.313
77.000
76.679
69.735
58.412
55.942
42.753
33.375
31.707
29.105
26.271
25.950
23.094
22.438
14.007
13.177
OMe
Hex
H
Ph
Me
OMe
150
100
50
0
PPM

5h

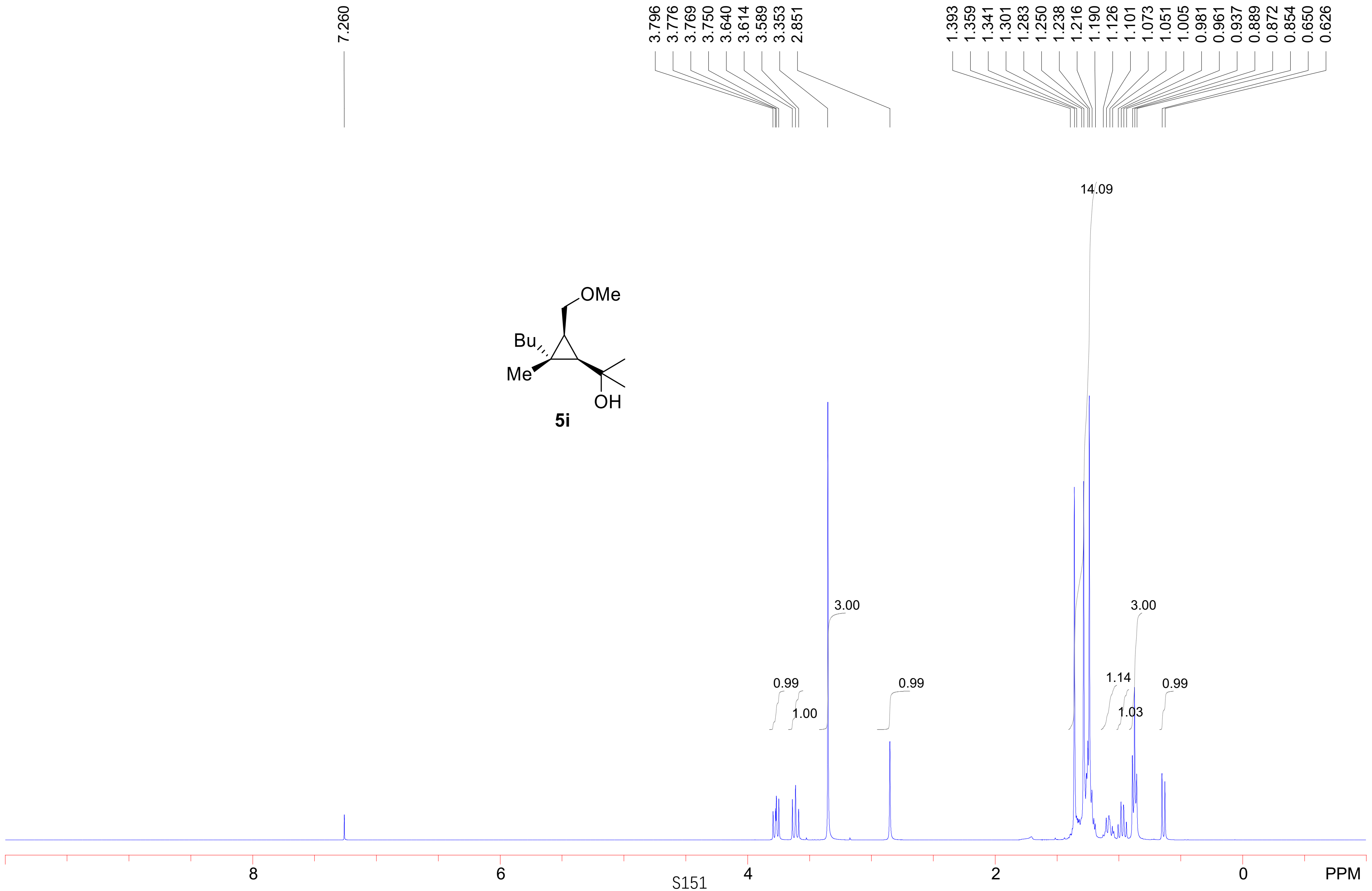
7.260
3.796
3.776
3.769
3.750
3.640
3.614
3.589
3.353
2.851
1.393
1.359
1.341
1.301
1.283
1.250
1.238
1.216
1.190
1.126
1.101
1.073
1.051
1.005
0.981
0.961
0.937
0.889
0.872
0.854
0.650
0.626
14.09
OMe
Bu
Me
OH
5i
3.00
0.99
1.00
0.99
1.14
1.03
3.00
0.99
8
6
4
2
0
PPM

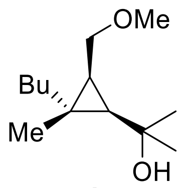
OMe
Bu
Me
OH

5i

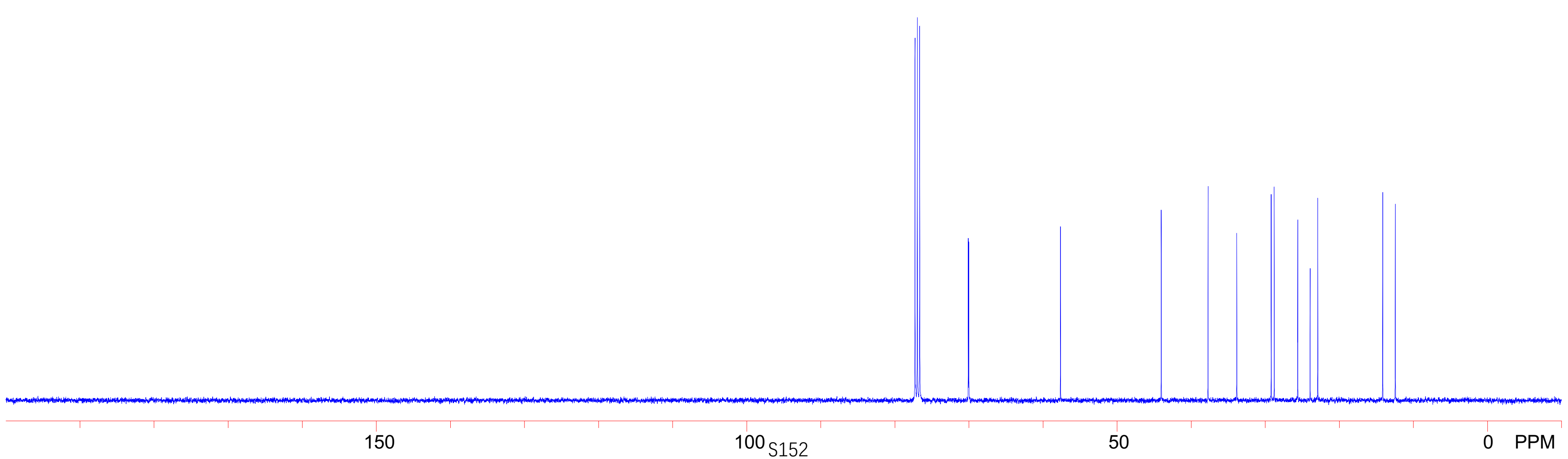
77.313
77.000
76.679
70.121
70.049
57.661
44.057
37.733
33.849
29.200
28.792
25.615
23.946
22.912
14.139
12.448
150
100
50
0
PPM

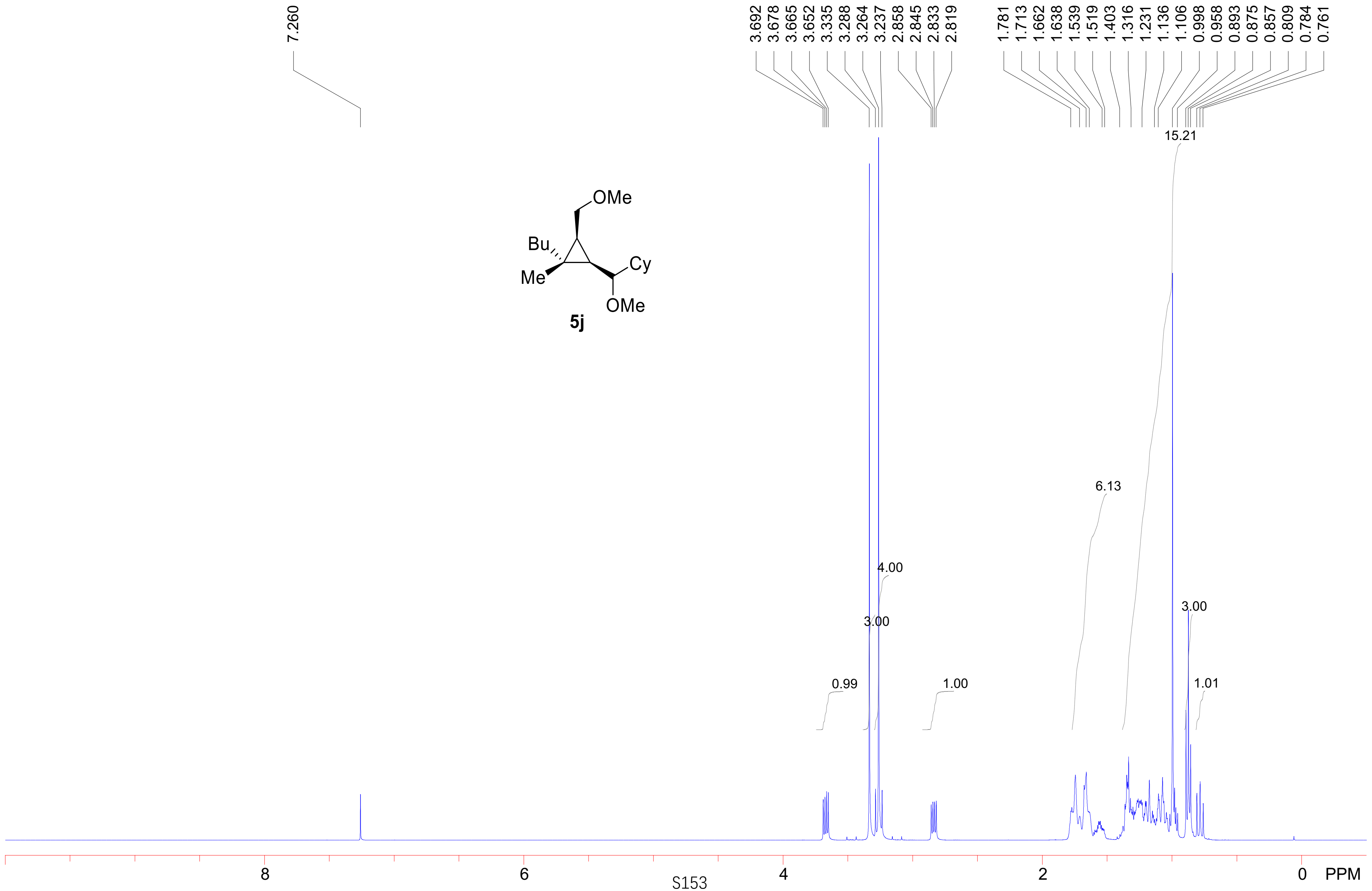
7.260
3.692 3.678 3.665 3.652 3.335 3.288 3.264 3.237 2.858 2.845 2.833 2.819
1.781 1.713 1.662 1.638 1.539 1.519 1.403 1.316 1.231 1.136 1.106 0.998 0.958 0.893 0.875 0.857 0.809 0.784 0.761
OMe
Bu
Me
Cy
OMe
5j
15.21
6.13
4.00
3.00
3.00
0.99
1.00
1.01
8
6
4
2
0
PPM

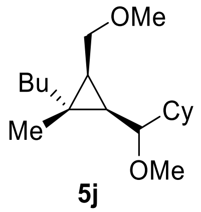

OMe
Bu
Cy
Me
OMe

5j

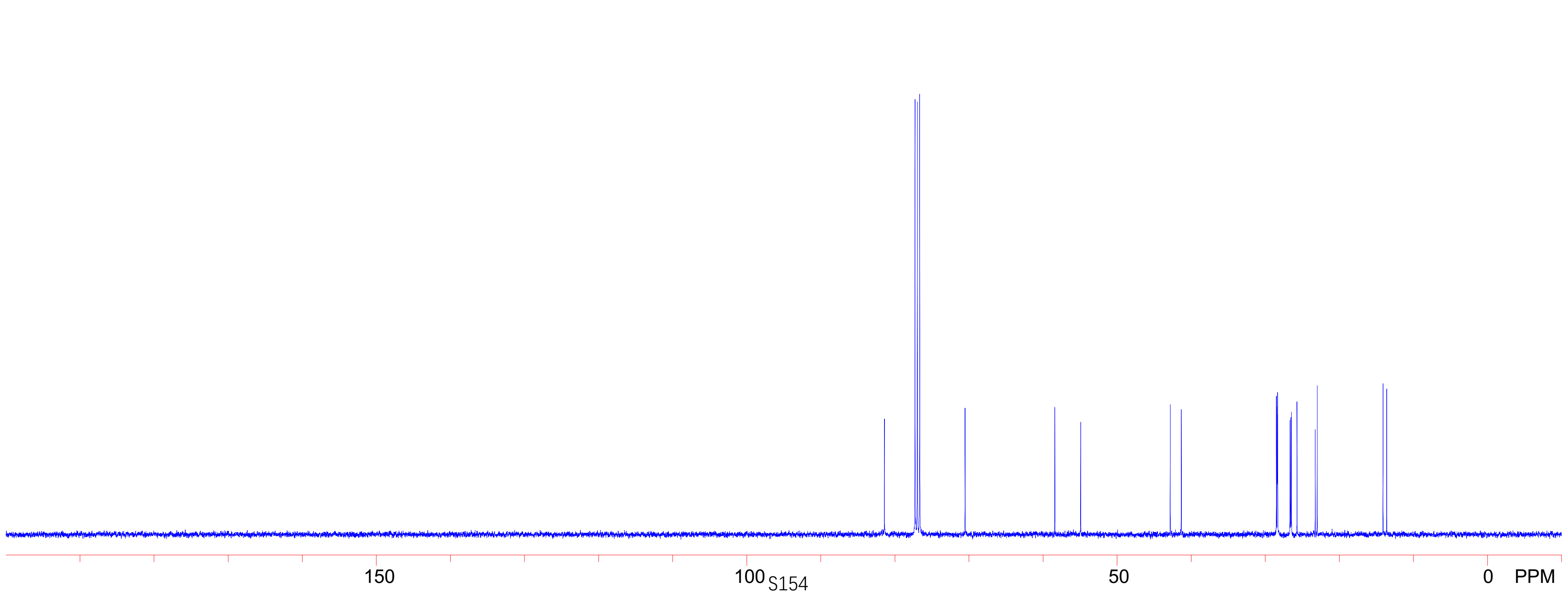

81.445
77.313
77.000
76.679
70.551
58.426
54.929
42.840
41.347
28.493
28.384
28.347
28.311
26.664
26.562
26.475
25.710
23.254
22.985
14.102
13.607
150
100
50
0
PPM

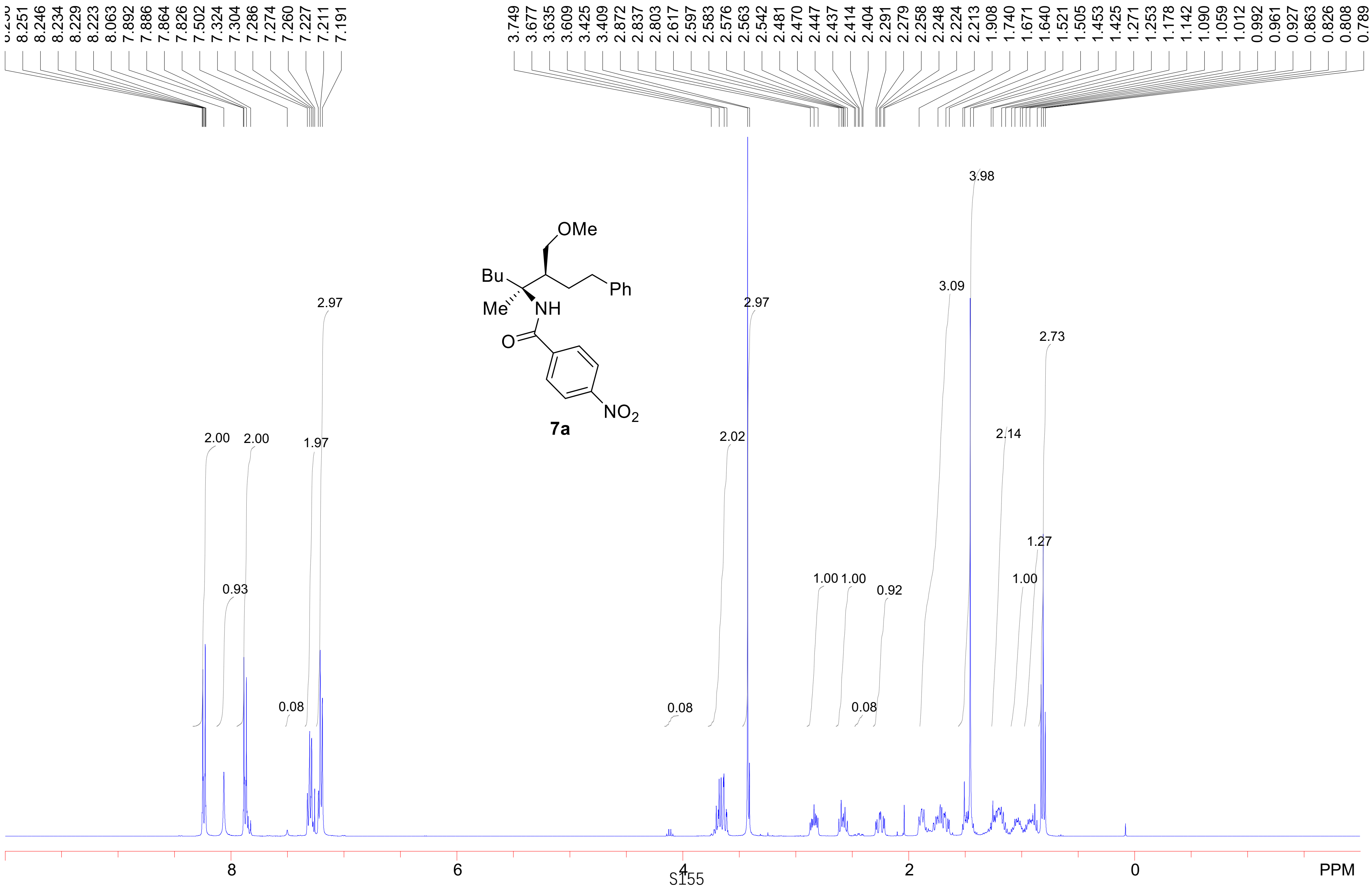
8.251 8.246 8.234 8.229 8.223 8.063 7.892 7.886 7.864 7.826 7.502 7.324 7.304 7.286 7.274 7.260 7.227 7.211 7.191
3.749 3.677 3.635 3.609 3.425 3.409 2.872 2.837 2.803 2.617 2.597 2.583 2.576 2.563 2.542 2.481 2.470 2.447 2.437 2.414 2.404 2.291 2.279 2.258 2.248 2.224 2.213 1.908 1.740 1.671 1.640 1.521 1.505 1.453 1.425 1.271 1.253 1.178 1.142 1.090 1.059 1.012 0.992 0.961 0.927 0.863 0.826 0.808 0.789
OMe
Bu
Me
NH
Ph
O
NO_2
7a
2.00 0.93 2.00 0.08 1.97 2.97
0.08 2.02 2.97 1.00 1.00 0.08 0.92 3.09 3.98 2.14 1.00 1.27 2.73
8
6
4
2
0
PPM

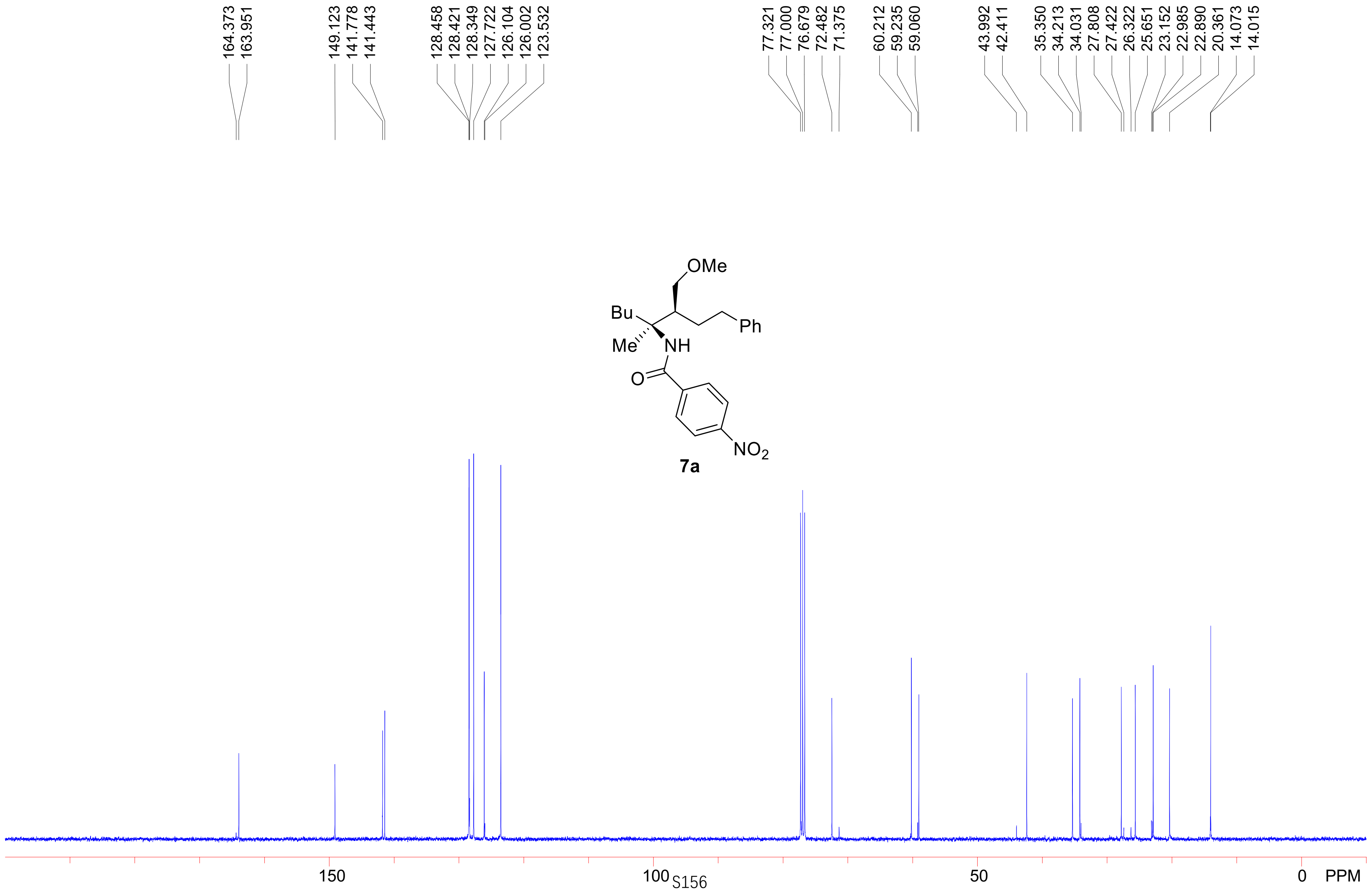
164.373
163.951
149.123
141.778
141.443
128.458
128.421
128.349
127.722
126.104
126.002
123.532
77.321
77.000
76.679
72.482
71.375
60.212
59.235
59.060
43.992
42.411
35.350
34.213
34.031
27.808
27.422
26.322
25.651
23.152
22.985
22.890
20.361
14.073
14.015
OMe
Bu
Ph
Me
NH
O
NO_2
7a
150
100
50
0
PPM

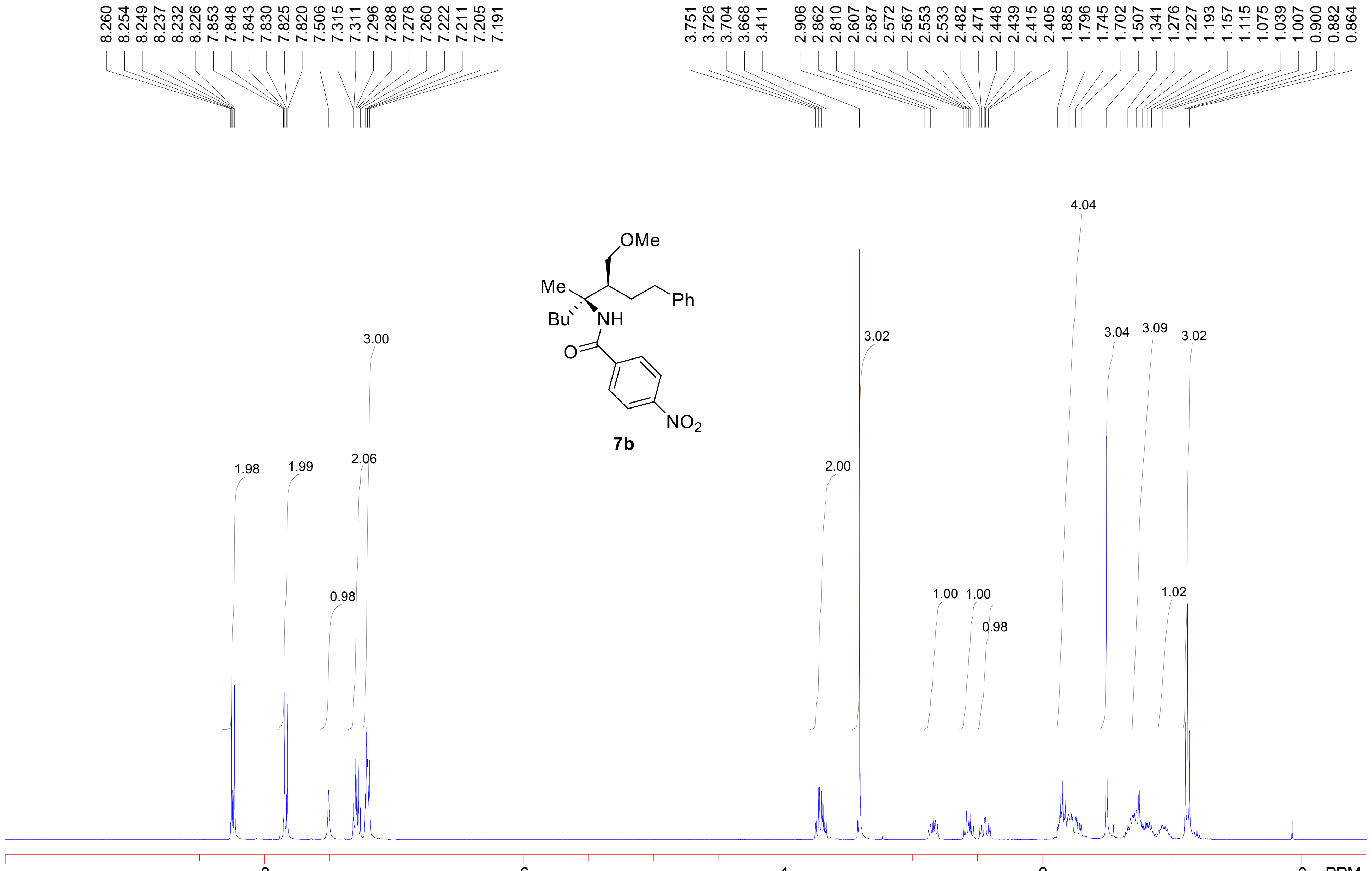

8.260
8.254
8.249
8.237
8.232
8.226
7.853
7.848
7.843
7.830
7.825
7.820
7.506
7.315
7.311
7.296
7.288
7.278
7.260
7.222
7.211
7.205
7.191
3.751
3.726
3.704
3.668
3.411
2.906
2.862
2.810
2.607
2.587
2.572
2.567
2.553
2.533
2.482
2.471
2.448
2.439
2.415
2.405
1.885
1.796
1.745
1.702
1.507
1.341
1.276
1.227
1.193
1.157
1.115
1.075
1.039
1.007
0.900
0.882
0.864
OMe
Me
Ph
Bu
NH
O
NO_2
7b
4.04
3.00
3.02
3.04
3.09
3.02
1.98
1.99
2.06
2.00
0.98
1.00
1.00
0.98
1.02
8
6
4
2
0
PPM

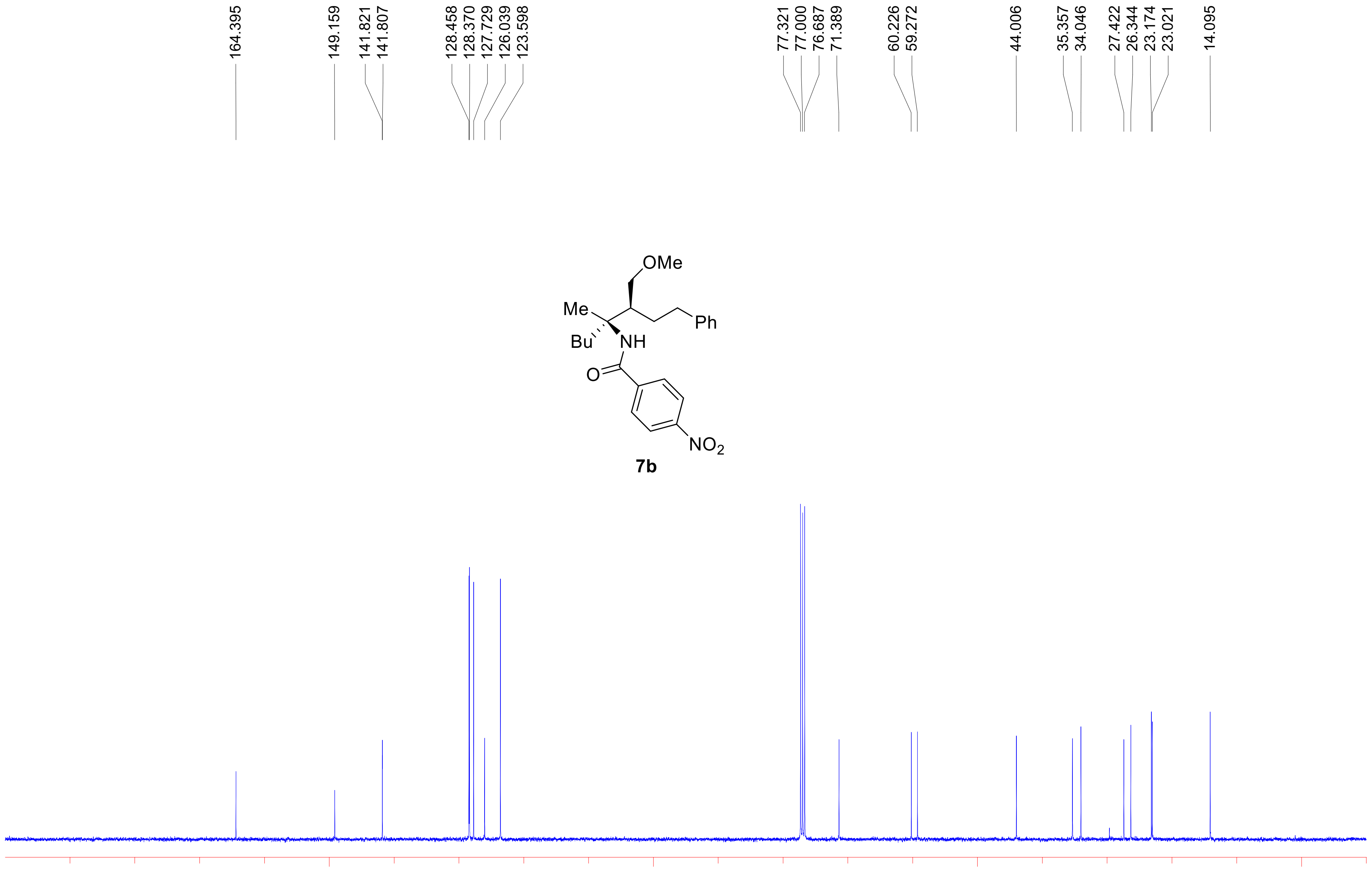
164.395
149.159
141.821
141.807
128.458
128.370
127.729
126.039
123.598
77.321
77.000
76.687
71.389
60.226
59.272
44.006
35.357
34.046
27.422
26.344
23.174
23.021
14.095
150
100
50
0
PPM
OMe
Me
Ph
Bu
NH
O
NO2

7b

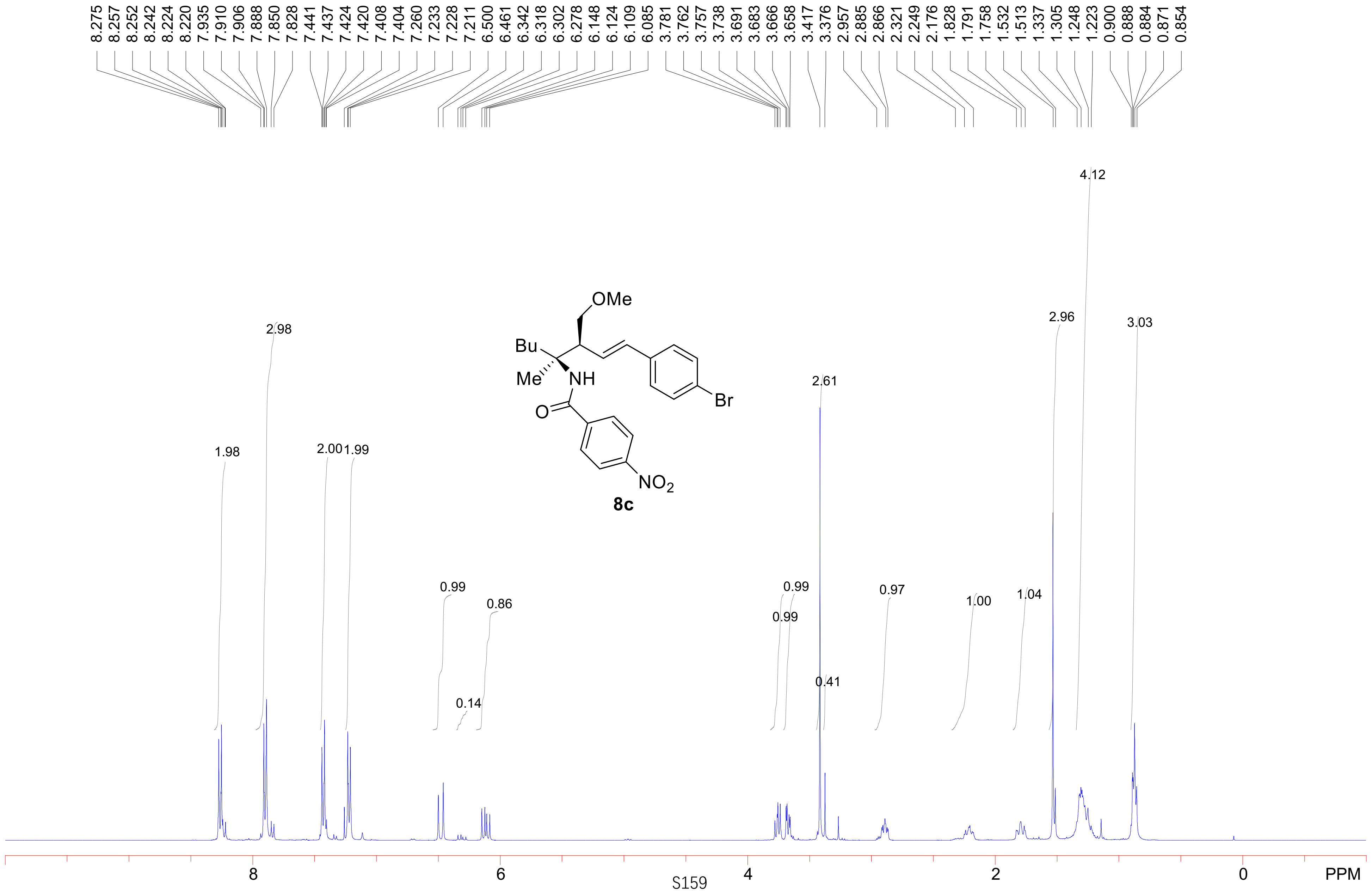
8.275 8.257 8.252 8.242 8.224 8.220 7.935 7.910 7.906 7.888 7.850 7.828 7.441 7.437 7.424 7.420 7.408 7.404 7.260 7.233 7.228 7.211 6.500 6.461 6.342 6.318 6.302 6.278 6.148 6.124 6.109 6.085 3.781 3.762 3.757 3.738 3.691 3.683 3.666 3.658 3.417 3.376 2.957 2.885 2.866 2.321 2.249 2.176 1.828 1.791 1.758 1.532 1.513 1.337 1.305 1.248 1.223 0.900 0.888 0.884 0.871 0.854
1.98
2.98
2.00
1.99
0.99
0.14
0.86
0.99
0.99
2.61
0.41
0.97
1.00
1.04
2.96
4.12
3.03
OMe
Bu
Me
NH
Br
O
NO2
8c
8
6
4
2
0
PPM

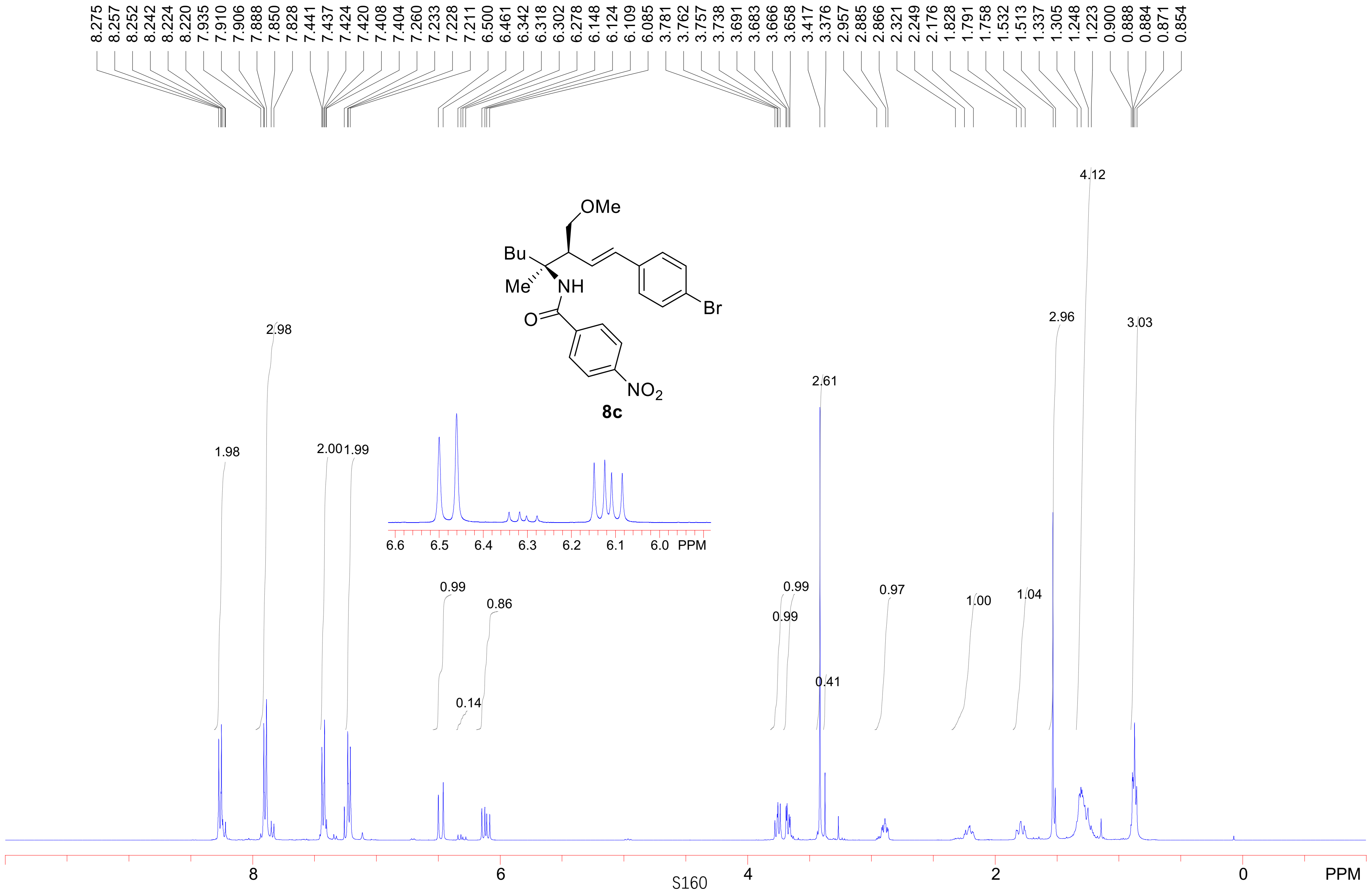
8.275
8.257
8.252
8.242
8.224
8.220
7.935
7.910
7.906
7.888
7.850
7.828
7.441
7.437
7.424
7.420
7.408
7.404
7.260
7.233
7.228
7.211
6.500
6.461
6.342
6.318
6.302
6.278
6.148
6.124
6.109
6.085
3.781
3.762
3.757
3.738
3.691
3.683
3.666
3.658
3.417
3.376
2.957
2.885
2.866
2.321
2.249
2.176
1.828
1.791
1.758
1.532
1.513
1.337
1.305
1.248
1.223
0.900
0.888
0.884
0.871
0.854
OMe
Bu
Me
NH
O
Br
NO2
8c
1.98
2.98
2.00
1.99
0.99
0.86
0.14
6.6
6.5
6.4
6.3
6.2
6.1
6.0
PPM
2.61
0.41
0.99
0.99
0.97
1.00
1.04
2.96
4.12
3.03
8
6
4
2
0
PPM

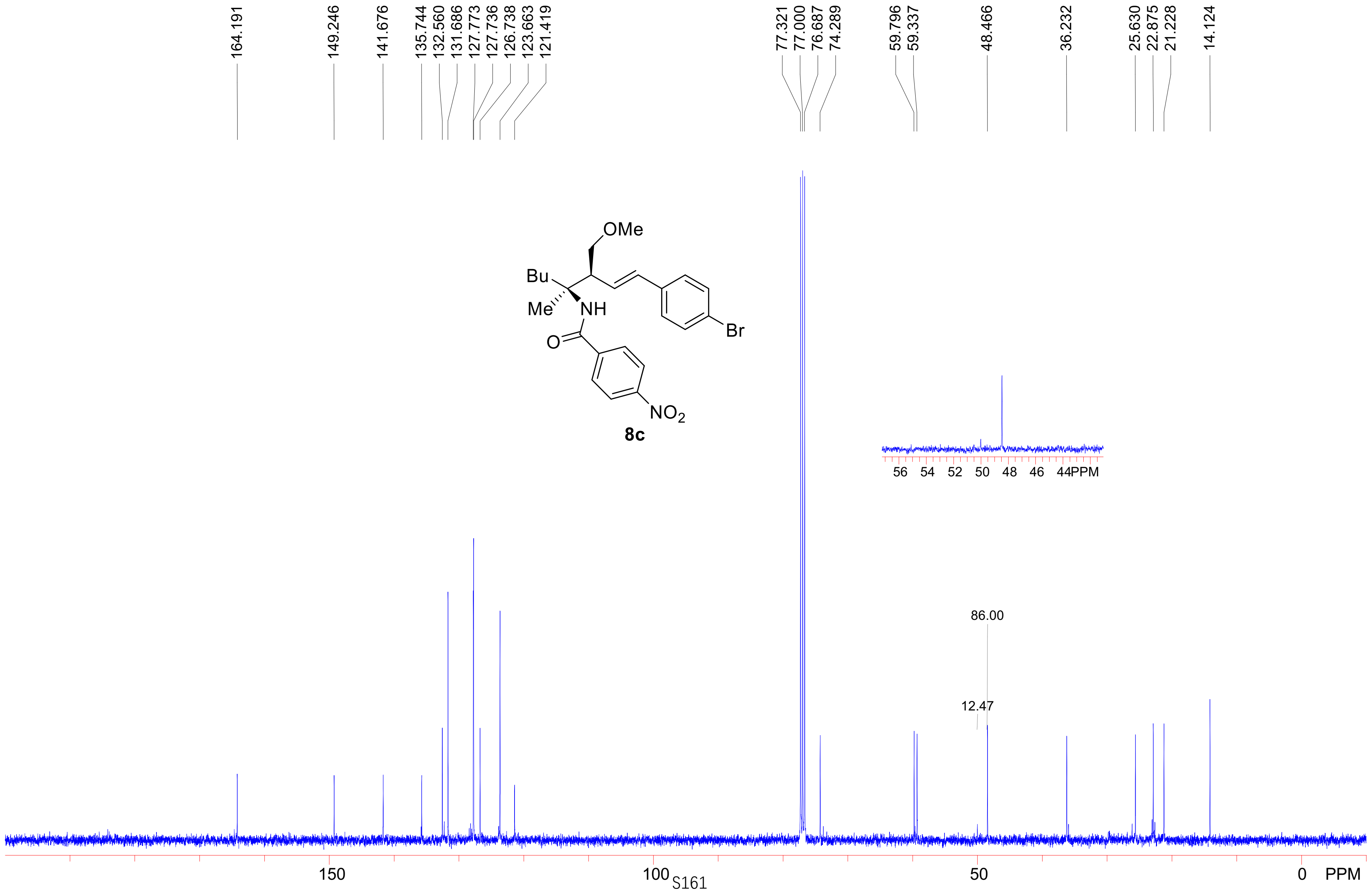
164.191
149.246
141.676
135.744
132.560
131.686
127.773
127.736
126.738
123.663
121.419
77.321
77.000
76.687
74.289
59.796
59.337
48.466
36.232
25.630
22.875
21.228
14.124
OMe
Bu
Me
NH
O
Br
NO2
8c
56 54 52 50 48 46 44PPM
86.00
12.47
150
100
50
0
PPM

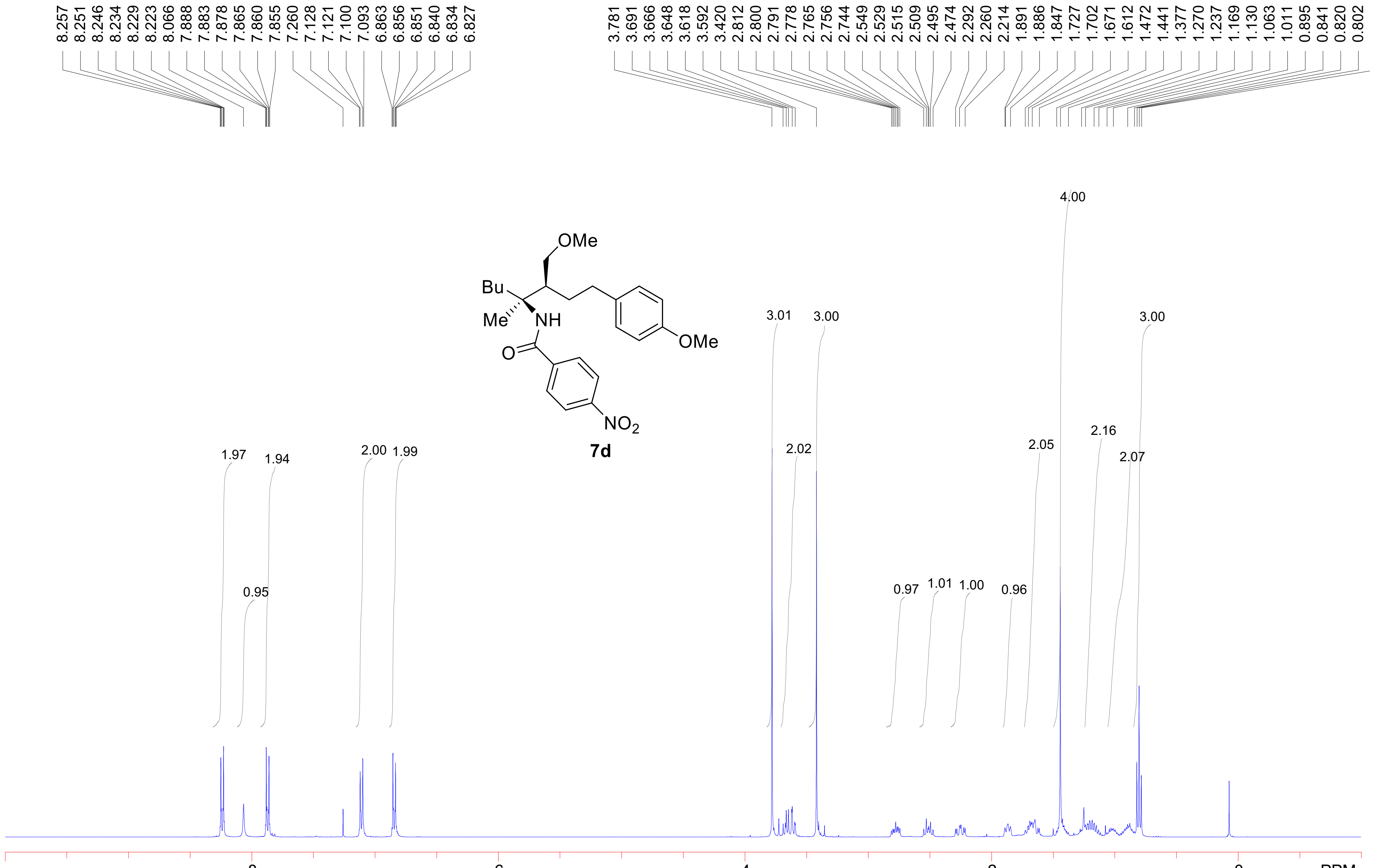
8.257
8.251
8.246
8.234
8.229
8.223
8.066
7.888
7.883
7.878
7.865
7.860
7.855
7.260
7.128
7.121
7.100
7.093
6.863
6.856
6.851
6.840
6.834
6.827
3.781
3.691
3.666
3.648
3.618
3.592
3.420
2.812
2.800
2.791
2.778
2.765
2.756
2.744
2.549
2.529
2.515
2.509
2.495
2.474
2.292
2.260
2.214
1.891
1.886
1.847
1.727
1.702
1.671
1.612
1.472
1.441
1.377
1.270
1.237
1.169
1.130
1.063
1.011
0.895
0.841
0.820
0.802
OMe
Bu
Me
NH
O
OMe
NO_2
7d
1.97
0.95
1.94
2.00
1.99
3.01
2.02
3.00
0.97
1.01
1.00
0.96
2.05
4.00
2.16
2.07
3.00
8
6
4
2
0
PPM

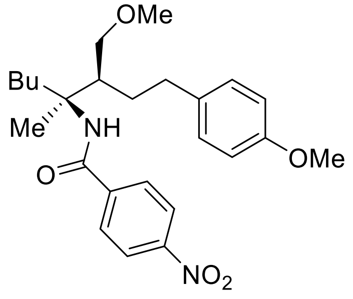
OMe
Bu
Me
NH
O
OMe
NO2

7d

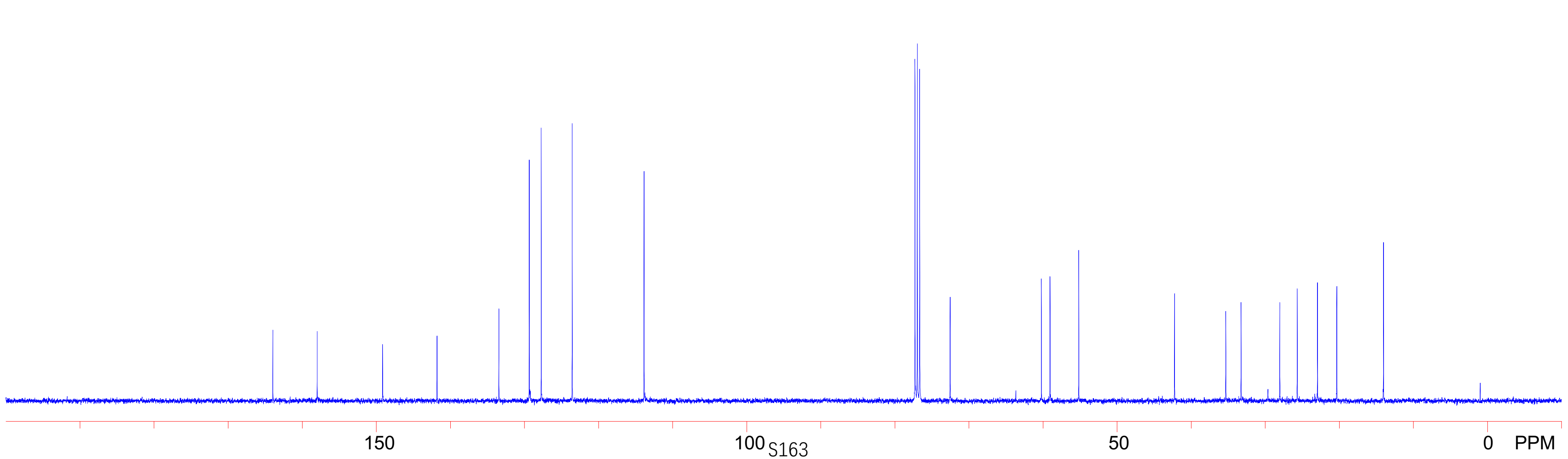
163.973
157.983
149.152
141.807
133.456
129.354
127.744
123.561
113.870
77.321
77.000
76.687
72.570
60.241
59.075
55.191
42.258
35.335
33.266
28.041
25.681
22.948
20.340
14.037
150
100
50
0
PPM

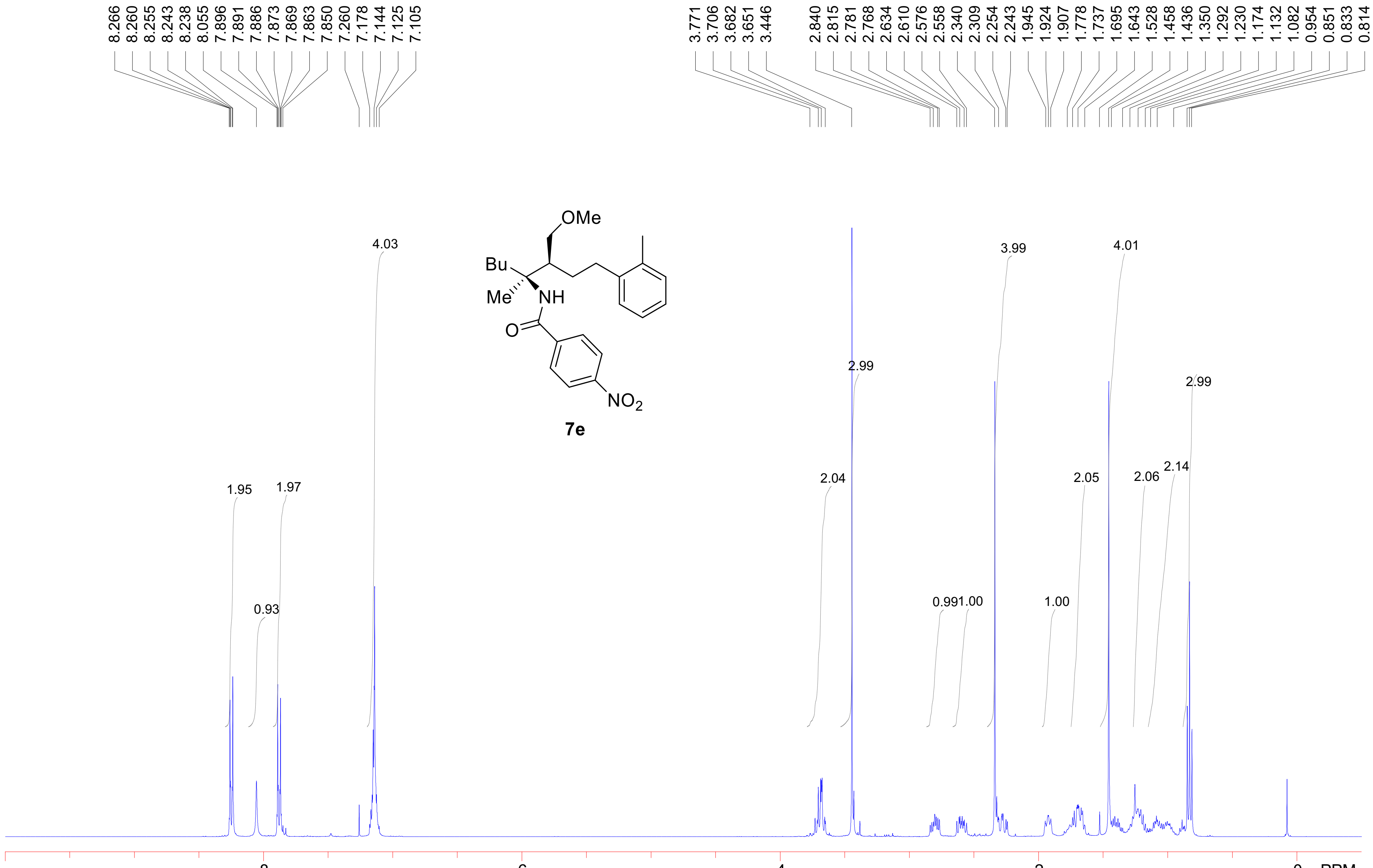
8.266
8.260
8.255
8.243
8.238
8.055
7.896
7.891
7.886
7.873
7.869
7.863
7.850
7.260
7.178
7.144
7.125
7.105
3.771
3.706
3.682
3.651
3.446
2.840
2.815
2.781
2.768
2.634
2.610
2.576
2.558
2.340
2.309
2.254
2.243
1.945
1.924
1.907
1.778
1.737
1.695
1.643
1.528
1.458
1.436
1.350
1.292
1.230
1.174
1.132
1.082
0.954
0.851
0.833
0.814
OMe
Bu
Me
NH
O
NO2
7e
1.95
0.93
1.97
4.03
2.04
2.99
0.99
1.00
3.99
1.00
2.05
4.01
2.06
2.14
2.99
8
6
4
2
0
PPM

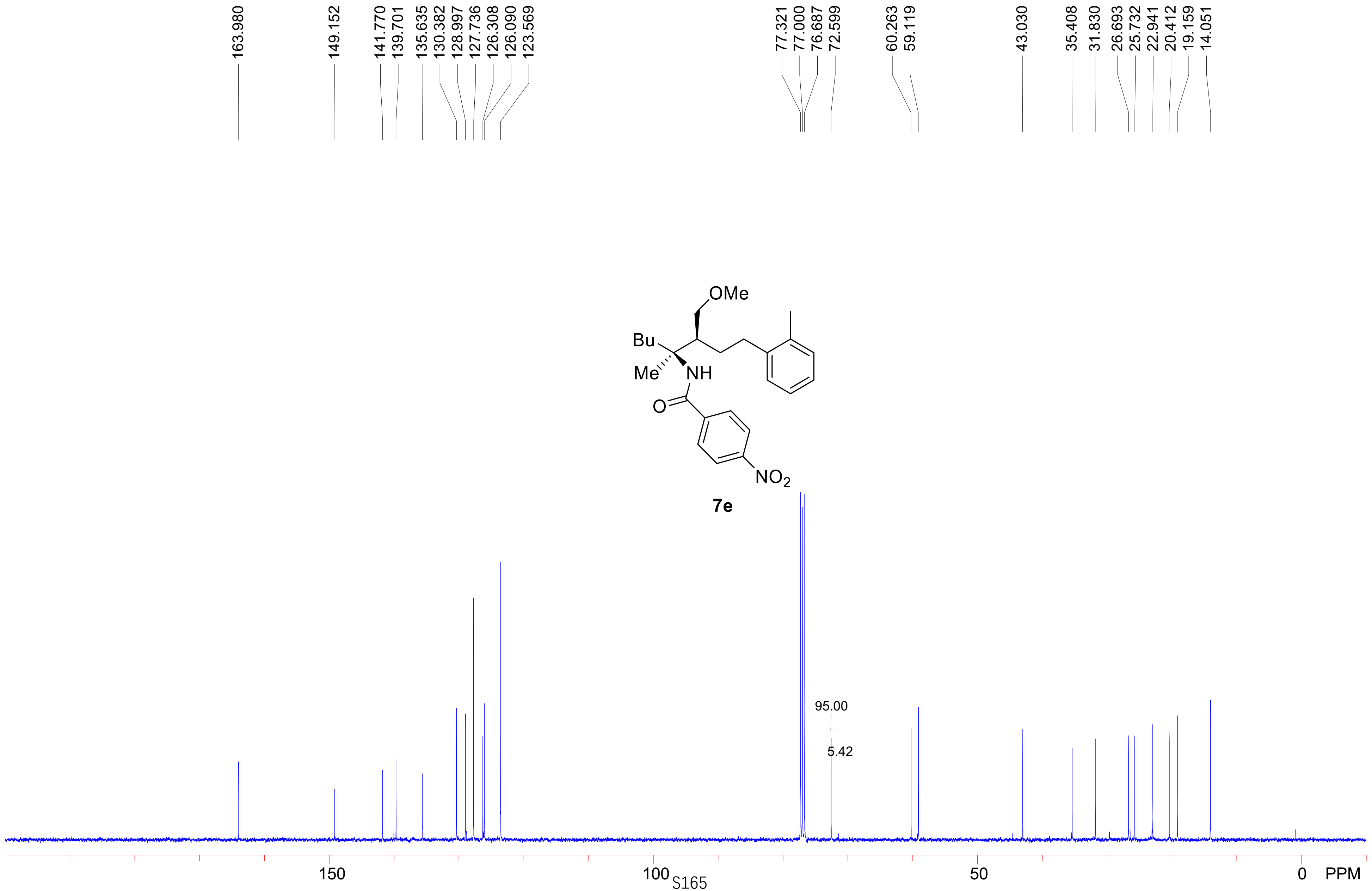
163.980
149.152
141.770
139.701
135.635
130.382
128.997
127.736
126.308
126.090
123.569
77.321
77.000
76.687
72.599
60.263
59.119
43.030
35.408
31.830
26.693
25.732
22.941
20.412
19.159
14.051
OMe
Bu
Me
NH
O
NO_2
7e
95.00
5.42
150
100
50
0
PPM

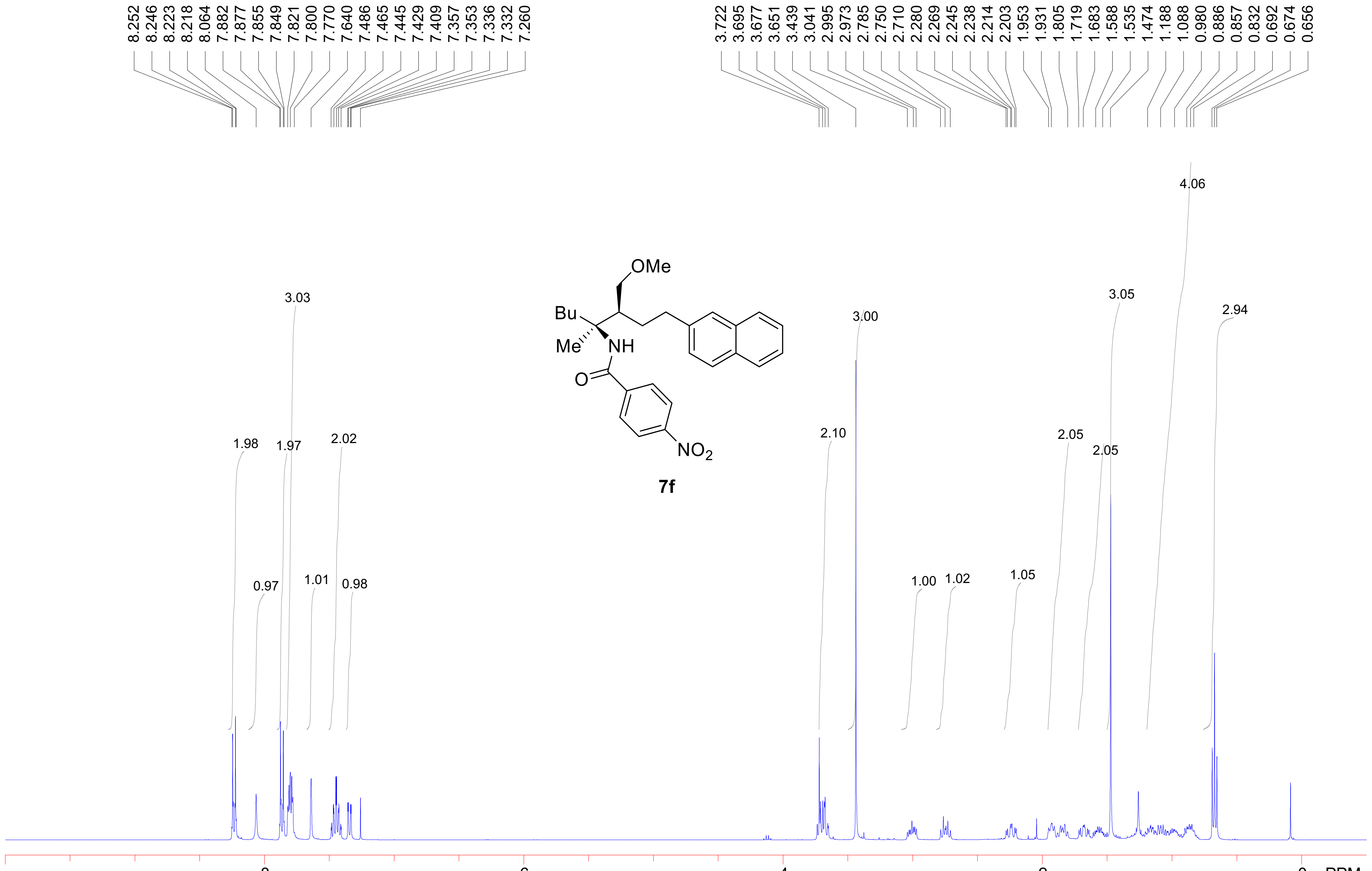
8.252
8.246
8.223
8.218
8.064
7.882
7.877
7.855
7.849
7.821
7.800
7.770
7.640
7.486
7.465
7.445
7.429
7.409
7.357
7.353
7.336
7.332
7.260
3.722
3.695
3.677
3.651
3.439
3.041
2.995
2.973
2.785
2.750
2.710
2.280
2.269
2.245
2.238
2.214
2.203
1.953
1.931
1.805
1.719
1.683
1.588
1.535
1.474
1.188
1.088
0.980
0.886
0.857
0.832
0.692
0.674
0.656
OMe
Bu
Me
NH
O
NO2
7f
1.98
0.97
1.97
3.03
1.01
2.02
0.98
2.10
3.00
1.00
1.02
1.05
2.05
2.05
3.05
4.06
2.94
8
6
4
2
0
PPM

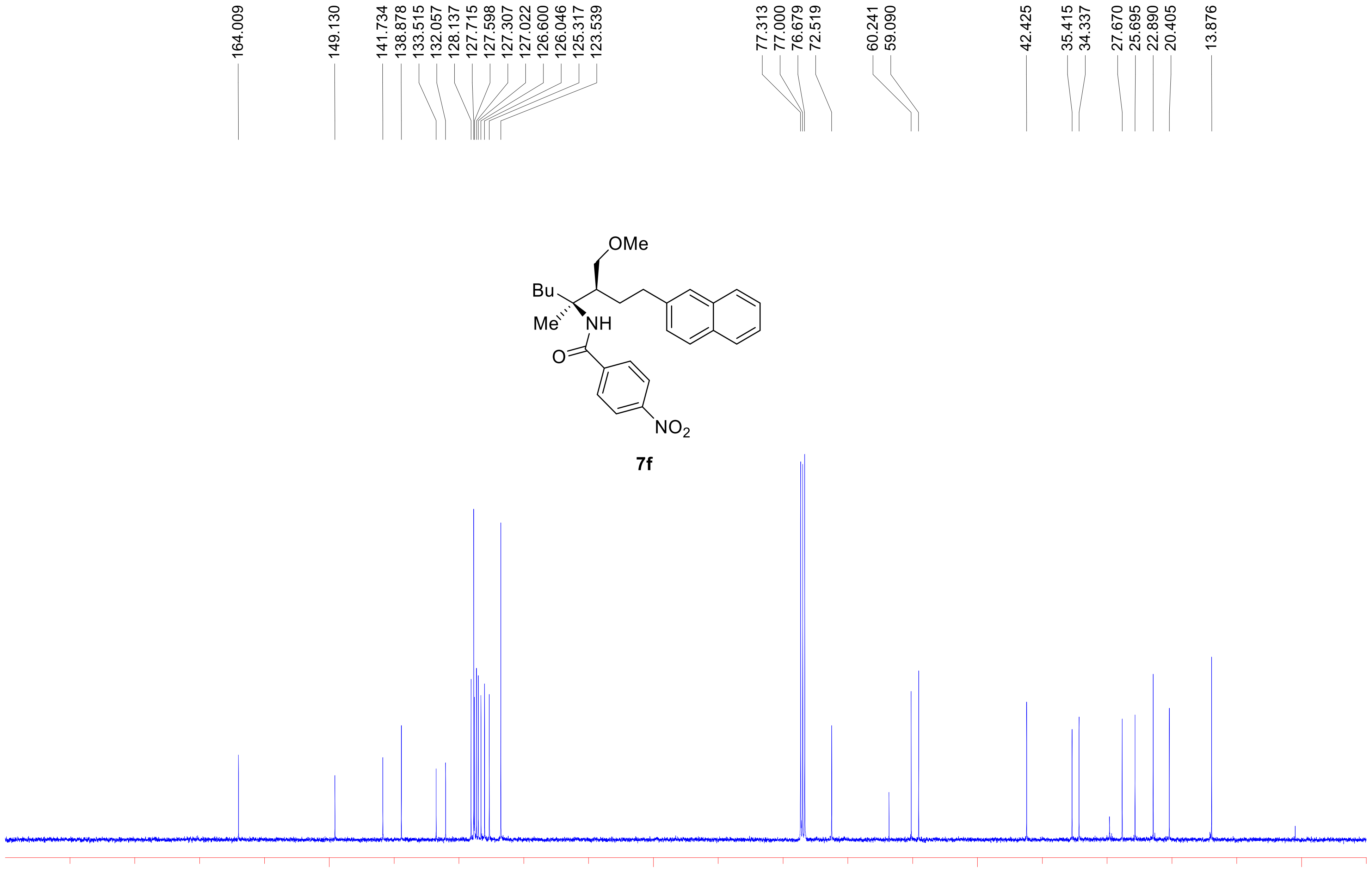
164.009
149.130
141.734
138.878
133.515
132.057
128.137
127.715
127.598
127.307
127.022
126.600
126.046
125.317
123.539
77.313
77.000
76.679
72.519
60.241
59.090
42.425
35.415
34.337
27.670
25.695
22.890
20.405
13.876
150
100
50
0
PPM
OMe
Bu
Me
NH
O
NO2

7f

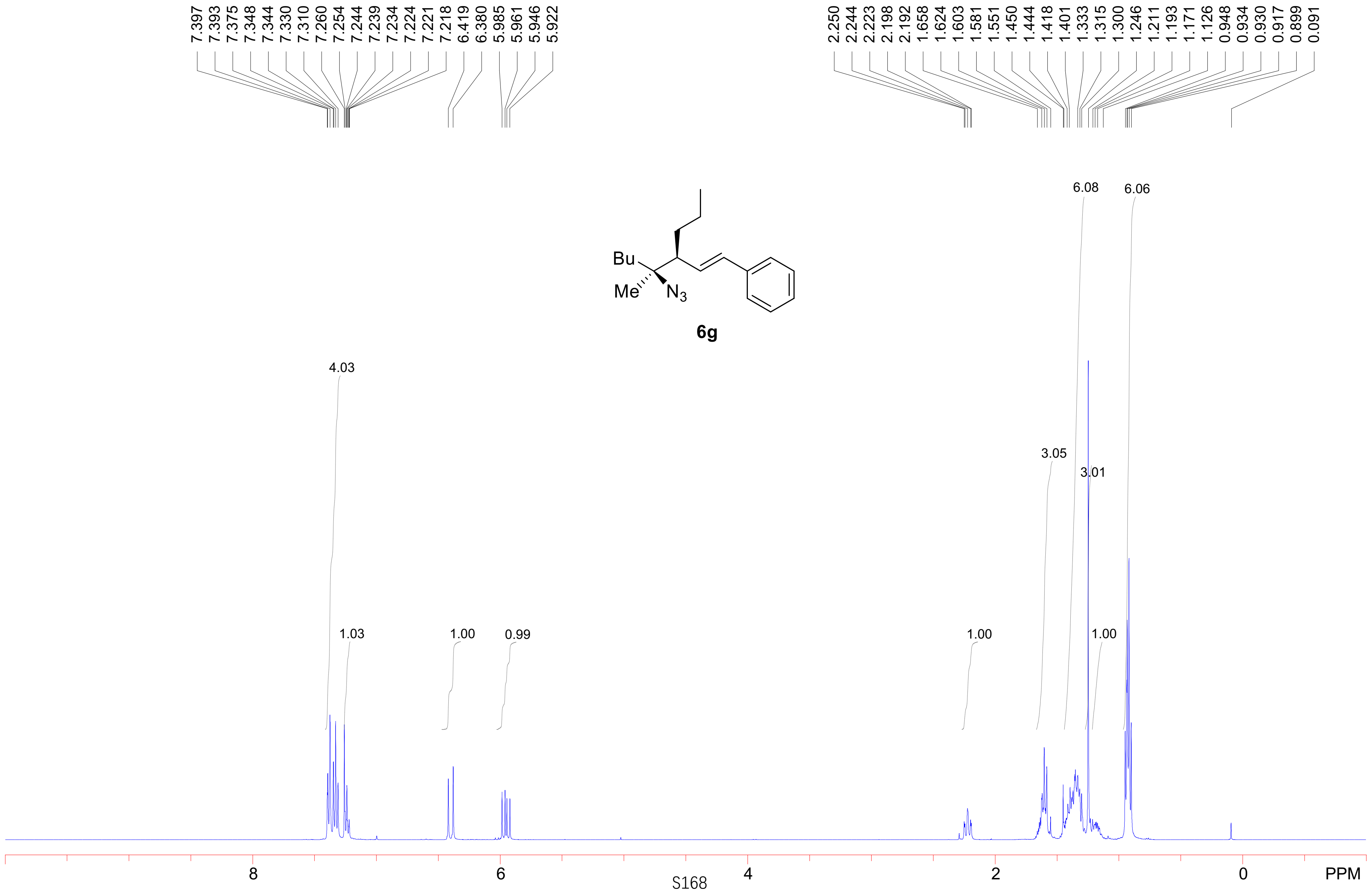
7.397
7.393
7.375
7.348
7.344
7.330
7.310
7.260
7.254
7.244
7.239
7.234
7.224
7.221
7.218
6.419
6.380
5.985
5.961
5.946
5.922
2.250
2.244
2.223
2.198
2.192
1.658
1.624
1.603
1.581
1.551
1.450
1.444
1.418
1.401
1.333
1.315
1.300
1.246
1.211
1.193
1.171
1.126
0.948
0.934
0.930
0.917
0.899
0.091
Bu
Me
N3
6g
4.03
1.03
1.00
0.99
1.00
3.05
6.08
3.01
1.00
6.06
8
6
4
2
0
PPM

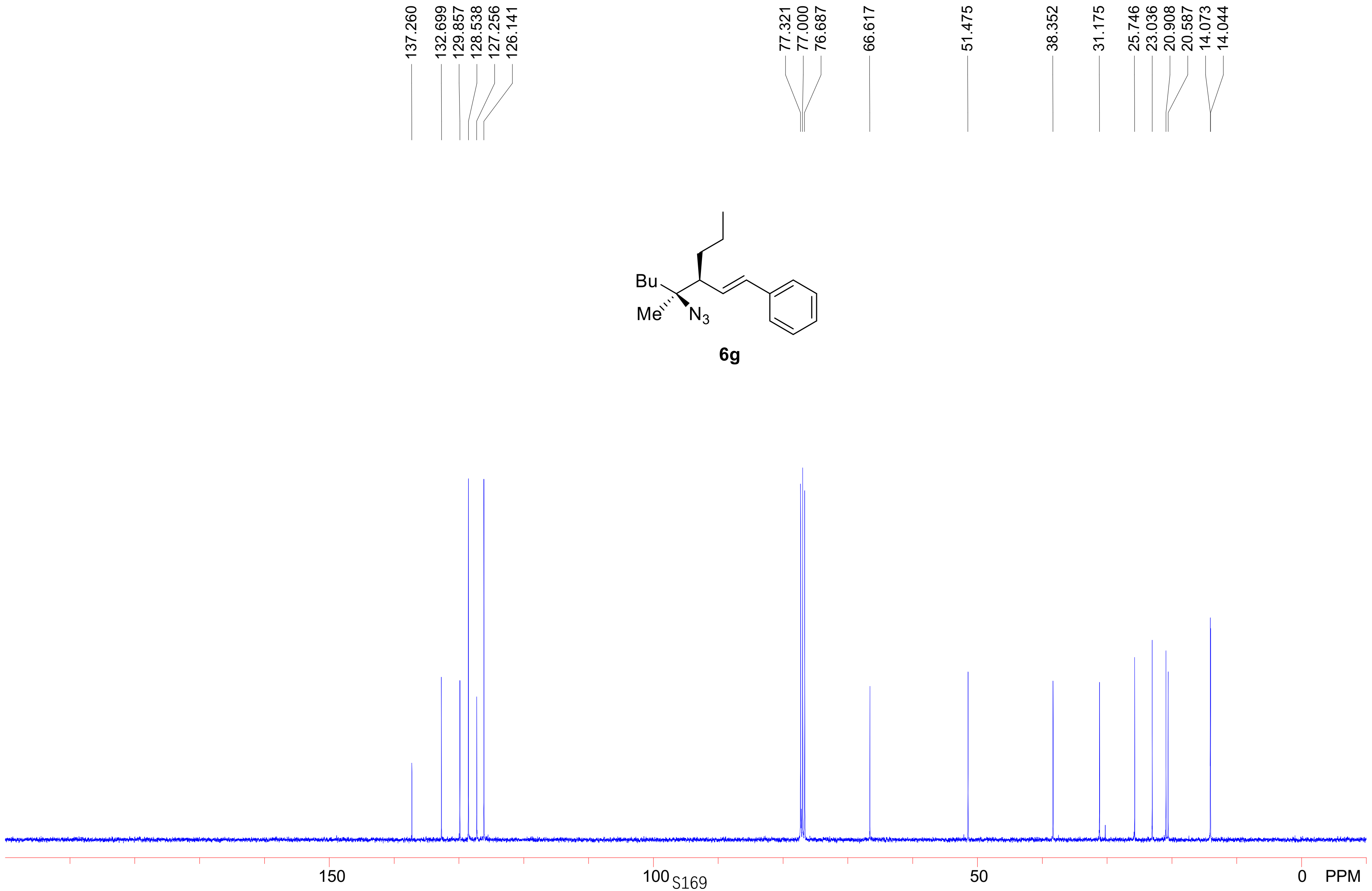
137.260
132.699
129.857
128.538
127.256
126.141
77.321
77.000
76.687
66.617
51.475
38.352
31.175
25.746
23.036
20.908
20.587
14.073
14.044
Bu
Me
N_3
6g
150
100
50
0
PPM

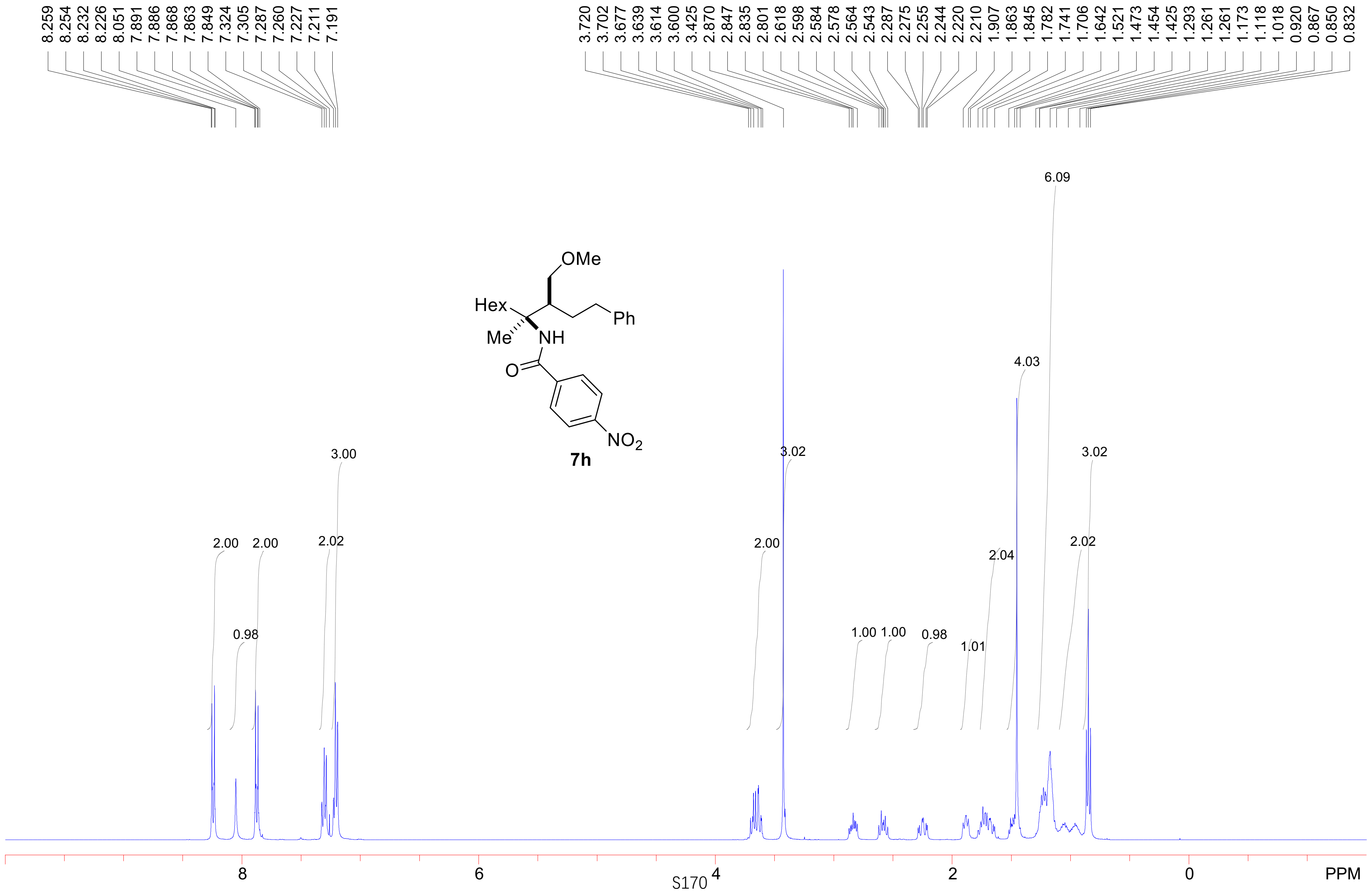
8.259
8.254
8.232
8.226
8.051
7.891
7.886
7.868
7.863
7.849
7.324
7.305
7.287
7.260
7.227
7.211
7.191
3.720
3.702
3.677
3.639
3.614
3.600
3.425
2.870
2.847
2.835
2.801
2.618
2.598
2.584
2.578
2.564
2.543
2.287
2.275
2.255
2.244
2.220
2.210
1.907
1.863
1.845
1.782
1.741
1.706
1.642
1.521
1.473
1.454
1.425
1.293
1.261
1.173
1.118
1.018
0.920
0.867
0.850
0.832
OMe
Hex
Ph
Me
NH
O
NO2
7h
6.09
4.03
3.00
3.02
3.02
2.00
2.00
2.02
2.00
2.04
2.02
0.98
1.00
1.00
0.98
1.01
8
6
4
2
0
PPM

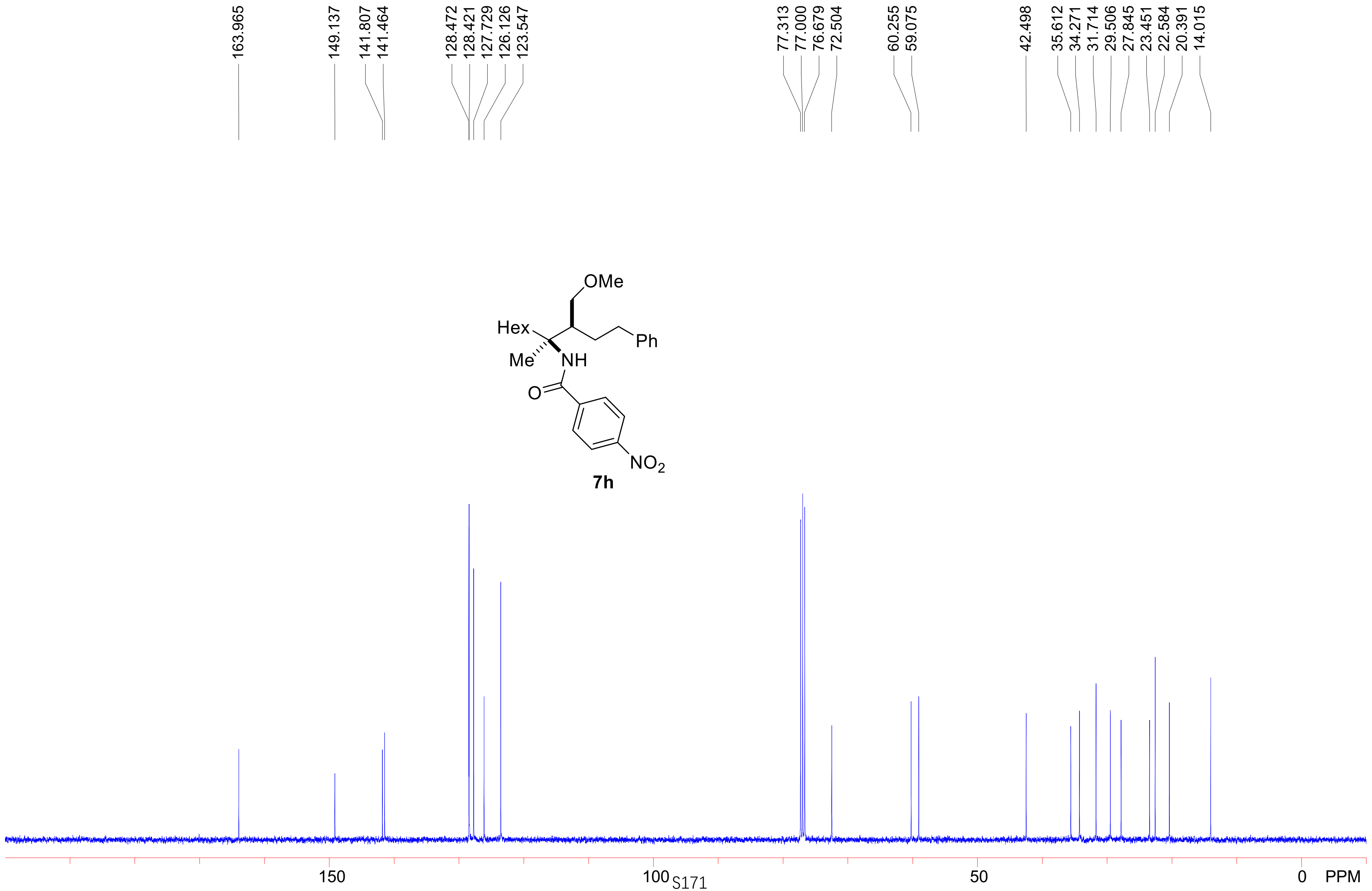
163.965
149.137
141.807
141.464
128.472
128.421
127.729
126.126
123.547
77.313
77.000
76.679
72.504
60.255
59.075
42.498
35.612
34.271
31.714
29.506
27.845
23.451
22.584
20.391
14.015
OMe
Hex
Ph
Me
NH
O
NO2
7h
150
100
50
0
PPM

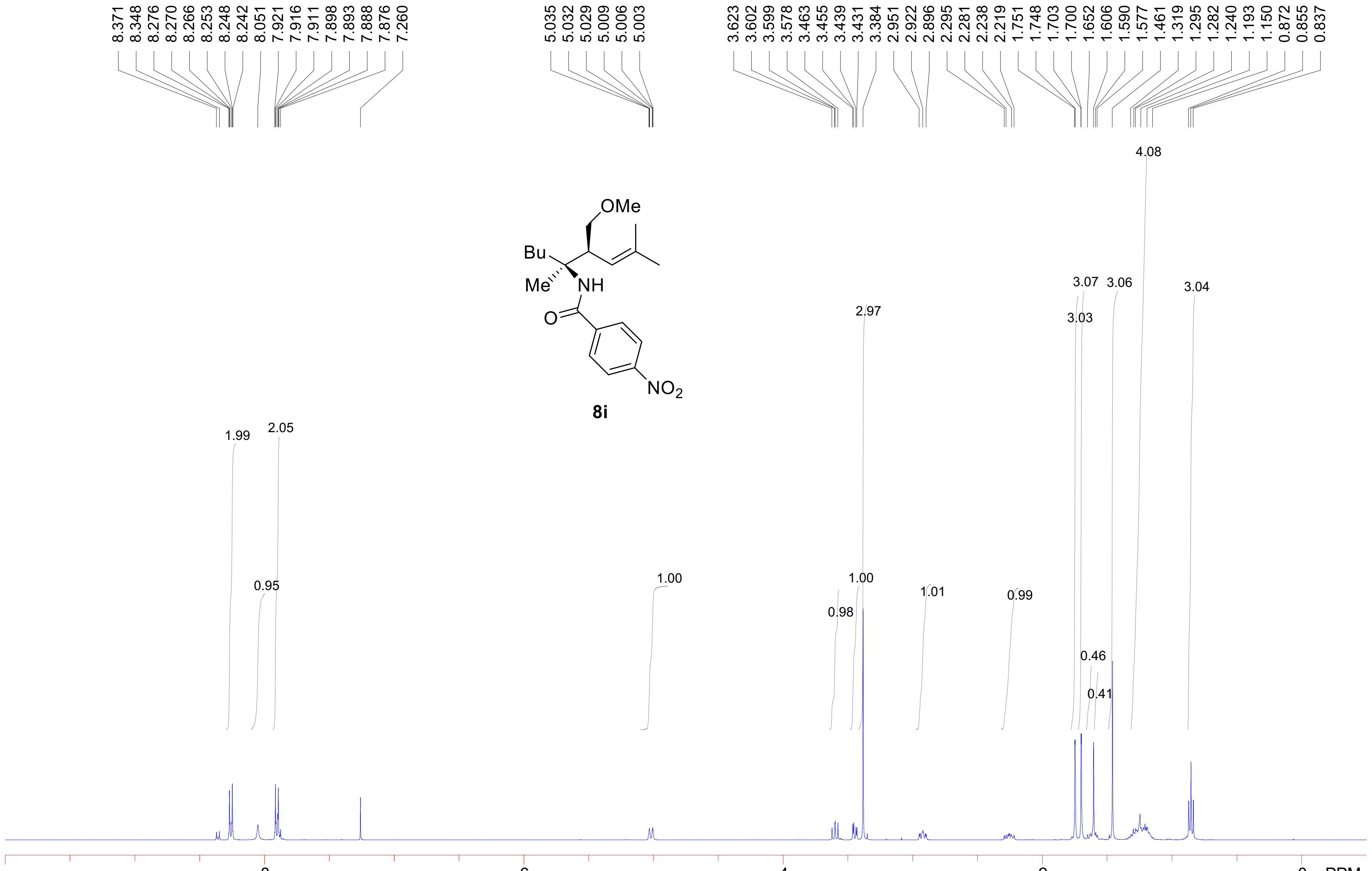
8.371 8.348 8.276 8.270 8.266 8.253 8.248 8.242 8.051 7.921 7.916 7.911 7.898 7.893 7.888 7.876 7.260
5.035 5.032 5.029 5.009 5.006 5.003
3.623 3.602 3.599 3.578 3.463 3.455 3.439 3.431 3.384 2.951 2.922 2.896 2.295 2.281 2.238 2.219 1.751 1.748 1.703 1.700 1.652 1.606 1.590 1.577 1.461 1.319 1.295 1.282 1.240 1.193 1.150 0.872 0.855 0.837
OMe
Bu
Me
NH
O
NO_2
8i
1.99
2.05
0.95
1.00
0.98
1.00
2.97
1.01
0.99
3.07
3.03
3.06
4.08
3.04
0.46
0.41
8
6
4
2
0
PPM

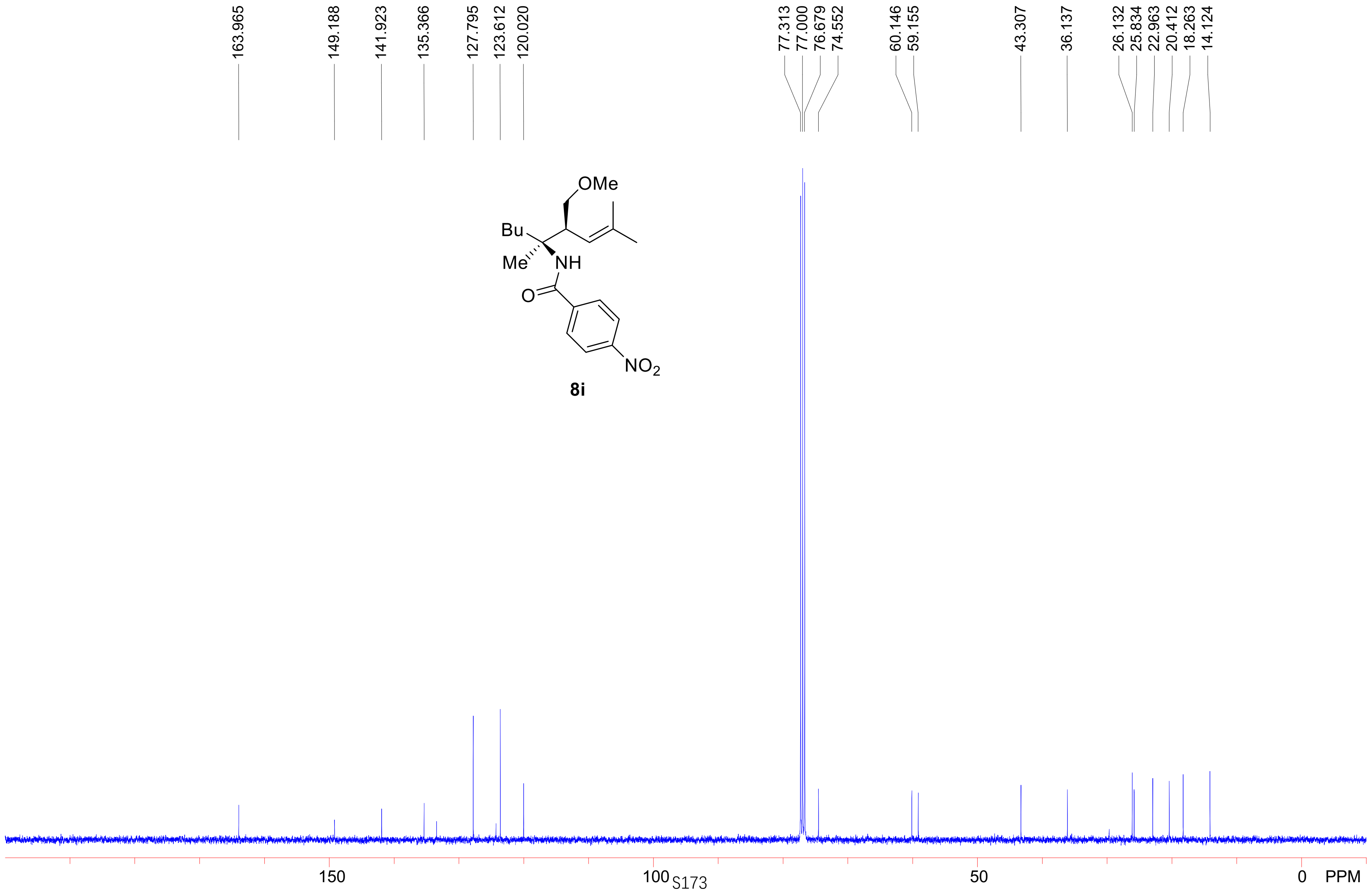
163.965
149.188
141.923
135.366
127.795
123.612
120.020
77.313
77.000
76.679
74.552
60.146
59.155
43.307
36.137
26.132
25.834
22.963
20.412
18.263
14.124
OMe
Bu
Me
NH
O
NO2
8i
150
100
50
0
PPM

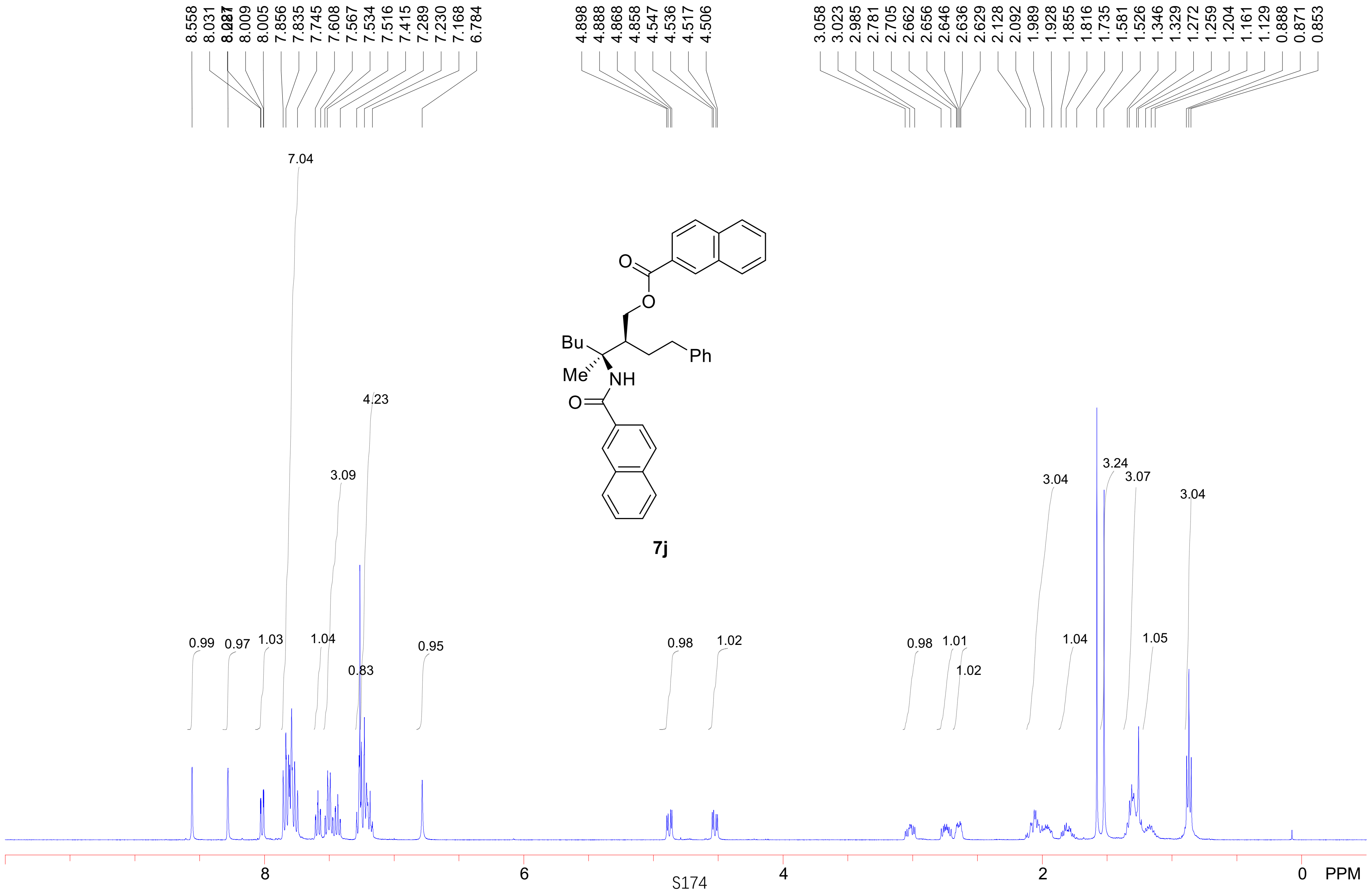
8.558
8.031
8.009
8.005
7.856
7.835
7.745
7.608
7.567
7.534
7.516
7.415
7.289
7.230
7.168
6.784
4.898
4.888
4.868
4.858
4.547
4.536
4.517
4.506
3.058
3.023
2.985
2.781
2.705
2.662
2.656
2.646
2.636
2.629
2.128
2.092
1.989
1.928
1.855
1.816
1.735
1.581
1.526
1.346
1.329
1.272
1.259
1.204
1.161
1.129
0.888
0.871
0.853
7.04
4.23
3.09
0.99
0.97
1.03
1.04
0.83
0.95
0.98
1.02
0.98
1.01
1.02
3.04
1.04
3.24
3.07
1.05
3.04
Bu
Me
NH
O
Ph
8
6
4
2
0
PPM

7j

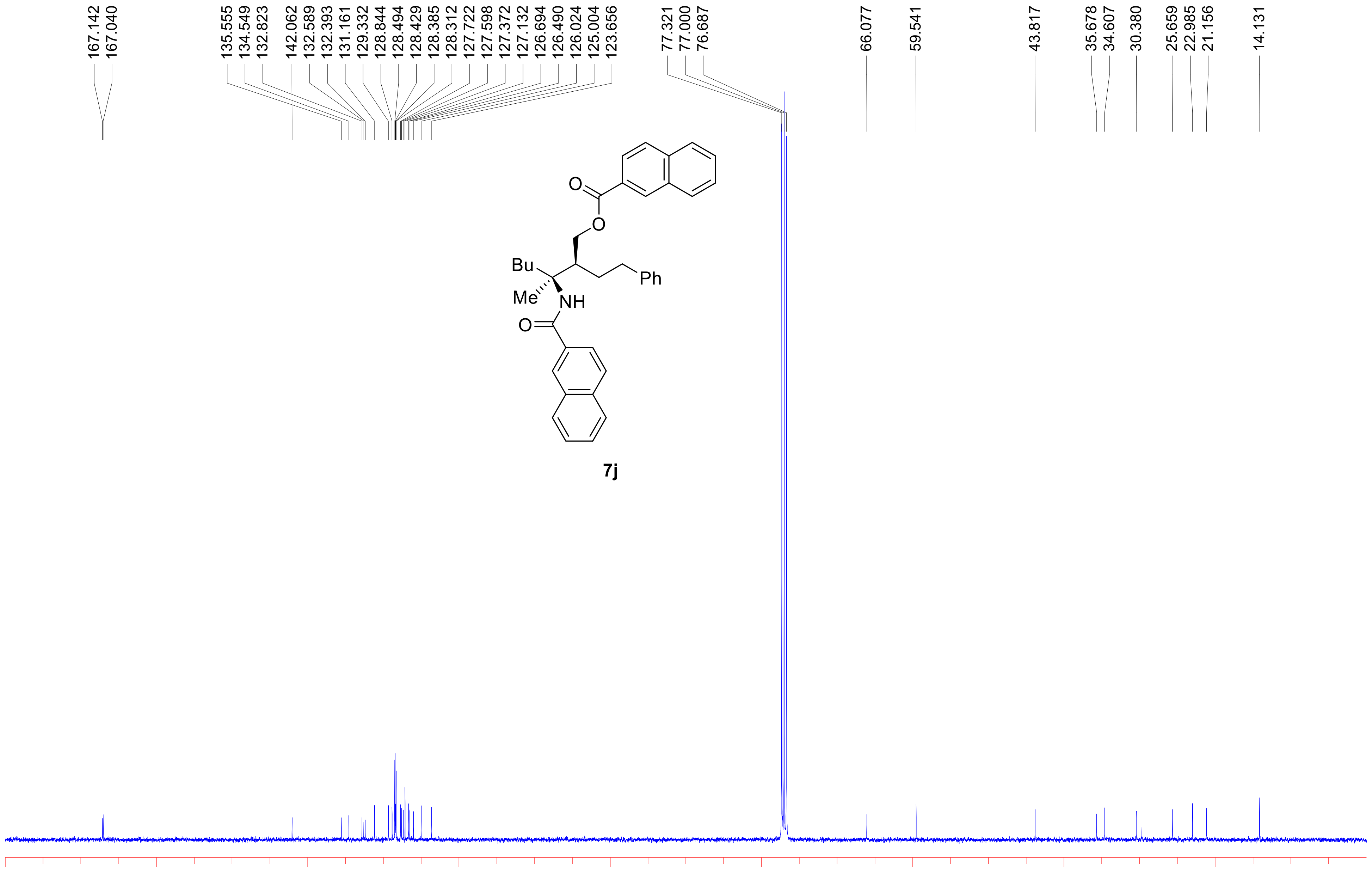
167.142
167.040
135.555
134.549
132.823
142.062
132.589
132.393
131.161
129.332
128.844
128.494
128.429
128.385
128.312
127.722
127.598
127.372
127.132
126.694
126.490
126.024
125.004
123.656
77.321
77.000
76.687
66.077
59.541
43.817
35.678
34.607
30.380
25.659
22.985
21.156
14.131
O
O
Bu
Me
NH
Ph
O
7j
160
140
120
100
80
60
40
20
PPM
